# Recombination Dynamics of a Human Y-Chromosomal Palindrome: Rapid GC-Biased Gene Conversion, Multi-kilobase Conversion Tracts, and Rare Inversions

**DOI:** 10.1371/journal.pgen.1003666

**Published:** 2013-07-25

**Authors:** Pille Hallast, Patricia Balaresque, Georgina R. Bowden, Stéphane Ballereau, Mark A. Jobling

**Affiliations:** Department of Genetics, University of Leicester, Leicester, United Kingdom; Aarhus University, Denmark

## Abstract

The male-specific region of the human Y chromosome (MSY) includes eight large inverted repeats (palindromes) in which arm-to-arm similarity exceeds 99.9%, due to gene conversion activity. Here, we studied one of these palindromes, P6, in order to illuminate the dynamics of the gene conversion process. We genotyped ten paralogous sequence variants (PSVs) within the arms of P6 in 378 Y chromosomes whose evolutionary relationships within the SNP-defined Y phylogeny are known. This allowed the identification of 146 historical gene conversion events involving individual PSVs, occurring at a rate of 2.9–8.4×10^−4^ events per generation. A consideration of the nature of nucleotide change and the ancestral state of each PSV showed that the conversion process was significantly biased towards the fixation of G or C nucleotides (GC-biased), and also towards the ancestral state. Determination of haplotypes by long-PCR allowed likely co-conversion of PSVs to be identified, and suggested that conversion tract lengths are large, with a mean of 2068 bp, and a maximum in excess of 9 kb. Despite the frequent formation of recombination intermediates implied by the rapid observed gene conversion activity, resolution via crossover is rare: only three inversions within P6 were detected in the sample. An analysis of chimpanzee and gorilla P6 orthologs showed that the ancestral state bias has existed in all three species, and comparison of human and chimpanzee sequences with the gorilla outgroup confirmed that GC bias of the conversion process has apparently been active in both the human and chimpanzee lineages.

## Author Summary

The sex-determining role of the human Y chromosome makes it male-specific, and always present in only a single copy. This solo lifestyle has endowed it with some bizarre features, among which are eight large DNA units constituting about a quarter of the chromosome's length, and containing many genes important for sperm production. These units are called palindromes, since, taking into account the polarity of the DNA strands, the sequence is the same read from either end of the unit. We investigated the details of a process (gene conversion) that transfers sequence variants in one half of a palindrome into the other, thereby maintaining >99.9% similarity between the halves. We analysed patterns of sequence variants within one palindrome in a set of Y chromosomes whose evolutionary relationships are known. This allowed us to identify past gene conversion events, and to demonstrate a bias towards events that eliminate new variants, and retain old ones. Gene conversion has therefore acted during human evolution to retard sequence change in these regions. Analysis of the chimpanzee and gorilla versions of the palindrome shows that the dynamic processes we see in human Y chromosomes have a deep evolutionary history.

## Introduction

**Figure 1:**
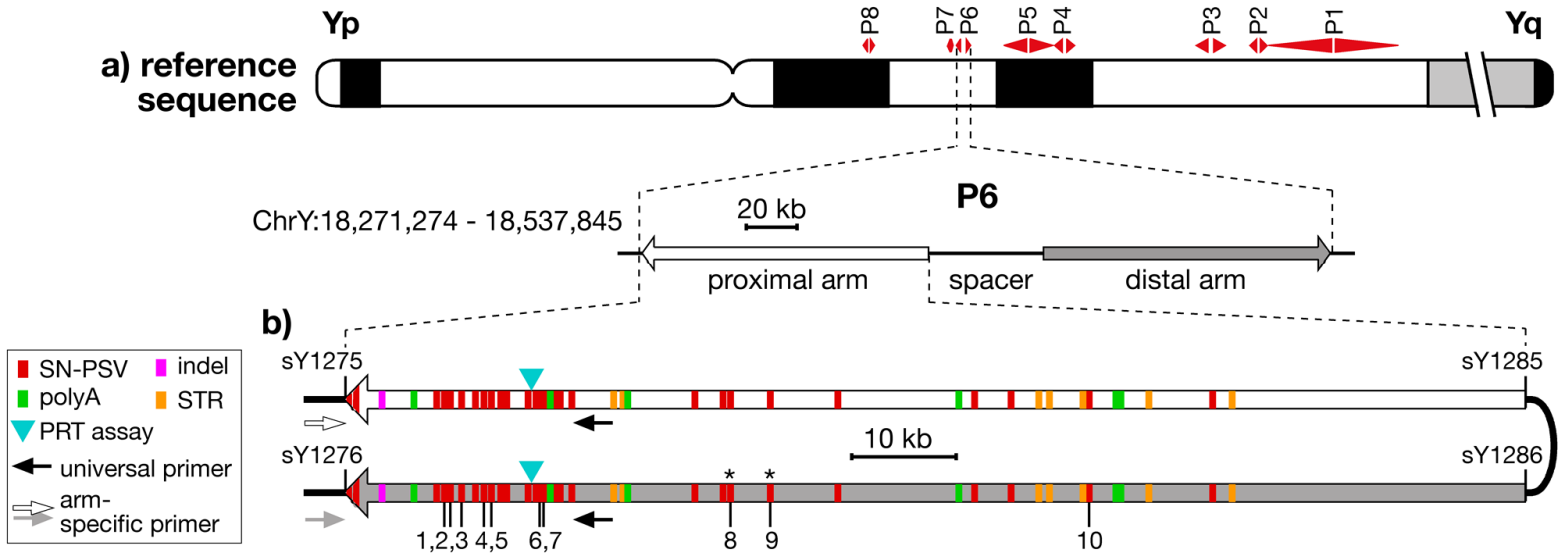
Location and structure of palindrome P6, showing positions of PSVs analysed. a) Idiogram of Y chromosome, showing positions of the 8 palindromes, with structure and coordinates (in GRCh37) of P6 below. b) Position and nature of the differences between the arms of P6, indicating the 10 SN-PSVs analysed, and the positions of PCR primers used in arm-specific amplifications. STSs marking the arm boundaries are also shown (with ‘sY’ prefixes). Asterisks indicate the two SN-PSVs identified from a haplogroup O3a chromosome.

The male-specific region of the human Y chromosome (MSY) is constitutively haploid, yet contains a high proportion (∼35%) of pseudo-diploid duplicated regions, eight of which are arranged as large inverted repeats (‘palindromes’, known as P1 - P8; Figure 1a), with arms in most cases separated by non-duplicated spacers [1]. The arms of each palindrome are >99.9% similar in sequence due to the homogenising effect of gene conversion. Human-chimpanzee sequence divergence within palindrome arms is significantly lower than that within spacers, and compared to the MSY (non-palindrome, non-spacer) average [2]. This suggests that gene conversion since speciation may have been directional, tending to return new mutations that arise within arms to their ancestral states. Most palindrome arms are enriched in testis-specific genes, important in spermatogenesis, and the suggestion has been made that directional gene conversion between pseudo-diploid copies may protect these genes against evolutionary decay [2].

It is becoming increasingly recognised that such palindromic structures are far from being a peculiarity of great ape Y chromosomes, but have more general biological significance as a feature of independently arising constitutively haploid sex chromosomes in other mammals [3]–[5], birds [6], [7] and insects [8], as well as of the mammalian X chromosome [9], [10], which is haploid in males. Yet despite this general importance, and despite some theoretical analyses of palindrome evolution [11], [12], little is known about the dynamics of conversion within these remarkable structures.

**Figure 2:**
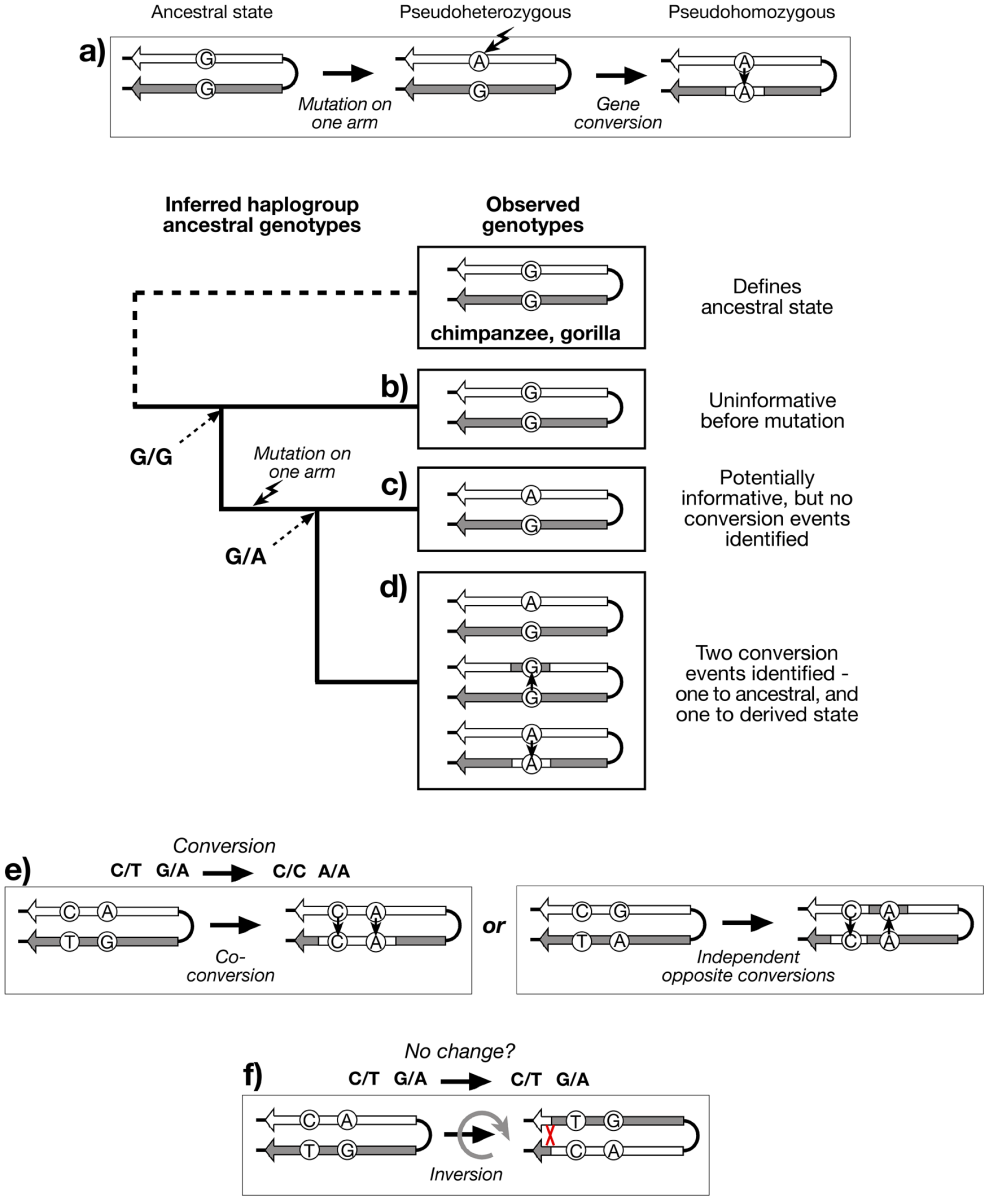
Recognition of gene conversion, co-conversion and inversion events. a) Existence of three genotypes at a hypothetical PSV indicates that gene conversion has taken place, if recurrent mutation is neglected. Genotyping the PSV in a phylogenetic context, and applying the principle of maximum parsimony, allows the recognition of: b) Haplogroup descending from an ancestor in which the PSV mutation has not yet arisen (G/G), and is therefore uninformative; c) Haplogroup descending from an ancestor in which the PSV mutation has arisen (G/A), but shows no variation, and therefore no evidence for gene conversion; d) Haplogroup descending from an ancestor in which the PSV mutation has arisen, and shows evidence of at least two bidirectional conversion events (G/G and A/A); e) Recognition of co-conversion of more than one PSV requires ‘phase’ information, as does (f) recognition of inversions.

Large stretches of sequence identity between palindrome arms represent compelling evidence for rapid gene conversion, and yet, paradoxically, provide a barrier to understanding the dynamics of the conversion process. Conversion rate, tract length, and directionality cannot be examined when there are no sequence differences (paralogous sequence variants; PSVs) between arms that might allow specific conversion events to be recognised. However, when a PSV does exist (e.g. the ‘pseudoheterozygous’ state G/A), then the observation in other chromosomes of the two other possible genotypes, the ‘pseudohomozygous’ G/G and A/A, indicates that gene conversion must have occurred within the history of the examined sequences (Figure 2a), assuming that recurrent mutation can be neglected. Such an observation tells us nothing about how many independent conversion events underlie the three genotypes. But the availability of a detailed and robust Y phylogeny, defined by stable single nucleotide polymorphisms (SNPs) outside the palindromic regions, allows the evolutionary relationships of palindrome sequences to be known, and genotyping within this phylogenetic context can then provide an estimate of the minimum number of conversion events (Figure 2b–d).

Genotyping PSVs within a phylogenetic context provides evidence for past gene conversion events, but the resulting genotypes (pursuing the analogy of diploidy) are ‘unphased’ - we do not know which allele of a PSV lies on which palindrome arm. Because of the high degree of sequence identity and the scarcity of PSVs within palindromes, phasing is technically challenging, but nonetheless important if we are to gain an understanding of the lengths of conversion tracts, suggested by sets of co-converted adjacent PSVs (Figure 2e). If phased PSV data for palindromes were available, it would also be possible to address an additional important aspect of the dynamics of these structures: the ratio of non-reciprocal exchanges (conversions) to reciprocal exchanges (inversions; Figure 2f).

Here, we analyse paralogous sequence variants (PSVs) within the arms of human palindrome P6, taking the approaches outlined above. We demonstrate through a phylogenetic analysis of conversion events five cardinal features of the palindrome conversion process during human evolution: (i) the conversion process has been rapid throughout the evolution of modern human Y-chromosomal lineages; (ii) it shows significant bias to the fixation of GC base pairs; (iii) it is biased towards the retention of ancestral states of PSVs; (iv) conversion tracts can encompass several kilobases; and (v) despite the high frequency of recombination events within palindrome arms, these resolve overwhelmingly via non-reciprocal exchange (conversions) rather than reciprocal exchange (inversions). We then extend our findings to deeper evolutionary time by determining the sequence of gorilla P6, showing that ancestral state bias has existed in the gorilla lineage as well as in humans and chimpanzees, and allowing us to ascertain the direction of evolutionary changes in the human and chimpanzee lineages, revealing a possible long-term GC conversion bias.

## Results

### Palindrome P6 as a study system

To study gene conversion dynamics we first sought a segment of a palindrome carrying a suitable number and density of PSVs. Arm-to-arm alignment of the reference sequence (belonging to haplogroup R1b1b2* [13]) for palindrome P6 (Figure 1b) demonstrated a 99.97% sequence similarity between its 110-kb arms, but revealed a total of 49 discrete sequence differences, which we supplemented with two additional single-nucleotide PSVs identified from the sequencing of a flow-sorted Y chromosome from a different source, belonging to haplogroup O3a [14]. Twenty-nine of these represent simple single-nucleotide PSVs (SN-PSVs) that are unlikely to undergo mutational reversion or recurrence. Furthermore, 16 SN-PSVs lie within 20 kb of the outer arm boundaries, potentially allowing arm-specific PCR anchored in flanking single-copy DNA to determine in which arm a particular variant lies (‘phasing’). Two additional factors favour P6: chimpanzee and gorilla orthologs exist that allow the ancestral state of its PSVs to be determined; and P6 lacks protein-coding genes, meaning that direct effects of natural selection are less likely than for other palindromes.

We sought to design reliable typing assays for all SN-PSVs, and this was successfully accomplished (see Materials & Methods) for ten, indicated in Figure 1b.

**Figure 3:**
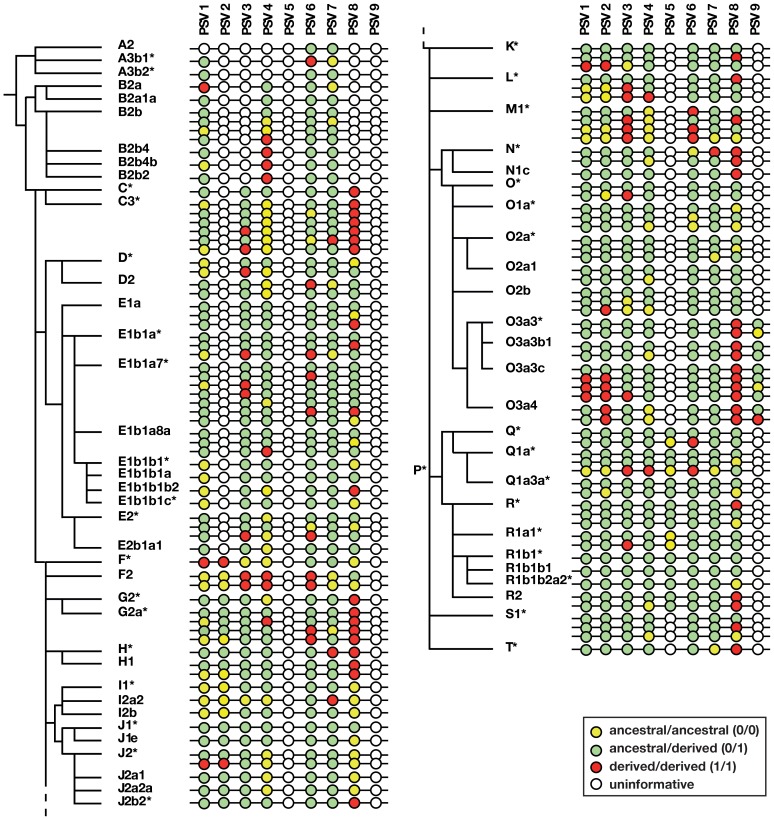
Phylogenetic analysis of gene conversion events within P6. Adjacent to the Y phylogeny based on binary markers is shown a schematic representation of allelic states of nine informative PSVs (PSV10 is omitted because it is invariant). Note that the number of haplotypes shown within each haplogroup is less than the number of samples genotyped. Circles represent PSVs with inter-PSV distances not to scale, and the colours of circles indicate uninformative, pseudoheterozygous and pseudohomozygous PSVs, as shown in the key.

In order to identify gene conversion events between the arms of P6, we required a set of Y chromosomes for which detailed phylogenetic relationships were well established. We exploited the availability of the CEPH-Human Genome Diversity Project (HGDP) panel of DNA samples [15], which has good global coverage and for which data were available for 184 Y-chromosomal binary markers ([16]–[18]; www.cephb.fr/en/hgdp/), supplementing this by typing an additional 23 binary markers, to define a total of 63 haplogroups. The tree thus defined, the markers, the haplogroup nomenclature and the sources of data are shown in Figure S1. A simplified version of the tree is shown in Figure 3.

### PSV genotypes and gene conversion bias within P6

The ten PSVs were analysed in a subset of 378 of the 684 HGDP male samples, chosen to cover the haplogroup diversity of the sample set. Each PSV genotype was recorded as pseudoheterozygous (e.g. G/A) or pseudohomozygous (e.g. G/G or A/A), and, by comparison to the orthologous sequences in chimpanzee [19] and gorilla ([20], and our own gorilla sequence – see below) each PSV allele coded as ancestral (0) or derived (1).

Figure 3 illustrates the patterns of variation observed in the sample, and full details are given in Table S1. Some PSVs (e.g. PSV6) are variable across all haplogroups, suggesting that the variant arose at the root of the Y phylogeny. Others show variability only in specific haplogroups (hg), suggesting (assuming maximum parsimony, and no recurrent mutation) that they arose in their founders (e.g. PSV2 in hgF, PSV5 in hgP, and PSV9 in hgO3a). PSV10 was monomorphic in all 378 cases tested, suggesting that it represents a recently arising variant. For any haplogroup, we can deduce whether the founder was pseudoheterozygous (0/1); when this is so, the finding of pseudohomozygous states (0/0 or 1/1) among chromosomes within the haplogroup indicates that conversion must have occurred (Figure 2d). Treating each PSV as an independent site of gene conversion, it is thus possible to both count the total number of conversion events, and to ask what proportion of these are conversions to the ancestral state (i.e. 0/1 to 0/0), or the derived state (i.e. 0/1 to 1/1).

This analysis identified a total of 146 converted SN-PSVs, of which 86 represent conversion to the ancestral, and 60 to the derived state (Tables S1 and S2). This difference is statistically significant (p = 0.0314; Chi-square test), which is consistent with published observations based on human-chimpanzee comparisons [2].

We can also ask if there is a tendency towards the fixation of GC base-pairs rather than AT base-pairs: this is so-called GC-biased gene conversion, and results from a bias in the repair of AC and GT mismatches that form in heteroduplex recombination intermediates [21]. Of the 146 converted SN-PSVs, some are uninformative because they involve transversions (from CG to GC, or AT to TA), but among the 79 informative cases 59 involve the fixation of GC, and 20 of AT (p = 1.1×10^−5^; Chi-square test). From these observations, gene conversion among the studied PSVs appears to be strongly GC-biased, and slightly but significantly biased towards the retention of ancestral state.

Having counted the number of observed gene conversion events in our dataset, we can estimate an average rate of gene conversion by dividing by the number of generations encompassed in the phylogeny that relates the studied Y chromosomes (Materials & Methods). For a 25-year generation time, this yields a rate of 2.9–8.4×10^−4^ events per generation.

### Infrequent inversions within P6

The above analysis provides evidence of a highly active gene conversion process within P6: but does the frequent formation of recombination intermediates that this implies also give rise to frequent inversions of the palindrome arms? As explained in the Introduction (Figure 2f), identification of inversion events requires the palindrome arms to be ‘phased’ at pseudoheterozygous sites. In order to do this, an arm-specific long-range PCR approach was developed, using one universal primer binding within the arm, and another binding to a distal-arm-specific region outside the outer palindrome boundary. This generated a product of ∼18.9 kb incorporating seven of the studied PSVs (PSV1–7) that could then be typed in an arm-specific manner, thus determining their phase.

**Figure 4:**
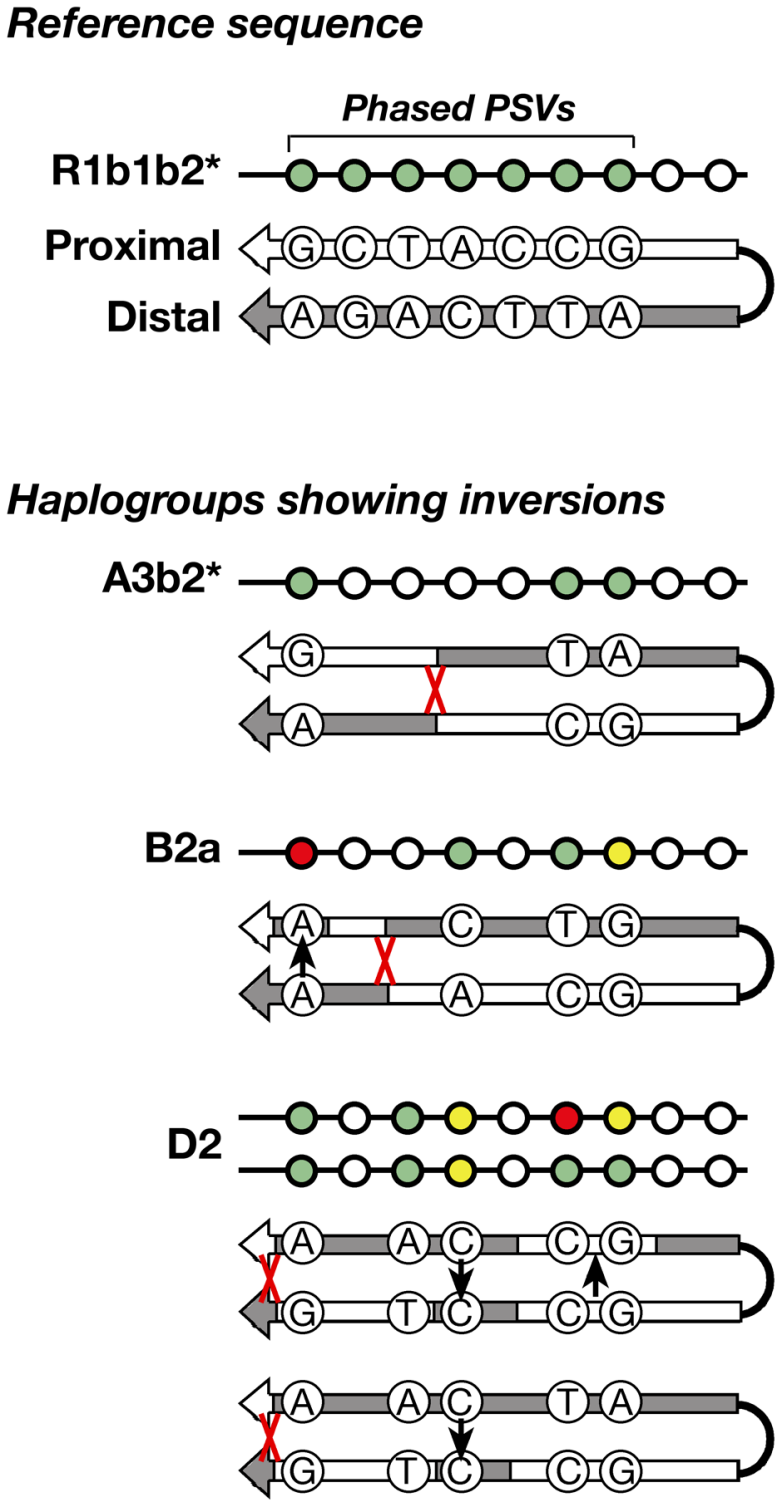
Schematic representation of the inversions identified within three haplogroups. The approximate position of each inversion is indicated by a red cross.

Arm-specific haplotypes from 83 selected DNA samples representing all of the haplogroups were compared to the Y-chromosome reference sequence, whose phase is known from BAC clone sequencing [1]. All but five samples were found to have identical phase to the reference sequence (Figure 4) at informative (pseudoheterozygous) sites; this corresponds to just three independent inversion events, in haplogroups A3b2*, B2a, and D2. Where phase information is available for several chromosomes within a haplogroup, these are always concordant – in other words, inversions are rare. This strong preponderance of conversion over inversion allows us to infer the phase of unphased chromosomes within haplogroups.

Among the 83 phased chromosomes, the three inversion events compare with 56 gene conversion events (assuming each converted PSV represents a single event). In the same set of chromosomes, and under the same assumptions, the per-generation rate of inversion is 1.36–1.72×10^−5^, compared to 2.54–3.21×10^−4^ for conversion. The latter rate differs from that given in the section above due to the smaller number of chromosomes phased and analysed here.

### Co-conversion of PSVs

All of the analysis above assumes that PSVs are independently converted, but from simple inspection of the behaviour of the adjacent PSVs 1 and 2, separated by only 81 bp, it is evident that co-conversions must be occurring: for example, of the 14 instances where conversion affecting PSV1 and PSV2 is informative, 11 involve apparent co-conversion of both markers (e.g. in hgM1*; Figure 3). We therefore wished to examine co-conversion more systematically, and the phasing information allows us to do this (as shown in Figure 2e). The true number of co-conversion events is impossible to estimate, because the apparent co-conversion of adjacent variants could actually reflect the sum of two independent events. However, we can estimate the minimum number of co-conversions that explain the observed data: first, we identify adjacent pairs of pseudohomozygous PSVs within a haplogroup whose founder is deduced to be pseudoheterozygous for the same PSVs; and second, to exclude independent opposite conversions as an explanation (Figure 2e), we count only those PSV pairs that match a single arm-specific haplotype of the reference sequence. We then assume that these reflect a single conversion tract. On this basis, 49 of the 107 (45.8%) individual conversion events among ‘phased’ PSVs (1–7) can be explained by a minimum of 18 co-conversion tracts.

We cannot arrive at a useful estimate of maximum co-conversion tract length, because most tracts are not flanked by informative genotyped markers that would indicate their outer limits. However, we can estimate minimum lengths by considering the distance between the outer converted markers within each tract. The mean value of these minimum estimates is 2068 bp: this is much longer than most recorded gene conversion events, which are typically a few hundred bp in length, and rarely exceed 1 kb [22].

Some apparent co-conversion tracts are very long indeed. For example, within hgQ1a* we observe PSVs 1–8 in the pseudoheterozygous state, but also a case where the first seven of these variants are pseudohomozygous. This case seems unlikely to have arisen as a result of a series of consecutive small-scale conversion events, because the allelic state of the variants matches a single arm-specific haplotype in the same haplogroup. An alternative trivial explanation is that one arm in this chromosome has been lost by deletion, and that the PSVs are being observed in a pseudohemizygous, rather than pseudohomozygous state. To eliminate this possibility we confirmed that two arms were present, and were of the expected length, using two methods: a paralog-ratio test (PRT) to measure the copy-number of the palindrome arm with respect to a reference sequence on the X chromosome; and a long-PCR assay specific for each arm in turn. The most parsimonious explanation for the observed genotype in this chromosome is therefore a massive conversion event that spans at least 9023 bp (the distance between PSVs 1 and 7 on the proximal arm).

The analysis carried out above, to detect biases in gene conversion towards retention of the ancestral state and fixation of GC base-pairs, treated each converted nucleotide as an independent replicate in a statistical test. However, since we have inferred that co-conversions occur, some variants are not independent; we therefore repeated both tests after removing the putative co-conversion events. In both cases, the statistical significance of the bias is retained (Table S2).

### Deeper evolutionary history of conversion bias

In order to study the deeper history of gene conversion activity and its impact on palindrome evolution, we required an outgroup to human and chimpanzee P6. A high-quality MSY sequence is available for rhesus macaque that contains three palindromes, but a P6 ortholog is not among them [3]. A gorilla Y-chromosome reference sequence is not yet available, but this species is known to carry both P6 arm-spacer boundaries with almost identical sequence to human and chimpanzee [2]. We constructed a partial sequence of gorilla P6 by merging Illumina paired-end sequencing data from two whole-genome-sequenced male gorillas [20] and from an independent male analysed in a sequence capture experiment. A total of 88,031 bp of merged gorilla P6 arm and 31,206 bp of gorilla spacer were assembled using the human Y-chromosome sequence as a reference. These data represent 80% of the human proximal arm and 67.5% of human spacer. The presence of both P6 arms in gorilla is confirmed by the fact that the mean coverage of proximal arm for all three gorillas is approximately twice that of the spacer (Protocol S1).

Pairwise alignments between human, chimpanzee and gorilla show that nucleotide divergence in all three comparisons is highly significantly reduced in the arms of P6 compared to spacer (Table 1). This is consistent with previous results [2] showing a similar pattern when comparing segments of Y-chromosome palindromes between human and chimpanzee. Our findings therefore confirm that the processes influencing palindrome evolution are active in both human and chimpanzee lineages, and also probably active in gorilla.

**Table 1:** Inter-specific sequence divergence in arms and spacers of palindrome P6.

Species comparison	Region of P6	Ungapped length (bp)	No of nt substitutions	Divergence (%)	P-value (arm vs spacer)^a^
**Human vs chimpanzee**					
	Arm	104230	1497	1.44	1.52×10^−15^
	Spacer	45959	919	2.00	
**Human vs gorilla**					
	Arm	88031	1726	1.96	5.04×10^−8^
	Spacer	31206	773	2.48	
**Chimpanzee vs gorilla**					
	Arm	84096	1820	2.16	6.04×10^−7^
	Spacer	31097	828	2.66	

a 2×2 contingency table, Chi-square test with Yates correction.

Availability of an outgroup sequence also allows possible long-term GC-bias to be examined in human and chimpanzee lineages. We used a phylogenetic approach to study nucleotide replacements in palindrome arms and spacer. Since the universally low (∼0.02%) arm-to-arm divergence suggests that conversion is highly active within each species, all replacements found in arms can be assumed to be due to mutation followed by gene conversion; in spacers the divergence is expected to arise solely from mutational processes. From the alignment of human, chimp and gorilla P6 sequences (Dataset S1), we determined the types of all fixed differences, noting G or C (S) nucleotides that changed to A or T (W) nucleotides, and vice versa. We also determined the evolutionary direction of each of these fixed differences: if a nucleotide was identical between chimpanzee and gorilla but divergent in human, a replacement on the human lineage was assumed; if human and gorilla were identical, a replacement in chimpanzee was assumed.

Table 2 summarises the numbers and types of nucleotide replacements in both the human and chimpanzee arms and spacers. In the arm, the proportion of W to S changes slightly exceeds that in the spacer, but the proportion of S to W changes is significantly lower than that in the spacer. Furthermore, in human the proportion of W to S and S to W changes in the arm are approximately equal, while in the spacer S to W changes significantly predominate (as has been observed previously for substitutions not associated with gene conversion [23]). These observations indicate a relative bias towards W to S changes in arms. In chimpanzee P6, the proportion of W to S changes in the arm is significantly higher than that of S to W. In order to eliminate the potential influence of hypermutable CpG dinucleotides, all sites in CpG, TpG, or CpA sequences were removed from the raw sequence alignment, and the comparisons repeated. In both human and chimpanzee P6, the differences between arm and spacer remain. These striking differences in substitution patterns in arms and spacer seems likely to reflect the preferential fixation of GC base-pairs in arms due to the action of GC-biased gene conversion.

**Table 2:** Patterns of P6 nucleotide replacements in the human and chimpanzee lineages.

		Total no. replacements	W to S changes/total AT nt (%)	Ratio of changes to W/to S^a^	S to W changes/total GC nt (%)
**Before dinucleotide removal**					
Human	Arm	487	235/51201 (0.46)	1.07	161/32892 (0.49)
	Spacer	238	72/19687 (0.37)	2.97**	124/11404 (1.09)
	Arm: spacer ratio^a^		1.25		0.45**
Chimpanzee	Arm	659	366/51201 (0.71)	0.77**	181/32892 (0.55)
	Spacer	297	110/19687 (0.56)	2.15**	137/11404 (1.20)
	Arm: spacer ratio^a^		1.28*		0.46**
**After dinucleotide removal**					
Human	Arm	180	80/38071 (0.21)	1.83**	74/19215 (0.39)
	Spacer	100	24/14935 (0.16)	5.28**	56/6596 (0.85)
	Arm: spacer ratio^a^		1.31		0.45**
Chimpanzee	Arm	270	135/38071 (0.35)	1.29	88/19215 (0.46)
	Spacer	134	43/14935 (0.29)	3.95**	75/6596 (1.14)
	Arm: spacer ratio^a^		1.23		0.40**

a 2×2 contingency table, Chi-square test with Yates correction.

* p-value<0.05.

** p-value<0.01.

## Discussion

In this study we have used a phylogenetic approach to the diversity of sequences within a Y-chromosomal palindrome, P6, to illuminate the dynamic processes of recombination that distinguish these remarkable structures.

Analysis of a set of ten PSVs in 378 chromosomes has revealed 146 individual PSV conversion events in the Y phylogeny, and confirms that gene conversion is an ongoing and rapid process. Our findings add to the body of evidence showing that, despite its exemption from the otherwise ubiquitous process of meiotic crossing over, the MSY is highly active in gene conversion, involving not only palindromes [2], [24], but also widely separated direct repeats [25] and minisatellite arrays [26]. As well as intrachromosomal conversions, gametologous regions on the Y have been shown to exchange sequences with the X chromosome in humans [27], [28] as well as other mammals [29]–[32].

### Basic parameters of gene conversion

We observe a conversion rate of 2.9–8.4×10^−4^ events per generation among the 10 surveyed PSVs. This equates to a per-PSV rate of 2.9–8.4×10^−5^ events per generation, though this represents a minimum estimate, since not all of the PSVs are informative in all studied chromosomes. Based on a measure of inter-arm divergence and an estimate of the base-substitution rate, Rozen et al. [2] estimated a conversion rate per generation per nucleotide in Y palindromes of 2.2×10^−4^.

Although gene conversion tracts several kilobases in length occur frequently in yeast [33], in mammals tracts are short, typically ranging from a few tens of base pairs [34] to 1 kb [22]. In palindrome P6, we infer minimum gene conversion tract lengths up to 9023 bp with mean minimum length of 2068 bp. These lengths do not represent direct measurements, and it remains possible that the inferred patterns of long conversion tracts could be created by multiple independent events. However, the longest inferred tract, including 7 PSVs, would require several independent events all in the same direction (from proximal to distal arm), so the most parsimonious explanation is a single event. It is possible that long conversion tracts are a typical characteristic of palindromes, but this remains to be tested by future studies.

### Resolution of recombination intermediates as inversions

Recombination is initiated by double-strand breaks (DSBs), and their repair can result in either reciprocal crossover, or non-reciprocal conversion. In considering the effects of these different pathways in P6, we need to differentiate between inter- and intramolecular events since, while conversion between or within chromatids will have the same molecular outcomes, this is not the case for crossover. Inter-chromatid crossover is expected to lead to an isodicentric chromosome and an acentric fragment, both of which are selected against. For example, 7/8 human palindromes are involved in crossover events between sister chromatids resulting in large-scale rearrangements in patients with disorders ranging from spermatogenic failure to sex reversal and Turner syndrome [35]. By contrast, intra-chromatid crossover will lead to simple inversion of palindrome arms, which seems unlikely to have strong effects on fitness. As an example, crossover between IR3 inverted repeats on Yp, resulting in apparently asymptomatic inversion, has occurred at least twelve times in the history of the Y phylogeny [36]. This different consequences of the two pathways means that while observed conversions reflect both inter- and intramolecular events, observed inversions are the result of intra-chromatid events only, and this complicates the interpretation of conversion: crossover ratios.

Phylogenetic detection of intra-chromatid crossovers leading to palindrome inversions is possible if the phase of the PSVs is known. Phasing of seven of the studied PSVs, located within the first ∼19 kb from the outer palindrome boundaries provides evidence of only three independent inversions among the studied chromosomes (Figure 4). The deduced rate of inversion, 1.36–1.72×10^−5^ per generation, compares to a published rate of 2.3×10^−4^ for the IR3 inverted repeats [36]. Notably, we have ascertained only those inversions with breakpoints occurring in the outer ∼16% of the arms of P6, whereas the published study was able to ascertain all intra-chromatid inversions by determining the orientation of markers between the IR3 repeats. Our finding of 56 conversion events in the same chromosome set indicates that observed recombination events in P6 are strongly biased towards conversions rather than crossovers. Among the studied chromosomes, intra-chromatid inversions are comparatively well ascertained, because a crossover in the interval between any pair of informative PSVs will be detected reliably. Conversion, however, is under-ascertained because it is only observed when it transfers a particular informative PSV. The scarcity of PSVs means that the observed conversion: intra-chromatid crossover bias is actually an underestimate of the true value. Additional uncertainty is introduced by our inability to accurately identify co-conversion.

A bias towards non-crossovers is commonly observed in recombination analysis. According to cytological studies the repair of only 10% of DSBs in mammals results in crossovers, while the remainder are assumed to be repaired as non-crossovers [37]. Most mammalian data on conversion: crossover ratios come from studies of meiotic recombination hotspots in humans and mice. The ratio varies significantly between different human hotspots (from 2.7∶1 at hotspot DNA3 to <1∶12 at the β-globin hotspot); there are also considerable differences among individuals, driven in part by variation in trans-acting factors [38]–[41].

In comparing MSY gene conversion with conversion affecting other chromosomes, its singular status as a constitutively haploid chromosome must be remembered. As discussed above, both inter- and intra-chromatid conversion can occur, but neither of these processes is linked with the highly regulated ‘normal’ processes of synapsis and meiotic crossing over. Many questions therefore remain about the timing and mechanism of MSY conversion processes.

### GC-bias in palindrome gene conversion

In a number of organisms recombination has been associated with GC-bias arising from biased repair of mismatches in heteroduplex DNA [42]. Consistent with this, we found evidence of highly statistically significant GC-bias among the P6 gene conversion events within the Y phylogeny.

We also asked whether GC-bias in gene conversion had a deeper evolutionary history by comparing the patterns of nucleotide replacements among human, chimpanzee and gorilla P6 sequences. Spacers show a statistically significantly greater proportion of replacements of S nucleotides by W nucleotides than arms do (Table 2). This is true for both human- and chimpanzee-specific nucleotide replacements. It is possible that these differences could be due to regional variation in GC-content, repeat content, mutation rates or some other factors, but the observed replacement patterns in palindrome arms are nonetheless consistent with the action of GC-biased gene conversion. We might expect the long-term action of such bias to lead to elevated GC-content in arms compared to spacers. For P6, this is the case (Table S6): 38.8% (arms) is significantly greater (p = 2.7×10^−11^; Chi-square test) than 37.0% (spacer). We can make the same comparisons for other palindromes, setting aside P1 and P2, which have very large arms and very small spacers. P3 also shows a significant elevation of GC-content in its arms (p = 1.0×10^−56^), while P4, P5, P7 and P8 show no significant differences; the pattern is therefore complex, but notably none of these palindromes shows significantly higher GC-content in spacer compared to arms. The observed differences could in principle reflect the enrichment of protein-coding genes in palindrome arms compared to spacers; however, the observed pattern persists when the genes are removed (Table S6).

### Apparent bias to ancestral state in gene conversion

Our comparisons of human, chimpanzee and gorilla P6 sequences concur with previous observations [2] in revealing significantly lower inter-specific divergence among arms than among spacers, in all three possible comparisons (Table 1). This suggests either that the rate of initial mutation in arms is lower than that in spacers, or that gene conversion is acting to preferentially return new mutations arising in one arm to the ancestral state, via conversion from the unmutated arm. Our observation that individual gene conversion events among human Y chromosomes are significantly biased towards retention of the ancestral states of PSVs tends to support the second explanation. Natural selection acting directly on the PSV sites seems an unlikely explanation for the bias: examination of ENCODE [43] data (as represented in the UCSC Genome Browser; April 2013) shows P6 to be devoid of functionally significant elements, apart from a 107-bp snRNA gene in the arms ∼20 kb proximal to the inner arm boundary. There is no evidence for functional elements overlapping the variants tested. An alternative explanation is that the ancestral state bias emerges from the GC-bias. Notably, of the six PSVs that are informative about GC-bias acting at individual sites, five have a G or C nucleotide as their ancestral state. Whether GC-bias provides a more general explanation for the conservation of palindrome sequences will require more data on a larger number of palindrome sequence variants.

Y-chromosomal palindromes are not alone in showing apparent ancestral-state bias in conversion: comparison of human and chimpanzee orthologs of an X-chromosomal palindrome [44] also display significantly reduced interspecific divergence in arms compared to spacers. This bias in conversion may therefore be a general property of palindromic repeats. Its consequence is that palindromes are ‘hard wired’ for conservation; although this will be largely beneficial because most mutations are deleterious, it may also ultimately limit adaptability of genes in palindromes by limiting the opportunity for fixing beneficial mutations.

### Future developments

Our understanding of the molecular evolution of the Y chromosome would be greatly improved by the availability of additional accurate sequences both from non-human primates and humans. In principle, next-generation sequencing technologies offer the opportunity to generate such sequences, but in practice the complex repetitive structure of the Y chromosome means that sequence assembly is impossible with current methods. Successful generation of useful Y-chromosome sequences from humans and other species [1], [3], [15], [19] has required shot-gun sequencing of assembled tiling arrays of BAC clones, an expensive and laborious process. An additional problem is that genome sequencing projects in non-human primates focus on females, in order to provide good coverage of the X chromosome. The structures of palindromes, the phase of variants within them, and gene conversion tract lengths will be illuminated by the advance of third-generation sequencing methods that have very long read lengths, and also high-throughput haplotyping of single sperm molecules, a method that has already proved successful in identifying the longest known allelic gene conversion tract of 22 kb [45].

## Materials and Methods

### DNA samples and Y haplotyping

We analysed a total of 378 male samples chosen from the CEPH-HGDP Cell Line Panel (Table S1) [15]. Choice was motivated by existing information on haplogroup, and practicality: we wanted to ensure representation of several members of each known haplogroup in order to detect gene conversion events (Figure 2), but to avoid analysing all 684 males in the panel due to the laborious nature of PSV typing and phasing.

Y-chromosome binary polymorphism data for HGDP samples were compiled as follows: 145 SNPs from CEPH 2011 (www.cephb.fr/en/hgdp/ - data supplement 10), 37 from Shi et al. 2010 [16] and Peter de Knijff (unpublished observations), 10 from Li et al. 2008 [18] and three from Sengupta et al. 2006 [17]. In addition, 23 SNPs (M112, M119, M150, M182, M217, M223, M231, M267, M285, M287, M3, M32, M35, M38, M6, M75, M78, M8, P15, P2, P37, P45 and P312) were typed as part of a GoldenGate custom genotyping assay (see section below). Eleven samples representing haplogroup K*(xL,M,N,O,P) [16] were typed for M254 and P204 using published PCR primers [13] and Sanger sequencing.

The whole dataset is described in Figure S1 and Table S1. For the phylogeny, the total of 200 mutational events gave rise to 122 possible Y-chromosome haplogroups, of which we observed 63 among the 378 samples analysed. Haplogroup nomenclature is as described [13], with shorthand names for some haplogroups, as described in Table S1.

There were two inconsistencies between data sources: (i) The phylogenetic relationships of markers P7 and M169 within hgB2 were consistent with the data of [46] rather than the original description [13]; (ii) Four samples (HGDP numbers 541, 542, 553 and 662) are listed in the data of [16] as belonging to hgK(xL,M1,NO,P), with the hg-M1-defining marker M106 ancestral; however, these same samples are listed under CEPH 2011 (www.cephb.fr/en/hgdp/ - data supplement 10) as derived for both M106 and the phylogenetically equivalent marker M189. Given that two markers are in agreement in this dataset, we regard them here as hgM1 chromosomes.

### Ethics statement

This study uses human DNA samples from the CEPH-HGDP panel, a widely available anonymised set of lymphoblastoid cell-lines (LCLs). The original paper describing this panel [15] states that the blood specimens that served as sources of the LCLs were freely donated under conditions of informed consent and confidentiality by reviewing consent forms, institutional review board approvals, or detailed reports from those who organised collections.

### Genotyping of P6 PSVs

The ten typed PSVs were labelled PSV1 to PSV10 based on their proximal-to-distal order on the proximal palindrome arm in the reference sequence (Figure 1b, Table S4).

As a convenient medium-throughput system for typing SN-PSVs, we chose the Illumina GoldenGate Genotyping Assay (Illumina, San Diego, CA). This system does not allow assay design or reliable calling for some variants in particular sequence contexts, and was eventually used for the successful typing of seven analysed PSVs (PSV2, 3, 5, 7–10). Experiments were carried out at the Genomics Core Facility of the University of Leicester. Genotypes were called with the Illumina GenomeStudio software version 3.1.0.0 (Illumina). Results were validated by Sanger sequencing of 5% of samples (n = 19) for each PSV (133 sequencing reads in total). PSV1 and PSV4 were typed by PCR-RFLP analysis using the restriction enzyme *Tst*I (Fermentas) for the former and *Hpy166*II (NEB) for the latter. PSV6 was typed by allele-specific PCR.

### Phasing of palindrome arms

In order to phase the palindrome arms an arm-specific long-range PCR approach was developed, using one universal primer binding within the arm and an arm-specific primer binding outside the outer palindrome boundary, generating a distal-arm-specific fragment of 18,893 bp incorporating seven of the studied PSVs. This fragment was then used as a template in nested PCR followed by re-typing of the seven PSVs. Five of the PSVs were typed by PCR-RFLP analysis using the following restriction enzymes (all NEB except PSV1): PSV1 - *Tst*I, PSV2 - *Acu*I, PSV3 - *Hin*fI, PSV4 - *Hpy166*II and PSV7 – *Mnl*I. Sanger sequencing and allele-specific PCR were used for PSV5 and PSV6, respectively. All primer sequences are listed in Table S5.

Arm-specific haplotypes were compared to the known phase of the human Y-chromosome reference sequence. In total 83 samples were examined and all but three found to have identical phase to the reference sequence (Table S1).

### PCR approaches to verify the presence of both palindrome arms

In order to ascertain the presence of both palindrome arms in samples with long apparently pseudohomozygous stretches, a paralog ratio test (PRT) [47] was developed. PRT primers were designed to amplify fluorescently labelled 390-bp test fragments from both arms of P6 (Figure 1b), plus a single 387-bp reference region from chromosome X (Table S5). Products were resolved and quantified using an ABI3130xl Genetic Analyzer and GeneMapper software v4.0 (Applied Biosystems, Carlsbad, CA). A normal male is expected to have two palindrome arms and one X chromosome, resulting in a test-to-reference ratio of 2∶1. In total 50 samples were tested, each at least twice, including pseudoheterozygous controls known to contain both palindrome arms (Table S1). All samples showed the expected ∼2∶1 ratio except one (HGDP00445), which showed a ratio of ∼1∶1. Semi-quantitative analysis using the amelogenin sex test [48], which simultaneously amplifies different-sized X- and Y-specific fragments, showed an X∶Y ratio of 2∶1, consistent with this cell-line having a 47,XXY karyotype.

The presence of both palindrome arms was also checked by an additional PCR-based approach. Firstly, PCR primers were designed to specifically extend over and amplify both the inner and outer boundaries of the palindrome. Secondly, long-range PCR primers were used to amplify ∼10-kb fragments arm-specifically from the outer boundary of both arms followed by gel electrophoresis to check for changes in product length. The presence of all four palindrome boundaries and expected lengths of arm-specific PCR products was confirmed for all samples tested.

### Estimation of gene conversion rate

Mean gene conversion rate (assuming each converted SN-PSV represented an independent event) was estimated by dividing the number of conversion events (n), by the number of generations (g) encompassed in the phylogeny relating the 378 tested Y chromosomes. Estimation of g was based on a study [36] in which ∼80 kb of DNA were resequenced in 47 Y chromosomes covering most of the major branches of the Y phylogeny to ascertain unbiased nucleotide divergence, revealing a total of 95 base substitutions. Assuming a TMRCA of 118,000 years (supported by more recent large-scale resequencing [49]), a generation time of 25 years, and a human-chimpanzee divergence time of 6.5 million years, the 47 chromosomes encompassed 52,000 generations [36]. The 378 Y chromosomes we studied also included most haplogroups in the phylogeny, but also multiple examples in individual haplogroups. We estimated the number of additional generations contributed by these: for the lower bound we assumed that all chromosomes sharing major haplogroups contributed no additional base substitutions in excess of the haplogroup-specific branch lengths; for the upper bound we assumed that each additional chromosome in a given haplogroup contributed an additional number of base substitutions equivalent to its descending from the root of the clade independently. This led to a range of total base substitutions of 323–935, corresponding to ∼175,000–505,500 generations (Table S3).

### Sequencing and assembly of gorilla P6, and evolutionary comparisons

A partial consensus sequence of gorilla P6 was constructed from Illumina paired-end sequencing reads from: (i) whole genomes of two male gorillas giving an overall ∼6× Y-chromosome coverage [20]; (ii) a sequence-capture library (SureSelect, Agilent), using a repeat-masked probe-design based on the human reference sequence, of a male gorilla giving a mean coverage of targetable portions of P6 of 232× (Protocol S1). Reads from all samples were mapped against the spacer and proximal arm of P6 in the human reference (GRCh37) and a consensus sequence for a given nucleotide called where it was covered by at least 5 concordant reads and minimum base quality score 20.

Chimpanzee MSY sequence used in interspecific comparisons was taken from [19]. Sequence alignments were performed using the web-based ClustalW2 (http://www.ebi.ac.uk/Tools/msa/clustalw2) and Stretcher implemented in the EMBOSS package (http://emboss.sourceforge.net/).

## Supporting Information

Dataset S1 Sequence alignment of human, chimpanzee and gorilla P6.
(DOCX)

Figure S1 Y phylogeny, showing markers typed and data sources.
(TIF)

Table S1 Genotyping results.
(XLSX)

Table S2 Summary of identified gene conversion events.
(XLSX)

Table S3 Estimation of gene conversion rate.
(XLSX)

Table S4 Genotypes of 10 studied PSVs in different primate species and genomic coordinates in the human reference sequence.
(XLSX)

Table S5 Primer sequences.
(XLSX)

Table S6 Comparison of GC-content between human palindrome arms and spacers.
(XLSX)

Protocol S1 Sequencing and analysis of gorilla Y-chromosome palindrome, containing summary of NGS sequence data and coverage.
(DOCX)
