# Supplementary material for: Recombination Dynamics of a Human Y-Chromosomal Palindrome: Rapid GC-Biased Gene Conversion, Multi-kilobase Conversion Tracts, and Rare Inversions
Source: PLoS Genet. 2013 Jul 25;9(7):e1003666. doi: 10.1371/journal.pgen.1003666 (PMC3723533; doi:10.1371/journal.pgen.1003666)
Supplement: Dataset S1 — Sequence alignment of human, chimpanzee and gorilla P6. (DOCX) [file pgen.1003666.s001.docx]

**Dataset S1. Sequence alignment of human, chimpanzee and gorilla P6**

Human TCTTTCAGCATCATTTCAACTATTTCACCCCGTTACATGCATTGTAGTTAATGCCCTGAA 60

Chimpanzee TCTTTCAGCATCATTTCAACTATTTCACCCCGTTACAGGCATTGTAGTTAATGCCCTGAA 60

Gorilla TCTTTCAGCATCATTTCAATTATTTCACCCTGTTACATGCATTGTAGTTAATGCCCTGAA 60

******************* ********** ****** **********************

Human ATGTAGTTTCCTGCCAAACAAAATATCTTGAGAACACGTTGTGGCTGAGCCTGTATATTT 120

Chimpanzee ATGTAGTTTCCTGCCAAACAAAATATCTTGAGAACACATTGTGGCTGAGCCTGTATATTT 120

Gorilla ATGTAGTTTCCTGCCAAACAAAATATCTTGAGAACACGTTGTGGCTGAGCCTGTATATTT 120

************************************* **********************

Human ATCTACATTGTCTAAATAGCTATAGGTCATCAGAGAGCAGAAATATCCTATGCACTTATG 180

Chimpanzee ATCTGCATTGTCTAAATAGCTATAGGTCATCGGAGAGCAGAAATATGCTATGCACTTATG 180

Gorilla ATCTACATTGTCTAAATAGCTATAGGTCATCAGAGAGCAGAAATATGCTATGCACTTATG 180

**** ************************** ************** *************

Human TGACTCTTTCAATAGAGTTAACCACTTAGGACATCTCCAAATTCCTACAAGTGCAATGTT 240

Chimpanzee TGACCCTTTCAATAGAGTTAACCACTTAGGACATCTCCAAATTCCTACAAGTGCAATGCT 240

Gorilla CGACCCTTTCAATAGAGTTAACCACTTAGGACGTCTCCAAATTCCTGCAAGTGCAATGTT 240

*** *************************** ************* *********** *

Human GCAATGATGTTTTCTAGTTGGTGGCTCACTGTGAACATAACCTATTGAAATATCTATAAA 300

Chimpanzee GCAATGATGTTTTCTAGTTGGTGGCTCACTGTGAACATAACCTATTGAAATATCTATAAA 300

Gorilla GCAATGATGTTTTCTAGTTGGTGGCTCACTGTGAACATAACCTATTGAAATACCTATAAA 300

**************************************************** *******

Human GGTAGATATATAAATGTGCCCATTTTGGTCACTGGTCTTACTATGCTCACATCATGTGAA 360

Chimpanzee GATAGATATATAAATGTGCCCACTTTGGTCACTGGTCTTACTATGCTCACATCATGTGAA 360

Gorilla GATAGATATATAAATGTGCCCACTTTGGTCACTGGTCTTACTATGCTCACATCATGTGAA 360

* ******************** *************************************

Human AAAGATGAAGTAACTGACTAAATATGAAATATTGATAGGAACAGGAGGCAGGTAAATTCT 420

Chimpanzee AAAGATGAAGTAACTGACTAAATATGAAATATTGATAGGAACAGGAGGCAGGTAAATTCT 420

Gorilla AAAGATGAAGTAACTGACTAAATATGAAATATTGATAGGAACAGGAGGCAGGTAAATTCT 420

************************************************************

Human GGGTAGAAGAGGGTGGGTCCCTGGCAAGGGCCTC--ACCCTCAAGCCAAAAAGGCTAACA 478

Chimpanzee GGGTAGAAGAGGGTGGGTCCCTGGCAAGGGCCTCTCACCCTCAAGCTAAAAAGGCTAACA 480

Gorilla GGGTAGAAGAGGGTGGGTCCCTGGCAAGGGCCTC--ACCCTNAAGCCAAAAAGGCTAACA 478

********************************** ***** **** *************

Human CTGAAACTGACAGTGAGAATTGACATCCTTGCTTTCCCACTCAGATGTTGCCTTTTCTAA 538

Chimpanzee CCGAAACTGACAGTGAGAATTGACATCCTTGCTTTCCCACTCAGATGTTGCCTTTTCTAA 540

Gorilla CTGCAACTGACAGTAAGAATTGACATCCTTGCTTTCCCACTCAGATGTTGCCTTTTCTAA 538

* * ********** *********************************************

Human AACCACCCATTGTCTGCCCTATCCTGCTCCTGTGCCCAGAAAAAACCCCAGGCTCAGCAA 598

Chimpanzee AACCACCCATTGTCTGCCCTATCCTGCTCCTGTGCCCAG-AAAAACCCCAGGCTCAGCAA 599

Gorilla AACCACCTATTGTCTGCCCTATCCTGCTCTTGTGCCCAG-AAAAACCCCAGGCCCAGCNN 597

******* ********************* ********* ************* ****

Human GCAGAGAAAGGAGAAGAGGAGATGCAGCAGGACCTCAGGGACTACGGTTGGACATCAGAG 658

Chimpanzee GCAGAGAAAGGAGAAGAGGAGAAGCAGCAGGACCTCAGGGACTACGGTTGGACATCAGAG 659

Gorilla NNNNNNNNNNNNNNNNNNNNNNNGCAGCAGGACNNNNNNNNNNNNNNNNNNNNNTCAGAG 657

********** ******

Human AGATGCAGCTTGACTTCACAGGGACAGCTTGACAGTGTAGCTTTGGTGAGGAGTCCAACT 718

Chimpanzee AGATGCAGCTTGACTTCACAGGGACAGCTTGACAGTGTAGCTTTGGTGAGGAGTCCAGCT 719

Gorilla AGATNNNNNNTGACTTCACAGGGACAGCTTGACAGTGTAGCTTTGGTGAGGNNNNNNNNN 717

**** *****************************************

Human GTCCCCAGGGGAAGATTACTTTCCCTCTCTGTCACTGTTTCATCTCTCTTCCCGCTGAGA 778

Chimpanzee GTCCCCAGGGGAAGATTACTTTCCCTCTCTGTCACTGTTTCATCTCTCTTCCCGCTGAGA 779

Gorilla GTCCCCAGGGGAAGATTACTTTCCCTCTCTGTCACTGTTTCA-CTCTCTTCCCGCTNNNN 776

****************************************** *************

Human GCCACTTTCATCATCAATAAAATCCCCACATTTACCTCCTTCAATTCATTTGTGTGACGT 838

Chimpanzee GCCACTTTCATCATCAATAAAATCCCCACATTTACCTCCTTCAATTCATTTGTGTGACCT 839

Gorilla NNNNNNTTCATCNNNNNNNNNNNNNNNNNNNNNNNNNNNNNNNNNNNNNNNNNNNNNNNN 836

******

Human CTTTCCTCCTGGATGCAGGACGACTTGGGTGCCAAGAGTGTAAGTGCAAACGGCTGTTGC 898

Chimpanzee CTTTCCTCCTGGATGCAGGGCGACTTGGGTGCCAAGAGTGTAAGTGCAAACGGCTGTAGC 899

Gorilla NNNNNNNNCTGGATGCAGGACAACTTGGGTGCCAAGAGTGTAAGTGCAAACGGCTGTTGC 896

*********** * *********************************** **

Human ACTGACCCTCCACTGAGCTGTTAACTTTTAAGCCATCCATGGGTGGCAAAGCTAAAAAGG 958

Chimpanzee ACTGACCCTCCACTGAGCTGTTAACTTTTAAGCCATCCATGGGTGGCAAAGCTAAAAAGG 959

Gorilla ACTGACCCTCCACTGAGCTGTTAACTTTTNNNNNNNNCNNNNNNNNNNNNNNNNNNNNNN 956

***************************** *

Human TACTGTAACACTTCTCTGGGGATTCAGGAGTCATAGGTGAGGGGTGGAGCGCAGTGGGTC 1018

Chimpanzee TACTGTAACACTTCTTTGGGGATTCAGGAGTCATAGGTGAGGGGTGGAGCGCAGTGGGTC 1019

Gorilla NACTGTAACACTTNNTTGGGGATTCAGGAGTCATAGNNNNNNGGGGNNNNNNNNNNNNNN 1016

************ ******************** ** *

Human AAGTGAGTGGGGTTCGCTCCTGCTGACACCTGTGCACTGCAATTCCCACCCCTGAAAGGG 1078

Chimpanzee AAGTGAGTGGGGTTCGCTCCTGCTGACACCTGTGCACTGCAATTCCCACCCCTGAAAGGG 1079

Gorilla NNNNNNNNNNNNNNNNNNNNNNNNNNNNNNNNNNNNNNNNNNNNNNNNNNNNNNNNNNNN 1076

Human TCAGGGAACTATCCTGTTTCAATATCATATCTGATAATATAGAATTTGCTGCTATTTGCA 1138

Chimpanzee TCAGGGAACTATCCTGTTTCAGTATCACATCTGATAATATAGAATTTGCTGTTATTTGCA 1139

Gorilla NNNNNNNNNNNNNNNNNNNNNNNNNNNNNNNNNNNNNNNNNNNNNNNNNNNNNNNNNNNN 1136

Human TTCAATAAATCTGGAGTTAAACAAAACAAAACCATTTGTAATTCCAAATAATAAACACAC 1198

Chimpanzee TTCAATAAATCTGGAGTTAAACAAAACAAAACCATTTGTAATTCCAAATAATAAACACAC 1199

Gorilla NNNNNNNNNNNNNNNNNNNNNNAAAACAAAACCATTTGTAATTCCAAATAATAAACANNN 1196

***********************************

Human ACCTCCTCAATGATAGTTTCAGAAGCAATGATACCAGCTCCTCTTCATACCTCTAGAAGA 1258

Chimpanzee ACCTCCTCAATGATAGTTTCAGAAGGAATGATACCAGCTCCTCTTTGTACCTCTAGAAGA 1259

Gorilla NNNNNNNNNNNNNNNNNNNNNNNNNNNNNNNNNNNNNNNNNNNNNNNNNNNNNNNNNNNN 1256

Human AGTCAGCTGTGATGCCTTCTAGCCCTGGGCTTTTTTT-GGTTAGTAGGCTATTAGTTACT 1317

Chimpanzee ATTCAGCTGTGATGCCTTCTAGCCCTGGGCTTTTTTTTGGTTAGTAGGCTATTAGTTACT 1319

Gorilla NNNNNNNNNNNNNNNNNNNNNNNNNNNNNNNNNNNNNNNNNNNNNNNNNNNNNNNNNNNN 1316

Human GCCTCAATTTCAGAAGTTGTTATTGTTACATTCAGGGATTCGAATTCTTCCTCATTTAGT 1377

Chimpanzee GCCTCAATTTCAGAAGTTGTTATTGTTATATTCAGGGATTCAAATTCTTCCTCGTTTAGT 1379

Gorilla NNNNNNNNNNNN-AAGTTGTTATTGTTATATTCAGGGATTCGAATTCTTCCTCGTTTAGT 1375

*************** ************ *********** ******

Human ATTTGGAGGGTGTAGGTGTCCAGGAATTTATCCATTTCTTCTGGATTTTCTAGTTCAGTT 1437

Chimpanzee ATTTGGAGGGTGTAGGTGTCCAGGAATTTATCCATTTCTTCTGGATTTTCTAGTTCAGTT 1439

Gorilla ATTTGGAGGGTGTAGGTGTCCAGGAATTTATCCATTTCTTCTGGATTTTGTAGTTCAGTT 1435

************************************************* **********

Human GCATATAGGTGTTTATAGTATTCTCTGATGGTAGTTTGTATTTCTGTGGGATAAAATACT 1497

Chimpanzee GCATGTAGGTGTTTATAGTGTTCTCTGATGGTAGTTTGTATTTCTGTGGGATAAAATACT 1499

Gorilla GCATATAGGTGTTTATAGTATTCTCTGATGGTAGTTTGTATTTCTGTGGGATAAAATACT 1495

**** ************** ****************************************

Human CCCTTTATCATTTTTTGGGGGGAGTTAGACATAAATATTATGGAAGTTAAGAAACGATAA 1557

Chimpanzee CCCTTTATCATTTTTT-GGGGGAGTTAGACATAAATATTATGGAAGTTAAGAAACGATAA 1558

Gorilla CCCTTTATCATTTTTT-GGGGGAGTTAGACATAAATATTATGGAAGTTAAGAAACGATAA 1554

**************** *******************************************

Human AACCCTAAGCTTCTCTGGCTTAACAGGATCAACTCTGTTGGAGATGAAATCACTGTGGTG 1617

Chimpanzee AACCCTAAGCTTCTCTGGCTTAACAGGATCAACTCTGTTGGAGATGAAATCACTGTGGTG 1618

Gorilla AACCCTAAGCTTCTCTGGCTTAACAGGATCAACTCTGTTGGAGATGAAATCACTGTGGTG 1614

************************************************************

Human GGGAATAGATTGGAAGCCCCTTGCCACCATGAGTGCCTTCTGTTGGAGAGTCATTTCTTC 1677

Chimpanzee GGGAATAGATTGGAAGCCCCTTACCACCATGAGTGCCTTCTGTTGGAGAGTCATTTCTTC 1678

Gorilla GGGAATAGATTGGAAGCCCCTTACCACCATGAGTGCCTTCTGTTGGAGAGTCATTTCTTC 1674

********************** *************************************

Human TTCCAGCCTGTATGCATTCTGTTCTCTAAACTCATATTTTTATTGCTATAAACTACAGCA 1737

Chimpanzee TTCCAGCCTGTATGCATTCTGTTCTCTAAACTCATATTTTTATTGCTATAAACTACAGCA 1738

Gorilla TTCCAGCCTGTATGCATTCTGTTCTCTAAACTCATATTTTTATTGCTATAAACTACAGCA 1734

************************************************************

Human GCATTATTTCTGGAATTATTTTCTTTCTTCGACACCTATGTTTGTGATTAATTTATAACA 1797

Chimpanzee GCATTATTTCTGGAATTATTTTCTTTCTTCAACACCTATGTTTGTGATTAATTTATAACA 1798

Gorilla GCATTATTTCTGGAATTATTTTCTTTCTTCAACACCTATGTTTGTGATTAATTTATAACA 1794

****************************** *****************************

Human TATGAGATATATATGAATCTATATCTATATAATTTTTGCAGATATAATTGACATAAGATG 1857

Chimpanzee TATGAGATATATAGGAATCTATATCTATATAATTTTTGCAGATGTAATTGACATAAGATG 1858

Gorilla TATGGGATGTATATGAATCTATATCTATATAATTTTTGCAGATATAATTGACATAAGATG 1854

**** *** **** ***************************** ****************

Human AAGCAATACTGGATTATGGCAGAACCTAAATCTGACAGCTGGTATCTTTGTAACAGAAGG 1917

Chimpanzee AAGCAATACTGGATTATGGCAGAACCTAAATCTGACAGCTGGTATCTTTGTAACAGAAGG 1918

Gorilla AAGCAATACTGGATTATGGCAGAACCTAAATCTGACAGCTGGTATCTTTGTAACAGAAGG 1914

************************************************************

Human AGAGGAAGAATTGGATAAAGAGACATATGGAAGATCTGAAGATGGAAGCTATGTTCGAAT 1977

Chimpanzee AGAGGAAGAATTGGATAAAGAGACATATGGAAGATCTGAAGATGGAAGCTATGTTCGAAT 1978

Gorilla AGAGGAAGAATTGGATNNNNNNNNNNNNNNNNNNNNNNNNNNNNNNNNNNNNNNNNNNNN 1974

****************

Human GATGTGGTAACAAGCCAAGGGATTCCTGAATCAACCAGGAGCTGGGAAAGGCAGGAAAGG 2037

Chimpanzee GATGTGGTAACAAGCCAAGGGATTCCTGAATCAACCAGGAGCTGGGAAAGGCAGGAAAGG 2038

Gorilla NNNNNNNNNNNNNNNNNNNNNNNNNNNNNNNNNNNNNNNNNNNGGGAAAGGCAGGAAGGG 2034

************** **

Human TCCTCCCTTGGAGCCTGTGGAGGGAGAGACGCACTGCCTCACTTTGATTTTTAAACTGCC 2097

Chimpanzee TCCTCCCTTGGAGCCTGTGGAGGGAGAGACGCACTGCCTCACTTTGATTTTTAAACTCCC 2098

Gorilla TCCTCCCTTGGAGCCTATGGAGGGAGAGACACACTGCCTCNNNNNNNNNNNNNNNNNNNN 2094

**************** ************* *********

Human AGCAGCAGGAGAGATTACATTTCCTAAGTAGTGGGGATTACAAGCATGAGCCACCAGGCC 2157

Chimpanzee AGCATCAGGAGAGACTACATTTCCTAAGTAGTGGGGATTACAAGCATGAGCCACCAGGCC 2158

Gorilla NNNNNNNNNNNNNNNNNNNNNNNNNNNNNNNNNNNNNTNNNNNNNNNNNNNNNNNNNNNC 2154

* *

Human TGGGGAATTTTTGTAATTTTAATAGAGAAAGGGTTTCACCATGTTGGCCACACTACTCAT 2217

Chimpanzee TGGGGAATTTTTGTAATTTTAATAGAGAAAGGGTTTCACCATGTTGGCCACACTACTCAT 2218

Gorilla TGGGAAATNNNNNNNATTTTAATAGAGAAAGGGTTTCACCATGTTGGCCACACTACTCAT 2214

**** *** *********************************************

Human GAAGTCCTGACCTTAGGTGGTCTGCCCACCTCGGCCTCCCAAAGTGCTGGGATTACAGGC 2277

Chimpanzee CAAGTCCTGACCTTAGGTGGTCTGCCCACCTCGGCCTCCCAAAGTGCTAGAATTACAGGC 2278

Gorilla GAAGTCCTGACCTTAGNNNNNNNGCCCACCTTGGCCTCCCAAAGTNNNNNNNNNNNNNNN 2274

*************** ******** *************

Human ATAAACCAATGCACCTGGCCAGAAATTCTTATTGTTTTAAGCCACCCAGTTTGTAGTTAT 2337

Chimpanzee ATAAACCAATGCGCCTGGCCAGAAATTCTTATTGTTTTAAGCCACCCAGTTTGTAGTTAT 2338

Gorilla NNNNNNNNNNNNNCCTGGCCAGAAATTCTTATTGTTTTAAGCCACCCAGTTTGTAGTTAT 2334

***********************************************

Human TTCAATGGCAGGCATGGGAAACTTCTAAAACACATATACATGGATGTATAGGCATATTCA 2397

Chimpanzee TTCAATGGCAGGCATGGGAAACTTCTAAAACACATATACATGGATGTATAGGCATATTCA 2398

Gorilla TTCAAAGGCAGGCATGGGAAACTTCTAAAACACATATACATAGATGTATAGGCATATTCA 2394

***** *********************************** ******************

Human CATACACACTTGTATGTACAGTCATATGCTACATAATGACATTTTAGTCAATGACAGACT 2457

Chimpanzee CATACACACTTGTATGTACAGTCATATGCTACATAATGACATTTTAGTCAATGACAGACT 2458

Gorilla CATACACACTTGTATGTGCAGTCATATNCNNNNNNNNNNNNNNNNNNNNAATGACAGNNN 2454

***************** ********* * ********

Human ACATATATGATAAGAATTCCATGAGATTATAATGGACCTCAGATATTCCTATTATAATGT 2517

Chimpanzee ACATATATGATAAGAATTCCATGAGATTATAATGGGCCTCAGATATTCCTATTATAATGC 2518

Gorilla NNNNNNNNNNNNNNNNNNNNNNNNNNNNNNNNNNNNNNNNNNNNNNNNNNNNNNNNATGC 2514

***

Human GCTGTCCTAGGCTTCCTAGGGTTGTAGCACAAAAGCATTACTCATGTGATTGTGTCCATG 2577

Chimpanzee GCTGCCCTAGGCTTCCTAAGGTTGTAGCACAAAAGCATTACTCATGTGATTGTGTCCATG 2578

Gorilla GCTGTCCTAGGCTTCCTAGGGTTGTAGTACAAAAGCATTACTCATGTGATTGTGTCCACG 2574

**** ************* ******** ****************************** *

Human CTAGTGTAGAGGCATGTTGCCAGTTAAATAAAAGTGTGCAATAATGTCGCAGGTCTTCAT 2637

Chimpanzee CTAGTGTAAAGGCATGTTGCCAGTTAAATAAAAGTGTGCAATAATGTTGCAGGTCTTCAT 2638

Gorilla CTAGTGTAAAGGCATGTTGCCAGTTAAATAAAAGTGTGCAATAATGTCACAGGTCTTCAT 2634

******** ************************************** ***********

Human ATCCCCTCATCACCCACTCACAGACTCACCCAGAGCAACCTCCAGTCTTGCAAGCTCCAT 2697

Chimpanzee ATCCCCTCATCACCCACTCACAGACTCACCCAGAGCAACCTCCAGTCTTGCAAACTCCAT 2698

Gorilla ATCCCCTCATCACCCGCTCAGAAACTCACCCAGAGCAACCTCCAGTCTTGCAAGCTCCAT 2694

*************** **** * ****************************** ******

Human TCACGGTAACTGCCCTATACAGGGCTACCATTTTATATTTTTGATGCTGCATTCTTACTG 2757

Chimpanzee TCACGGTAACTGCCCTATACAGAGCTACCATTTCATATTTTTGATGCTGCATTCTTACTG 2758

Gorilla TCATGGTAACTGCCCTATACAGGGCTACCATTTTGTATTTTTGATGCTGCATTCTTACTG 2754

*** ****************** ********** *************************

Human TACGTTTTCAGTGTTTAGATGAACAAGTACTTGCCACTGTGTTGCAGTTGTCTGTGGTGT 2817

Chimpanzee TACGTTTTCAGTGTTTAGATGAACAAGTACTTGCCACTGTGTTGCAGTTGTCTGTGGTGT 2818

Gorilla TACAATTTCAGTGTTTAGATGAACAAGTACTTGCCACTGTGTTGCAGTTGTCTGTGNNNT 2814

*** *************************************************** *

Human TTAGCATGGTATTGTGCTGTACAGGTTTGTGGCCTAGGAGCAATAGGCTCTACCATATAG 2877

Chimpanzee TTAGCATGGTATTGTG-TGTACAGGTTTGTGGCCTAGGAGCAATAGGCTCTACCATATAG 2877

Gorilla TNNNNNNNNNNNNNNNNNNNNNNNNNNNNNNNNNNNNNNNNNNNNNNNNNNACCATATAG 2874

* *********

Human CCTAAGTGTGTAGTAGGCTGTACCATCTAAGACTGCATAAGTACACTCTAGGATGTTCGC 2937

Chimpanzee CCTAAGTGTGTAGTAGGCTGTACCATCTAAGACTGCATAAGTACACTCTAGGATGTTCGC 2937

Gorilla CCTTAGTGTGTAGTAGGCTGTACCATCTAAGACTGCATAAGTACACTCTAGGATGTTCGC 2934

*** ********************************************************

Human ACGAGAACTAAATCAAACAAGGACACATTTCTCAGAAGTATTGTCATTAAGTAACACATG 2997

Chimpanzee ACGAGAACTAAATCAAACAAGGACACATTTCTCAGAAGTATTGTCATTAAGTAACACATG 2997

Gorilla ACGAGAACTACATCAACCAAGGACACATTTCTCAGAAGTATTGTCATTAAGTAACACATG 2994

********** ***** *******************************************

Human ACTCTTTATACATACACATAGGCATAGGCATATAGAAAAAACAAATGTAACTGAAATGGG 3057

Chimpanzee ACTGTTTATACATACACATAGGCATAGGCATATAGAAAAAACAAATGTAACTGAAATGGG 3057

Gorilla ACTGTTTATACATACACATTGGCATAGGCATATAGAAAAAACAAATGTAACTGAAATGGG 3054

*** *************** ****************************************

Human ATCTGGCAATGTGCAGAAACCAAGGCAGCTTTTCTCTGGGTCTCTCTCACAGGAGTCAGC 3117

Chimpanzee ATCTGGCAATGTGCAGAAACCAAGGCAGCTTTTCTCTGGGTCTCTCTCACAGGAGCCAGC 3117

Gorilla ATCTGGCAATGTGCAGAAACCAAGGCAGCTTTTCTCTGGGTCTCTCTCACAGGAGTCAGC 3114

******************************************************* ****

Human ATCCCTTTGCACATGGGATCCAACATGCTTCTCTGCGTATATGATTCGTCTTCTATCATG 3177

Chimpanzee ATCCCTTTGCACATGGGATCCAACATGCTTCTCTGCGTATATGATTCGTCTTCTATCATG 3177

Gorilla ATCCCTTTGCACATGGGATCCAACATGCTTCTCTGCGTATATGATTCGTCTTCTATCATG 3174

************************************************************

Human GGCCATCATTAGTCCTAGCTTAGAGTACATTAATAAAAACATGGCCTTTAATAGGTGGGA 3237

Chimpanzee GGCCATCATTAGTCCTATCTTAGAGTACATTAATAAAAACATGGCCTTTAATAGGTGGGA 3237

Gorilla GGCCATCATTAGTCCTAGCTTAGAGTACATTAATAAAAACATGGCCTTTAATAGGTGGGA 3234

***************** ******************************************

Human GTCACATCAAGTTTTACATTATCCAAGTGGCTGTGTGTGACACCCTATAAACAGTAGCAA 3297

Chimpanzee GTCACATCAAGTTTTACATTATCCAAGTGGCTGTGTGTGACACCCTATAAACAGTAGCAA 3297

Gorilla GTCACATCAAGTTTTACATTATCCAAGTGGCTGTGTGTGACACCCTATAAACAGTAGCAA 3294

************************************************************

Human CAACCCCTGAGATCTCTGTATCTCTGTATCTTGCTTGTCTTTCTAGAGAGTTATAGCAGA 3357

Chimpanzee CAACCCCTGAGATCTCTGTATCTCTGTATCTTGCTTGTCTTTCTAGAGAGTTATAGCAGA 3357

Gorilla TAACCCCTGAGATCTCTGTATCTCTGTATCTTGCTTGTCTTTCTAGAGAGTTATAGCAGA 3354

***********************************************************

Human AACACAAGGTTTGTAACCTATTTGCCAGAGTGTAACCATAAGCTCCCCTTTTTCCCATAA 3417

Chimpanzee AACACAAGGTTTGTAACCTATTTGCCAGAGTGTAACCATAAGCTCCCCTTTTTCCCATAA 3417

Gorilla AACACAAGGTTTGTAACCTATTTGCCAGAGTGTAACCATAAGCTCCCCTTTTTCCCATAA 3414

************************************************************

Human CTAATGAGGTAGTGGTAAATTCAGAATAGGTCTATATATGCAGGTGTGTGTGTGTGTGTA 3477

Chimpanzee CTAATGAGGTAGTGGTAAATTCAGAATAGGTCTATATATGCAGGTGTGTGTGTGTGTGTA 3477

Gorilla CTAATGAGGTAGTGGTAAATTCAGAATAGGTCTATATATGCAGGTGTGTGTGTGTGTGTA 3474

************************************************************

Human TATGTATATGTGTGTGTGTATATGTATATATGTGTGTGTGTATATATATATATATATGAT 3537

Chimpanzee TATGTATATGTGTGTATATATGTGTATATGTGTGTGTGT--------ATATATATATGAT 3529

Gorilla TATGTATATGTGNNNNNNNATATGTATATATGTNNNNNNNNNNNNNNNNNNNNNNNNNNN 3534

************ ** ******* ***

Human ATAGTGTTCTTTATACTCCTTGTGAGAGAATGTAATATATATCCACATATAGATAATATT 3597

Chimpanzee ATAGTGTTCTTTATACTCCTTGTGAGAGAATGTAATATATATCCACATATAGATAATATT 3589

Gorilla NNNNNNNNNNNNNNNNNNNNNNNNNNNNNNNNNNNNNNNNNNNNNNNNNNNNNNNNNNNN 3594

Human GTGTAT---ATTATATAATACGTATGAACTGTATTGTATGTTTATATTTAT-AAATATAA 3653

Chimpanzee GTGTATGATATTATATAATACATATGAACTGTATTGTATGTTTATATTTATTAAATATAA 3649

Gorilla NNNNNN---NNNNNNNNNNNNNNNNNNNNNNNNNNNNNNNNNNNNNNNNNN-NNNNNNNN 3650

Human TGCTTTTATATTATAGAAATATACATATATTTTTATGTTTTAAAAGTAAATA-ATAAATA 3712

Chimpanzee TGCTTTTATATTATAGAAATATACATATATTTTTATGTTTTAAAAGTAAATATATAAATA 3709

Gorilla NNNNNNNNNNNNNNNNNNNNNNNNNNNNNNNNNNNNNNNNNNNNNNNNNNNN-NNNNNNN 3709

Human GAATTGTACAAATATGATGTGTAATATAAATATTACATATTTTATTTATATGTAAGTTGT 3772

Chimpanzee TAATTGTACAAATATGATGTGTAATATAAGTATTACATATTTTATTTATATGTAAGTTGT 3769

Gorilla NNNNNNNNNNNNNNNNNNNNNNNNNNNNNNNNNNNNNNNNNNNNNNNNNNNNNNNNNNNN 3769

Human ATTTTATTTTTATGTAAGTTATATATTACATATATGTAAATATTTATGTACTGTATGTTT 3832

Chimpanzee ATTTTATTTTCATGTAAGTTATATATTACATATATGTAAATATTTGTGTACTGTATGTTT 3829

Gorilla NNNNNNNNNNNNNNNNNNNNNNNNNNNNNNNNNNNNNNNNNNNNNNNNNNNNNNNNNNNN 3829

Human GTATATTTTATATTTACTATATGTAAAATTTATATATATAAATATACATATATTACATGT 3892

Chimpanzee GCATATTTTATATTTACTGTATGTAAAATTTATATATATAAATATACGTATATTACATGT 3889

Gorilla NNNNNNNNNNNNNNNNNNNNNNNNNNNNNNNNNNNNNNNNNNNNNNNNNNNNNNNNNNNN 3889

Human TTAAGGTATATTTCTATTATAGTACAAGTTTATATTAGAAATAAATATACACATATATTT 3952

Chimpanzee TTAAGGTATATTTCTATTATAGTACAAGTTTATATTAGAAATAAATATATACATATATTT 3949

Gorilla NNNNNNNNNNNNNNNNNNNNNNNNNNNNNNNNNNNNNNNNNNNNNNNNNNNNNNNNNNNN 3949

Human CTATATTAGAAATTTATATATTATATGTATTACATGTATATGTTTTTATATATAATATGC 4012

Chimpanzee CTATATTAGAAATTTATATATTATATGTATTACATGTATATGTTTTTATATATAATATGC 4009

Gorilla NNNNNNNNNNNNNNNNNNNNNNNNNNNNNNNNNNNNNNNNNNNNNNNNNNNNNNNNNNNN 4009

Human CTATTATATATATTCTAGTATCTATTATATTTACATATTACATATTATTTAGTCTTTGTG 4072

Chimpanzee CCATTATACATATTCTAGTATCTATTATATTTACATATTACATATTATTTAGTCTTTGTG 4069

Gorilla NNNNNNNNNNNATTCTAGTATCTATTATATTTACATATTACATATTATTTAGTCTTTGTG 4069

*************************************************

Human TGAACATCCTAGAGTGTACTTACACAATCTTAGATGGTACAGCCATGTATATATACTCAT 4132

Chimpanzee TGAACATCCTAGAGTGTACTTACACAATCTTAGATGGTACAGCCATGTATACATACTCAT 4129

Gorilla TGAACATCCTAGAGTGTACTTACACAATCTTAGATGGTACAGCCATGTATATATACTCAT 4129

*************************************************** ********

Human ATATATATGAGGTTCTCCAAAGTGTTTCCGGTAAAAACACTAGGTCACTGGCCGTTGTGC 4192

Chimpanzee ATATATATGAGGTTCTCCAAAGTGTTTCCGGTAAAAACACTAGGTCACTGGCCATTGTGC 4189

Gorilla ATATATATGAGGTTCTCCAAAGTGTTTCCGGTAAAAACACTAGGTCACTAGCCATTGTGC 4189

************************************************* *** ******

Human TGTGCTAGGATTCATGACTAGAAGATACACTAGTATCTTCCCTCAAGAGTTTAACATTAA 4252

Chimpanzee TGTGCTAGGATTCATGACTAGAAGGTACACTAGTATCTTCCCTCAAGAGTTTAACATTAA 4249

Gorilla TGTGCTAGGATTCATGACTAGAAGATACACTAGTATCTTCCCTCAAGAGTTTAACATTAA 4249

************************ ***********************************

Human AGATGCATGGACACACAATAATAAATATAGTGGTAATATTGTAACTTCTATGTTTCTTGC 4312

Chimpanzee AGATGCATGGACACACAATAATAAATATAGTGGTAATATTGTAACTTCTATGTTTCTTGC 4309

Gorilla AGATGCATGGACACACAATAATAAATATAGTGGTAATATTGTAACTTCTATGTTTCTTGC 4309

************************************************************

Human CTAGCCACACCAAAGAATTAGTGTGACAGCTGACTGCAGCCAGTGATAGAGACACAGACC 4372

Chimpanzee CTAGCCACACCAAATAATTAGTGTGGCAGCTGACTGCAGCCGGTGATAGAGACACAGACC 4369

Gorilla CTAGCCACACCAAAGAATTAGTGTGGCAGCTGACCGCAGCCAGTGATAGAGACACAGACC 4369

************** ********** ******** ****** ******************

Human GATAGAAAAAGCTGTAGGCTTTATTGAGCAGAGTGAAAGTACAAAGATTCCACAGCGTGG 4432

Chimpanzee GATAGAAAAAGCTGTAGGCTTTATTGAGCAGAGTGAAAGTACAAAGATTCCACAGCGTGG 4429

Gorilla GAAAGAAAAAGCTGTAGGCTNNNNNNNNNNNNNNGAANNNNNNNNNNNNNNNNNNNNNNN 4429

** ***************** ***

Human AAGGGATCCTGAACAGGTAGCCAGAGTTAGATTATAGAAATGCCTTCTAATTTCTTTAAG 4492

Chimpanzee AAGGGATCCTGAACAGGTAGCCAGAGTTAGATTATAGAAATGCCTTCTAATTTCTTTAAG 4489

Gorilla NNNNNNNNNNNNNNNNNNNNNNNNNNNNNNNNNNNNNNNNNNNNNNNNNNNNNNNNNNNN 4489

Human GTGGGAAATACCTGTGGCGGGAAAATGTTACCAAAGCAAGAAACAAAGACAGTTAACAAT 4552

Chimpanzee GTGGGAAATACCTGTGGTGGGAAAATGTTACCAAAGCAAGAAACAAAGACAGTTAACAAT 4549

Gorilla NNNNNNNNNNNNNNNNNNNNNNAAATGTTACCAAAGCAAGAAACAAAGACAGTTAACAAT 4549

**************************************

Human TTGTGGCATGTTTTAGATCTTGAGGAAAACTGGAATTGTAACTTAGGATTTGTCTACTTT 4612

Chimpanzee TTGCGGCATGTTTTAGATCTTGAGGAAAACTGGAATTGTAACTTAGGATTTATCTACTTT 4609

Gorilla TTGTGGCATGNNNNNNNNNNNNNNNNNNNNNNNNNTTGTAACTTAGGATTTATCTACTTT 4609

*** ****** **************** ********

Human AGGACCTTGCTGCAGTATGGCAAAGGAGACAGAATATCACAGAACTTTACAAAGCATGTT 4672

Chimpanzee AGGACCTTGCCGCAGTATGGCAAAGGAGACAGAATATCACAGAACTTTACAAAGCATGTT 4669

Gorilla AGGACCTTGCTGCAGTATGGCNAAGGAGACAGANNNNNNNNGAACTTTNCAAAGTATGNN 4669

********** ********** *********** ******* ***** ***

Human TACAAGGAATTGGAATTGGGAGCATAGATAAGGTCTGCTGATCACAGAAAAATGGACAGC 4732

Chimpanzee TACAAGGAATTGGAATTGGGAGCATAGATAAGGTCTGCTGATCCCAGAAAAATGGACAGC 4729

Gorilla NNNNNNNNATTGGAATTGGGAGCATNNNNNNNNNNNNNNNNNNNNNNNNNNNNNNNNNNN 4729

*****************

Human TAACATTCCTTTTACTTTAGTTTTGTGGGAGGGGAAGGCGGAGAGGGAGAGAAGACACAG 4792

Chimpanzee TAACATTCCTTTTACTTTAGTTTTGTGGGAGGGGAAGGCGGAGAGGGAGAGAAGACACAG 4789

Gorilla NNNNNNNNNNNNNNNNNNNNNNNNNNNNNNNNNNNNNNNNNNNNNGGAGAGAAGACACAG 4789

***************

Human GAAAACTTACAGCAAAATTGTTCCTGTTTATAGCTTTCTTGGGGGAGAAAACACATGAAC 4852

Chimpanzee GAAAACTTACAGCAAAATTGTTCCTGTTCATAGCTTTCTTGGGGGAGAAAACACATGCCC 4849

Gorilla GAAAACTTACAGCAAAATTGTTCCTGTTTGTAGCTTTCTTGGGGGAGAAAACANNNNNNN 4849

**************************** ***********************

Human AAATCCTGGTGTTAGGAATAGTTTAAGCCTATATCTTCGGTATCATTCATCCAGGATTGA 4912

Chimpanzee AAATCCTGGTGTTAGGAATAGTTTAAGCCTATATCTTCGGTATCATTCATCCAGGATTGA 4909

Gorilla NNNNNNNNNNNNNNNNNNNNNNNNNNNNNNNNNNNNNNNNNNNNNNNNNNNNNNNNNNNA 4909

*

Human GGTAATTCCTGATGCAGGAAATCAGTGAGTTTCCCAGCTTTCTGGGCCCCTACTGAATCC 4972

Chimpanzee GGTAATTCCTGATGCAGGAAATCAGTGAGTTTCACAGCTTTCTGGGTCCCTACTGAATCC 4969

Gorilla NNNNNNNNNNNNNNNNNNNNNNNNNNNNNNNNNNTAGCTTTCTGGGCCCCTACNNNNNNN 4969

*********** ******

Human AGGAAGCCCAGCCGGCCCCTCCTTTCAGTCCCCCCTCTAAACAGGACCCTCCAACTGCTC 5032

Chimpanzee AGGAAGCCCAGCTGGCCCCTCCTTTCAGTCCCCCCTGTAAACAGGACCCTCCAACTGCTC 5029

Gorilla NNNAAGCCCANNCAGCCCCTCCTTTCAGTTCCCCCTCTAAACAGGACCCTCCAACTGCTC 5029

******* *************** ****** ***********************

Human TTGAGAACTGGGTGGCGGTCGTTCTGGCTACTTCCTGCTTGTTAGGGGCAAAGAAGGGAC 5092

Chimpanzee TTGAGAACTGGGTGGCGGTCGTTCTGGCTACTTCCTGCTTGTTAGGGGCAAAGAAGGGAC 5089

Gorilla TTGAGAACTGGGTGGTGGTCATTCTGGCTACTTCCTGCTTGTTAGGGGCAAAGAAGGNNN 5089

*************** **** ************************************

Human CCTGTAGTTGTGGTGTTCTTCAGAGGGGAACTTTCTAGGCCAGTCAAGGTTCCAATGTGT 5152

Chimpanzee CCTGTAGTTGTGGTGTTCTTCGGAGGGGAACTTTCTAGGCCAGTCAAGGATCCAATGTGT 5149

Gorilla NNNNNNNNNNNNNNNNTCTTCAGAGGGGAACTTTCTAGGCCAGTCAAGGGTCCAATGTGT 5149

***** *************************** **********

Human CAATCCAGGTGTCCTCAGTAGAAGCTGTGATTTGAGCTGATTTGAGGTTCCATTTGTACG 5212

Chimpanzee CAATCCGGGTGTCCTCAGTAGAAGCTGTGATTTGAGCTCATTTGAGGTTCCATTTGTAAG 5209

Gorilla CAATCCAGGTGTCCTCGGTAGAAGCTGTGATTTGAGCTCATTTGAGGTTCCNNNNNNNNN 5209

****** ********* ********************* ************

Human ACCATTTGTAGCATGATGGCCTCGATCCTGGAGGAAACAAATTTGACACAGAGGTTAAAA 5272

Chimpanzee GCCATTTGTAGCATGATGGCCTCGATCCTGGAGGAAACAAATTTGACACAGAGGTTAAAA 5269

Gorilla NNNNNNNNNNNNNNNNNNNNNNNNNNNNNNNNNNNNNNNNNNNNNNNNNNNNNNNNNNNN 5269

Human ATGCAAGGCCTAAAGGTGAGTAAAAATAGGATGGGTGTCACAGGACCTAGAAAGGGGAGT 5332

Chimpanzee ATGCAAGGCCTAAAGGTGAGTAAAAATAGTATGGGTGTCACAGGACCTAGAAAGGGGAGT 5329

Gorilla NNNNNNNNNNNNNNNNNNNNNNNNNNNNNNNNNNNNNNNNNNNNNNNNNNNNNNNNNNNN 5329

Human AGCCAAGGTATCCATTGGTTAAACATATTTCAGGGTTCAGAGTGATCAAGCTCCTTTTTC 5392

Chimpanzee AGCCAAGGTATCCATAGGTTAAACATATTTCAGGGTTCAGAGTGATCAAACTCCTTTTTC 5389

Gorilla NNNNNNNNNNNNNNNNNNNNNNNNNNNNNNNNNNNNNNNNNNNNNNNNNNNNNNNNNNNN 5389

Human CTACTTTTTTCCGTTCTCTTATTTATTTAAACTTTTCAGTAATGATTGCTGACTGGTTAA 5452

Chimpanzee CTACTTTTTTCCGTTCTCTTATTTATTTAAACTTTTCAGTAATGATTGCTGACTGGTTAA 5449

Gorilla NNNNNNNNNNNNNNNNNNNNNNNNNNNNNNNNNNNNNNNNNNNNNNNNNNNNNNNNNNNN 5449

Human TGAAACAGCAACCTTCTTCTCCCAAGAAGATGAAGGTTCCTCTTCTTTTAGCTGTTAATA 5512

Chimpanzee TGAAATAGCAACATTCTTCTCCCAAGAAGATGAAGGTTCCTCTTCTTTTAGCTGTTAATA 5509

Gorilla NNNNNNNNNNNNNNNNNNNNNNNNNNNNNNNNNNNNNNNNNNNNNNNNNNNNNNNNNNNN 5509

Human GGTCCAGGGATCTTTGGTTTTGGAGGACCACTGCAGCTAGAGAAGTAAGCTGGCTTTGTA 5572

Chimpanzee GGTCCAGGGATCTTTGGTTTTGGAGGACCACTGCAGCTAGAGAAGTAAGCTGGCTTTGTA 5569

Gorilla NNNNNNNNNNNNNNNNNNNNNNNNNNNNNNNNNNNNNNNNNNNNNNNNNNNNNNNNNNNN 5569

Human GGGTCACTAGGGTGTTGGAAATTTGTTCCATGTCATCATTTAATTTTCGTGATAATTTAT 5632

Chimpanzee GGGTCACTAGGGAGTTGGAAATTTGTTCCATGTCATCATTTAATTTTCGTGATAATTTAT 5629

Gorilla NNNNNNNNNNNNNNNNNNNNNNNNNNNNNNNNNNNNNNNNNNNNNNNNNNNNNNNNNNNN 5629

Human AATAGAATTGAGTGGAGGAGGTTATGCCTCCAATTCCAATAGGAGCCCACCTATTATTCT 5692

Chimpanzee AATAGAATTGAGTGGAGGAGGTTATGCCTCCAATTCCAATAGGAGCCCACCTATTATTCT 5689

Gorilla NNNNNNNNNNNNNNNNNNNNNNTATGCCTCCAATTCCAATAGGAGNNNNNNNNNNNNNNN 5689

***********************

Human GGCTCCTACAATAAAAGAGACAATAAGGGCTCATATGTGGCAAGATTGTGTTATAAGGAG 5752

Chimpanzee GGCTCCTACAATAAAAGAGACAATAAGGGCTCATATGTGGCAAGATTGTGTTATAAGGAG 5749

Gorilla NNNNNNNNNNNNNNNNNNNNNNNNNNNNNNNNNNNNNNNNNNAGATTGTGNNNNNNNNNN 5749

********

Human ACTTTGTAACTCTTGTTCAGTGCATATGGAGATGGGAGTTGCTATAAAGAAGAGAAAGCA 5812

Chimpanzee ACTTTGTAACTCTTGTTCAGTGCATATGGACATGGGAGTTGCTATAAAGAAGAGAAAGCA 5809

Gorilla NNNNNNNAACTCTTGTNNNNNNNNNNNNNNNNNNNNNNNNNNNNNNNNNNNNNNNNNNNN 5809

*********

Human TAATTCTTTTGGAGTGCTATTTAGGCATCAACAGGCTGTGTTATTATAGATGAAAAAGAT 5872

Chimpanzee TAATTCTTTTGGAGTGCTATTTAGGCATCAACAGGCTGTGTTATTATAGATGAAAAAGAT 5869

Gorilla NNNNNNNNNNNNAGTGCTATTTAGGCATNNNNNNNNNNNNNNNNNNNNNNNNNNNNNNNN 5869

****************

Human GCCTGAAGTTAGACAGGTGAAACCTGACATTACTGACATCCAGGAATGACATTGGGGGGT 5932

Chimpanzee GCCTGAAGTTAGACAGGTGAAACCTGACATTACTGACATCCAGGAATGACATTGGGAGGT 5929

Gorilla NNNNNNNGTTAGACAGGNNNNNNNNNNNNNNNNNNNCATCCAGNNNNNNNNNNNNNNGGN 5929

********** ******* **

Human GTTTTTATTGAGAGTCATGCTGAAGTTCATGCATGTGAGATTTGAGGCCTCTGTGGCTGG 5992

Chimpanzee GTTTTTATTGAGAGTCATGCTGAAGTTCATGCATGTGAGATTTGAGGCCTCTGTGGCTGG 5989

Gorilla NNNNNNNNNNNNNNNNNNNNNNNNNNNNNNNNNNNNNNNNNTTGAGGCCTCTGTGGCTGG 5989

*******************

Human TAAATTGGTGACTATGGGACCGATTAATTTGGTGGTGTTTAGGACTGGGGTGGATAAGTT 6052

Chimpanzee TAAATTGGTGACTATGGGACCGATTAATTTGGTGGTGTTTAGGACTGGGATGGATAAGTT 6049

Gorilla TAAATTGGTGACTATGGGACCGATTAATTTGGTGGTGTTTAGGACTGGGGTGGATAAGTT 6049

************************************************* **********

Human CCACTATCTGGGGACAGGGACTGGGACATATGGTTGGAAACACAAGGGGAGACACATCCA 6112

Chimpanzee CCACTGTCTGGGGACAGGGACTGGGACATATGGCTGGAAACACAAGGGGAGACACATCCA 6109

Gorilla CCACTGTCTGGGGACAGGGACTGGGACACATGGTTGNNNNNACAAGGGGAGACACATTCA 6109

***** ********************** **** ** **************** **

Human ATAGTTGGTTGGATTATTAGGAGAGGTCTCTTGTATTCTTGTAAGGGTGGTGTTAAACAG 6172

Chimpanzee ATAGTTGGTTGGATTATCAGGAGAGGTCTCTTGTATTCTTGTAAGGGTGGTGTTAAACAG 6169

Gorilla ATAGTTGGTTGGATTATTAGGAGAGGTCTCTTGTATTCCTGTAAGGGTGGTGTTAAACAG 6169

***************** ******************** *********************

Human GCTCCAAGGAACAGTTTTATAACATTCTTTTCTCATGTAAGTATGAGTGGCAAAAGATGT 6232

Chimpanzee GCTCCAAGGAAGAGTTTTATAACATTCTTTTCTCATGTAAGTATGAGTGGCAAAAGATGT 6229

Gorilla GCTCCAAGGAACAGTTTTATAACATTCTTTTCTCATGTAAGTATGAGTGGCAAAATATGT 6229

*********** ******************************************* ****

Human GGGTGTTTGGAAGGGGCTACCCAATCTGCAAGAGTTTCTGATAGAATGCCAGTTGTCTCC 6292

Chimpanzee GGCTGTTTGGAAGGGGCTACCCAATCTGCAAGAGTTTCTGATAGAATGCCAGTTGTCTCC 6289

Gorilla GGGTGTTTGGANNNNNNNNNNNNNNNNNNNNNNNNNNNNNNNNNNNTGCCAGTTGTCTCC 6289

** ******** **************

Human TCCAGGGGAGGCACACTGACACGGGGTGTGTGTTGTTGTTGGAGAAAGGGACACGAGTAT 6352

Chimpanzee TCCAGGGGAGGCACACTGACACGGG-TGTGTGTTGTTGTTGGAGAAAGGGACACGAG--- 6345

Gorilla TCCACGGGAGGCACACTGACATGGGGTGTGTGTTGTTGTTGGAGAAAGGGACACGAGTAT 6349

**** **************** *** *******************************

Human GAAGAGGATGACAGGAAGCCTAAGAGGAATCATGAAGTGTTGTAAGTGAAATGGTCACAG 6412

Chimpanzee ------------------------AGGAATCATGAAGTGTTGTAAGTGAAATGGTCACAG 6381

Gorilla GAAGAGGATGGCAGGAAGCCTAAGAGGAATTATGAAGTGTTGTAAGTGAAATGGTCNNNN 6409

****** *************************

Human GAAAGAGGAGGGAAAAAGGAGTGAATTGCTGTTACAAGGAAGGCAGATAGAAGAAACGT- 6471

Chimpanzee GAAAGAGGAGGGAAAAAGGAGTGAATTGCTGTTACAAGGAAGGAAGATAGAAGAAACGTT 6441

Gorilla NNNNNNNNNNNNNNNNNNNNNNNNNNNNNNNNNNNNNNNNNNNNNNNNNNNNNNNNNNN- 6468

Human ------------------------------------------------------------

Chimpanzee GATGTGATTAAGATTTTTGTCCCACCCGGAGCTACAGTATATAATCCTACTGCAAAGAGT 6501

Gorilla ------------------------------------------------------------

Human --------------GCTTAATAATGTAACAAAACAGTAGAAGTCTTCCATTAAAGAGAGC 6517

Chimpanzee ATAGTTAGTATACTGCTTAATAGTGTAACAAAACAGTAGAAGTCTCCCATTAAAGAGAGC 6561

Gorilla --------------NNNNNNNNNNNNNNNNAAACAGTAGAAGTCTTCCATTAAAGAGAGC 6514

*************** **************

Human AAGGAGAGAAGTTAAAGATCATGTTGG-TTTTTTTTTTTTTTTTTTTTCCCCACTGATCC 6576

Chimpanzee AAGGAGAGAAGTTAAAGATCATGTTGGGTTTTTTTTTTTTTTTTTCCCCCCCACTGATCC 6621

Gorilla AAGGAGAGAAGTTAAAGATCATGTTGNNNNNNNNNNNNNNNNNNNNNNNNNNNNNNNNNN 6574

**************************

Human TCATTTAGGTAAGGAGAAGTCCCTCTTCAGGATTAACTGCAGGATTTTTTCTGGTCTGAA 6636

Chimpanzee TCCTTTAGGTAAGGAGAAGTCCCTCTTTAGGATTAACTGCAGGGTTTTTTCTGGTCTGAG 6681

Gorilla NNNNNNNNNNNNNNNNNNNNNNNNNNNNNNNNNNNNNNNNNNNNNNNNNNNNNNNNNNNN 6634

Human GTGTTCCTTTCCAAAATAGGAGATGCAGGTCCTCTTGTGGTGGCCAGGTGTATCGAAGCT 6696

Chimpanzee GTGTTCCTTTCCAAAATAGGAGATGCAGGTCCTCTTGTGGCGGCCAGGTGTATCGAAGCT 6741

Gorilla NNNNNNNNNNNNNNNNNNNNNNNNNNNNNNNNNNNNNNNNNNNNNNNNNNNNNNNNNNNN 6694

Human GGTCTGGCTGACTTGTGACTCCTGAGTTGATGATTCTGTAAGTTTCTCAGGAGGTGTCCA 6756

Chimpanzee GGTCTGGCTGACTTGTGACTCGTGAGTTGATGATCCTGTAAGTTTCTCAGGAGGTGTCCA 6801

Gorilla NNNNNNNNNNNNNNNNNNNNNNNNNNNNNNNNNNNNNNNNNNNNNNNNNNNNNNNNNNNN 6754

Human AGGCTTAATTTGGGTGTGGTGAATCCAGGATTCCATTCCCGTCACTTTAACTGCAGTGGG 6816

Chimpanzee AGGTTTAATTTGGGTGTGGTGAATCCAGGACTCCATTCCGATCACTTTAACTGCAGTGGG 6861

Gorilla NNNNNNNNNNNNNNNNNNNNNNNNNNNNNNNNNNNNCCTG-TNNNNTTNACTGCAGTGGG 6813

* * ** ***********

Human GGTAGAGAGGATTACCAAGTATGGTTCCTCCCATTAGGGATCCATAGAAGGAGAGCTAGA 6876

Chimpanzee GGTAGAGAGGATTACCAAGTATGGTTCCTCCCATAAGGGATCCATAGAAGGAGAGCTAGA 6921

Gorilla GGTAGAGAGGATTACCAAGTATGGTTCCTGCCATAAGGGATCCATAGAAGTANNNGTNNA 6873

***************************** **** *************** * * *

Human GGGGAGAGACTTGACCAATACTAGATCTCCTGGTTGAAACATCTCTATTCCTTTTTCCCT 6936

Chimpanzee TGGGAGAGACTTGACCAATACTAGATCTCCTGGTTGAAACAACTGTATTCCTTTTTCCCT 6981

Gorilla GGGGAGAGACTTGACCAATACTAGATCTCCTGGTTGAAACAACTCTATTCCTTTTCCCCT 6933

**************************************** ** ********** ****

Human GTCACACTTTTCAGGTAAAGTTTTAAGATTTTGTTGATATTTTGCCAGAGAAGTTATATC 6996

Chimpanzee GTCACACTTTTCAGGTAAAGTTTTAAGATTTTGTTGACATTTTGCCAGAGAAGTTATATC 7041

Gorilla GTCACACTTTTCAGGTAAAGTTTTAAGATTTTGTTGATATTTTGCCAGAGAAGTTATANN 6993

************************************* ********************

Human TTTTACTAAATTGGCCTTTTCCTGATTGAGCAGGAGGTCATCTGTGAGAAAAGCCATCCA 7056

Chimpanzee TTTTAGTAAATTGGCCTTTTCCTGATTGAGCAGGAGGTCATCTGTGAGAAAAGCCATCCA 7101

Gorilla NNNNNNNAAATTGGCCTTTTCCTGATTAAGCAGGAGGTCATCTNNGNNNNNNNNCATCCA 7053

******************** *************** * ******

Human TACAACACTTCATATGGACTGAGCCCCATTCTGTGAGGGGAGTTTCAGATTCTTAATAAG 7116

Chimpanzee TACAACATTTCATATGGACTGAGCCCCATTCTGTGAGTGGAGTTTCAGATTCTTAATAAG 7161

Gorilla TACAACATTTCATATGGACTGAGCCCCATTCTGTGAGGGGAGTTTCAGATTCTTAATAAG 7113

******* ***************************** **********************

Human TCCATGGGCAACAGAGTGGGCCGAGCAAGATGAGTTTCTTGCTCTAGTTTTCTCAAATGC 7176

Chimpanzee GCCATGGGCAACAGAGTGGGCCGAGCAAGATGAGTTTCTTGCTCTAGTTTTCTCAAATGC 7221

Gorilla GCCATGGGCAACAGAGTGGGCCGAGCAAGATGAGTTTCTTGCTCTAGTTTTCTCAAATGC 7173

***********************************************************

Human CTCTTGAGAGTTTCATTTGCCTTTTCAACTTTCCTTGAGGATTGTGCCCTCCAGGCACAG 7236

Chimpanzee CTCTTGAGAGTTTCATTTGCCCTTTCAACTTTCCTTGAGGATTGTGCCCTCCAGGCACAG 7281

Gorilla CTCTTGAGAGTTTCATTTGCCTTTTCAACTTTCCTTGAGGATTGTGCCCTCCAGGCACAG 7233

********************* **************************************

Human TGAAGTTGATATTGTATTCCTAGTGCCTTGGAAATTCCTTAAGTTGTTGTAGCTTTAAAA 7296

Chimpanzee TGAAGTTGATATTGTATTCCTAGTGCCTTGGAAATTCCTTAAGTTGTTGTAGCTTTAAAA 7341

Gorilla NNNNNNNNNNNNNNNNNNNNNNNNNNNNNNNNNNNNNNNNNNNNNNNNNNNNNNNNNNNN 7293

Human GCTGGACAATTGTCACTCTATAAGCTATGGGAAGTCCAAATCTAGGAATTATTTCATGAA 7356

Chimpanzee GCTGGACAGTTGTCACTCTGTAAGCTATGGGAAGTCCAAATCTAGGAATTATTTCATGAA 7401

Gorilla NNNNNACAATTGTCACTCAGTAAGCTATGGGAAGTCCAAATNNNNNNNNNNNNNNNNNNN 7353

*** ********* *********************

Human CTAAGACTTTAACCACTTCTTGGACCTTCTCTATTCTACAAGGGAAGGCTTCTACTCAAT 7416

Chimpanzee CTAAGACTTTAACCACTTCTTGGACCTTCTCTATTCTACAAGGGAAGGCTTCTACTCAAT 7461

Gorilla NNAAGACTTTAACCACTTCTTNNNNNNNNNCTATTCTACANNNNNNNNNNNNNNNNNNNN 7413

******************* **********

Human TTGTAAAGGTATCAACCCAGACCAATAAGTATTGAAATTCTCTTGGGTTTGGCATATGGG 7476

Chimpanzee TTGTAAAGGTTTCAACCCAGACCAATAAGTATTGAAATTCTCTTGGGTTTGGCATATGGG 7521

Gorilla NNNNNNNNNNNNNNNNNNAGACCAATAAGTATTGAAATTCTCTTGGGTTTGGCATATGGG 7473

******************************************

Human TAAAATCTAACTGCCAGTCCTCTCTGGGATAATGTCCTGTTCTTTGTCCTCCTGGAGAGG 7536

Chimpanzee TAAAATCTAACTGCCAGTCCTCTCTGGGATAATGTCCTGTTCTTTGTCCTCCTGGAGAGG 7581

Gorilla TAAAATCTAACTGCCAGTCCTCTCTGGGATAATGNNNNNNNCTTTGTCCTCCTGGAGAGG 7533

********************************** *******************

Human CCTTACAGTGGGCCAAGGGGTTGTTCTTTTGGCACATTTCACAGGCTTTGACTACTTGCC 7596

Chimpanzee CCTTACAGTGGGCCAAGGGGTTGTTCTTTTGGCACATTTCACAGGCTTTGACTACTTGCC 7641

Gorilla CCTTACAGTGGGCCAAGGGGTTGTTCTTTTGGCACATTTCACAGGCTTTGACTACTTGCC 7593

************************************************************

Human TGATGGTTTTGAGGAGGTTTGGCCCTGTAAATAGAGACGTGGTCATGTTATGGGTACTTT 7656

Chimpanzee TGATGGTTTTGAGGAGGTTTGGCCCTGTAAATAGAGACGTGGTCATGTTATGGGTACTTT 7701

Gorilla TGATGGTTTTGAGGAGGTTTGGCCNNNTAAATAGAGATGTGGTCATGTTATGGGTACTTT 7653

************************ ********** **********************

Human CAATAACAGTATGAAAAGTTTGGTGAAAGGTTTTAAGTATTTTCCAGTGGCTGGCTTCAG 7716

Chimpanzee CAATAACAGTATGAAAAGTTTGGTGGAAGGTTTTAAGCATTTTCCAGTGGCTGGCTTCAG 7761

Gorilla CAATAACAGTATGAAAAGTTTGGTGAAAGGTTTTAAGTATTTTCCAGTGGCTGGCTTCAG 7713

************************* *********** **********************

Human GTATGAGCACCTTTCCTTCCTATGTTATTAGCCACCCCGACGGGAGAAAACTATGTCCTT 7776

Chimpanzee GTATGAGCACCTTTCCTTCCTATGTTATTAGCCACCCCGATGGGAGAAAACTATGTCCTT 7821

Gorilla GTATGAGCACCTTTCCTTCNNNNGTTATTANCCACCCCGATGNGNGAAAACTATGTCCTT 7773

******************* ******* ********* * * ***************

Human GTGAAAGTCCCCATTCTATTTCAGTTGGAGAGTACTGGGGCTTAACCTCCTGGAGGGAGG 7836

Chimpanzee GTGAAAGTCCCCATTCTATTTCAGTTGGAGAGTACTGGGGCTTAACCTCCTGGAGGGAGG 7881

Gorilla GTGAAAGTCCCCATTCTATTTCAGTTNNNGAATACTGGGGCTTAACCTCCTGGAGGGAGG 7833

************************** ** ****************************

Human TTTCTCCATACCAGGTGTCCTTCCTTGGGCATTTCTGAAGGAAACTCCTGCCTGGCAGCA 7896

Chimpanzee TTTCTCCATACCAGGTGTCCTTCCTTGGGCATTTCTGAAGGAAACTCCTGCCTGGCAGCA 7941

Gorilla TTTCTCCATACCAGGTGTCCTTCCTTGGGCATTTCTGAAGGAAACTCCTGCCTGGCAGCA 7893

************************************************************

Human ATTTTGGCCTCAGACTCTGTTTGGTACTTTCCTTCTGCTTCTTCTCCTTCACCTTTCTGA 7956

Chimpanzee ATTTTGGCCTCAGACTCTGTTTGGCACTTTCCTTCTGCTTCTTCTCCTTCACCTTTCTGA 8001

Gorilla ATTTTGGCNNNNNNNNNNNNNNNNNNNNNNNNNNNNNNNNNNNNNNNNNNNNNNNNNNNN 7953

********

Human TGG----CCCCAGCAGTGTAAAACTGCCACCTCTTTAGGTTTTTGCACTGTGTGCATTAA 8012

Chimpanzee TGGATGGCCCCAGCAGTGTAAAACTGCCACCTCTTTAGGTTTTTGCACTGTGTGCATTAA 8061

Gorilla NNNN----CCCAGCAGTGTAAAACTGCCACCTCTTTAGGTTTTTGCACTGTGTGCATTAA 8009

****************************************************

Human TTCCATGATTTATCTGTGGTATTTAATAGGTGTTCCTCCGGAGGTTAAGAACTCTCTTTC 8072

Chimpanzee TTCCATGATTTATCTGTGGCATTTAATAGGTGTTCCTCTGGAGGTTAAGAACTCTCTTTC 8121

Gorilla TTCCATGATTTATCTGTGGTATTTAATAGGTGTTCCTCCGGAGGTTAAGAACTCNNNNTN 8069

******************* ****************** *************** *

Human TTTCCATATTGCAGCATGTGCATGTAAGACTAGAAAAGCATACTTGCTATCTGTGTATAC 8132

Chimpanzee TTTCCATATTGCAGCATGTGCATGTAAGACTAGAAAAGCATACTTGCTGTCTGTGTATAC 8181

Gorilla NNNNCATANNNNNNNNNNNNNNNNNNNNNNNNNNAANNNNNNNTTGCTATCTGTGTATAC 8129

**** ** ***** ***********

Human ATTTATGCTTTCTTCTTCTCCCAGTTCTAAGGCTCAGATTAGCACAATGAATTCTGCTAA 8192

Chimpanzee ATTTATGCTTTCTTCTTCTCCCAGTTCTAAGGCTCAGATTAGCACAATGAATTCTGCTAA 8241

Gorilla ATTTATGCTTTATTCTTCTCCCAGTTNNNNGGCTCAGATTAGCACAATGCATTCTGGTAA 8189

*********** ************** ******************* ****** ***

Human CTGAGCACTGGTCCCTAGAGGAAGAAGTTTATTTTCAAGTACTGCTGTATCACTAACTAT 8252

Chimpanzee CTGAGCACTGGTCCCTAGAGGAAGAAGTTTATTTTCAAGTACTGCTGTATCACTAACTAT 8301

Gorilla CTGAGCACTGTTCCCCAGAGGAAGAAGTTTATTTTCAAGTACTGCTGTATCACTAACTAT 8249

********** **** ********************************************

Human GGCATAGCCTGCCTTTTGTACCCAATTTTCTACAAATGAACTTCCATCAGAGTACAGATT 8312

Chimpanzee GGCATAGCCTGCCTTTTGTACCCAATTTTCTACAAATGAACTTCCATCAGAGTACAGATT 8361

Gorilla GGCATAGCCTGCCTTTTGTACCCAATTTTCTACAAATNNNNNNCCANNNNNNNNNNNNNN 8309

************************************* ***

Human AAGGTCAGGGTTAGTTAAGGGGACTTCTAAGAGATCTTTTCGGGTGGCAAAAGTATGAGC 8372

Chimpanzee AAGGTCAGGGTCAGTTAAGGGGACTTCTAAGAGATCTTTTCGGGTGGCAAAAGTATGAGC 8421

Gorilla NNNNTCAGGGTTAGTTAAGGGGACTTCTAAGAGATCTTTTCGGGTNGCAANNNNNNNNNN 8369

******* ********************************* ****

Human TAAAATTTGTTGGCAGTCATACTCAATTGGTTCCTCACCCTCTGGGAGAAAAGTGGCAGG 8432

Chimpanzee TAAAATTTGTTGGCAGTCATACTCAATTGGCTCCTCACCCTCTGGGAGAAAAGTGTCAGG 8481

Gorilla NNNNNNNNGTTGGCAGTCATACTCAATTGGTTCCTCACCCTCTGGGAGAAAAGTGGCAGG 8429

********************** ************************ ****

Human ATTGAGGGCCACACACATGCATATTTGAAATACTGGTACTCAAGGAGTGGCGCCTGGTAT 8492

Chimpanzee ATTGAGGGCCACACACATGCATATTTGAAATACTGGTACTCAAGTAGTAGTGCCTGGTAT 8541

Gorilla NNNNNNNNNNNNNNNNNNNNNNNNNNNNNNNNNNNNNNNNNNNNNNNNNNNNNNNNNNNN 8489

Human TTAAGTAGTCCACTATCTGACAGCCACAGACTTCCTTTAGCATTTAATATACCACTTACA 8552

Chimpanzee TTAAGTAGTCCATTATCTGACAGCCACAGACTTCGTTTAGCATTTAATATACCATTTACA 8601

Gorilla NNNNNNNNNNNNNNNNNNNNNNNNNNNNNNNNNNNNNNAGCATTTAATATACCACTTACA 8549

**************** *****

Human TCATGAGTGTTCCAGAAAGTGAGATCCTTTCCTTGTATTATTTTAACAGCCTCTGACACT 8612

Chimpanzee TCATGAGTGGTCCAGAAAGTGAGATCTTTTCCTTGTATTATTTTAACAGCCTCTGACACT 8661

Gorilla TCATGAGTGGTCCANNNNNNNNNNNNNNNNNNNNNNNNNNNNNNNNNNNNNNNNNNNNNT 8609

********* **** *

Human AAGATGGCTACTGCTGCAACCACCGATAAACAGTGAGGCCAGCCTTTGACTACTACATCA 8672

Chimpanzee AAGATGGCTACTGCTGCAACCACCCATAAACGGTGAGGCCAGCCTTTGACTACTACATCA 8721

Gorilla AAGATGGCTACTGCTGCAACCACCNNNNNACAGTGAGGCCAGCCTTTGACTACTACATCA 8669

************************ ** ****************************

Human ATTTCTTTACTTAGGTATGCCACTGGTTGTGGGGCTGTACCAGGAGTCTGAGTAAGAACT 8732

Chimpanzee ATTTCTTTACTTAGGTATGCCACTGGTTGTGGGGCTGTACCAGGAGTCTGAGTAAGAACT 8781

Gorilla ATTTCTTTACTTAGNNNNNNNNNNNNNNNNNNNNNNNNNNNNNNNNNNNNNNNNNNNNNN 8729

**************

Human CCAAGAGCTATTCCTACTCTTTCAGTGACATATAAAGATAAATTTTGTCCTGGGGGATGC 8792

Chimpanzee CCAAGAGCTATTCCTACTCTTTCAGTGACATATAAAGATAAATTTTGTCCTGGGGGATGC 8841

Gorilla NNNNNAGCTATTCCTACNCTTTCAGTGACATATAAAGATAAATTTTGTCCTGGGGGATNN 8789

************ ****************************************

Human TCAGGGCTGGAGCTTGTACCAGGGTCTGCTTTAAAGTTTTGAAGGCTGTTTCCACCTCCA 8852

Chimpanzee TCAGGGCTGGAGCTTGTACCAGGGTCTGCTTTAAAGTTTTGAAGGCTGTTTCCACCTCCA 8901

Gorilla NNNNNNNNNNNNNNNNNNCCAGGGTCTGCTTTAAAGTTTTGAAGNNNNNNNNNNNNNNNN 8849

**************************

Human GTTCCCATTCTACTAGGTGGGTATTTGCCTTCTGATTTTCCTTTATCAGGGGATAGAGTG 8912

Chimpanzee GTTCCCATTCTACTAGGTGGGTATTTGCCTTCTGATTCTCCTTTATCAGGGTATAGAGTG 8961

Gorilla NNNNNNNNNNNNNNNNNNNNNNNNNNNNNNNNNNNNNNNNNNTTNNNNNNNNNNNNNNNN 8909

**

Human GCCTGGCCATTTCACTCTATCCAGGAATTCACAGTTGGCAAAAGTCAGTGATTCCAAGGA 8972

Chimpanzee GCCTGGCCATTTCACTCTATCCAGGAATTCACAGTTGGCAAAAGTCAGTGATTCCAAGGA 9021

Gorilla NNNNNNNNNNNNNNNNCTNNCCANNNNNNNNNNNNNNNNAGAAGCCAGNGATTCCAAGGA 8969

** *** * *** *** ***********

Human ACCCCCCCTCAACTTTTTCAACGTCTTAGGGTGAGGGTAGGCCAGTATAAGATAAATACA 9032

Chimpanzee ACCCCCC-TCAACTTTTTCAACGTCTTAGGGTGAGGATAGGCCAGTATAAGATAAATACA 9080

Gorilla ACCCCCC-TCAACTTTTTCAACGTCTTAGGGTNNNNNNNNNNNNNNATAAGATAAATACA 9028

******* ************************ **************

Human CTCTTTGCTGAGGACTCCAGTTCATTTGGCCAGGATTAGGCCTAGACACTTAACTTGTTG 9092

Chimpanzee CTCTTTGCTGAGGACTCTAGTTCATTTGGCCAGGATTAGGCCTAGATACTTAACTTGTTG 9140

Gorilla CTCTTTGCTGANNNNNNNNNNNNNNNNNNNNNNNNNNNNNNNNNNNNNNNNNNNNNNNNN 9088

***********

Human TAGACAGAGCTGGGCCTTTGTCCTAGATGTTTTGTGCCCTTGATTAGCTAGAAAGTTTAA 9152

Chimpanzee TAGACAGAGCTGGGCCTTTGTCCTAGATGTTTTGTGCCCTTGATTAGCTAGAAAGTTTAA 9200

Gorilla NNNNNNNNNNNNNNNNNNNNNNNNNNNNNNNNNNNNNNNNNNNNNNNNNNNNNNNNNNNN 9148

Human GAGGTCTAGAGTAGCCTGCTAACATGTGGCTTCCAAACTCATAGCCAGAAGTATGTCATC 9212

Chimpanzee GAGGTCTAGAGTAACCTGCTAACATGTGGCTTCCAAACTCATAGCCAGAAGTATGTCATC 9260

Gorilla NNNNNNNNNNNNNNGCTGCTAACATGTGGCTTCCAAACTCATAGCCAGAAGTATGTCATC 9208

*********************************************

Human CATGTATTGAA-GGAACAGAGTGCCTGGACTGGAGAATAGGCCTAGGTCCCGGGCCAGTG 9271

Chimpanzee CACGTATTGAAAGGAACAGAGTGCCTGGACTTGAGAATAGGCCTAGGTCCCGGGCCAGTG 9320

Gorilla CACGTATTGAA-GGAACAGAGTGCCTGGACTTGAGAATAGGCTTAGGTCCCGGGCCAGTG 9267

** ******** ******************* ********** *****************

Human CCTGCCCAAACAGATGAGGGCTATCTCTAAACCCTTGGGGTAAGACTGTCCACATAAGCT 9331

Chimpanzee CCTGCCCAAACAGATGAGGGCTATCTCTAAACCCTTGGGATAAGACTGTCCACATAAGCT 9380

Gorilla CCTGACCANATAGATGGGGGCTATCTCTAAACCCTTGGGGTAAGACGGTCCACATAAGCT 9327

**** *** * ***** ********************** ****** *************

Human GGGACATGTGCTCTGTATAATCCTCAAAGGCAAAGAGGAACTGGGAGTCAGAGTGCAGAA 9391

Chimpanzee GGGACATGTGCTCTGTATAATCCTCAAAGGCAAAGAGGAACTGGGAGTCAGAGTGCAGAA 9440

Gorilla NNNNNNNNNNNNNNNNANNNNNNNNNNNNNNNNNNNNNNNNNNNNNNNNNNNNNNNNNNN 9387

*

Human GAAGGCATCTTTGAGGTCCAGAACAGCGAACCATTCTGCTTTTTCTGGTATCTGAGAGAG 9451

Chimpanzee GAAGGCATCTTTGAGGTCCAGAACAGTGAACCATTCTGCTTTTTCTGGTATCTGAGAGAG 9500

Gorilla NNNNNNNNNNNNNNNNNNNNNNNNNNNNNNNNNNNNNNNNNNNNNNNNNNNNNNNNNNNN 9447

Human CCGTGTATAGGGGTTGGGTACAGCTGGATATAAAGTAATTACTTCTTCATTGATGATTCT 9511

Chimpanzee CAGTGTATAGGGGTTGGGTACAGCTGGATATAAAGTAATTACTTCTTCATTGATGATTCT 9560

Gorilla NNNNNNNNNNNNNNNNNNNNNNNNNNNNNNNNNNNNNNNNNNNNNNNNNNNNNNNNNNNN 9507

Human GAGGTCTTGCACTAGTCTCCACTGACCATTTGGTTTTTGTATTCCTAGGATTGGGGTGTT 9571

Chimpanzee GAGGTCTTGCACTAGTCTCCACTGACCATTTGGTTTTTGTATTCCTAGGATTGGGGTGTT 9620

Gorilla NNNNNNNNNNNNNNNTCTCCACTGACCATTNNNNNNNNNNNNNNNNNNNNNNNNNNNNNN 9567

***************

Human GCAATGACTGCTTACATTTTCTTACTAAGCCTTGAGCTTTTAACTGTCTAAAAATATCTT 9631

Chimpanzee GCAATGACTGCT-ACATTTTCTTACTAAGCCTTGAGCTTTTAACTGTCTAAAAATATCTT 9679

Gorilla NNNNNNNNNNNNNNNNNNNNNNNNNNNNNNNNNNNNNNNNNNNNNNNNTAAAAATATCTT 9627

************

Human GTAATCCTTTGTGAGCTTCAGGCCTTAAGGGATATTGACTTTGATAAGGAAAAGAGGTGG 9691

Chimpanzee GTAATCCTTTGTGAGCTTCAGGCCTTAAGGGATATTGACTTTGATAAGGAAAAGAGGTGG 9739

Gorilla GTAANNNNNNNNNNNNNNNNNNNNNNNNNNNNNNNNNNNNNTGATAAGGAAAAGAGGTGG 9687

**** *******************

Human GGTCTTTTAGCCTGATTTTAACTGGATGGGCATTCTTTGCCCTTCTGAATTGTCCATCCA 9751

Chimpanzee GGTCTTTTAGCCTGATTTTAACTGGATGGGCATTCTTTGCCCTTCTGAATTGTCCATCCA 9799

Gorilla GGTCTTTTAGCCTGATTTTAACTGGATGGGCATTCTTTGCCCTTCTGAATTTTCCATCCA 9747

*************************************************** ********

Human AGGACCAGACTTCAGAGTTGACTCATTCTTCAAGTAGGGGACAACAAAGGGGTAATTTGT 9811

Chimpanzee AGGACCAGACTTCAGAGTTGACTCATTCTTCAAGTAGGGGACAACAAAGGGGTAATTTGT 9859

Gorilla AGNNNNNNNNNNNAGAGTNNNNNNNNNNNNNNNNNNGGGGACAACAAAGGGGTAATTCGT 9807

** ***** ********************* **

Human TCCCCATATTCATGTAGATAATAGCCACAGCTTTGGCTAATATGTCCCTCCCTAATAAAG 9871

Chimpanzee TCCCCATATTCATGTAGATAATAGCCCCAGATTTGGCTAATATGTCCCTCCCTAATAAAG 9919

Gorilla TCCCCATATTCATGNAGANNNNNNNNNNNNNNNNNNNNNNNNNNNNNNNNNNNNNNNNNN 9867

************** ***

Human GTGTGGGACTTTCAGGCATAACAAGAAAGGCATGTGAAAAGAGCAAAGTCTCCCAATTAC 9931

Chimpanzee GTGTGGGACTTTCAGGCATAACAAGAAAGGCATGTGAAAAGAGCAAAGTGTCCCAATTGT 9979

Gorilla NNNNNNNNNNNNNNNNNNNNNNNNNNNNNNNNNNNNNNNNNNNNNNNNNNNNNNNNNNNN 9927

Human AACTGAGGAGCTGGGAGAAATGCCTGGTTACAAGCTATCCTAAGATTCCTCGGATAGTAA 9991

Chimpanzee AGCTGAGGAGGTGAGAGAAATGCCTGGTTACAAGCTATCCTAAGATTCCTCAGATAGTAA 10039

Gorilla NNNNGAGGAGGTGAGAGAAATGCCTGGTTACAGGCTATCCTAAGATTCCTCGGATAGTAA 9987

****** ** ****************** ****************** ********

Human CAGACTTTGCAGACAGTCGTCTGGGGCAGGTGATTAAAATTGAGAAGGCAGGGCCAGTGT 10051

Chimpanzee CAGACTTTGCAGACAGTCGTCTGGGGCAGGTGATTAAAACTGAGAAGGCAGGGCCAGTGT 10099

Gorilla CAGACTTTGCAGACAGNNNNCTGGGGCAGGTGATTAAAATTGAGAAAGCAGGGCCAGTGT 10047

**************** ******************* ****** *************

Human CTAGGAGGAAGTCCACTTACTGGCCCTCAATGTTCAAACTTACCTGGGGCTCTTTGAGGG 10111

Chimpanzee CTAGGAGGGAGTCCACTTCCTGGCCCTCAATGTTCAAAGTTACCTGGGGCTCTTTGAGGG 10159

Gorilla CTAGGAGGAAGTCCACTTCCTGGCCCTCAATGTTCAAACTTACCTGGGGCTCTTTGAGGG 10107

******** ********* ******************* *********************

Human TGATGGCCTGAGCTGTCGCTTGCCCTGGCACCCTCAGTCCTGTTGCTGAATCACCTGACT 10171

Chimpanzee TGATGGCCTGAGCTGTCACTTGCCCTGGCACCCTCAATCCTGTTGCTGAATCACCTGACT 10219

Gorilla TGATGGCCTGNNNNNNNGCTTGNNNNGGTNNNCTCAGTCCTGTTGCTGAATCACCTGACT 10167

********** **** ** **** ***********************

Human GGGTGCTTCTGGCCCAGAGGGCCTTCATCCTCTGGGGCAGTGTGACTTCCAGTGATTACC 10231

Chimpanzee GGGTGCTTCTGGCCCAGAGGGCCTTCATCCTCTGGGGCAGTGTGACTTCCAGCGATTACC 10279

Gorilla GGGTGCTTCTGGACCAGAGGGCCTTCATCCTCTGGGGCAGTGTGACTTCCANNNNNNNNN 10227

************ **************************************

Human TTGGCATCTTGGACATGGGTAAGGGGGCAGCAACTGTCCTACTCCTCTTTTGGTTCCCTC 10291

Chimpanzee TTGGCATCTTGGACATGGGTAAGGGGGCAGCAACTGGCCTACTCCTCTTTTGGTTCCCTC 10339

Gorilla NNNNNNNNNNNNANNNNNNNNNNNNNNNNNNNNNNNNNNNNNNNNNNNNNNNNNNNNNNN 10287

*

Human TGAGCTGCCAAGGTCTGCCTGTCTGAGGGCCATGACTAAGGCTGGAGACTTTCTCTTATA 10351

Chimpanzee TGAGCTGCCAAGGTCTGCCTGTCTGAGGGCCATGACTAAGGCTGGAGACTTTCTCTTATA 10399

Gorilla NNNNNNNNNNNNNNNNNNNNNNNNNNNNNNNNNNNNNNNNNNNNNNNNNNNNNNNNNNNN 10347

Human TCGCTTTTCCCTTTTGGCATGTTCCTCTTGGTCCCTATTATAGAACACTGAGGTTGCCAA 10411

Chimpanzee TCTCTTTTCCCTTTTGGCATGTTCCTCTTGGTCCCTATTATAGAACACTGAGGTTGCCAA 10459

Gorilla NNNNNNNNNNNNTTTGGCATGTTCCTCTTGNNNCCTATTATAGGACACTGAGNNNNNNNN 10407

****************** ********** ********

Human GTTCAATAATGCCTCCAAATTTTGTTCTGGGCCTAAGGCAGACTTTTGGAGTTTTTTCCT 10471

Chimpanzee GTTCAATAATGCCTCCAAATTTTGTTCTGGGCCTAAGGCAGACTTTTGGAGTTTTTTCCT 10519

Gorilla NNNNNNNNNNNNNNNNNNNNNNNNNNNNNNGCCTAAGGCAGACTTTTGGAGTTTTTTCCT 10467

******************************

Human AATGTCAGTCACTGACTGGGTGATAAATTTATCCTTTATGATAAGTTGGCTTTCCATGGA 10531

Chimpanzee AATGTCAGTCACTGACTGGGTGATAAATTTATCCTTTATGATAAGTTGGCTTTCCATGGA 10579

Gorilla AATGTCAGTCACTGACTGGGTGATAAATTTATCCTTTATGATAAGTTGGCTTTCCATGGA 10527

************************************************************

Human ATCCAGAGCTAAGGAGGTGTGCTTTCTTAGGATCTCCTTTAACCTTTGTAGAAAAGTTGA 10591

Chimpanzee ATCCAGAGCTAAGGAGGTGTGCTTTCTTAGGGTCTCCTTTAACCTTTGTAGAAAAGTTGA 10639

Gorilla ATCCAGAGCTAAGGAGNNNNNNNNNNNTAGNNNNNNNNNNNNNNNNNNNNNNNNNNNNNN 10587

**************** ***

Human GGGGTTTTCCTCTTTTCCTTGCACAACTGTGGATAGCATTGAGTAGTTCATAGGCTTTTT 10651

Chimpanzee GGGGTTTTCCTCTTTTCCTTGCACAACTGTGGATAGCATTGAGTAGTTCATAGGCTTTTT 10699

Gorilla NNNNNNNNNNNNNTTTCCTTGCACAACTGTGGATAGCATTGAGTAGTTCNNNGGCTTTTT 10647

************************************ ********

Human CCTAGTTTTCCTCAACTCTTCCAAAATGCAACTTAGCAAATGCCTGTGGCTCCAGTCTCC 10711

Chimpanzee CCTAGTTTTCCTCAACTCTTCCAAAATGCAAATTAGCAAATGCCTGTGGCTCCAGTCTCC 10759

Gorilla CCTAGNNNNNNNNNNNNNNNNNNNNNNNNNNNNNNNNNNNNNNNNNNGGCTCCAGTCTCN 10707

***** ************

Human AGGATCTGAGTCAGTATCTCAGTGAGGGTTTACAGTAGGGACTGCCTTTTGCCCTGTGGG 10771

Chimpanzee AGGATCTGAGTCAGTATCTCAGTGAGGGTTTACAGTAGGGACTGCCTTTTGCCCTGTGGG 10819

Gorilla NNNNNNTGAGTCAGTATCTCAATGAGGGTTTACAGTAGGGACTGCNNNNNNNNNNNNNNN 10767

*************** ***********************

Human GAATTTTTCCCTCACCTTCTGGGCCATTTGATCATTTACCTGGCTAAGGTACCACGGATC 10831

Chimpanzee GAATTTTTCCCTCACCTTCTGGGCCATTTGATCATTTACCTGGCTAAGGTACCACAGATC 10879

Gorilla NNNNNTTTCCCTCACCTCCTGGGCCATTTGGTCATTTACCTGGCTAAGATACCACAGATC 10827

************ ************ ***************** ****** ****

Human CCCAAATTGCCAGGTTGTTGTTAAAGCTTCTTGCTTTTCAGTAGGACATAATGTCTAATC 10891

Chimpanzee CC-AAATTGCCAGGTTGTTGTTAAAGCTTCTTGCTTTTCAGTAGGACATAATGTCTAATC 10938

Gorilla CCCAAATTACCAGGTTGTTGTTAAAGCTTCTTGCTTTTCAGTAGGACACAATGTCTAATC 10887

** ***** *************************************** ***********

Human AAGAAGTAAAATTATATCTCTCCATGTTAGATCAAAGGACTGCCTTAATCCTTGCAAGGC 10951

Chimpanzee AAGAAGTAAAATTATATCTCTCCATGTTAGATCAAAGGACTGCCTTAATCCTTGCAAGAC 10998

Gorilla AAGAAGTAAAATTACATCTCTCCATGTTAGATCAAAGNNNNNNNNNNNNNNNNNNNNNNN 10947

************** **********************

Human ATCTATATACTTATCAGGTTCATCTGAGAATTTCCCTAAGTTTGACTTTATTTGTTTTAA 11011

Chimpanzee ATCTATATAGTTATCAGGATCATCTGAGAATTTCCCTAAGTTTGACTTTATTTGTTTTAA 11058

Gorilla NNNNATANNNNNNTCNNNNNNNNCTGAGAATTTCCCTAAGTTTGACTTTATTTGTTTTAA 11007

*** ** *************************************

Human GTCTGAGAGTGTGAAGAGACTCGTACAAGGAAGGGCCCAAATTCCTCTCCTACTGCTTGT 11071

Chimpanzee GTCTGAGAGTGTGAAGAGACACGTACAAGGAAGGGCCCAAATTCCTCTCCTACTGCTTGT 11118

Gorilla GTCTGAGAGTGTGAAGAGNNNNNNNNNNNNNNNNNNNNNNNNNNNNNNNNNNNNNNNNNN 11067

******************

Human AAGGGACATAGTTTGGGTGCCTGGCTTGGGGAGTTTACATTCTCTTTACCACTTCAGTGG 11131

Chimpanzee AAGGGACATAGTTTGGGTGCCTGGCTTGGGGAGTTTACATTCTCTTTACCACTTCAGTGG 11178

Gorilla NNNNNNNNNNGTNNNNNNGCCTGGCTNNNNNATTTTACATTCTCTNNNNNNNNNNNNNNN 11127

** ******** * ************

Human GGCAGGATGGAGGAGAGTCAGTGGGGGAGGAGAGTGGGGCTGAGGGAAGAGGCTGTGAGT 11191

Chimpanzee GGCAGGATGGAGGAGAGTCAGTGGGGGAAGAGAGTGGGGCTGAGGGAAGAGGCTGTGAGT 11238

Gorilla NNNNNNNNNNNNNNNNNNNNNNNNNNNNNNNNNNNNNNNNNNNNNNNNNNNNNNNNGAGT 11187

****

Human ATGGAGGTAACTGTGGGCCTTTGTCATTTTGGCAAAGCTTGTAGGCTTGGCACAGGGCAG 11251

Chimpanzee ATGGAGGTAACTGTGGGCCTTTGTCATTTTGGCAAAGCTTGTAGGCTTGGCACAGGGCAG 11298

Gorilla ATGGAGGTAACTGTGGGCCTTTGTCATTTTGGCAAAGCTTGTAGGCTTGGNNNNNNNNNG 11247

************************************************** *

Human TATTGTCATGAAGGGCAAAGAAAGCCTGTACATAAGGGACTATACTCTATTTATCTTCCT 11311

Chimpanzee TATTGTCATGAAGGGCAAAGAAAGCCTGTACATAAGGGACTATACTCTATTTATCTTCCT 11358

Gorilla TATNNNNNNNNNNNNNAANGAAAGCCTGTACATAAGGGGCTATACTCTATTTATCNNCCT 11307

*** ** ******************* **************** ***

Human GTTCACAGAAAAGATCTAGCTGTAGAAGGGTATTATAATTAATACTTCCCTCAGGGGGCC 11371

Chimpanzee GTTCACAGAAAAGATCTAGCTGTAGAAGGGTATTATAATTAATACTTCCCTCAGGGGGCC 11418

Gorilla GTTCATAGAAAAGATCTAGCTGTAGAAGGGTNNNNNNNNNNNNNNNNNCCTCAGGGGNCC 11367

***** ************************* ********* **

Human AAGTTTTTCCATTTTGTAAGAAATTCCATGGTCAGGCAGTGGTATAGAAGAAAATTAGCT 11431

Chimpanzee AAGTTTTTCCATTTTGTAAGAAATTCCGTGGCCAGGCAGTGGTATAGAAGAAAATTAGCT 11478

Gorilla AAGTTTTTCCATTTTGTAAGAAATNNNNNNGCNNNNNNNNNNNNNNNNNNNNNNNNNNNN 11427

************************ *

Human GCTTCTTTTGTAAGGTTTCAGGGTTGAATTTGTCCCAGTTATTCAGGATACATCTTAAGG 11491

Chimpanzee GCTTCTTTTGTAAGGTTTCAGGGTTGAATTTGTCCCAGTTATTCAGGATACATCTTAAGG 11538

Gorilla NNNNNNNNNNNNNNNNNNNNNGGTTGAATTTGNNNNNNNNNNNNNGGATACATNNNNNNN 11487

*********** ********

Human GGGTGTCCAGTTTTGAAGGTATGGAGCCCATCTGGAATTTTACACACAGAGATGCCCACA 11551

Chimpanzee GGGTGTCCAGTTTTGAAGGTATGGAGCCCATCTGTAATTTTACACACAGAGATGCCCACA 11598

Gorilla NNNNNNNNAGTTTTGAAGGTANNNNNCCCATCTGGAATTTTACACACAGAGATGCCCGCA 11547

************* ******** ********************** **

Human CCCCTGGTTAGTACTGGGACTCTTCTTCTCTTAGGGTGTCCCCCAAGGGTCAGGTCCTAT 11611

Chimpanzee CCCCTGGTTAGTACTGGGACTCTTCTTCTCTTAGGGTGTCCCCCAAGGGTCAGGTCCTAT 11658

Gorilla TCCCTGGTTAGTACTGGGACTTGTNNNNNNNNNNNNTGTCCTCCAAGNNNNAGGTCCTAT 11607

******************** * ***** ***** *********

Human TGTGCTCAAAGCTCATGGCTGCATTTCCTGAGCCCTCCGCCTGCCGGATTTAACCATGCT 11671

Chimpanzee TGTGCTCAAAGCTCATGGCTGCATTTCCTGAGCCCTCCGTCTACCGGATTTAACCATGCT 11718

Gorilla TGTGCTAAAAGCNNNNNNNNNNNNNNNNNNNNNNNNNNNNNNNNNNNNNNNNNNNNNNNN 11667

****** *****

Human TACCAGCAGGATGGAAACTTCCTTTGCCCCTGCCATGCACCCATTGACCACTAAATGGGG 11731

Chimpanzee TACCAGCAGGATGGAAACTTCCTTTGCCCCTGCCATGCACCCATTGACCACTAAATGGGG 11778

Gorilla NNNNNNNNNNNNNNNANNNNNNNNNNNNNNNNNNNNNNNNCCATTGACCACTAAATGGNN 11727

* ******************

Human CACAAGGACTGTTGGATTTATTGTGGTCCTTCCTCCAACACATCCTACCTTTTCCAGGGT 11791

Chimpanzee CACAAGGACTGTTGGATTTATTGTGGTCCTTCCTCCAACACATCCTACCTTTTCCAGGGT 11838

Gorilla NNNNNNGACTGTTNGNNNNATTGTGGTCCTTCCTCCAACACATCCTACCTTTTCCAGGGT 11787

******* * *****************************************

Human GACAAGGCCTGGGTCGGGGCACCACTGATGCTTGCATGCTAAGGCCCAATTTATGTGGGC 11851

Chimpanzee GACAAGGCCTGGGTCGGGGCACCACTGATGCTTGCATGCTAAAGCCCAATTTATGTGGGC 11898

Gorilla GACAAGGCCTGGNNNNNNGCACCACTGATGCTTGCAGGCTAAGGCCCAATTTATGTGGGC 11847

************ ****************** ***** *****************

Human CTGGTCATCAAAACTGTCCTTCAAGGAGAAATCTCTGAATTAGCAACAGGAGGCTTAAGA 11911

Chimpanzee CTGGTCATCAAAACTGTCCTTCAAGGATAAATCTCTGAATTAGCCACAGGAGGCTTAAGA 11958

Gorilla CTGGTCATCAAAACTGTCCTTCAAGGAGAAATCTNNNAATTANNNNNNNGAGGCTTAATA 11907

*************************** ****** ***** ********* *

Human AGCTTTAAGGGTGTGGTGGATGTCCTCTAGGCCAGGGCCGAGAGAACAGCTGCTGTACTC 11971

Chimpanzee AGCTTTAAGGGTGTGGTGGATGTCCTCTAGGCCAGGGCCGAGAGAACAGCTGCTGTACTC 12018

Gorilla AGCTNNAAGGGTGTGGTGGATGTCCTCTAGGCCAGGGCCGAGAGAACANNTGCTGTANNN 11967

**** ****************************************** *******

Human TAGCCTTCTGTCCCCACTTGCCATCAAAGTAGTAAGCCCCCCTTTCAAGGTGGTACTAGT 12031

Chimpanzee TAGCCTTCTGTCCCCACTTGCCATCAAAGTAGTAAGCCCCCCTTTCAAGGTGGTACTAGT 12078

Gorilla NNNNNTTCTGTCCCCACTTGCCATCAAAGTAGTAAGCCCCCCTTTCAAGGTGGTACTAGT 12027

*******************************************************

Human ATCATGTGTCCAAACTGACCGTATATTCACCCTTCCATTACCAATTATTGAATGGTTGAA 12091

Chimpanzee ATCACGTGTCCAAACTGACAGTATATTCACCCTTCCATTACCAATTATTGAATGGTTGAA 12138

Gorilla ATCATGTNNNNNNNNNNANNNNNNNNNNNCCCTTCCATTACCAATTATTGAATNATTNNN 12087

**** ** * ************************ **

Human ACAACAATTCAACAACTCTAAGACTTTGCCTATGCACAACAGATTGTCCTTGAGTGGCCC 12151

Chimpanzee ACAACCATTCAACAACTCTAAGACTTTGCCTATGCACCACAGATTGTCCTTGAGTGGCCC 12198

Gorilla NNNNNNNNNNNNNNNNNNNNNNNNTTTGCCTATGCACCACAGATTGTCCTTGAGTGGCCC 12147

************* **********************

Human CAGGTAGGAGAAAGCCTATCTGGGGAGCAATGGTGGAAATGCCCTAAGGCTTCTACTATC 12211

Chimpanzee CAGGTAGGTGAAAGTCTATCTGGGGAGCAATGGTGGAAGTGCCCTAAGGCTTCTACTATC 12258

Gorilla CAGGTAGGTGAANNNNNNNNNNNNNNNNNNNGNNNNNNNNNNNCTANNNCTTCTACTATC 12207

******** *** * *** ***********

Human CACATGAAAATTACAGACCTGTCTTTAAATTGTCCTGATGTGGGTGGCCATACACTATGA 12271

Chimpanzee CACATGAAAATTACAGACCTGTCTTTAAATTGTCCTGATGTGGGTGACCATACACTATGA 12318

Gorilla CACNTGAAAATTACAGACCTGTCTTTAAATTGTCCTGATGTGGGTGACCATACACTATGA 12267

*** ****************************************** *************

Human TGGGGGACTGGCCTTTCAAAGTGGCCATCAAATGGTGACACCTGCCTAAATCCCAGAGGG 12331

Chimpanzee TGGGGGACTGGCCTTTCAAAGTGGCCATCAAATGGTGACACCTGCCTAAATCCCAGAGGG 12378

Gorilla TGGGGGACTGGCCTTTCAAAGTGGCCATCAAATGGTGACACCTGCCTAAATCCCAGAAGG 12327

********************************************************* **

Human CAACATGAACAGAGGTCTTCTGGGCACCACCCCAAGAATTTAAGACTTCTAAATAGGGAA 12391

Chimpanzee CAACATGAACAGAGGTCTTCTGGGCACCACCCCAAGAATTTAAGACTTCTAAATAGGGAA 12438

Gorilla CAACATGAACAGAAGTCTTCTGGGCACCACCCCAAGAATTTANNNNNNNNNNNNNNNNNN 12387

************* ****************************

Human TCTTGATCCTGCCTAGCAGGAATAACCTACTTGTGAGATGAGGAAAGAAGTTTAGCCATT 12451

Chimpanzee TCTTGATCCTGCCTAGCAGGAATAATCTACTTGTGAGATGAGGAAAGAAGTTTAGCCATT 12498

Gorilla NNNNNNNNNNNNNNNNNNNNAATAACCTACTTGTGTGATAAGGAAAGAAGTTTAGCNNNN 12447

***** ********* *** ****************

Human GGACATAAGGACCCAGGAGGCAGGAGTCAGAAGATGTCTCATGCTCAGTAACCTCTGCAG 12511

Chimpanzee GGACATAAGGACCCAGGAGGCAGGAGTCAGAAGATGTCTCATGCTCAGTAACCTCTGCAG 12558

Gorilla NNNNNNNNNNNNNNNNNNNNNNNNNNNNNNNNNNNNNNNNNNNCTCAGTAACCTCTGCAG 12507

*****************

Human GGGTAGACTCTGGTGGGACCATGGTCTCAACCAGGGTATCTGGGAGACTAAGATGTTCAC 12571

Chimpanzee GGGTAGACTCTGGTGGGACCATGGTCTCAACCAGGGTATCTGG-AGACTAAGATGTTCAC 12617

Gorilla GGGTAGACTCTGGTGGGACCATGGTCTCAACCAGGGTNNNNGGGAGACTAAGATGTTCAC 12567

************************************* ** ****************

Human TGAGCACTCCCAGTTGTACTTTGGGACCACCATGGGAAGCAAAACTGGGAACAAATTCTC 12631

Chimpanzee TGAGCACTCCCAGTTGTACTTTGGGACCACCATGGAAAGCAAAACTGGGAACAAATTCTC 12677

Gorilla NGAGCACTCCCAGGTGTACTTTGGGACCACCATGGAAAGCAAAACTGGGAACAAATTCTC 12627

************ ********************* ************************

Human CCAACCCCAAAGAGTCGTGGGTTGTTAGAGAGCCCTTTTCCAGACAGCCTGACATCCGTG 12691

Chimpanzee CCAACCCCAAAGAGTCGTGGGTTGTTAGAGAGCCCTTTTCCAGACAGCCTGACATCCGTG 12737

Gorilla CCAACCCCAAAGAGTCGTGGGTTGTTAGAGAGCCCTTTTCCAGACAGCCTGACATCCGTG 12687

************************************************************

Human TCTTTAGTCCAGTGGCCATGCTAATTGCCTCTAAGTGGCCAACTGGTTTTTATCCTCCTA 12751

Chimpanzee TCTTTAGTCCAGTGGCCATGCTAATTGCCTCTAAGTGGCCAACTGGTTTTTATCCTCCTA 12797

Gorilla TCTTTAGTCCAGTGGCCATGCTAATTGCCTCTAAGTGGCCAACTGGTTTTTATCCTCCTA 12747

************************************************************

Human ATTCTAAGGAAGGATACAACAGAATAGCAAGAGAAATAAGTCCAATCATACCCACCACCT 12811

Chimpanzee ATTCTAATGAAGGATACAACAGAATAGCAAGAGAAGTAGGTCCAATCATACCCACCACCT 12857

Gorilla ATTCTAAGGAANNNNNNNNNNGAATAGNNNGAGAAANNNNNNNNNNNATACCCACCACCT 12807

******* *** ****** ***** *************

Human GAAGATCTAGGTGCTTTTCCTGGAGCCTCCTGGCTGGCTCACCGAAATGTCACAGCTAAG 12871

Chimpanzee GAAGATCTAGGTGCTTTTCCTGGAGCCTCCTGGCTGGCTCACCGAAATGTCACAGCTAAG 12917

Gorilla GAAGATCTNNNNNNNNNNNNNNNNNNNNNNNNNCTGGCTCANCGAAATGTCACAGCTAAG 12867

******** ******** ******************

Human GTTGTTGCCTAGCCATGCCAAAGAATTGGTGTGGTGGCTGAACCTGGGCAAGTGATAGTG 12931

Chimpanzee GTTGTTGCCTAGCCATGCCAAAGAATTGGTGTGGTGGCTGAACCTGGGCAAGTGACAGTG 12977

Gorilla GTTGTTGCCTAGCCATGCCANNGAATTGGTGTGGTGGCTGAACCTGGGCAAGTGATAGTG 12927

******************** ********************************* ****

Human ACATGGCCCGATAGAGAGAAAAAGCTGTAAGTTTTGTTGAGCAGAGTGAAAGTATAAAGC 12991

Chimpanzee ACATGGCCCGATGGAGAGAAAAAGCTGTACGTTTTGTTGAGCAGAGTGAAAGTACAAAGC 13037

Gorilla ACATGGCCCGATAGAGAGAAAAAGCTGTAAGTTTTGTTGAGCAGAGTGAAAGTACANAGC 12987

************ **************** ************************ * ***

Human TTCCACAGCATGGAAGGGATCCCAAATGTGTAGCCAGAATTAGAGTATATGAATGCCTTT 13051

Chimpanzee TTCCACAGCATGGAAGGGGTCCCAAATGTGTAGCCAGAATTAGAGTATATGAATGCCTTT 13097

Gorilla TTCCACAGCATGGAAGGGGTCCCAAATGTGTAGCCAGAATTAGAGTATATGAATGCCTTT 13047

****************** *****************************************

Human TAAACTCCATAAGGTGGAAAATACGTGCAACTTAGGTTTTATCTACTTTATGACCTTGCC 13111

Chimpanzee TAAACTCCATAAGGTGGAAAATATATGCAACTTAGGTTTTATCTACTTTATGACCTTGCC 13157

Gorilla TAAACTCCATAAGGTGGAAAATANNNNNNNNNNNNNNNNNNTCTACTTTATCACCTTGCC 13107

*********************** ********** ********

Human ACAGCATGGCAAAGGAGACAGGATTTTACAGGATTTTACAAAGTATGTTTACAAGGAATT 13171

Chimpanzee ACAGCATGGCAAAGGAGACAGGATTTTACAGGATTTTACAA-GTATGTTTACAAGGAATT 13216

Gorilla ACAGCATGGCAAAGGAGACAGGATTTTACAGGATTTTACAAAGTATGTTTACAAGGAATT 13167

***************************************** ******************

Human GGAATTGAGAGCATAGATAAGGTCTGCTGGTCACAGAAAAGTGGAAAGCTAACATTCCTT 13231

Chimpanzee GGAATTGAGAGCATAGATAAGGTCTGCTGGTCACAGAAAAGTGGACAGCTAACATTCCTT 13276

Gorilla NNNNNNNNNNNNNNNNNNNNNNNNNNNNNNNNNNNNNNNNNNNNNNNNNNNNNNNNNNNN 13227

Human TTACTTTAGTTTTGGGAGAGGGGGAAGGCAGAGAGGGAGAGAGGACACAGGAAAACTTAC 13291

Chimpanzee TTACTTTAGTTTTGGGAGAGGGGGAAGGCAGAGAGGGAGAGAGGACACAGGAAAACTTAC 13336

Gorilla NNNNNNNNNNNNNNNNNNNNNNNNNNNNNNGAGAGGGNNNNNNNNNNNNNNNNNACTTAC 13287

******* ******

Human AGCAAAATTGTTCCTGTTTATAGCTTTCTTGGGGAAGAAAACACATGCACAAATCCTGGT 13351

Chimpanzee AGCAAAATTGTTCCTGTTTATAGCTTTCTTGGGGAAGAAAACACATGCACAAACCCTGGT 13396

Gorilla AGCANNNNNNNNNNNNNNNNNNNNNNNNNNNNNNNNNNNNNNNNNNNNNNNNNNNNNNNN 13347

****

Human GTTAGGAATAGTTTAAGCATATATCTTCAATACTATCCATCCAGGATGGAAACAATTCCT 13411

Chimpanzee GTTAGGAATAGTTTAAGCATGTATCTTCAATACTATCCATCCAGGATGGAAATAATTCCT 13456

Gorilla NNNNNNNNNNNNNNNAGCATATANNNNNNNNACTATCCATCCATGATGGAAATAATTCCT 13407

***** ** ************ ******** *******

Human AATGCAGGAAATCAGTGAGTTTCACAGCTTTCTGAGCCCCTACTCAACCCAGGAAACCTA 13471

Chimpanzee AATGCAGGAAATCAGTGAGTTTCACAGCTTTCTGAGCCCCTACTCAACCCAGGAAACCTA 13516

Gorilla AATGCAGGAAATCAGTGAGTTTCACAGCTTTCTGAGCCCCTACTCAACCCAGGAAGCCTA 13467

******************************************************* ****

Human GCTAGCTTCTCCAGTCAATATTGCAAGTTATTACATTAAAAACGTAATTATCTGAACAAA 13531

Chimpanzee GCTAGCTTCTCCAGTCAATATTGCAAGTTATTACATTAAAAACGTAATTATCTGAACAAA 13576

Gorilla GCTAGCTTCTCCAGTCAATATTGCAAGTTATTACATTAAAAACGTAATTATCTGAACAAA 13527

************************************************************

Human GCTTTATGAATGACAGTGCTTAACAAGGTGGCAGTGAAAAAGTGAAGATAAAGAAGACAG 13591

Chimpanzee GCTTTATGAATGACAGTGCTTAACAAGGTGGCAGTGAAAAAGTGAAGATAAAGAAGACAG 13636

Gorilla GCTTTATGAATGATAGTGCTTAACAAGGTGGCAGTGAAAAAGTGAAGATAAAGAAGACAG 13587

************* **********************************************

Human GGTTTCAAGAACTGTATACCTACTTTGCAGCCATGTCACTAAACCAGTTGTGATCAGAAT 13651

Chimpanzee GGTTTCAAGAACTGTATACCTACTTTGCAGCCATGTCAATAAACCAGTTGTGATCAGAAT 13696

Gorilla GGTTTCAAGAACTGTATACCTACGTTGCAGCCATGTCAATAAACCAGTTGTGATCAGAAT 13647

*********************** ************** *********************

Human GACAATTCAAAGAAAGAGGAGTATTCTGTTTAAATTGTCTCTGAGCTTGAGGAGTAAGAA 13711

Chimpanzee GACAATTCAGAGAAAGAGGAGTATTCTGTTTAAATTGTCTCTGAGCTTGAGGAATAAGAA 13756

Gorilla GACAATTCAAAGAAAGAGGAGTATTCTGTTTAAATTGTCTCTGAGCTTGAGGAATAAGAA 13707

********* ******************************************* ******

Human GTATAAAACATGGGAGAAACTGATTGCATGGAAATATAGTGGAAACAAATTGTAGATGAG 13771

Chimpanzee GTATAAAACATAGGAGAGACTGATTGCATGGAAATATAGTGGAAACAAATTGTGGATGAG 13816

Gorilla GTATAAAACATGGGAGAAACTGATTGCATGAAAATATGGTGGAAACAAATTGTGGATGAG 13767

*********** ***** ************ ****** *************** ******

Human GGTTTGCATCCGCAGAAGCTAGTGATGGGATGGGAATTCAAGGAACTCCTAGTGAAATGC 13831

Chimpanzee AGTTTGCATCCACAGAAGCTAGTGATGGGATGGGAATTCAAGGAACTCCTAGTGAAATGC 13876

Gorilla AGTTTGCATCCGCAGAAGCTAGTGATGGGATGGGAATTCAAGGAACTCCTAGTGAAATGC 13827

********** ************************************************

Human TCAAACATTTTCAGGGACATAAGATAAAATTCCTGGAAAGTTTACAGAATTGGAAAAAGC 13891

Chimpanzee TCAAACATTTTCAGGGACATAAGAGAAAATTCCTGGGAAGTTTACAGAATTGGAAAAAGC 13936

Gorilla TCAAACATTTTCAGGGACATAAGAGAAAATTCCTGGAAAGTTTACAGAATTGGAAAAAGC 13887

************************ *********** ***********************

Human ACAGGCAGCACAGTCTCACTGTGAAAATAATTGCAGCTAAGTTATAATGGATGTTTAGAC 13951

Chimpanzee ACAGGCAGTACAGTCTCATTGTGAAAATAATTGCAGCTAAGTTATAATGGATGTTTAGAC 13996

Gorilla ACAGGCAGCAGAGTCTCACTGTGAAAATAATTGCAGCTAAGTTATAATGGATGTTTAGAC 13947

******** * ******* *****************************************

Human TATATCACATTTGGCACCAGCTATTTATGCTCATTTCATAATTTATCAGTGCATCAGTTG 14011

Chimpanzee TATATCACATTTGGCACCAGCTATTTATGCTCATTTCATAATTTATCAGTGCATCAGTTG 14056

Gorilla TATATCACATTTGGCACCAGCTATTTATGCTCATTTCATAATTTATCAGTGCATCAGTTG 14007

************************************************************

Human TACCAGGAAGATAATAATATTCAGCTGCTTTCCCAGAGTCTGAAATCATCTGCACACATT 14071

Chimpanzee TACCAGGCAGATAATAATATTCAGCTGCTTTCCCAGAGTCTGAAATCATCTGGACACATT 14116

Gorilla TACCAGGCAGATAATAATATTCAGCTGCTTTCCCAGAGTCTGAAATCATCTGGACACATT 14067

******* ******************************************** *******

Human CCAAGGGTGACAGATGGCAGACTTGCTAGGGGACAACTCTCTTCTTCCCCCTCCCATAGA 14131

Chimpanzee CCAAGGGTGACAGATGGCAGACTTGCTAGGGGACAACTGTCTTCTTCCCTCTCCCATAGA 14176

Gorilla CCAAGGGTGACAGATGGCAGCCTTGCTAGGGGACAACTCTCTTCTTCCCTCTCCCATAGA 14127

******************** ***************** ********** **********

Human AACCTTTCAAATTCTAGCACGTGTAAAAGAGGAAGCCATTTCCTCAAAGCACCATTTTTT 14191

Chimpanzee AACCTTTCAAATTCTAGCACGTGTAAAAGAGGAAGCCATTTCCTCAAAGCACCATTTTTT 14236

Gorilla AACCTTTCAAATTCTAGCATGTGTAAAAGAGGAAGCCATTTCCTCAAAGCACCATTTTTT 14187

******************* ****************************************

Human CTTTGCTTATTTCCCCTACCAAACTTCTTCCTTTGATTTTAAGTCCAACTGCAGTCCTTT 14251

Chimpanzee CTTTGCTTATTTCCCCTACCAAACTTCTTCCTTTGATTTTAAGTCCAACTGCTGTCCTTT 14296

Gorilla CTTTGCTTATTTCCCCTACCAAATTTCTTCCTTTGATTTTAAGTCCAACTGCAGTCCTTT 14247

*********************** **************************** *******

Human CAAAGAATCAAAATCAGCAGCAATGCCGTTACTTAACAGCAAAAGTTACTCAAATTCTGA 14311

Chimpanzee CAAAGAATCAAAATCAGCAGCAATGCCATTACTTAACAGCAAAAGTTACTCAAATTCTGA 14356

Gorilla CAAAGGATCAAAATCAGCAGCAATGCTGTTACTTAACAGCAAAAGCTACTCAAATTCTGA 14307

***** ******************** ***************** **************

Human ATTAATAAGTGCCATTTGGAAATTTAGCTGCAGAAACTCAAAAAATGAAACTAGAGAAAC 14371

Chimpanzee ATTAATAAGTGCCATTTGGAAATTTAGCTGCAGAAACTCAAAAAATGAAACTAGAGAAAC 14416

Gorilla ATTAATAAGTGACATTTGGAAATTTAGCTGCAGAAACTCAAAAAATGAAACTAGAGAAAC 14367

*********** ************************************************

Human CCATAACATAATAGGGGATTTGACAAACTTCTCTAGGGAACTGATAAAATAACTAGACAA 14431

Chimpanzee CCATAACATAATAGGGGATTTGACAAACTTCTCTAGGGAACTGATAAAATAACTAGACAA 14476

Gorilla CCATAACATAATAGGGGATTTGACAAACTTCTCTAGGGNACTNNNNNNNNNNNNNNNNAA 14427

************************************** *** **

Human AGAATCAGTAGAAGGTTATAAATATTTAAAATTGGAATACATGATTAGTAAACTAGTCTT 14491

Chimpanzee AAAATCAGTAGAAGGTTATAAATATTTAAAATTGGAATACATGATTAGTAAACTAGTCTT 14536

Gorilla AAAATCAGTNNNNNNNNNNNNNNNNNNNNNNNNNNNNNNNNNGATTAGNNNNNNNNNNNN 14487

* ******* ******

Human GTAATCTATAAAACCTTACATCCACAGTGTAAAATACACATTCTTTAGAACATGTGAAAT 14551

Chimpanzee GTAATCTATAAAACCTTACATCCACAGTGTAAAATACACATTCTTTAGAACATGTGAAAT 14596

Gorilla NNNNNNNNNNNNNNNNNNNNNNNNNNNNNNNNNNNNNNNNNNNNNNNNNNNNNNNNNNNN 14547

Human AATAACTGTGGAAGGAAACTATCTGGGGGGCTCCAAAATCACTAATCTAAATGGAAAAGT 14611

Chimpanzee AATAACTGTGGAAGGAAAATATCTGGGGG-CTCCAAAATCACTAATCTAAATGGAAAAGT 14655

Gorilla NNNNNNNNNNNNNNNNNNNNNNNNNNNNNNNNNNNNNNNNNNNNNNNNNNNNNNNNNNNN 14607

Human CAAGCTGGGAACTGCTTAGGGCAAATCTGCCTCCCCTTCTATTCAAAGTCTTCCCTCTGT 14671

Chimpanzee CAAGCTGGGAACTGCTTAGGGCAAATCTGCCTCCCCTTCTATTCAAAGTCTTCCCTCTGT 14715

Gorilla NNNNNNNNNNNNNNNNNNNNNNNNNNCNNNNNNNNNNNCTATTCAAAGTCTTCCNNNNNN 14667

* ****************

Human TTACTGAGATAAATGCATACCTGATTTTCTCCTTTAAAGAGGCTAATCGGAAACTCAAAA 14731

Chimpanzee TTACTGAGATAAATGCATATCTGATTTTCTCCTTTAAAGAGGCTAATCGGAAACTCAAAA 14775

Gorilla NNNNNNNNNNNNNNNNNNNNNNNNNNNNNNNNNNNNNNNNNNNNNNNNNNNNNNNNNNNN 14727

Human GAATGCAACCATTTATCTCCTATCTACTTCCAACCTAGAAGACCCCTCCCGACTTGAACC 14791

Chimpanzee GAATGCAACCATTTATCACCTATCTACTTCCAACCTAGAAGACCCCTCCCGACTTGAACC 14835

Gorilla NNNNNNNNNNNNNNNNNNNCTATCTACATCCAACNNNNNNNNNNNNNNNNNNNNNNNNNN 14787

******** ******

Human CTTCTAGGCTGAACCAATGTTTATTTTACATATATGTTGATTGATGTCTCCCATCTCCTT 14851

Chimpanzee CTTCTAGGCTGAACCAATGTTTATCTTACATATATGTTGATTGATGTCTCCCATCTCCTT 14895

Gorilla NNNNNNNNNNNNNNNNNNNNNNNNNNNACATATATGTTGATTGATGTCTCCCATNNNNNN 14847

***************************

Human ACAGTGTGTAAAGCCAAACTGTACTCTGACCAACTTGGGCATATGTCCACGTGTCCATAT 14911

Chimpanzee ACAGTGTGTAAAACCAAACTGTACTCTGACCAACTTGGGCATATGTCCATGTGTCCATAT 14955

Gorilla NNNNNNNNNNAAACCANNNNNNNNNNNNNNNNNNNTGGGCNNNNGTCNNNNNNNNNNNAT 14907

** *** ***** *** **

Human AGAAGACCACGTGAACAGGCTTTGTGTGAGCAAACAAGGCTGTTTATTCAGTTGGGTGCA 14971

Chimpanzee AGAAGACCACATGAACAGGCTTCGTGTGAGCAA-CAAGGCTGTTTATTCAGTTGGGTGCA 15014

Gorilla AGAAGACNNNNNNNNNNNNNNNNNNNNNNNNNNNNNNNNNNNNNNNNNNNNTTGGGTGCA 14967

******* *********

Human AGTGGGCTGAGTCTGAAAAGAAAGTCAGAGAAGGGAGATAGGAGAGGAGAAGCTTTATAG 15031

Chimpanzee AGTGGGCTGAGTCTGAAAAGAAAGTCAGAGAAGGGAGATAGGAGAGGAGAAGCTTTATAG 15074

Gorilla AGTGGGCTGAGTCTGAAAAGAAAGTCAGAGAAGGGAGATNNNNNNNNNNNNNNNNNNNNN 15027

***************************************

Human GGCTTGGGTAGGCAGTGGAAAGTTAGAGTTAAAGGTGGTTGTCTGTTTTCAGTAGGGGAG 15091

Chimpanzee GGCTTGGGTAGGCAGTGGAAAGTTAGAGTTAAAGGTGGTTGTCTGTTTTCAGTAGGGGAG 15134

Gorilla NNNNNNNNNAGNCAGTGGAAAGTTAGAGTTAAAGGTGGCTGTCTGTTTTCAGTAGGGAAG 15087

** ************************** ****************** **

Human GGGGTCACATGGTTCCTGGTGGAGAGATGATGAGACTCATTGTCCAGGAGAAGAATGTCA 15151

Chimpanzee GGGGTCACATGGTTCCTGGTGGAGAGATGATGAGACTCATTGTCCAGGAGAAGAATGTCA 15194

Gorilla GGGGTCACATGGTTCCTGGTGGAGAGATGATGAGACTCATTGTCCAGGAGAAGAATGTCA 15147

************************************************************

Human CAGGATCAATTGATCAGTTTGGGCAAGGCAGAAACAAGTCATAATGGTGGAATGTCATAA 15211

Chimpanzee CAGGATCAATTGATCAGTTTGGGCAAGGCAGAAACAAGTCATAATGGTGGAATGTCATAA 15254

Gorilla CAGGATCAATTGATCAGTTTGGGCAAGNNAGAAANNNNNNNNNNNNNNNNNNNNNNNNNN 15207

*************************** *****

Human GTTGGTGGAATGTCATGAGGTCGGTCAATCAGTTAGGGCA-------------------- 15251

Chimpanzee GTTGGTGGAATGTCATGAGGTCGGTCAATCAGTTAGGGCATGTTAGAATCTTGCTCAAGA 15314

Gorilla NNNNNNNNNNNNNNNNNNNNNNNNNNNNNNNNNNNNGGCA-------------------- 15247

****

Human ------------------------------------------------------------

Chimpanzee TTCTACTTCGGGATGGGGCCACCGCCAGAGACCACCCGACACGGAGATCACAGCAAAGAG 15374

Gorilla ------------------------------------------------------------

Human ------------------------------------------------------------

Chimpanzee CACACTTTATTACTAGCGCGCTAGGGTCTCCAGCACAAGAGGCCGAGAGACCCCGAACGG 15434

Gorilla ------------------------------------------------------------

Human ------------------------------------------------------------

Chimpanzee CTGTTTTCACACAGTTTATATAGGCAAAAACCACACATCAAAACAAGGGAGGGGTTACAC 15494

Gorilla ------------------------------------------------------------

Human ------------------------------------------------------------

Chimpanzee AGCAATTAGGGGGAGGTTACAAATTCAAACAAGGGGAGGATACACACATTTGATTGGGTC 15554

Gorilla ------------------------------------------------------------

Human ------------------------------------------------------------

Chimpanzee ACATATTAAGGCGCGAGGACTGGGAGTAACTGATTGGTTCTTAATTATGGCCTGAGCCAG 15614

Gorilla ------------------------------------------------------------

Human ------------------------------------------------------------

Chimpanzee CTGTCTCACTCTGGTTGGTCAGGGGGCTTACAGGTGGCGGGCAGTATTTCCTGGAAATGT 15674

Gorilla ------------------------------------------------------------

Human ------------------------------------------------------------

Chimpanzee TTTTCTGTTTTTAGTTCCCGGAACAGGGGAGGGGGACCGGGGCTCCCTGCCCTGCCCTGG 15734

Gorilla ------------------------------------------------------------

Human ------------------------------------------------------------

Chimpanzee GCTTCTGGTGCCCTTGATAGCCATCGACACACCCTGACTCCTGACCATCAGGTGTTTTTC 15794

Gorilla ------------------------------------------------------------

Human ------------------------------------------------------------

Chimpanzee AAGTGGCCATCTGGTGTTTTTCTCTAAGAGACTAGCTGGTGTTTTTCCTTAAGAGACTGT 15854

Gorilla ------------------------------------------------------------

Human ------------------------------------------------------------

Chimpanzee CTAGCGTTTTTCTCTAGGGTCTCTCATTCCCTCCTCTTTTTGTGACTTAGAGGCTCAATC 15914

Gorilla ------------------------------------------------------------

Human ------------------------------------------------------------

Chimpanzee TTGAGTCTCTTCGTCAGTTTTGATGGCCTGGTACCGCTGAGCCAGGACCATAGCCTGCAC 15974

Gorilla ------------------------------------------------------------

Human ------------------------------------------------------------

Chimpanzee TATGTTTAACTTACTCTGAATAAAAGCCATCAAGCGGTTAAAAATGCAAGGTCCAAAAGT 16034

Gorilla ------------------------------------------------------------

Human ------------------------------------------------------------

Chimpanzee TAAAATGACTAGAAGCAAGATTAGGGGTCCTAAGATAGTGGAAATGAGAGTGCTAAACCA 16094

Gorilla ------------------------------------------------------------

Human ------------------------------------------------------------

Chimpanzee GGGGGATTGGTTATACCAAGATTCAAACCAGCTTTGTTGGGATTCTCTTTCTTTTTGTCT 16154

Gorilla ------------------------------------------------------------

Human ------------------------------------------------------------

Chimpanzee ATCATCTAGTCTGTTTCTAAGTTTTGCCATAGAGTCTTTAACTACTCCTGAATGATCAGC 16214

Gorilla ------------------------------------------------------------

Human ------------------------------------------------------------

Chimpanzee ATAAAAGCAACACTGCACAAAGGCCGCCCTCTTTCAGAAAAATTATGTCCAGTCCCTGTC 16274

Gorilla ------------------------------------------------------------

Human ------------------------------------------------------------

Chimpanzee GATTTTGGAGTACTACTTCAGACAGGGAGGTTAAAGATTCTTCAAGTTTGGTAATGGAGT 16334

Gorilla ------------------------------------------------------------

Human ------------------------------------------------------------

Chimpanzee GTTCTATGGCCCTGAGGTCTTCATCTACAGCTGCCCTGAGTTGTTGCATTTGATAACTGC 16394

Gorilla ------------------------------------------------------------

Human ------------------------------------------------------------

Chimpanzee CGGGCACTAATGCTGCGGTGCCTGTCCCGACCCCAGCCGCTACTCCTAATCCTAACATAA 16454

Gorilla ------------------------------------------------------------

Human ------------------------------------------------------------

Chimpanzee CAGCTAGGGTGAGGGAAACAGGCTCTCTCTTTATTCTAGCAGTTAGGTATTTTTGCTGAA 16514

Gorilla ------------------------------------------------------------

Human ------------------------------------------------------------

Chimpanzee ACTGGGACTCAAAAGACTCTCCAGTTTGATAATAAACTCGGGGCACAATTTGGACTAATA 16574

Gorilla ------------------------------------------------------------

Human ------------------------------------------------------------

Chimpanzee TACAATAGTCCTCACTGTCCTTGAAGACAGCTGCGGACACACAAGGGGTGAGCCCAGTCT 16634

Gorilla ------------------------------------------------------------

Human ------------------------------------------------------------

Chimpanzee TGCAAGCCCACCAGTCTGGCCCAGAGGGTATTAGATAGTGACTAGTTCTGGGCACTGCCA 16694

Gorilla ------------------------------------------------------------

Human ------------------------------------------------------------

Chimpanzee GGGTTCTATTACAAAGATGCTGATGACTAGGGGGCACCCGGCCTATACAAGTTCCCGACC 16754

Gorilla ------------------------------------------------------------

Human ------------------------------------------------------------

Chimpanzee CTGACACTTCTGCTAGGGTAAGTTTCTTATGCTGGTCCCATGCACACCCAGAATGGCTAG 16814

Gorilla ------------------------------------------------------------

Human ------------------------------------------------------------

Chimpanzee TGGAGTTAGTAAGATTACCAATAGAGGCAATACCTTCATAGTAAGGGGGGCTCGCGGCCA 16874

Gorilla ------------------------------------------------------------

Human ------------------------------------------------------------

Chimpanzee GGCAGAGCCAACAGGAGGTGGTGAATTCGGGCTTTGTCTGGTTCAGGGCGAGGTAGGCGC 16934

Gorilla ------------------------------------------------------------

Human ------------------------------------------------------------

Chimpanzee CTTTGATGAGGTTGAAGAGCCTATTGGTTACTTCCGGGTCGGGAGGCCGGCGGGTGGTCC 16994

Gorilla ------------------------------------------------------------

Human ------------------------------------------------------------

Chimpanzee CCGGGAGCGCCGACGGGGCCCTCGGGAGCGCCGAGGGGGAGGAGTTGGTCGACGAAGGGG 17054

Gorilla ------------------------------------------------------------

Human ------------------------------------------------------------

Chimpanzee ACAGGGAGGAGTTGGATGACAGAGTGGAGTTGGACGACGAAGGAGACGGGGTAGGGGCCG 17114

Gorilla ------------------------------------------------------------

Human ------------------------------------------------------------

Chimpanzee GGGAAGTGGTGGTAGTGGTGGTTGGGACTTTTTGCCGACTCCCTGACTGAGGAGGTGCTC 17174

Gorilla ------------------------------------------------------------

Human ------------------------------------------------------------

Chimpanzee TCCCTGTTAGAACCGGGTTTGGCCCTACCGGGACTGAGGCTGAGACCGGGTTAACTAATA 17234

Gorilla ------------------------------------------------------------

Human ------------------------------------------------------------

Chimpanzee GCCTAATTTGGATAGGGAGCCCAGATGCTGGAGTTTGGTATAAATTTAGGCCCCAAATTA 17294

Gorilla ------------------------------------------------------------

Human ------------------------------------------------------------

Chimpanzee GTCCAGTTGTCCAGCGGGGGTCAGTTTTTGCGGCCTCCTCAAATTTGACACGGATTAAGT 17354

Gorilla ------------------------------------------------------------

Human ------------------------------------------------------------

Chimpanzee TACAAGTGGCCGAATACCTGGTCCTAGTACAGGGCTGGACATAGGACATGGTCACATACC 17414

Gorilla ------------------------------------------------------------

Human ------------------------------------------------------------

Chimpanzee AGGGTTGGGTTTCCCATTTCCGTTCTCCATCATTAGTAGTTACACATGACCAACTTGCAC 17474

Gorilla ------------------------------------------------------------

Human ------------------------------------------------------------

Chimpanzee AGTATAGGGACATTATTTCACCACAGGTCTTTTTCATAGGCCCCGTCCGGAATCCAGGGC 17534

Gorilla ------------------------------------------------------------

Human ------------------------------------------------------------

Chimpanzee AGGCATAAAACCCGTTTCTACTTAGGGAGACTCTCCTTGCTCGTTTTCTCCACTCGCCAT 17594

Gorilla ------------------------------------------------------------

Human ------------------------------------------------------------

Chimpanzee CCTCCATTTCATCTATCATGGCTATTTTGTCAAGATTAAAGTACAAGTCAGGGAACCAGG 17654

Gorilla ------------------------------------------------------------

Human ------------------------------------------------------------

Chimpanzee TATTTAAAGGGGCTACATGTGAAGTCTCGTTGAGGACTTCATGGGTTTCAAAGTTAGTTA 17714

Gorilla ------------------------------------------------------------

Human ------------------------------------------------------------

Chimpanzee TTTGCCAAGTCAAGCGATAGGGCTGGTGGGGGTTACTGCTGCTGGTAACACAGGGAAGCA 17774

Gorilla ------------------------------------------------------------

Human ------------------------------------------------------------

Chimpanzee AAAGCAAAAGGGCTAACAGTAGGTGGGAGGTTAGGCACGAGAGAGTCTTATCTTGAGGGG 17834

Gorilla ------------------------------------------------------------

Human ------------------------------------------------------------

Chimpanzee GTCGTCGGAGCGGCGAAGTTTCCATTTCTCAGGCGATGCTGATCCTGACGCCTTCGGAGC 17894

Gorilla ------------------------------------------------------------

Human ------------------------------------------------------------

Chimpanzee AGCTTTCACGTGGGATGCGTGGATCCAGGCGGCGACCCCATCAACTTTCACAGCAGTGGG 17954

Gorilla ------------------------------------------------------------

Human ------------------------------------------------------------

Chimpanzee TGTGGTGAGAAGAACGATGTATGGTCCCTTCCACCGGGGTTCTAGTCCTTGGGAGCGGTG 18014

Gorilla ------------------------------------------------------------

Human ------------------------------------------------------------

Chimpanzee TCGTCTGACATAGACGGAGTCTCCCACTTGGAACGGGTGACTGGTGTGTGGGTGGCCCGG 18074

Gorilla ------------------------------------------------------------

Human ------------------------------------------------------------

Chimpanzee CTGGTAAAGTTCTGCCAAAGGAGCCCAAATTTGGGCTTGCACTGCCTGTAGTCCTTTTAG 18134

Gorilla ------------------------------------------------------------

Human ------------------------------------------------------------

Chimpanzee CCGAGCCTGCAAGTCAGTCTCAGGGTTAGAGGGGGAGAAAGAATCAAGCAAGGTTGACAA 18194

Gorilla ------------------------------------------------------------

Human ------------------------------------------------------------

Chimpanzee GGGAGGTGGTCCTCCGTAGAGGATTTCATAAGGGGTGAGCCCAAAGCGATTAGGCGTGTT 18254

Gorilla ------------------------------------------------------------

Human ------------------------------------------------------------

Chimpanzee TCGGGCCCTTAAGAGAGCTAAGGATAGGAGGCATCTCCAATCTTTTAAGCCAGTCTCTAA 18314

Gorilla ------------------------------------------------------------

Human ------------------------------------------------------------

Chimpanzee GGTCAATTTTGTCAAGGTCTCTTTAATAGTTCTATCCATCCGTTCTACCTGCCCTGAACT 18374

Gorilla ------------------------------------------------------------

Human ------------------------------------------------------------

Chimpanzee CTGGGGCCTGTATGCACAATGAAGTTTCCAATTAATCCCCAGTATCCTGGCAAGTCCCTG 18434

Gorilla ------------------------------------------------------------

Human ------------------------------------------------------------

Chimpanzee ACTTACCTGGGAGACGAAGGCTGGCCCATTGTCTGACCCGATTACCTTGGGAAGTCCAAA 18494

Gorilla ------------------------------------------------------------

Human ------------------------------------------------------------

Chimpanzee TCTGGGGAAAATTTCTTCTAATATCTTCTTGGCCACTATGTGGGCCGTTTCTTGACGGGT 18554

Gorilla ------------------------------------------------------------

Human ------------------------------------------------------------

Chimpanzee GGGGTAGGCTTCTACCCATCCTGAAAATGTATCTACAAACACTAATAAATACTTATATCC 18614

Gorilla ------------------------------------------------------------

Human ------------------------------------------------------------

Chimpanzee AGCATGGTGAGGCTTTATTTCAGTAAAGTCTACTTCCCAATAGGTCCCAGGGCGGTTGCC 18674

Gorilla ------------------------------------------------------------

Human ------------------------------------------------------------

Chimpanzee CCGTGCCCTTGTCCCCGCTGGGACTCGCGTAGCCCCGGCGTTTACTTGTTGGCAGGCCTT 18734

Gorilla ------------------------------------------------------------

Human ------------------------------------------------------------

Chimpanzee GCAAGCGAGCGTTACTTGCTCCAGGAGGGCGCCGACCCTGGGGATTAGGAAGTCAGTCTT 18794

Gorilla ------------------------------------------------------------

Human ------------------------------------------------------------

Chimpanzee TTCAATGAGCAGTTTTAGCTTCTTACTACTTAAATGTGTCCAAGAGTGCATCTGCTGCAG 18854

Gorilla ------------------------------------------------------------

Human ------------------------------------------------------------

Chimpanzee CATGGCCTTGGCCTCTTTTTGTGGGAGGACTGTCTTCCCGTCTTTTCCCCAATTTTTAGT 18914

Gorilla ------------------------------------------------------------

Human ------------------------------------------------------------

Chimpanzee CTCTGGGTTTTCTGTGGCTCCTAAGGCTACTGCTTCTTCCCGGTCCTCTGGTGTATAGAT 18974

Gorilla ------------------------------------------------------------

Human ------------------------------------------------------------

Chimpanzee ATAGCTTGTGGAAGGGGGTACCTGGTCAGCCTCTTTGGTTCTTGAAACTAAAGTCAATGT 19034

Gorilla ------------------------------------------------------------

Human ------------------------------------------------------------

Chimpanzee CTCTACTCTAGCGGCCTGCTTGGCCGCCTGGTCAGCTTGCCTGTTACCCTGGGCGACAGG 19094

Gorilla ------------------------------------------------------------

Human ------------------------------------------------------------

Chimpanzee GTCTTGCCCTTTTTGATGTCCAGGACAATGGATTATGGCCACCTTCTTAGGGAGGAAAAG 19154

Gorilla ------------------------------------------------------------

Human ------------------------------------------------------------

Chimpanzee GGCCTTTAATAAGGCGATTATTTCGGCCTTATTCTTGATTTCCTTTCCTTCTGATGTCAG 19214

Gorilla ------------------------------------------------------------

Human ------------------------------------------------------------

Chimpanzee GAGACCTCGTCTCTCGTAAATGCTCCCATGGGTGTGGGCTGTGGCAAAGGCATACCGACT 19274

Gorilla ------------------------------------------------------------

Human ------------------------------------------------------------

Chimpanzee GTCTGTGTAGATGTTAGCCTTTTTCCCCTGTGATAGCTCTAAGGCCTTTGTCAAAGCTAT 19334

Gorilla ------------------------------------------------------------

Human ------------------------------------------------------------

Chimpanzee CAGTTCAGCCTTTTGAGCAGACGTGCCGGGAGGCAGTGCTTGCGCCCATACTGTGGCATG 19394

Gorilla ------------------------------------------------------------

Human ------------------------------------------------------------

Chimpanzee TCCATCCACTACCACCGCCCCCGCCCTCCGGGTACCTGCGTCCATGAAGCTGCTACCGTC 19454

Gorilla ------------------------------------------------------------

Human ------------------------------------------------------------

Chimpanzee TGTGTACCAAGTGTGGTCCGCGTCTGGGAGTTCGTAGTCCTGGAGGTCTTCTCGAGTTCC 19514

Gorilla ------------------------------------------------------------

Human ------------------------------------------------------------

Chimpanzee GTGGGTCTCCGCTAGCACTTGCCGGCAGTCGTGGGAGCTCAGCAGGACCTCCGGTACAGG 19574

Gorilla ------------------------------------------------------------

Human ------------------------------------------------------------

Chimpanzee TAGCAAGGTGGCAGGATTCAGAGTGACTGGAGGGCCAAAGCTGACACGGTCCATGTCTAG 19634

Gorilla ------------------------------------------------------------

Human ------------------------------------------------------------

Chimpanzee TAGGAGGGCTTGGTAGTGGGTTAGGCGGGCGTTGGTGATCCAACGGTCCGGGGGCTGCCG 19694

Gorilla ------------------------------------------------------------

Human ------------------------------------------------------------

Chimpanzee CACTATGGCCTCCAAGGCATGCGGGGTAATGACAGTCAGTGGTTGCCCAAGGGTTAACTT 19754

Gorilla ------------------------------------------------------------

Human ------------------------------------------------------------

Chimpanzee AGCAGAGTCTTTGACTAGCATAGCGGTGGCTGCCATGATGCGGAGGCAAGGAGGCCATCC 19814

Gorilla ------------------------------------------------------------

Human ------------------------------------------------------------

Chimpanzee AGCCGCCACAGGGTCCAGTTTCTTAGATAAGTAGGCTACCGGTCTCTTCCAGGGCCCCAG 19874

Gorilla ------------------------------------------------------------

Human ------------------------------------------------------------

Chimpanzee TTTTTGAGTCAAGACTCCTTTGGCTATCCCCCGCCTCTCGTCTACATAGAGGGTGAAAGG 19934

Gorilla ------------------------------------------------------------

Human ------------------------------------------------------------

Chimpanzee CTTGGATGTGTCAGGTAGCCCGAGGGCTGGGGCAGAGAGGAGCGCCTTCTTTAAAGCCTC 19994

Gorilla ------------------------------------------------------------

Human ------------------------------------------------------------

Chimpanzee GAAGGCCTGCTGGTGTTCTTCCGACCAGGTAAAGGGGTTGCTCCCTTTAGTGAGGGCATA 20054

Gorilla ------------------------------------------------------------

Human ------------------------------------------------------------

Chimpanzee AAGAGGGGCTGCCAACTCAGCAAAACCAGGTATCCACAGGCGGCAGAACCCAGCAGTCCC 20114

Gorilla ------------------------------------------------------------

Human ------------------------------------------------------------

Chimpanzee TAGGAACTCACGCACCTCCTTGGGACTCCGGGGCAGTGGAATGCTGGCTACAGTCTCTAT 20174

Gorilla ------------------------------------------------------------

Human ------------------------------------------------------------

Chimpanzee TCGCCCAGGGGTGAGCCACCTCTTTCCTTCACTTAGGATGTACCCCAGGTAGGTTACCTT 20234

Gorilla ------------------------------------------------------------

Human ------------------------------------------------------------

Chimpanzee AGTCTGGCAGATTTGTGCTTTCTTGGCAGATGCTCGGTATCCTTTCTCTCCGAGCTCCTG 20294

Gorilla ------------------------------------------------------------

Human ------------------------------------------------------------

Chimpanzee GAGCAGGTGTCTGGTGCCCTGCAGGCAGGCTTCCTTGGTGGGGGCGGCCAGGAGGAGGTC 20354

Gorilla ------------------------------------------------------------

Human ------------------------------------------------------------

Chimpanzee GTCTACATACTGGAGCAGAGTTAAATCTGGGTGCTGGGTGCGAAAATTAGTCAAGTCTCG 20414

Gorilla ------------------------------------------------------------

Human ------------------------------------------------------------

Chimpanzee GTGAAGAGCCTCATCAAAGAGGGTAGGAGAGTTCTTGAACCCTTGGGGAAGCCGAGTCCA 20474

Gorilla ------------------------------------------------------------

Human ------------------------------------------------------------

Chimpanzee AGTTAATTGGCCCGAGATCCCCTTCTCTGGGTCTCTCCATTCAAAGGCAAAGAGTCCCTG 20534

Gorilla ------------------------------------------------------------

Human ------------------------------------------------------------

Chimpanzee GCTTTGGGGAGCCAGGGGCAAGCAAAAGAATGCATCTTTCAGGTCTAGCACCGTATACCA 20594

Gorilla ------------------------------------------------------------

Human ------------------------------------------------------------

Chimpanzee GTTGTGATCTGGCCTTAAGGTACGGAGCAGGTTGTAAGGGTTGGGGACTGTGGAATGTAT 20654

Gorilla ------------------------------------------------------------

Human ------------------------------------------------------------

Chimpanzee GTCCATGGGTCTCTTATTAATTTCTCTCAAGTCCTGGACGGGCCTATAATCTTGGGTACC 20714

Gorilla ------------------------------------------------------------

Human ------------------------------------------------------------

Chimpanzee AGGTTTCTTTACCGGGAGGAGCGGCGTGTTCCAAGGTGAGCGGCATGGCCGCAAGACTCC 20774

Gorilla ------------------------------------------------------------

Human ------------------------------------------------------------

Chimpanzee TAATTCCAGAAATTTATCAATATGCTGCCGGATGCCCATACGGGCTTCTTGGCTCATGGG 20834

Gorilla ------------------------------------------------------------

Human ------------------------------------------------------------

Chimpanzee GTATTGCTTAATGGACACTGGCACTGCAGTGGGTTTGAGGTCAACTATAATCGGAGCTTG 20894

Gorilla ------------------------------------------------------------

Human ------------------------------------------------------------

Chimpanzee AAATTTAGCCAGTCCAAGTCCCCCTGTCTCTGCCCAAGCCTGGGGAAATTCTTGCAGCCA 20954

Gorilla ------------------------------------------------------------

Human ------------------------------------------------------------

Chimpanzee ATTATCAGAAGGGCTGGTGGGGATAGGAGTCTCAAAAAGCCGGTACTCATCTTGCAAGGA 21014

Gorilla ------------------------------------------------------------

Human ------------------------------------------------------------

Chimpanzee AACGGTCAGAATTTGGATAGGCTGACCATCCTCACCCAGTACCTGGGCCCCTCTCTCTGA 21074

Gorilla ------------------------------------------------------------

Human ------------------------------------------------------------

Chimpanzee GAAATGTATCTGGGCTCCGAGCTTGGTCAACAGATCCTGCCCCAGAAGGGGATATGGGCA 21134

Gorilla ------------------------------------------------------------

Human ------------------------------------------------------------

Chimpanzee TTCAGGTACTACTAAGAAAGAATGAGTCACCATACCTTTTCCAAGGTTTACTGTTCGGTG 21194

Gorilla ------------------------------------------------------------

Human ------------------------------------------------------------

Chimpanzee GGTCATCCACTTGTGCAGCTTTCCTCCTGTTGCTCCTTGGACCCAAGAGGTGCGGGATGA 21254

Gorilla ------------------------------------------------------------

Human ------------------------------------------------------------

Chimpanzee AAGAGGCCCGGTGTCTACTAGAAAGGTGGTGGGGCGCCCCCCTGTAGAAAGGGTTAGCCG 21314

Gorilla ------------------------------------------------------------

Human ------------------------------------------------------------

Chimpanzee GGGCTCGGTGGGGGGGGGGGGGCTTTGGAGCCCTGACGCCCCTAATCACTGTCCTCTCCC 21374

Gorilla ------------------------------------------------------------

Human ------------------------------------------------------------

Chimpanzee AGGGTCAGGACAGGAGTGGGCTTCTTCCGGTCCTTAGGACGTTTAGGACATTCCTTGACC 21434

Gorilla ------------------------------------------------------------

Human ------------------------------------------------------------

Chimpanzee CAATGTCCTCGTTCCTTGCAATAGGCACATTGGTCTTTATCCACCTTCGGGCGCCTTCGT 21494

Gorilla ------------------------------------------------------------

Human ------------------------------------------------------------

Chimpanzee TCTCCCCCCTCTCTCCCTGGCCCTGGCCCTTTCCCTGTCACTACTGCTGCCAGGATTTTA 21554

Gorilla ------------------------------------------------------------

Human ------------------------------------------------------------

Chimpanzee GTCAAATGCCTGTCTCTCTTACGATCTCGTCGATCTTCCCGCTCCTCCTGTTCCTTTGCT 21614

Gorilla ------------------------------------------------------------

Human ------------------------------------------------------------

Chimpanzee AGCCTAGCTTCTTTCTCCTCAATAGTCTCTCTTTTATTATAAACTTTTTCTGCCTCTCTA 21674

Gorilla ------------------------------------------------------------

Human ------------------------------------------------------------

Chimpanzee ACTAGTTCTTGCAGCCCATAGGTCTGGATCCCGTCCAATCTTTGGAGCTTACTCTTTATG 21734

Gorilla ------------------------------------------------------------

Human ------------------------------------------------------------

Chimpanzee TCTAGTGCTGCCTGATCTATGAATGACATTGCCACGGTGGCCTTGTGCTCTGGAGCCTCG 21794

Gorilla ------------------------------------------------------------

Human ------------------------------------------------------------

Chimpanzee GGGTTAAATGGAGTGTACATCCGGAACCCCTCTAAGAGCCTTTCCATGAAGGTCGCCATG 21854

Gorilla ------------------------------------------------------------

Human ------------------------------------------------------------

Chimpanzee CTTTCATTCTTTCCCTGAGTAATAGTTCTTACCTTAGCCAAATTGGTGGGGCGCTTTCCG 21914

Gorilla ------------------------------------------------------------

Human ------------------------------------------------------------

Chimpanzee GCCCCTTTGAGACCTGCCAACAGAGCCTGGCGATAGATTCGGAGACTCTCCCTACCTGTT 21974

Gorilla ------------------------------------------------------------

Human ------------------------------------------------------------

Chimpanzee GCAGTTTCATAGTCCCAGTCCGGGCGGGTGAGGGGAAATCCCTCATCTATCTCATTAGGG 22034

Gorilla ------------------------------------------------------------

Human ------------------------------------------------------------

Chimpanzee AGTTGGGTCGGGAATCCTCCTGGCCCCAGCACATTTTTGCGGGCCTCGAGGAGGACTCAC 22094

Gorilla ------------------------------------------------------------

Human ------------------------------------------------------------

Chimpanzee TGTCTCTCTTCTGTGGTCAGAAGAACCTGTAAGAGCTGTTGGCAGTCATCCCAGGTGGGT 22154

Gorilla ------------------------------------------------------------

Human ------------------------------------------------------------

Chimpanzee TGGTGAGTGAGGAGAATGGACTCTATCAGAGAGGTCAGAGCCTGCGGGTCTTGGGAGAAA 22214

Gorilla ------------------------------------------------------------

Human ------------------------------------------------------------

Chimpanzee GGGGGGTTATGGGTCTTCCAATTGTAGAGATCTGAGGCCGAAAAGGGCCAATACTGGACC 22274

Gorilla ------------------------------------------------------------

Human ------------------------------------------------------------

Chimpanzee GTGCGGTTCACGGTGCGGAGGCGAAAGAGAGAAGACTGCCAGGTGGGCTGGTCACCGGAG 22334

Gorilla ------------------------------------------------------------

Human ------------------------------------------------------------

Chimpanzee TCCTTGGCCCGCCGTAAGCGGAGGCGGGGTGTCTGAGGCGGGGTCTGAGGGGTGAGTTCA 22394

Gorilla ------------------------------------------------------------

Human ------------------------------------------------------------

Chimpanzee GGTGGAGCCGGAGAAAGGGATGGGTACAGAGAGGGGGTGGAAGAAGAGGAGGTCGGCGCC 22454

Gorilla ------------------------------------------------------------

Human ------------------------------------------------------------

Chimpanzee GAAGAAGGGGAGGTTGGAGAAAGAGTAGGAGCCGAAGGGGTGGAAGAAGGGGCAGGGGAC 22514

Gorilla ------------------------------------------------------------

Human ------------------------------------------------------------

Chimpanzee AACATGGGGGCTGAGGGTGTGGAGTAGGGAGGGGGGTTGAGGAGAGGGTTGTGAGGCGGA 22574

Gorilla ------------------------------------------------------------

Human ------------------------------------------------------------

Chimpanzee GGAGGGAGAGGGTCTAGAAGGAGGAGGTCCCTCTGACCCTCATCCGGGAGAACAGGTTTC 22634

Gorilla ------------------------------------------------------------

Human ------------------------------------------------------------

Chimpanzee AGGGGGACCGAGGTCAGGTTTCGGGGGGCTTTCAAGGCGAGGAAGGTCGACTGGGAAGGA 22694

Gorilla ------------------------------------------------------------

Human ------------------------------------------------------------

Chimpanzee GGAGCTGAGGAGATGAAGGGCTTCACCCAAGAGGGTGGATCCCGAACCAGGTCTACGGAG 22754

Gorilla ------------------------------------------------------------

Human ------------------------------------------------------------

Chimpanzee GTGATGATATAGGCCACCTGGTCAGGGCGGCCTCGTGGCCCTGGATCCATCACTTTTGCC 22814

Gorilla ------------------------------------------------------------

Human ------------------------------------------------------------

Chimpanzee TTAACCTGTAAGATAATTGAGAGGTCAAAAGTTCCATCCTGGGGCCACCCTACACCGAGG 22874

Gorilla ------------------------------------------------------------

Human ------------------------------------------------------------

Chimpanzee ATGGGCCATTCGGAAGAGCAAAAGGTTTTCCATCGCCCTTTGCGAATTTCAACAGGAAGA 22934

Gorilla ------------------------------------------------------------

Human ------------------------------------------------------------

Chimpanzee TTGTGGGCTCGAGCCTTTACGTCAGGAAAGTGAGTCAGAGTCAGAGAGAGAGGAGTCGTC 22994

Gorilla ------------------------------------------------------------

Human ------------------------------------------------------------

Chimpanzee AGTGTCTGTCCCATAGTCAAGCCCAAGTCAATTTCTAACACACAGACAAACAGGACAACA 23054

Gorilla ------------------------------------------------------------

Human ------------------------------------------------------------

Chimpanzee GCAAAAACAGTGACTGCACACACACACAGAAGCCGCGCGGCGAGAGAACGGCGCCACAAT 23114

Gorilla ------------------------------------------------------------

Human ------------------------------------------------------------

Chimpanzee TCCACGAAGGATTCAGATGGCCGGGAGAACCGGCCTACAGAGGTGAGGAGACTGACGGTA 23174

Gorilla ------------------------------------------------------------

Human ------------------------------------------------------------

Chimpanzee TCGTCAGTTCTCCTCCAGACAACCGCCAGAGCGGTTCGGATGGCCGGGAGAACCGGCCTA 23234

Gorilla ------------------------------------------------------------

Human ------------------------------------------------------------

Chimpanzee CAGATGGCAGGAAAAACCCGCCTACAGAGGTGAGGAGACCGACGGGATCGTCAGTTTTCC 23294

Gorilla ------------------------------------------------------------

Human ------------------------------------------------------------

Chimpanzee TCCAGACTACCACCAGGGCGTCCCCCGGGGCGTAAGTGGGAAGGTGCCGGGCATGTCTAC 23354

Gorilla ------------------------------------------------------------

Human ------------------------------------------------------------

Chimpanzee CCCGCTTCCACACTTATACAGACTGAGCACCAGTACTCTTCAGATGGCAGGAAGAACCCG 23414

Gorilla ------------------------------------------------------------

Human ------------------------------------------------------------

Chimpanzee CCTGCAGATTTAGCCGGCTACAAAAATACACAAGTCAAGCAGACAAACAGAACAAAACGA 23474

Gorilla ------------------------------------------------------------

Human ------------------------------------------------------------

Chimpanzee AAGTACCTGCGCAGGAGGTTTGGACGTCCGTCGTTGGCCCGGGCCGAGCGGGTCTTCGCC 23534

Gorilla ------------------------------------------------------------

Human ------------------------------------------------------------

Chimpanzee ATGCGGGTCGTGGGGGTGTCGTCAGGCGGTGACCAATCCCGGACGAGCCCCGAAATGTTA 23594

Gorilla ------------------------------------------------------------

Human ------------------------------------------------------------

Chimpanzee GAATCTTGCTCAAGATTCTACTTCGGGATGGGGCCGCCGCCAGAGACCACCCGACACGGA 23654

Gorilla ------------------------------------------------------------

Human ------------------------------------------------------------

Chimpanzee GATCACAGCAAAGAGCACACTTTATTACTAGCGCGCTAGGGTCTCCAGCACAAGAGGCCG 23714

Gorilla ------------------------------------------------------------

Human ------------------------------------------------------------

Chimpanzee AGAGACCCCGAACGGCTGTTTTCACACAGTTTATATAGGCAAAAACCACACATCAAAACA 23774

Gorilla ------------------------------------------------------------

Human ------------------------------------------------------------

Chimpanzee AGGGAGGGGTTACACAGCAATTAGCGGGAGGTTACAAATTCAAACAAGGGGAGGATACAC 23834

Gorilla ------------------------------------------------------------

Human ------------------------------------------------------------

Chimpanzee ACATTTGATTGGGTCACATATTAAGGCGCGAGGATTGGGAGTAACTGATTGGTTCTTAAT 23894

Gorilla ------------------------------------------------------------

Human ------------------------------------------------------------

Chimpanzee TATGGCCTGAGCCAGCTGTCTCACTCTGGTTGGTCAGGGGGCTTACAGGTGGCGGGCAGT 23954

Gorilla ------------------------------------------------------------

Human ------------------------------------------------------------

Chimpanzee ATTTCCTGGAAATGTTTTTCTGTTTTTAGTTCCCGGAACAGGGGAGGGGGACCGGGGCTC 24014

Gorilla ------------------------------------------------------------

Human ------------------------------------------------------------

Chimpanzee CCTGCCCTGCCCTGGGCTTCTGGTGCCCTTGATAGCCATTGACACACCCTGACTCCTGAC 24074

Gorilla ------------------------------------------------------------

Human ------------------------------------------------------------

Chimpanzee CATCAGGTGTTTTTCAAGTGGCCATCTGGTGTTTTTCTCTAAGAGACTAGCTGGTGTTTT 24134

Gorilla ------------------------------------------------------------

Human ---------------------------------------------GGAGCTGGCTGTTTC 15266

Chimpanzee TCCTTAAGAGACTGTCTAGCGTTTTTCTCTAGGGTCTCTCAGGCAGGAGCTGGCTGTTTC 24194

Gorilla ---------------------------------------------GGAGCTGGCTCTTTC 15262

********** ****

Human ACTTCTCTTGTGGTTTTTCAGCTGCTCCAGACTTCTTGGCTCCTGCAGGCCATCTAGATG 15326

Chimpanzee ACCTCTCTTGTGGTTTTTCAGCTGCTCCAGACTTCTTGGCTCCTGCAGGCCATCTAGATG 24254

Gorilla ACTTCTTTTGTGGTTTTTCAGCTGCTCCAGACTTCTTGGCTCCTGCAGGCCATNNNNNNN 15322

** *** **********************************************

Human TATATGTGAAGGTCACAGAGGCTATAATGGCTGAGCTTTGGCTCAGAGGCCTGACGAGAC 15386

Chimpanzee TATATGTGAAGGTCACAGAGGCTATAATGGCTGAGCTTTGGCTCAGAGACCTGACAAGAC 24314

Gorilla NNNNNNNGAAGGTCACAGAGGCTATAATGGCTGAGCTTTGGCTCAGAGGCCTGACAAGAC 15382

***************************************** ****** ****

Human CACCAAACAGACCACATCCTGGGCTGTAAAGCAAAGTTCAATAACTTCAACAACTTTTTT 15446

Chimpanzee CGCCAAACAGACCACATCCTGGGCTGTAAAGCAAAGTTCAATAACTTCAACAACTCTTTT 24374

Gorilla TGNNNNNNNNNNNNNNNNNNNNNNNNNNNNGCNNNNTTCAATAACTTCAACAACTTTTTT 15442

** ******************* ****

Human TCTCTGAACACAAAGTTTCACCACTAGAAAAAAACGCTAAAATGTTTGAAATTAAGAAAG 15506

Chimpanzee TCTCTGAACACAAAGTTTCACCACTAGAAAAAA-CCCTAAAATGTTTGAAATTAAGAAAG 24433

Gorilla TCTCTGAACACAAAGTTTCACCANNNNNNNNNNNNNNNNNNNNNNNNNNNNNNNNNNNNN 15502

***********************

Human AAAGTTCTAAGTAGTTCCTTTACCAACTGAAAATAAATATGGAAATTAGAAAATACTCAG 15566

Chimpanzee AACGTTCTAAGTAGTTCCTTTACCAACTGAAAATAAAAATGGAAATTAGAAAATACTCAA 24493

Gorilla NNNNNNCTAAGTAGTTCCTTTACCAACTGNNNNNNNNNNNNNNNNNNNNNNNNNNNNNNN 15562

***********************

Human AAATAACAAGGAAACTTTCATATGACACCTGGCAATTTTTTGGAGGCCATGAAAAATGGT 15626

Chimpanzee AAATAACAAGGAAACTTTCATATGACACCTGGCAATTTTTTGGAGGCCATGAAAAATGGT 24553

Gorilla NNNNNNNNNNNNNNNNNNNNNNNNNNNNCTGGCAATTTTTTGGAGGCCACNAAAAATGGT 15622

********************* *********

Human GCTAAGCAAATTTAATTATTTTAAGTAAATTTGGCCCTACAACAACATAAAGATTAGGGA 15686

Chimpanzee GCTAAGCAAATTTAATTATTTTAAGTAAATGTGGCCCTACAACAACATAAAGATTAGGGA 24613

Gorilla GCTAAGCAAATTTAATTATTTTAAGTAAATTTGGCNNNNNNACANNNNNNNNNNNNNNNN 15682

****************************** **** ***

Human CACGGATCCCCACTCACAATCAACAGTCTGCATTTAATTTCTGACTGCCCCGGAACTCTA 15746

Chimpanzee CACGGATCCCCACTGACAATCAACAGTCTGCATTTAATTTCTGACTGCCCTGGAACTCTA 24673

Gorilla NNNNNNNNNNNNNNNNNNNNNNNNNNNNNNNNNNNNNNNNNNNNNNNNNNNNNNNNNNNN 15742

Human CTCTTAATAGTGTACTTTTGACCAGAAGCCTTATTGATAATACAAACAGCCAATAAACAC 15806

Chimpanzee CTCTTAATAGCGTACTTTTGACCAGAAGCCTTATTGATAATACAAACAGCCAATAAACAC 24733

Gorilla NNNNNNNNNNNNNNNNNNNNNNNNNNNNNNNNNNNNNNNNNNNNNNNNNNNNNNNNNNNN 15802

Human ATATTTTGTATGATCTATGCATTACATACTGCTATACTGTATTCTTACAATAAAGTAAGC 15866

Chimpanzee ATATTTTGTATGATCTATGCATTACATACTGCTATACTGTATTCTTACGATAAAGTAAGC 24793

Gorilla NNNNNNNNNNNNNNNNNNNNNNNNNNNNNNNNNNNNNNNNNNNNNNNNNNNNNNNNNAGC 15862

***

Human TACAGAAAAGAAAATGGTATTAAGAAAATCATAAGGAAGAGAAAATATATTTACTATTTA 15926

Chimpanzee TACAGAACAGAAAATGGTATTAAGAAAATCGTAAGGAAGAGAAAATATATTTAGTATTTA 24853

Gorilla TACANAAAAGAAANNNNNNNNNNNNNNNNNNNNNNNNNNNNNNNNNNNNNNNNNNNNNNN 15922

**** ** *****

Human TTAAGTGGAAGTGGATCATCATAAAGGTCTTCATCTTCATTGTTTTCACATTGAGTAGGC 15986

Chimpanzee TTAAGTGGAAGTGGATCATCATAAAGGTCTTCATCTTCATTGTTTTCACATTGAGTAGGC 24913

Gorilla NNNNNNNNNNNNNNATCATNNNNNNNNTCTTCATCTTCATTGTTTTCACATTGAATAGGC 15982

***** *************************** *****

Human TGAGGAAGAGGAAGATTAGGAAGAGAAAAAGGAGAAGAGGTTGTTCTTGCTGTCTCAGGG 16046

Chimpanzee TGAGGAAGAGGAAGATTAGGAAGAGAAAAAGGAGAAGAGGTTGTTCTTGCTGTCTCAGGG 24973

Gorilla TGAGGAAGNNNNNGATTAGGAANAGAAAAAGGAGAAGAGGTTGTTCTTGCTGTCTCAGGN 16042

******** ********* ************************************

Human GTGTCAGAAGTAGAAGAAAATTCATGCATGTGTGAACTCATGTGGTTCAAACCGATGCTT 16106

Chimpanzee GTGTCAGAAGTAGAAGAAAATTCATGCATGTGTGAACTCATGTGGTTCAAACCGATGCTT 25033

Gorilla GTGTNNNNNNNNNNNNNNNNNNNNNNNNNNNNNNNNNNNNNNNNNNNNNNNNNNNNNNNN 16102

****

Human TTCAAGGGTGAACTTAGTCATGTAAAATTGAGTTTGGAAAATAAGTTTGTAGCAGAGGGA 16166

Chimpanzee TTCAAGGGTGAACTTAGTCATGTAAAATTGAGTTTGGAAAATAAGTTTGTAGCAGAGGGA 25093

Gorilla NNNNNNNNNNNNNNNNNNNNNNNNNNNNNNNNNNNNNNNNNNNNNNNNNNNNNNNNNNNN 16162

Human TTAAAAAATGAGTCTTTCTGATATGTAGATGACATTCAATGGCATCCCACTGTATGAAAG 16226

Chimpanzee TTAAAAAATGAGTCTTTCTGATATGTAGATGACATTCAATGGCATCCCACTGTATGAAAG 25153

Gorilla NNNNNNNNNNNNNNNNNNTGANACGTNNNNNNNNNNNNNNNGCATCCCACTGTATGAAAG 16222

*** * ** *******************

Human GAGTGAATGTCAGTAAAAGACTGAGTCAGAATTTAACAATATCAGGGAAAAGCAAAGGAA 16286

Chimpanzee GAGTGAATGTCAGTAAAAGACTGAGTCAGAATTTAACAATATCAGGGAAAAGCAAAGGAA 25213

Gorilla GAGTGAATGTCAGTAAANNNCNNNNNNNNNNNTTAACAATATCAGGGAAAAGCAAAGGAA 16282

***************** * ****************************

Human ATGGCAAACTGAGAATTAGCAATGGGTATAGTAGGAAAAGAAACACACACACAAGCAAGG 16346

Chimpanzee ATGGCAAACTGAGAATTAGCAATGGGTATAGTAGGAAAAGAAACACACACACAAGCAAGG 25273

Gorilla ATGGCAAACTGAGAATTAGCAATGTGTATAGTAGGAAAANNNNNNCACANNNNNGCANNN 16342

************************ ************** **** ***

Human CAGTGTATTGTCCTAAAGACCAGTATGCAGAAGTGACAAGTCATTTCAAGATAGAGATTG 16406

Chimpanzee CAGTGCATTGTCCTAAAGACCAGTATGCAGAAGTGACAAGTCATTTCAAGATAGAGACTG 25333

Gorilla NNNNNNNNNNNNNNNNNNNNNNNNNNNNNNNNNNNNNNNNNNNNNNNNNNNNAGAGACTG 16402

***** **

Human TGAATTGAAAATAGAATTCAACACTGTGACGTTATCAGTGACCTTTACATAACTTCTCAG 16466

Chimpanzee TGAATTGAAAATAGAATTCAACACTCTGACGTTATCAGTGACCTTTACATAACTTCTCAG 25393

Gorilla TGAATTGAAAATAGAATTCAACACTATGACGTTATCAGTGACCTTTACATAACTTCTCAG 16462

************************* **********************************

Human AAAGGTAGTAGAAAATTTTGAATGGACATTATTAAAAGAGAATGGCAGGTGGAAAAAACA 16526

Chimpanzee AAAGGTAGTAGAAAATTTTGAATGGACATTATTAAAAGAGAATGGCAGGTGGAAAAAACA 25453

Gorilla AAAGGTNNNNNNNNNNNNNNNNNNNNNNNNNNNNNNNNNNNNNNNNNNNNNNNNNNNNNN 16522

******

Human GGGGCATGGTGAGGAGACACAGCTATCATGGAGGAATTTTGCTGATATAGGAGCACAAAG 16586

Chimpanzee GGGGCATGGTGAGGAGACACAGCTATCATGGAGGAATTTTGCTGATATAGGAGCACAAAG 25513

Gorilla NNNNNNNNNNNNNNNNNNNNNNNNNNNNNNNNNNNNNNNNNNNNNNNNNNNNNNNNNNNN 16582

Human AAAGGAGTGGGGTTGGTGCCACTATCTGACTGATAAAATGAGATTGTCTTAGCTCACTCA 16646

Chimpanzee AAAGGAGTGGGGTTGGTGCCACTATCTGACTGATAAAATGAGATTGTCTTAGCTCACTCA 25573

Gorilla NNNNNNNNNNNNNNNNNNNNNNNNNNNNNNNNNNNNNNNNNNNNTGTCTTAACTCGCTCA 16642

******* *** ****

Human GGCTGTTTTAACAGAATACATTAGACTGGATAATTTGGAAACAACAGAAATTTATTTCTT 16706

Chimpanzee GGCTGTTTTAACAGAATACGTTAGACTGGATAATTTGGAAACAACAGAAATTTATTTCTT 25633

Gorilla GGCTGTTTTAACAGAATACATNNNNNNNNNNAATTTGGAAANNNNNNNNNNNNNNNNNNN 16702

******************* * **********

Human ACCGTTCTGGGGCCTGAGATGTCCAAGATGAAGACATGGCAGATTCAGTGACAACTTGCT 16766

Chimpanzee ACAGTTCTGGGGCCTGAGACGTCCAAGATGAAGACATGGCAGATTCAGTGACAACTTGCT 25693

Gorilla NNNNNNNNNNNNNNNNNNNNNNNNNNNNNNNNNNNNNNNNNNNNTCAGTGACAACTTGCT 16762

****************

Human TTCTGCTCCTCTTACTGTGTCCTCACTTGGTGGAAGAGGCAAGGGAGCTCTATGAGGTGC 16826

Chimpanzee TTCTGCCCCTCTTACTGTGTCCTCTCTTGGTGGAAGAGGCAAGGGAGCTCTATGAGGTGC 25753

Gorilla TTCTGCCCCTCTTACTGTGTCCTCACTTGGTGGAAGAGGCAAGGGAGCTCTATGAGGTGC 16822

****** ***************** ***********************************

Human CTTCTATAAGGATACTAATCCCATTCATGAGGATTCACCCTCATGATCTCATCACCACAT 16886

Chimpanzee CTTCTATAAGGATACTAATCCCATTCATGAGGATTCACCCTCATGATCTCATCACCACAT 25813

Gorilla TTTCTATAAGGATACTAATCCCATTCATGAGGATTCACCCTCATGATCTCATCACCACAT 16882

***********************************************************

Human GAAGCCCCCACCTCCAAATACCATTGTAATATGGTGTGGCTGTGTCCCTACCCAAATCTC 16946

Chimpanzee GAAGCCCCCACCTCCAAATACCATTGTAATATGGTGTGGCTGTGTCCCTTCCCAAATCTC 25873

Gorilla GGAGCCCCCACCTCCAAATNNNNNNNNNNNNNNNNNNNGCTCTGTNNNTACCCAAATCTC 16942

* ***************** *** *** * **********

Human ACCTTGAATGATAATGATCCCCACATGTTAAGGGCAGGACCAAGTGGAGATAACTGAATC 17006

Chimpanzee ACCTTGAATGATAATGATCCCCACATGTTAAGGGCAGGACCAAGTGGAGATAACTGAATC 25933

Gorilla ACCTTGAATGATAATGATCCCCACATGTTAAGGGCAGGACCAAGTGGAGATAACTGAATC 17002

************************************************************

Human ATGGCAGTAGGTATTCCCCATGCTGTTCTCGTGATAGTGAATAAGTCTCATGAGAACTGA 17066

Chimpanzee ATGGCAGTAGGTATTCCCCATGCTGTTCTTGTGATAGTGAATAAGTCTCATGAGAACTGA 25993

Gorilla ATGGCAGTAGGTATTCCCCATGCTGTTCTTGTGATAGTGAANNNNNNNNNNNNANNNNNN 17062

***************************** ***********

Human TGGTTTTATAAAGGGGAGTTCCCCTGCAGAAACTCTTTTTCCTGCCACCATATAAGATGT 17126

Chimpanzee TGGTTTTATAAAGGGGAGATCCCCTGCAGAAACTCTTTTTCCTGCCACCATATAAGATGT 26053

Gorilla NNNNNNNNNNNNNNGGAGTNNNNNNNNNNNNNNNNNNNNNNNNNNNNNNNNNNNNNNNNN 17122

****

Human GCCTTTCTTCATGATTGTGAGCCTTACCCCTCAATCATGTGGAACTGTGAGTCAGTTAAA 17186

Chimpanzee GCCTTTCTTCATGATTGTGAGCCTTAACCCGCAATCATGTGGAACTGTGATTCAGTTAAA 26113

Gorilla NNNNNNNNNNNNNNNNNTGAGNCTTACCCCCCAGTCATGTGGANNNNNNNNNNNNNNNNN 17182

**** **** *** ** *********

Human GCTCTTTCCTTTGTAAATTGCTTTGTCTTGGGTATTAGCAGCATAAGAAGAGACTAATAC 17246

Chimpanzee GCTCTTTCCTTTGTAAATTGCCTTGTCTTGGGTATTAGCAGCATAAGAAGGGACTAATAC 26173

Gorilla NNNNNNNNNNTTGTAAATTGCCTTGTCTTGGGTATTANNNNNNNNNNNNNNNNNNNNNNN 17242

*********** ***************

Human ACATTGCATTGAGGTTTATGATTTCACCATAAACATTTCAGAAGACACAAAAATTCTTAC 17306

Chimpanzee ACATTGCATTGAGGTTTATGATTTCACCATAAACATTTCAGAAGACACAAAAATTCTTAC 26233

Gorilla NNNNNNNNNNNNNNNNNNNNNNNNNNNNNNNNNNNNNNNNNNNNNNNNNNNNNNNNNNNN 17302

Human CATAGCAAAGATCAAGATAATTTCTTTTACAATTATAGAAAATATGACAAATGTATGTGT 17366

Chimpanzee CATAGCAAAGATCAAGATAATTTATTTTACAGTTATAGAAAATATGACAAATGTATGTGT 26293

Gorilla NNNNNNNNNNNNNNNNNNNNNNNNNNNNNNNNNNNNNNNNNNNNNNNNNNNNNNNNNNNN 17362

Human TTGTTGGATGGAAAGGTTTCACTAGGGCAAGAGCTAAGTAGCTGTATCCTTGAAAGGAAA 17426

Chimpanzee TTGTTGGATGGAAAGGTTTCACTAGGGCAAGAGCTAAGTAGCTGTATCCTTGAAAGGAAA 26353

Gorilla NNNNNNNNNNNNNNNNNNNNNNNNNNNNNNNNNNNNNNNNNNNNNATCCTTGAAAGGAAA 17422

***************

Human AGAGAAGAGAGAATCTATTGCACAGATAAAGGGATTATGTTTAGAGGAAAACATGTACCA 17486

Chimpanzee AGAGAAGAGAGAATCTATTGCACAGATAAAGGGATTAGGTTTAGAGGAAAACATGTACCA 26413

Gorilla AGAGAAGAGAGAATCTATTGCACAGATAAAGGGATTATGTTTAGAGGAAAACATGTACCA 17482

************************************* **********************

Human CTCATCCACATTAAGAAACTACAGGGAAGAGTGTGTAGGTTGAGATGTCGGTAGCTTGAT 17546

Chimpanzee CTCACCCACATTAAGAAACTACAGGGAAGAGTGTGTAGGTTGAGATGTTGGTAGCTTGAT 26473

Gorilla CTCATCCACATTAAGAAACTACAGGGAAGAGTGTGTAGTTTGAGATGTTGGTANNNNNNN 17542

**** ********************************* ********* ****

Human GCATGTGCAGGTGGAAGCCCATGGAAGCTTGTATCTCATCCCTTCCCTACTCTTGATGAA 17606

Chimpanzee GCATGTGCAGGTGGAAGCCCATGGAAGCTTGTATCTCATCCCTTCCCTACTCTTGATGAA 26533

Gorilla NNNNNNNNNNNNNNNNNNNNNNNNNNNNNNNNNNNNNNNNNNNNNNNNNNNNNNNNNNNN 17602

Human AGAGAACTTAATCTCCTGATTAGCAGAGAAAAGTGTTGGAATTCCAGGAAAAGAAACTAT 17666

Chimpanzee AGAGAACTTAATCTCCTGATTAGCAGAGAAAAGTGTTGGAATTCCAGGAAAAGAAACTAT 26593

Gorilla NNNNNNNNNNNNNNNNNNNNNNNNNNNNNNNNNNNNNNNNNNNNNNNNNNNNNNNNNNNN 17662

Human AAAACAGCTCTCAGAAGGAGTTAAAGTATTCATAGACTGGGAAAGTATAGCAACAGCTCC 17726

Chimpanzee AAAACATCTCTCAGAAGGAGTTAAAGTATTCATAGACTGGGAAAGTATAGCAACAGCTCC 26653

Gorilla NNNNNNNNNNNNNNNNNNNNNNNNNNNNNNNNNNNNNNNNNNNNNNNNNNNNNNNNNNNN 17722

Human TGGAATCCCAACTTGGAGCTAATTAATCACAAATATAAAGTCTGAGCAGCTGGTATGGTT 17786

Chimpanzee TGGAATCCCAACTTGGAGCTAACTAATCACAAATATAAAGTCTGAGCAGCTGGTATGCTT 26713

Gorilla NNNNNNNNNNNNTTGGAGCTAATTAATCACAAATGTCAAGTCTGAGCANNNGGTATGGTT 17782

********** *********** * *********** ****** **

Human ATTTGTGTGCACAGATGAAGGCCAATGGAGTGTAGGTGGAGTTTTATTTAGCCAGAATTG 17846

Chimpanzee ATTTGTGTGCACAGATGAAGGCCAATGGAGTGTAGGCGGAGTTTTATTTAGCCAGAATTG 26773

Gorilla ATTTGTGTGCACAGATGAAGGCCAATGGAGTGTAGGTGGAGTTTTATTTAGNNNNNNNNN 17842

************************************ **************

Human TGGTTAAAACCCAGTCTAAAAGAAAGCAAGAAGGAAGCAAGAGATTCAGGGAATCCCCGT 17906

Chimpanzee TGGTTACGACCCAGTATAAAAGAAAGCAAGAACGAAGCAAGAGATTCAGGGAATCCCCGT 26833

Gorilla NNNNNNNNNNNNNNNNNNNNNNNNNNNNNNNNNNNNNNNNNNNNNNNNNNNNNNNNNNNN 17902

Human GGGAGTGATTGTGAGGTTTGGCAATGTGATTTCAGTGGGGAAATAGGAAAAAGAAAAGAA 17966

Chimpanzee GGGAGTGATTGTGAGGTTTGGCAATGTGATTTCAGTGGGGAAATAGGAAAAAGAAAAGAA 26893

Gorilla NNNNNNNNNNNNNNNNNNNNNNNNNNNNNNNNNNNNNNNNNNNNNNNNNNNNNNNNNNNN 17962

Human GATGCATTTCAGTAAAATGTAGTGGTGTGCTGCAAATACTTGAGTTATTAGAAAGGGGAA 18026

Chimpanzee GATGCATTTCAGTAAAATGCAGTGGTGTGCTGCAAATACTTGAGTTATTAGAAAGGGGAA 26953

Gorilla NNNNNNNNNNNNNNNNNNNNNNNNNNNNNNNNNNNNNNNNNNNNNNNNNNNNNNNNNNNN 18022

Human GTTGTGCTATTAAGATTATAGAGGAGTGGTACTTATTGGAACTTATAATACGTAAGAAAT 18086

Chimpanzee GTTGTGCTATTAAGATTATAGAGGAGTGGTACTTATTGGAACTTATAACACGTAAGAAAT 27013

Gorilla NNNNNNNNNNNNNNNNNNNNNNNNNNNNNNNNNNNNNNNNNNNNNNNNNNNNNNNNNNNN 18082

Human TAGGTAAAATATAGAAAAATGTATTGGAGGTAAGATTTTAGAGAATTTGAGAGGTGAGTG 18146

Chimpanzee TACGTAAAATATAGAAAAATATATTGGAGGTAAGATTTTAGAGAATTTGAGAGGTGAGTG 27073

Gorilla NNNNNNNNNNNNNNNNNNNNNNNNNNNNNNNNNNNNNNNNNNNNNNNNNNNNNNNNNNNN 18142

Human TGTTGAAAAATTACATGTATATATCCTAATATCAAGAATTAGGGCTTGGTGAAGTGGTTC 18206

Chimpanzee TGTTGAAAAATTACATGTATATATCCTAATATCAAGAATTAGGGCTTGGTGAAGTGGTTC 27133

Gorilla NNNNNNNNNNNNNNNNGNNNNNNNNNNNNNNNNNNNNATTAGGGCTTGGTGAAGTGGNNN 18202

* ********************

Human ACACCTGTAATCACAAGACTTTAGAAGGCTGAGGTGGTAGGATCACTTGTGGCCAGGAGT 18266

Chimpanzee ACACCTGTAATCACAAGACTTTAGAAGGCTGAGGTGGTAGGATCACTTGTGGCCAGGAGT 27193

Gorilla NNNNNNNNNNNNNNNNNNNNNNNNNNNNNNNNNNNNNNNNNATCACTTGTGGCCAGGAGT 18262

*******************

Human TTGAGAACATCCTGGGTAACATAGTAGACCCAGTTTCTACAAAATAATTAAAATAATTAG 18326

Chimpanzee TTGAGAACATCCTGGGTAACATAGTAGACCCAGTTTCTACAAAATAATTAAAATAATTAG 27253

Gorilla TTGAGAACANNNNNNNNNNNNNNNNNNNNNNNNNNNNNNNNNNNNNNNNNNNNNNNNNNN 18322

*********

Human CTGGGCATCATGGTGCATGCCTGTATTCCCAGCTATTCCAGAGGTTGAGGCAGGATGATC 18386

Chimpanzee CTGGGCATCATGGTGCATGCCTGTATTCCCAGCTATTCCAGAGGTTGAGTCAGGATGATC 27313

Gorilla NTGGGCATCATGGTGCATGCCTGTATTCCCAGCTANNNNNGAGGTTGAGGCAGGANNNNN 18382

********************************** ********* *****

Human ACTTGCCCTCAGGAGTTCCAGGCTGTAGTGAGCCATAATTGCACCACTGTACTCCAGCCT 18446

Chimpanzee ACTTGCCCTCAGGAGTTCCAGGCTGTAGTGAGCCATAATTGCACCACTGTACTCCAGCCT 27373

Gorilla NNNNNNNNNNNNNNNNNNCAAGCTGTAGTGAGCCNNAATTGCACCACTGTACTCCAGCCT 18442

** ************* ************************

Human GGGCCGTAGAATTAGATCCTGTCTCAAAAGAAAACAAAAAAGAATAACCTCATTAGGAGA 18506

Chimpanzee GGGCCGTAGAATTAGATCCTGTCTCAAAAGAAAACAAAAAAGAATAACCTCACTAGGAGA 27433

Gorilla GGGCCGTAGAATTNNNNCCTGTCTCAAAAGAAAACAAAAAAGAATAACCTCATTAGGAGA 18502

************* *********************************** *******

Human TGACGAAAATTAAGAAGGAGGAACAATGTTGAGAAATAATTGGTACTAAGTTGGGTGTCA 18566

Chimpanzee TGATGAAAATTAAGAAGGAGGAACAATGTTGAGAAATAATTGGGACTAAGTTGGGTGTCA 27493

Gorilla TGATGNNNNNNNAGAAGGAGGAACAATGNNNNNNNNTANNNGGGACTANNNNNNNNNNNN 18562

*** * **************** ** ** ****

Human GTATAGGAGGTTGATATAATTTGATATCATAAGATCATAAAATGATGAAATTGATAAAAT 18626

Chimpanzee GTATAGGAGGTTGATATAATTTGATATCATAAGATCATAAAACGATGAAATTGATAAAAT 27553

Gorilla NNNNNNNNGGTTNNNNNNANCTNNNNNNNNNNNNNNNNNAAATGNNGAAATTGATAAAAT 18622

**** * * *** * **************

Human CAGATGCCATGACATCCGAAGCTGGGTGGTTTAAGAAGCCAGAGGACAAATGATCTGAAG 18686

Chimpanzee CAGATGCCATGACATCCGAAGCTGGGTGGTTTAAGAAGCCAGAGGACAAATGATCTGAAG 27613

Gorilla CAGATGTCATGACATCCGAAGCTGGGTGNNNTAAGAAGCCAGAGGACAAATGATCTGAAG 18682

****** ********************* *****************************

Human GTGGAAATTTGAACGAGGCAAACATGGAAGCTAATTCCAGCATTAAGAGTCCAAGGCTTT 18746

Chimpanzee GTGGAAATTTGAACGAGGCAAACATGGAAGCTAATTCCAGCATTAAGAGTCCAAGGCTTT 27673

Gorilla GTGGAAANNNNNNCGAGGCAAACATGGAAGCTAATTCCAGCATTAAGAGTCCAAGGCTTT 18742

******* ***********************************************

Human AAGGCATTTCTTCTTATGACCCCTCCACTTAACCCAGAATCTTAAATAACTGCAGGGGAG 18806

Chimpanzee AAGGCATTTCTTCTTATGACCCCTCCACTTAACCCAGAATCTTAAATAACTGCAGGGGTG 27733

Gorilla AAGGCATTTCTTCTTATGACCCCTCCACNTAACCCAGAATCTTAAATAACTGCAGGGGAG 18802

**************************** ***************************** *

Human GTGATGTCCTTGAGGGAGGGTTTGAGAAGCAGTAGTAACAAGATCTCCATAATTCCATGA 18866

Chimpanzee GTGATGTCCTTGAGGGAGGGTTTTAGAAGCAGTAGTAACAAGATCACCATAATTCCATGA 27793

Gorilla GTGATGTCCTTGAGGGAGGGTTTTAGAAGCAGTAGNNNNNNGATCTCCATAATTCCATGA 18862

*********************** *********** **** **************

Human AGCAATGAGAAAATTTCAGAGGTTGTATACAGGAAGGAGATGAATCATCAGATGGGTTCC 18926

Chimpanzee AGCAATAAGAAAATTTCAGAGGTTGTATACAGGAAGGAGATGAATCATCAGATGGGTTCC 27853

Gorilla AGCAATGAGAAAATTTCAGAGGTTGTATACAGGAAGGAGATGAATCATCAGATGGGTTCT 18922

****** ****************************************************

Human ATGGATTATTGGAGTCCAGGAGGTGAAGCGTGATGACCTGTAAGCTACTGCTATTGTATG 18986

Chimpanzee ATGGATTATTGGAGTCCAGGAGGTGAAGCGTGATGACCTGTAAGCTACTGCTATTTTATG 27913

Gorilla ATGGATTATTTGAGTCCAGGAGGTGAAGTGTGATGACCTGTAAGCTACTGCTATTGTATG 18982

********** ***************** ************************** ****

Human ACTGATAAACACAGAGAAATGGAATGCCAGGCCACAGTGGGAGCCAGGACATGAGTGCCG 19046

Chimpanzee ACTGATAAACACAGAGAAATGTAATGCCAGGCCACAGTGGGAGCCAGGACATGAGTGCCG 27973

Gorilla ACTGATAAACACAGATAAATGGAATGCCAGGCCACAGTGGGAGCCAGGACATGAGTGCCG 19042

*************** ***** **************************************

Human CAGGTTAGGGCTGTCTGCGTTTCAAAAGTTTTCTTGTTACCCCTGGCAATGGAGCCTGGA 19106

Chimpanzee CAGGTTAGGGCTGTCTGCATTTCAAAAGTTTTCTTGTTACCCCTGGCAATGGAGCCTGGA 28033

Gorilla CAGGTTAGGGCTGTCTGCATTTCGAAAGTTTTCTTGTTACCCCTGGCAATGGAGCCTGGA 19102

****************** **** ************************************

Human AGAGGAGGTAAAGTATTATCATGTCCCAGAGCCTCACTTTTGGGAAGTTATGTGGCTCTG 19166

Chimpanzee AGAGGAGGTAAAGTATTATCATGTCCCAGAGCCTCACTTTTGGGTAGTTATGTGGCTCTG 28093

Gorilla AGAGAAGGTAAAGTATTATCATGTCTCAGAGCCTCACTTTTGGGTAATTATGTGGCTCTG 19162

**** ******************** ****************** * *************

Human GATTGTCTGAAATATCTAAAATCACTCTTATTTACACAACTGTATAATTATTTAAGCATT 19226

Chimpanzee GATTCTCTGAAATATCTAAAATCACTCTTATCTACACAACTGTATAAGTATTTAAGCGTT 28153

Gorilla GATTCTCTGAAATATCTAAAATCAGTCTTATTTACAGAACTGTGTAATTNNNNNNNNNNN 19222

**** ******************* ****** **** ****** *** *

Human AGACCTAAAACCATAAACACCCTAGAAGAAAACATAGGCATTACCATTCAGGACATAGGC 19286

Chimpanzee AGACCTAAAACCATAAACACCCTAGAAGAAAACCTAGGCATTACCATTCAGGACATAGGC 28213

Gorilla AGACCTANNNNNNNNNNNNNNNNNNNNNNNNNNNNNGGCATTACCATTCAGGACATAAGC 19282

******* ********************* **

Human ATGGGGAAGGACTTCATGTCTAAAACAACAAAAGCAATGGCAACAAAAGCCAAAATTGAC 19346

Chimpanzee ATGGGGAAGGACTTCATGTCTAAAACAACAAAAGCAATGGCAACAAAAGCCAAAATTGAC 28273

Gorilla ATGGGGAAGGACTTCATGTCTAANNNNNNNNNNNNNNNNNNNNNNNNNNNNNNNNNNNNN 19342

***********************

Human AAATGGGATCTAATTAAACTAAAGAGCTTCTGCACACCAAAAAAAAAAAAAAAAAAAA-- 19404

Chimpanzee AAATGGGATCTAATTAAACTAAAGAGCTTCTGCACAGCAAAAAAAAAAAAAAAAAAAAAA 28333

Gorilla NNNNNNNNNNNNNNNNNNNNNNNNNNNNNNNNNNNNNNNNNNNNNNNNNNNNNNNNNN-- 19400

Human -------------------CTAACATCAGAGTGAACAAGCAACCTACAAAATCGGAGAAA 19445

Chimpanzee AAAAAAAAAAAAAAAAAAACTAACATCAGAGTGAACAAGCAACCTACAAAACGGGAGAAA 28393

Gorilla -------------------NNNNNNNNNNNNNNNNNNNNNNNNNNNNNNNNNNNNNNNNN 19441

Human ATTTTCACAACCTACTCATCTGCCAAAGGGCTAATATCCAGAATCTACAATGAACTCAAA 19505

Chimpanzee ATTTTCACAACCTACTCATCTGACAAAGGGCTAATATCCAGAATCTACAATGAACTCAAA 28453

Gorilla NNNTTCACAACCTACTCATCTGCCAAAGGGCTAATATCNNNNNNNNNNNNNNNNNNNNNN 19501

******************* ***************

Human CAAATTTACAAAAAAAAAACACCAACAACCCCATCAAAAAGTGGGCGAAGGACATGAACA 19565

Chimpanzee GAAATTTACAAGAAAAAAAC--CAACAACCCCATCAAAAAGTGGGCAAAGGACATGAACA 28511

Gorilla NNNNNNNNNNNNNNNNNNNNNNNNNNNNNNNNNNNNNNNNNNNNNNNNNNNNNNNNNNNN 19561

Human GACACTTCTCAAAAGAAGACATTGATGCAGCCAAAAAACATATGAAAAAATGCTCACCAT 19625

Chimpanzee GACACTTCTCAAAAGAAGACATTGATGCAGCCAAAAAACGTATGAAAAAATGCTCACCAT 28571

Gorilla NNNNNNNNNNNNNNNNNNNNNNNNNNNNNNNNNAAAAACATATGAAAAAATGCTCACCAT 19621

****** ********************

Human CACTTGCCATCAGAGAAATGCAAATCAAAACCACAATGAGATACCAGCTCACACCATTTA 19685

Chimpanzee CACTTGCCATCAGAGAAATGCAAATCAAAACCACAATGAGATACCATCTCACACCATTTA 28631

Gorilla CACTTNNNNNNNNNNNNNNNNNNNNNNNNNNNNNNNNNNNNNNNNNNNNNNNNNNNNNNN 19681

*****

Human GAATGGTGATCATTAAAAAGTCAGCAAACAACAGGTGCTGGAGAGGATGTGGAGAAATAG 19745

Chimpanzee GAATGGTGATCATTAAAAAGTCAGCAAACAACAGGTGCTGGAGAGGATGTGGAGAAATAG 28691

Gorilla NNNNNNNNNNNNNNNNNNNNNNNNNNNNNNNNNNNNNNNNNNNNNNNNNNNNNNNNNNNN 19741

Human GAACGCTTTTACACTGTTGGTGGGACTGTAAACTAGTTCAAACATTGTGGAAGTCAGTGT 19805

Chimpanzee GAACGCTTTTACACTGTTGGCGGGACTGTAAACTAGTTCAAACATTGTGGAAGTCAGTGT 28751

Gorilla NNNNNNNNNNNNNNNNNNNNNNNNNNNNNNNNNNNNNNNNNNNNNNNNNNNNNNNNNNNN 19801

Human GGCAATTCCTCAGGGATCTAGAATTAGAAATACCATTTGACCCAGCCATCCCATTACTGG 19865

Chimpanzee GGCGATTCCTCAGGGATCTAGAATTAGAAATACCATTTGACCCAGCCATCCCATTACTGG 28811

Gorilla NNNNNNNNNNNNNNNNNNNNNNNNNNNNNNNNNNNNNNNNNNNNNNNNNNNNNNNNNNNN 19861

Human GTATATACCCAAAGGACTATAAATCATGCTGCTATAAAGACACATGCACACGTATGTTTA 19925

Chimpanzee GTATATACCCAAAGGACTATAAATCATGCTGCTATAAAGACACATGCACACGTATGTTTA 28871

Gorilla NNNNNNNNNNNNNNNNNNNNNNNNNNNNNNNNNNNNNNNNNNNNNNNNNNNNNNNNNNNN 19921

Human TTGTGGCACTATTCACAATAGCAAAGACTTGGAACCAACCCAAATGTCCAACAATGATGG 19985

Chimpanzee TTGTGGCACTATTCACAATAGCAAAGACTTGGAACCAACCCAAATGTCCAACAATGATGG 28931

Gorilla NNNNNNNNNNNNNNNNNNNNNNNNNNNNNNNNNNNNNNNNNNNNNNNNNNNNNNNNNNNN 19981

Human ACTGGATTAAGAAAATGTGGCACATATACACCATGGAATACTATTGCAGCCGTAAAAAAT 20045

Chimpanzee ACTGGATTAAGAAAATGTGGCACATATACACCATGGAATACTAT-GCAGCCATAAAAAAT 28990

Gorilla NNNNNNNNNNNNNNNNNNNNNNNNNNNNNNNNNNNNNNNNNNNNNNNNNNNNNNNNNNNN 20041

Human GATGAGTTCATGTCCTTTGTAGGGACATGGATGAAATTGGAAATCATCATTCTCAGTAAA 20105

Chimpanzee GATGAGTTCATGTCCTTTGTAGGGACATGGATGAAATTGGAAATCATCATTCTCAGTAAA 29050

Gorilla NNNNNNNNNNNNNNNNNNNNNNNNNNNNNNNNNNNNNNNNNNNNNNNNNNNNNNNNNNNN 20101

Human CTATCGCTAGAACAAAAAACCAAACACCGCATATTCTCACTTATAGGTGGGAATTGAACA 20165

Chimpanzee CTATCGCCAGAACAAAAAACCAAACACCGCATATTCTCACTTATAGGTGGGAATTGAACA 29110

Gorilla NNNNNNNNNNNNNNNNNNNNNNNNNNNNNNNNNNNNNNNNNNNNNNNNNNNNNNNNNNNN 20161

Human GTGAGAACGCATGGACACAGGAAGGGGAACATCACACTCTGGGGACTGTTGTGGGGTGGG 20225

Chimpanzee GTGAGAACACATGGACACAGGAAGGGGAACATCACACTCTGGGGACTGTTGTGGGGTGGG 29170

Gorilla NNNNNNNNNNNNNNNNNNNNNNNNNNNNNNNNNNNNNNNNNNNNNNNNNNNNNNNNNNNN 20221

Human GGGAGGGGGGAGGGATAGCATTAGGAGATATACCTAATGCTAAATGACGAGTTAATGGGT 20285

Chimpanzee GGGAGGGGGGAGGGATAGCATTAGGAGATATACCTAATGCTAAATGACGAGTAAATGGGT 29230

Gorilla NNNNNNNNNNNNGGATAGCATTAGGAGATATACCTAATGCTAAATGACAAGTTAATGGGT 20281

************************************ *** *******

Human GCAGCACACCAGCATGGCACATGTATACATATGTAACTAACCTGCACATTGTGCACATGT 20345

Chimpanzee GCAGCACACCAGCATGGCACATGTATACATATGTAACTAACCTGCACATTGTGCACATGT 29290

Gorilla GCAGCACACCAGCATGGCACATGTATACATATGTAACTTACCGGAACATTGTGCACATGT 20341

************************************** *** * ***************

Human ACCCTAAAACTTAAAGTATAATAATAAAAAAATCTATGATGGCATTTAGGAATTGCCTGG 20405

Chimpanzee ACCCTAAAACTTAAAGTATAATAATAAAAAAATCTATGATGGCATTTAGGAATTGCCTGG 29350

Gorilla ACCCTAAAACTTAAAGTATAATAATAAAAAAATCTACGATGGCATTTAGGAATTGCCTGG 20401

************************************ ***********************

Human ATACACTAGAAACGAAACCCAGGCAAAACCACCCAACAATCCTCAGTATGCATTTATTAA 20465

Chimpanzee ATACACTAGAAACGAAACCCAGGCAAAACCAACCAACAATCCTCAGTATGCATTTATTAA 29410

Gorilla AGACACTAGAAACCAAACCCAGGCAAAACCAACCAACAGTCTTCAGTATGCATTTATTAA 20461

* *********** ***************** ****** ** ******************

Human ATCAATGCTATAAAATCATCTTACCTTGTTAACATTTACAAAGTCCCCTGGAGGTTTTCT 20525

Chimpanzee ATCAATGCTATAAAATCATCTTACGTTGTTAACATTTACAAAGTCCCCTGGAGGTTTTCT 29470

Gorilla ATCAATGCTATAGAATCATCTTACCCTGTTAACATTTACAAAGTCCCCTGGAGGTTTTCT 20521

************ *********** **********************************

Human TCAGTGACTTAATCAGCTTGTGTCTTCAACACCTTCCTATATCCCTTATTCATTATAGAT 20585

Chimpanzee TCAGTGACTTAATCAGCTTGTGTCTTCAACACCTTCCTATATCCCTTATTCATTATAGAT 29530

Gorilla TCAGTGACTTAATCAGCTTGTGTCTTCAACACCTTCCCATACCCCTTATTCATTATAGAT 20581

************************************* *** ******************

Human CCTTCCTATATAGATGGTCTATGGGAACTGGCTGATTATTTTTAACATAGCACACATCAA 20645

Chimpanzee CCTTCCTATATAGATGGTCTATGGGAACTGGCTGATTATTTTTAACATAGCACACATCGA 29590

Gorilla CCTTCCTATATAGATGGTCTATGGGAACTGGCTGATTATTTTTAACATAACACACATCAA 20641

************************************************* ******** *

Human AATAGTTTTCCACTTTTTTACCTTGGAATTCAATAATTTTTAACAAAAGACACTGGTATT 20705

Chimpanzee AATAGTTTTCCACTTTTTTACCTTGGAATTCAATAATTTTTAACAAAAGACACTGGTATT 29650

Gorilla AATAGTTTTCCACTTTTTTACCTTGGAATTCAATAATTTTTAACAAAAGACACTGGTATT 20701

************************************************************

Human GTTTTCTTTCCATGAGTCAGAAATAAGTCAATTTGCTTTTTAACTTTCACTAAGTAAGTC 20765

Chimpanzee GTTTTCTTTCCATGAGTCAGAAATAAGTCAATTTGCTTTTTAACTTTCACTAAGTAAGTC 29710

Gorilla GTTTTCTTTCCATGAGTCGGAAATAAGTCAATTTGCTTTTCAACTTTCACTAAGTAAGTC 20761

****************** ********************* *******************

Human ATAGCAAAGAATCCAAAGTAAGTATCTTATGTATGCAAAATGTAGACGATGCTATTGGAA 20825

Chimpanzee ATAGCAAAGAATCAAAAGTAAGTATCTTATGTATGCAAAATGTAGACAATGCTATTGGAA 29770

Gorilla ATAGCAAAGAATCAAAAGTAAGTATCTTATGTATGCAAAATGTAGACAATGCTATTGGAA 20821

************* ********************************* ************

Human GTTTAGCAGTAATAGGTGTATACATGTTAAAGAAAAAGTTGAATATACCATATAGCACAA 20885

Chimpanzee GTTTAGCAGTAATAGGTGTATACATGTTAAAGAAAAAGTTGAATATACCATATAGCACAA 29830

Gorilla GTTTAGCAGTAATAGGTGTATACATGCTAAAGAAAAAGTTGAATATACCATATAGCACAA 20881

************************** *********************************

Human AATATTACTGAAGTTGGCTTCCCGATCTTTCAACAGGACTGAACAACAAGGTGCATAAAA 20945

Chimpanzee AATATTACTGAAGTTGGCTTCCCGATCTTTCAACAGCACTGAACAACAAGGTGCATAAAA 29890

Gorilla AATATTACTGAAGTTGGCTTCCCGATCTTTCAACAGGACTGAACAACAAGGTGCATAAAA 20941

************************************ ***********************

Human TTTCAGAGACATGGGAAACACTCTCAAGCAGAAAACAGAGTCAGCTCCTTATGGGACAAA 21005

Chimpanzee TTTCAGAGACATGGGAAATACTCTCAAGCAGAAAACAGAGTCAGCTCCTTATGGGACAAA 29950

Gorilla TTTCAGAGACATTGGAAATACTCTCAAGCAGAAAACAGAGTCAGCTCCTTATGGGACAAA 21001

************ ***** *****************************************

Human ATTGGCTTGAGCAATTATTCCTTGGAAATGTTGCAAAGAGTTCAATGGCCACCTATTGTC 21065

Chimpanzee ATTGGCTCGAGCAATTATTCCTTGGAAATGTTGCAAAGAGTTCAATGGCCACCTATTGTC 30010

Gorilla ATTGGCTTGAGCAATTATTCCTTGGAAATGTTGCAAAGAGTTCAATGGCCACCTATTGTA 21061

******* ***************************************************

Human ACCAAGAGCTGCTGATTTTGCTGGAAAGAGCTTCCCAAAATTGCCTCAGGCAGCTGCTAG 21125

Chimpanzee ACCAAGAGCTGCTGATTTTGCTGGAAAGAGCTTCCCACAATTGCCTCAGGCAGCTGCTAG 30070

Gorilla ACCAAGAGCTGCTGATTTTGCTGGAAAGAGCTTCCCAAAATTGCCTCAGGCAGCTGCTAA 21121

************************************* *********************

Human TGGAAGACAATCTGAAGCTCATGGGAGTGGACTTGACTTCAAGTTTGTAAGAAGTAAAGT 21185

Chimpanzee TGGAAGACAATCTGAAGCTCATGGGACTGGACTTGACTTCAAGTTTGTAAAAAGTAAAGT 30130

Gorilla TGGAAGACAATCTGAAGCTCATGGGACTGGACTTGACTTCAAGTTTGTAAAAAGTAAAGT 21181

************************** *********************** *********

Human AGAGGTTCCTCTTCAAAGACTTTCCTCCCCATTTAGGAATAAATAGTAACTTCTCTTAGA 21245

Chimpanzee AGAGGTTCCTCTTCAAAGACTTTCCTCCCCATTTAGGAATAAATAGTAACTTCTCTTAGA 30190

Gorilla AGAGGTTCCTCTTCAAAGACTTTCCTCCCCATTTAGGAATAAATAGTAACTTCTCTTAGA 21241

************************************************************

Human AGCAATATTTATTCAAAGACCTGTGCTAACATTCTTAAATGTCTGCTAGCCATAATGAAT 21305

Chimpanzee AGCAATATTTATTCAAAGACCTGTGCTAACATTCTTAAATGTCTGCTAGCCATAATGAAT 30250

Gorilla AGCAATATTTATTCAAAGACCTGTGCTAACATTCTTAAATGTCTGCTAGCCATAATGAAT 21301

************************************************************

Human CAATGTATTTTATGTTCTTAGCTCCCACAATTTAGCATAAATATTTGCCCTGGCATGCTT 21365

Chimpanzee CAATGTATTTTATGTTCTTAGCTCCCACAATTTAGCATAAATATTTGCCCTGGCATGCTT 30310

Gorilla CAATGTATTTTATGTNNNNNNNTCCCACAATTTAGCATAANNNNNNNNNNNNNNNNNNNN 21361

*************** ******************

Human ATACTGGTCCAAGCAAGCGTTAGGTCATAGTGTGTTCTTCTTCCTTATTTAAAAGTGTTT 21425

Chimpanzee ATACTGGTCCAAGCAAGTGTTAGGTCATAGTGTGTTCTTCTTCCTTATTTAAAAGTGTTT 30370

Gorilla NNNNNNNNNNNNNNNNNNNNNNNNNNNNNNNNNNNNNNNNNNNNNNNNNNAAAAGTGTTT 21421

**********

Human TTAACTTTCTCAACATCTCACAAGTTACTTCCTCCTTCCTTTGTTCTCCTATACCTTTGT 21485

Chimpanzee TTAACTTTCTCAGCATCTCACAAGTTACTTCCTCCTTCCTTTGTTCTCCTATACCTTTGT 30430

Gorilla TTAACTTTCTCAGCATCTCACAAGTTNNNNNNNNNNNNNNNNNNNNNNNNNNNNNNNNNN 21481

************ *************

Human GTCTTTTAAAACATTGCAAGTTGCTGGCCAATCGGGACAAATACAGAATGTGAGGTCACA 21545

Chimpanzee GTCTTTTGAAAAATTGCAAGTTGCTGGCCAATCGGGACAAATACAGAATGTGAGGTCACA 30490

Gorilla NNNNNNNNNNNNNNNNNNNNNNNNNNNNNNNNNNNNNNNNNNNNNNNNNNNNNNNNNNNN 21541

Human TTCCAGCTGATGGAGACTGGACACGGCAGTAGGATGGACGCATCAGGATATAAATGACCC 21605

Chimpanzee TTCCAGCTGATGGAGACTGGACACGGCAGTAGGATGGACGCATCAGGATATAAATGACCC 30550

Gorilla NNNNNNNNNNNNNNNNCTGGACATGGCAGTAGGATGGACGCATCAGGATATAAATGACCC 21601

******* ************************************

Human TGTCACCTTTGTTCGGTGTACTCTCGTGACAAAACTGCTGGAGAGTGTATACCCTTTTTA 21665

Chimpanzee TGTCACCTTTGTTCGGTGTACTCTCGTGACAAAACTGCTGGAGAGTGT--ACCCTTTCTA 30608

Gorilla TGTCACCTTTGTTTGGTGTACTCTTGTGACAAAACTGCTGGAGAGTG--TACCCTTTCTA 21659

************* ********** ********************** ******* **

Human CAGGAAGTAAAAATTGCCTTACTAAATAGATTAAATTTATGTTCGGGTGCTATTTCTTTA 21725

Chimpanzee CAGGAAGTAAAAATTGCCTTACTAAATAGATTAAATTTATGTTCGGGTGCTATTTCTTTA 30668

Gorilla CAGGAAGTAAAAATTGCCTTACTAAATAGATTAAATTTATGTTCAGGTGCTATTTCTTTA 21719

******************************************** ***************

Human TGGCACCAGGCCACAAACACTTCAGACAAGTTCATAAACTACCAGGTATGTTAAATTGAT 21785

Chimpanzee TGGCACCAGGCCACAAACACTTCAGACAAGTTCATAAACTACCAGGTATGTTAAATTGAT 30728

Gorilla TGGCACCAGGCCACAAACACTTCAGACAAGTTCATAAACTACCAGGTATGTTAAATTGAT 21779

************************************************************

Human TGGCCCCAATATTTCTATTGTCTACTGCTATACCAGTGTAAATCATTTTATTGGACATTT 21845

Chimpanzee TGGCCCCAATATTTCTATTGTCTACTGCTATACCAGTGTAAATCATTTTATTGGACATTT 30788

Gorilla TGGCCCCAATATTTCTATTGTCTACTGCTATACCAGTGTAAATCATTTTATTGGACATTT 21839

************************************************************

Human AAAGATGTTCGCAAATTTGAAGTTAAGAGTTTATGTGAGATAAGTAGGCAGGGTTGAAGG 21905

Chimpanzee AAAGATGTTCGCAAATTTGAAGTTAAGAGTTTATGTGAGATAAGTAGGCAGGGTTGAAGG 30848

Gorilla AAAGATGTTCGCAAATTTGAAGTTAAGAGTTTATGTGAGATAAGTAGGCAGGGTTGAAGG 21899

************************************************************

Human GAGAAGAAAGAAATTCTGTTACAAATATGATTACCATTTTTAGTAAGAAAGAAGAAAAAT 21965

Chimpanzee GAGAAGAAAGGAATTCTGTTACAAATATGATTACCATTTTTAGTAAGAAAGAAGAAAAAT 30908

Gorilla GAGGAGAAAGAAATTCTGTTACAAATATGATTACCATTTTTAGTAAGAAAGAAGAAAAAT 21959

*** ****** *************************************************

Human AAAAAGAGAATATTTAATGGCATTTTAAAAAATGTTCAGATACATAGTCATAAAGAGAAA 22025

Chimpanzee AAAAAGAGAATATTTAATGGCATTTTAAAAAATGTTCAGATACATAGTCATAAAGAGAAA 30968

Gorilla AAAAAGAGAATATTTAATGGCATTTTAAAAAATGTTCAGATACACAGTCATAAAGAGAAA 22019

******************************************** ***************

Human AACAGTAGTTCTTAGGGATGGGGTGACAAGTAGGAAATGGTAAGATGTACATCAAAAGAT 22085

Chimpanzee AACAGTAGTTCTTAGGGATGGGGTGACAAGTAGGAAATGGTAAGATGTACATCAAAAGAT 31028

Gorilla AACAATAGTTCTTAGGGA-GGGGTGACAAGTAGGAAATGGTAAGATGTACGTCAAAAGAT 22078

**** ************* ******************************* *********

Human ATAAAGTAGCAAATATGTGGATGAATAAATCTAAAGATCTAATATACAGCCTATGGACTA 22145

Chimpanzee ATAAAGTAGCAAATATGTGGATGAATAAATCTAAAGATCTAATATACAGCCTATGGACTA 31088

Gorilla ATAAAGTAGCAAATATGTGGATGAATAAAACTAAAGATCTANNNNNNNNNNNNNNNNNNN 22138

***************************** ***********

Human TAAGTAATAACACTATTGTTTGTAGGATTTCTACTAAAGGAGTAGATTATAACTCATCTT 22205

Chimpanzee TAAGTAATAACACTATTGTTTGTAGGATTTCTACTAAAGGAGTAGATTATAACTCATCTT 31148

Gorilla NNNNNNNNNNNNNNNNNNNNNNNNNNNNNNNNNNNNNNNNNNNNNNNNNNNNNNNNNNNN 22198

Human GCCAGAGGGGTGAAGTGAGTAACATGTTATGATAAATATGTAAATTAATTCCATCATAGT 22265

Chimpanzee GCCAGAGGGGTGAAGTGAGTAACATGTTATGATAAATATGTAAATTAATTCCATCATAGT 31208

Gorilla NNNNNNNNNNNNNNNNNNNNNNNNNNNNNNNNNNNNNNNNNNNNNNNNNNNNNNNNNNNN 22258

Human GAATGTTTTACTATATGTATGTAACTTATAACATCATGTTGTATACCTTAAATATATACG 22325

Chimpanzee AAATGTTTTACTATATGTATGTAACTTATAACATCATGTTGTATACCTTAAATATATACA 31268

Gorilla NNNNNNNNNNNNNNNNNNNNNNNNNNNNNNNNNNNNTGTTGTATACCTTAAATGTATACA 22318

***************** *****

Human ATAGATTTATTTTGTAAACAGTACATAATATGCAATACACATGTGTGTATACATATACAC 22385

Chimpanzee ATAGATTTATTTTGTAAACAGTACATAATATGCAATACAC--GTGTGTATACATATACAC 31326

Gorilla ATAGATTTATTTTGTAAACAGTACATAATATGCAATACACATGTGTGTATACATATACAC 22378

**************************************** ******************

Human ACATATGTACAAATATAAAATTTAAATATGCTTATAATTTACAATTGCCTATACCATAAT 22445

Chimpanzee ACATATGTGAAAATATAAAATTTAAATATGCTTATAATTTACAATTGCCTATACCATAAT 31386

Gorilla ACATATGTGCAAATATAAAATTTAAATATGCTTATAATTTACAATTGCCTATACCATAAT 22438

******** **************************************************

Human TTACACTTGAATTTACAGAATTTTTCACATGCTCAGAAGAGAA-----TAAAATGTAAAG 22500

Chimpanzee TTACACTTGAATTTACAGAATTTTTCACATGCTCAGAAGAGAAGAGAATAAAATGTAAAG 31446

Gorilla TTACACTTGAATTTACAGAATTTTTCACATGCTCAGAAGAGAA-----TAAAATGTAAAG 22493

******************************************* ************

Human GCAGAAAGAAAATACGATGGAATTAAAGCAAAGTTACACAAACAACCTGTTTTCAGATTC 22560

Chimpanzee GCAGAAAGAAAATACGATGGAATTAAAGCAAAGTTACACAAACAACCTGTTTTCAGATTC 31506

Gorilla GCAGGAAGAAAATACGATGGAATTAAAGCAAAGTTACACAAACAACCTGTTTTCAGATTC 22553

**** *******************************************************

Human CCTCGATCATGCTATTCTCATTGCCAGAGAATGTTTTGGGTTTAATGCCATAATTTGCTA 22620

Chimpanzee CCTCGATCATGCTATTCTCATTGCCAGAGAATGTTTTGGGTTTAATGCCATAATTTGCTA 31566

Gorilla CCTTGATCATGCTATTCTCATTGCCAGAGAATGTTTTGGGTTTAATGCCATAATTTGCTA 22613

*** ********************************************************

Human TGGTTGACCATACTTTATGCATTGAGCATAGTGTGGAAATATGAAAATAAAGATTGTCTG 22680

Chimpanzee TGGTTGACCATACTTTATGCATTGAGCACAGTGTGGAAATATGAAAATAAAGATTGTCTG 31626

Gorilla TGGTTAACCATACTTTATGCATTGAGCATAGTGTGGAAATATGAAAATAAAGATTGTCTG 22673

***** ********************** *******************************

Human CAGACAATCTTTTTTGTTTATGCAACCTACACTTAGAAAATGCAATTTCATTTTGCTAAA 22740

Chimpanzee CAGACAATCTTTTTTATTTATGCAACCTACACTTAGAAAATGCAATTTCATTTTGCTAAA 31686

Gorilla CAGACAATCTTTTTTATTTATGCAACCTACACTTAGAAAATGCAATTTCATTTTGCTAAA 22733

*************** ********************************************

Human TTAGGTCTTTAGTAAAATCTCATACATGTATTCTAGGACTTCAGTTTTCAAAGGATGTTT 22800

Chimpanzee TTAGGTCTTTAGTAAAATCTCATACATGTATTCTAGGACTTCAGTTTTCAAAGGATGTTT 31746

Gorilla TTAGGTCTTTAGTAAAATCTCATACATGTATTCTAGGACTTCAGTTTTCAAAGGATGTTT 22793

************************************************************

Human ATTGTATATCCTGAGTTTGAAAATTAAGTACAGTCTTCAGATGGATTCACTCCACTTTCT 22860

Chimpanzee ATTGTATATCCTGAGTTTGAAAATTAAGTACAGTCTTCAGATGGATTCACTCCACTTTCT 31806

Gorilla ATTGTATATCCTGAGTTTGAAAATTAAGTATAGTCTTCAGATGGATTCACTCCACTTTCT 22853

****************************** *****************************

Human GTTAAAAGTCTATGATAACTTCTTCTAAAATGTGAGGTTAAATATTTTTATTGTCGAACA 22920

Chimpanzee GTTAAAAGTCTATGATAACTTCTTCTAAAATGTGAGGTTAAATATTTTTATTGTCGAACA 31866

Gorilla GTTAAAAGTCTATGATAACTTCTGCTAAAATGTGAGGTTAAATATTTTTATTGTC-AACA 22912

*********************** ******************************* ****

Human ACCATGTCAATAACCATGCGTTTAAAGATTCACGATACATGAGGAAAGGGCTTATGTAAA 22980

Chimpanzee ACCATGTCAATAACCAGGCGTTTAAAGATTCATGATACATGAGGAAAGGGCTTATGTAAA 31926

Gorilla GCCATGTCAATAACCATGCGTTTAAAGATTCACGATACACGAGGAAAGGGCTTATGTAAA 22972

*************** *************** ****** ********************

Human GAATATGCTAAAACAGAAGTCTCTGTAAGTAGTGATGATATTAAGAAATATTTCTGGACA 23040

Chimpanzee GAATATGCTAAAACAGAAGTCTCTGTAAGTAGTGATGATATTAAGAAATATTTCTGGACA 31986

Gorilla GAATATGCCAAAACGGAAGTCTCTGTAAGTAGTGATGATATTAAGAAATATTTCTGGACA 23032

******** ***** *********************************************

Human AAGACTATACAACCAGTCATAATCAGTGATAACCTAGCTAAAGAATATAGTAACATTGTA 23100

Chimpanzee AAGACTGTACAACCAGTCATAATCAGTGATAACCTAGCTAAAGAATATGGTAACATTGTA 32046

Gorilla AAGACTATACAACCAGTCATAATCAGTCATAACCTAGCTAAAGAATATAGTAACATTGTA 23092

****** ******************** ******************** ***********

Human CTTTATCTATACAAACAGGTTTTATTAACCTAATGTATCTTCTTCATGTTTGTTTATAAG 23160

Chimpanzee CTTTATCTCTACAAACAGGTTTTATTAACCTAACATATCTTCTTTATGTTTGTTTATAAG 32106

Gorilla CTTTATCTATACAAACAGGTTTTATTAACCTAATGTATCTTCTTTATGTTTGTTTATAAG 23152

******** ************************ ********* ***************

Human TTAGCAAATTATTAGACTGTCTTCACCTGTTCAACTACATTAAACAGTTTTATTACTATC 23220

Chimpanzee TTAGCAAATTATTAGACTGTCTTCACCTGTTCAACTACATTAAACAGTTTTATCACTATC 32166

Gorilla TTAGCAAATTATTAGACTGTCTTCACCTGTTCAACTACATTAAACAGTTTTATTACTATC 23212

***************************************************** ******

Human CTTGCTGTCATAGTAAATTACCTTGTGTTCACAAGATAACATAACATACATTGTCCTGCT 23280

Chimpanzee CTTGCTGTCATAGTAAATTACCTTGTGTTCACAAGATAACATAACATACATTGTCCTGCT 32226

Gorilla CTTGCTGTCATAGTAAATTACCTTGTGTTCACAAGATAACATAACATACGTTGCCCTGCT 23272

************************************************* *** ******

Human TGTCTTTCTGATGTAGTTTTGCTTGGTGAAAATGCCTAACATTGAGGAATAAATGTGTGG 23340

Chimpanzee TGTCTTTCTGATGTAGTTTTGCTTGGTGAAAATGCCTAAAATTGAGGAATAAATGTATGG 32286

Gorilla TGTCTTTCTGATGGAGTTTTGCTTGGTGAAAATGCCTAAAATTGACGAATAACTGCATGG 23332

************* ************************* ***** ****** ** ***

Human TAGATTTTCTAGTTGGATCTTTGAGGTGGAGTCTCATTCTCTGGACCAGGCTGTAGTGTA 23400

Chimpanzee TAGATTTTCTAGTTGGATCTTTGAGGTGGAGTCTCATTCTCTGGACCAGGCTGTAGTGTA 32346

Gorilla TAGATTTTCTAGTTGGATCTTTGAGGTGGAGTCTCATTCTCTGGACCAGGCTGTAGTGTA 23392

************************************************************

Human GTGGCCTGATCTCGGCTCACTGCAACCTCTGCTTCCTGGGTTCAAGCAATTATCCTTCCT 23460

Chimpanzee GTGGCCTGATCTCGGCTCACTGCAACCTCTGCTTCCTGGGTTCAAGCAATTATCCTTCCT 32406

Gorilla GTGGCCTGATCTCGGCTCACTGCAACCTCTGCTTCCTGGGTTCAAGCAATTATCCTTCCT 23452

************************************************************

Human CAGACTCCCAAGTAGCTGGGATTACAGGTGTGCACCACCACGTCCAGTGAATTTTTGTAT 23520

Chimpanzee CAGACTCCCAAGTAGCTGGGATTACAGGTGTGCACCACCACGTCCAGTGAATTTTTGTGT 32466

Gorilla CAGACTCCCAAGTAGCTGGGATTACAGGTGTGCACCGCCACGTCCAGTGAATTTTTGTAT 23512

************************************ ********************* *

Human TTTAGTAGGGATGGGCTTTCATCATGTTGTCCAGGCTGCTCTCAAACTTGTGACATCAAG 23580

Chimpanzee CTTAGTAGGGATGGGCTTTCATCATGTTGTCCAGGCTGCTCTCAAACTTGTGACATCAAG 32526

Gorilla TTTAGTAGGGATGGG-TTTCATCATGTTGTCCAGGCTGGTCTCAAACTTGTGACATCAAG 23571

************** ********************** *********************

Human TTGTCATTCCACCTCAGTCTCCCAAAGTGCTGAAATTACAGGTGTTACCAACCATGCCCA 23640

Chimpanzee TTGTCATTCCACCTCAGTCTCCCAAAGTGCTGAAATTACAGGTGTTACCAACCATGCCCA 32586

Gorilla TTGTCATCCCACCTCAGTCTCCCAAAGTGCTGAAATTACAGGTGTTACCAACCATGCCCA 23631

******* ****************************************************

Human GCCAACTATAAACATATTTAATGATACACAACACACATATAAATATAAATATTTCTAACG 23700

Chimpanzee GCCAACTATAAACATATTTAATGATACACAACACACATATAAATATAAATATTTCTAACA 32646

Gorilla GCCAACTATAAACATATTTAATGATACACAACACACATATAAATATAAATATTTCTAACA 23691

***********************************************************

Human GAGTATTAGATTGCTTCTTTCTTTAAATTTTGCAGAGACAATTGATAAACGAATCGAAAG 23760

Chimpanzee GAGTATTAGATTGCTTCTTTCTTTAAATTTTGCAGAGACAATTGATAAACCAATCGAATG 32706

Gorilla GAGTATTAGATTGCTTCTTTCTTTAAATTTTGCAGAGACAATTGATAAATGAATCGAAAG 23751

************************************************* ******* *

Human GTGACTCCTACCTTAAAGGGAAGTCAAATAATTTTCTTATAAGAGGTCTAAAATCTTCAG 23820

Chimpanzee GTGACTCCTACCTTAAAGGGAAGTCAAATAATTTTCTTATAAGAGGTCTAAAATCTTCAG 32766

Gorilla GTGACTCCTACCTTAAAGGGAAGTCAAATAATTTTCTTATAAGAGGTCTAAAATCTTCAG 23811

************************************************************

Human ATTGTTTTATTACAATATGCAGCAGTAAAAGCACAAAAAGTTTCATTACTTATTGGCAGC 23880

Chimpanzee ATTGTTTTATTACAATATGCAGCAGTAAAAGCACAAAAAGTTTCATTACTTATTGGCAGC 32826

Gorilla ATTGTTTTATTACAATATGCAGCAATAAAAGCACAAAAAGTTTCATTACTTATTGGCAGC 23871

************************ ***********************************

Human TCGGCCTTACTGTATTTTCCCAGAAACTGTTATAACATTTTTATGTGTTGGGTTTATTAA 23940

Chimpanzee TCGGCCTTACTGTATTTTCCCAGAAACTGTTATAACATTTTTATGTGTTGGGTTTATTAA 32886

Gorilla TCGGCCTTACTCTATTTTCCCAGAAACTGTTATAACATTTTTATGTGTTGGGTTTGTTAA 23931

*********** ******************************************* ****

Human ACACTTCTATGGCATTTAACCTGTAATAAAAT---------------------------- 23972

Chimpanzee ACACTTCTATGGCATTTAACCTGTAATAAAATGTGTAATTTATTGGCCGGGCGCGGTGGC 32946

Gorilla ACACTTCTATGGCATTTAACCTGTAATAAAAT---------------------------- 23963

********************************

Human ------------------------------------------------------------

Chimpanzee TCACGCCTGTAATCCCAGCACTTTGGGAGGCCGAGGCGGGCGGATCACGAGGTCAGGAGA 33006

Gorilla ------------------------------------------------------------

Human ------------------------------------------------------------

Chimpanzee TCGAGACCATCCTGGCTAACACGGTGAAACCCCGTCTCTACTAAAAATACAAAAAAATTA 33066

Gorilla ------------------------------------------------------------

Human ------------------------------------------------------------

Chimpanzee GCCGGGCGTGGTAGCGGGCCCCTGTAGTCCCAGCTACTCGGGAGGCTGAGGCAGGAGAAT 33126

Gorilla ------------------------------------------------------------

Human ------------------------------------------------------------

Chimpanzee GGCGTGAACCCGGGAGGCGGAGCTTGCAGTGAGCCGAGATCGCGCCACTGCACTCCAGCC 33186

Gorilla ------------------------------------------------------------

Human -----------------------------------------------------TTGTAAT 23979

Chimpanzee TGGGCGACAGTGCGAGACTCCGTCTCAAAAAAAAAAAAAAAAAAAAAAAAAATTTGTAAT 33246

Gorilla -----------------------------------------------------TTGTAAT 23970

*******

Human TTATTATAAGGCATATATGTTGTATAATGCTTAAGTGTCAGGAACTACAGAGTAATACTT 24039

Chimpanzee TTATTATAAGGCATATATGTTGTATAATGCTTAAGTGTCAGGAACTACAGAGTAATACTT 33306

Gorilla TTATTATAAGGCATATATGTTGTATAATGCTTAAGTGTCAGGAACTACGGAGTAATACTT 24030

************************************************ ***********

Human CATATACCTTATCTTCCAAAACCATACTTGATTATGCCTAATGCTCTTGGGTCAACAAGA 24099

Chimpanzee CATATACCTTATCTTCCAAAACCATACTTGATTATGCCTAATGCTCTTGGGTCAACAAGA 33366

Gorilla CATATACCTTATCTTCCAAAACCATACTTGATTATGCCTAATGCTCTTGGGTCAACAAGA 24090

************************************************************

Human AGTCTAGAAATAGAATAGTCCCTGATTCTGATCTTTGATAATGAAAGAAAATCCTGCATT 24159

Chimpanzee AGTCTAGAAATAGAATAGTCCCTGATTCTGATCTTTGATAATGAAAGAAAATCTTGCATT 33426

Gorilla AGTCTAGAAATAGAATAGTCCCTGATTCTGATCTTTGATAATGAAAGAAAATCCTGCATT 24150

***************************************************** ******

Human TGACATATATAAAGGAAGAAATAGAAAATCCATCTATTGCCTCTGATCAAGGAGAGTCAA 24219

Chimpanzee TGACATATATAAAGGAAGAAATAGAAAATCCATCTATTGCCTCTGATCAAGGAGGGTCAA 33486

Gorilla TGACATATATAAAGGAAGAAATAGAAAATCCATCTATTGCCTCTGATCAAGGAGGGTCAA 24210

****************************************************** *****

Human GATCTGTGTGAGAAGGAGAAAGGTGTTTGCTGAGCATCCAACATCAGTTGGGGTTGGGGG 24279

Chimpanzee GATCTGTGTGAGAAGGAGAAAGGTGTTTGCTGAGCATCCAACATCGGTTGGGGTTGGGGG 33546

Gorilla GATCTGTGTGAGAAGGAGAAAGGTGTTTGCTGAGCATCCAACATCAGTTGGGGTTGGGGG 24270

********************************************* **************

Human AGGCATTTTCTAACAGTGATGTAGGTGCAAAGTGATCATCTCAATGGCAAGCAGGAAGAC 24339

Chimpanzee AGGCATTTTCTAATGGTGATGTAGGTGCAAAGTGATCATCTCAATGGCAAGCAGGAAGAC 33606

Gorilla AGGCATTTTCTAATGGTGATGTAGGTGCAAAGTGATCATCTCAATGGCAAGCAGGAAGAC 24330

************* *********************************************

Human TATTTTTTTTAGCTGGAAAGAAAGACCAGTGGACTGCCCATCATTGCTATTTTTTGCACA 24399

Chimpanzee TATTTTTTTTAGCTGGAAAGAAAGACCAGTGGACTGCCCATCATTGTTATTTTTTGCACA 33666

Gorilla TATTTTTTTTAGCTGGAAAGAAAGACCAGTGGACTGCCCATCATTGTTATTTTTTGCACA 24390

********************************************** *************

Human GTAAAAATAACTAACAGCTTTATTATTTGTACTTTAACCAGTTTTTATTGTTTTGTTTTG 24459

Chimpanzee GTAAAAATAACTAACAGCTTTATTATTTGTACTTTAACCAGTTTTTATTGTTTTGTTTTG 33726

Gorilla GTAAAAATAACTAACAGCTTTATTATTTGTACTTTAACCAATTTTTATTGTTTTGTTTAG 24450

**************************************** ***************** *

Human TTTTTGAGACAGGGTCTCATTCTATCTGCCAGCCTGGAGTACAGTGCTGCAATCAAAACT 24519

Chimpanzee TTTTTGAGACAGGGTCTCATTCTATCTGCCAGCCTGGAGTACAGCGCTGCAATCAAAACT 33786

Gorilla TTTTTGAGACAGGGTCTCATTCTATCTGCCAGCCTGGAGTACAGTGCTGCAATCAAAACT 24510

******************************************** ***************

Human CACTGCATCCTCAACTGCCTTGGCACCAGTGGTCATCCCATTTCAGCCTCCCAAGTACAG 24579

Chimpanzee CACTGCATCCTCAACTGCCTTGGCTCCAGTGGTCATCCCATCTCAGCCTCCCAAGTACAG 33846

Gorilla CACTGCATCCTCAACTGCCTTGGCTCCAGTGGTCATCCCATCTCAGCCTCCCAAGTACAG 24570

************************ **************** ******************

Human GTGTGCAGCACCATGAGAAGGTAATTTTTAAATTACTTGTAGAGATGGAGTTCTCACTAT 24639

Chimpanzee GTGTGCAGCATCATGAGAAGGTAATTTTTAAATTACTTGTAGAGATGGAGTTCTCACTAT 33906

Gorilla GTGTGCACCACCATGAGAGGGTAATTTTTAAATTACTTGTAGAGATGGAGTTCTCACTGT 24630

******* ** ******* *************************************** *

Human GTTTCCCATGCTGGTCTCAAACCCCTGGGCTCAACCAGTTCTTCCCACCTTGACTTCCAA 24699

Chimpanzee GTTTCCCATGCTGGTCTCAAACCCCTGGGCTTAACCAGTTCTTCCCACCTTGACTTCCAA 33966

Gorilla GTTTCCCATGCTGGTCTCAAACCCCTGGGTTTAACCACTTC-TCCCACCTTGACTTCCAA 24689

***************************** * ***** *** ******************

Human AGTGCTGGAATTACAGGCCTAAACTGTTGCTCCCAAACTTTTTTTTTTCCTTATATGCAG 24759

Chimpanzee AGTGCTGGAATTACAGGCCTAAACTGTTGCTCCCAAAC-TTTTTTTTTCCTTATATGCAG 34025

Gorilla AGTGCTGGAATTACAGGCCTAAACTGTTGCTCTCAAAC-TTTTTTTTTCCTTATATGCAG 24748

******************************** ***** *********************

Human GATATTTATAGATAACTAGTTTTTACTGATTAACACTATCTCATACTAATTTCTAAAAAT 24819

Chimpanzee GATATTTATAGATAACTAGTTTTTACTGATTAACACTATCTCATACTAATTTCTAAAAAT 34085

Gorilla GATATTTATAGATAACTAGTTTTTACTGATTAACACTACCTCATACTAATTTCTAAAAAT 24808

************************************** *********************

Human ACAAATTATATATTATTTTCTATGATAAACAACAATATACACACCAGTACATACACACAC 24879

Chimpanzee ACAAATTATATATTATTTTCTATGATAAACAACAATATACACACCACTACATATACACAC 34145

Gorilla ACAAATTATATATTATTTTCTATGATAAACAACAATGTACACACCAGTACATACACACAC 24868

************************************ ********* ****** ******

Human ATACATATATATAATGCACATCCTATACAGGCAATAATGTGCATATGTTAAACACCCATA 24939

Chimpanzee ATACATATATATAATGCACATCCTATACAGGCAATAATGTGCATATGTTAAACACACATA 34205

Gorilla ATACATATATATAATGCACATCCTATACAGGCAATAATGTGCATATGTTAAACACACATA 24928

******************************************************* ****

Human TATACACATATTTGTGTGTGTGTTTATATATACTCAATACATGCACACTCATACCTGTAT 24999

Chimpanzee TATACACATATTTGTGTGTGTGTTTATATATACTCAATACATGCACACTCATACCTGTAT 34265

Gorilla TATACACATATT--TGTGTGTGTTTATATATACTCAATACATGCACACTCATACCTGTAT 24986

************ **********************************************

Human AGAGTGTGATTTATCTACATTCCTAGAGCACCTGCAGCTCAAACTTTAGAAAACATTTCC 25059

Chimpanzee AGAGTGTGCTTTATCTACATTCCTAGAGCACCTGCAGCTCAAACTTTAGAAAACATTTCC 34325

Gorilla AGAGTGTGCTTTATCTACATTCCTAGAGCACCTGCAGCTCAAACTTTAGAAAACATTTCC 25046

******** ***************************************************

Human TCAATATTCCTCTTTCCTTTGTTGAGTATTCACCACCCAGAAGAGGGAGGGCTTATGGAA 25119

Chimpanzee TCAGTATTCCTCTTTCCTTTGTTGAATATTCACCACCCAGAAGAGGGAGGGCTTATGGAA 34385

Gorilla TCAATATTCCTCTTTCCTTTGTTGAATATTCACCACCCAGAAGAGGGAGGGCTTATGGAA 25106

*** ********************* **********************************

Human ATAACTCATGGCTGTTGGTATAGCAGATTTGAAGTAAATTTTATACTACTGGGAAAAAAT 25179

Chimpanzee ATAACTCATGGCTGTTGGTATAGCAGATTTGAAGTAAATTTTATACTACTGGGAAAAAAT 34445

Gorilla ATAACTCATGGCTGTTGGTATAGCAGATTTGGAGTAAATTTTATACTACTGGGAAAAAAT 25166

******************************* ****************************

Human ATACAAAATCATACATGCACACATG--CACACACACACACACACACACACACACACACAC 25237

Chimpanzee ATACAAAATCATACATGCACACATGTGCACACACACACACACACACACACACACACACAC 34505

Gorilla ATACAAAATCATACACACACACANN--CACAGACACACACACACACACACACACACACAC 25224

*************** ****** **** ****************************

Human ACAGAGCTGGTTGAATTTCACCTGGCACAACTTTGATACATTCTTTTCTTTATCTACTAC 25297

Chimpanzee ACAGAGCTGGTTGAATTTCACCTGGCACAACTTTGATACATTCTTTTCTTTATCTACTAC 34565

Gorilla ACAGAGCTGGTTGAATTTCACCTGGCACAACTTTGATACATTCTTTTCTTTATCTACTAC 25284

************************************************************

Human ATTTATGGACCCATGTGATTACATTGGCACTACCTGGAGAATTCAGGAAAATTTTCCATT 25357

Chimpanzee ATTTATGGACCCATGTGATTACATTGGCACTACCTGGAGAATTCAGGAAAATTTTCCATT 34625

Gorilla ATTTATGGACCCATGTGATTACATTGGCACTACCTGGAGAATTCAGGAAAATTTTCCATT 25344

************************************************************

Human GTCAAGGTCAACAAATAAATACTAAATTCAAAACAGGAAAAGAAAACACACCTATTGAAA 25417

Chimpanzee GTCAAGGTCAACAAATAAATACTAAATTCAAAGCAGAAAAAGAAAACACACCTATTGAAA 34685

Gorilla GTCAAGGTCAACAAATAAATTCTAAATTCAAAACAGAAAAAGAAAACACACCTGTTGAAA 25404

******************** *********** *** **************** ******

Human AATTTCACTAAGCCACCAAATATTCTTAATGGTAAAACAATAATAGATAACATACTTTGA 25477

Chimpanzee AATTTCACTAAGCCACCAAATATTCTTAATGGTAAAACAATAATAGATAACATACTTTGA 34745

Gorilla AATTTCACTAAGCCACCAAATATTCTTAATGGTAAAACAATAATAGATAACATACTTTGA 25464

************************************************************

Human CTGCTTACTGAATTCTATGTGTTATGATAAGCATCTCCAAACATTCTCATTTCTTTCTCA 25537

Chimpanzee CTGCTTACTGAATTCTATGTGTTATGATAAGCATCTCCAAACATTCTCATTTCTTTCTCA 34805

Gorilla CTGCTTACTGAATTCTATGTGTTATGATAAGCATCTCCAAACATTCTCATTTCTTTCTCA 25524

************************************************************

Human TTTCCACCCTATGAACTAGGTATTTATTGTTATCCCATTTCACAGAAGAGGAAACTGGAA 25597

Chimpanzee TTTCCACCCTATGAACTAGGTATTTATTGTTATCCCATTTCACAGAAGAAGAAACTGGAA 34865

Gorilla TTTCCACCCTATGAACTAGGTATTTATTGTTATCCCATTTCACAGAAGAGGAAACTGGAA 25584

************************************************* **********

Human ATGGGGACCTAAAAAAAAACAATATTCATTGGGAAAGATACACTGATTTTCTGGGTTAGG 25657

Chimpanzee ATGGGGACCTAAATAAAAACAATATTCATTGGGAAAGATACACTGATTTTCTGGGTTAGG 34925

Gorilla ATGGGGACCT-AAAAAAANCAATATTCATTGTGAAAGATACACTGATTTTCTGGGTTAGG 25643

********** ** **** ************ ****************************

Human GCTAACTAGTAGGAAAATCAGAGTTTTGTTTTTTTAAAGACTCTTCCCATTTTGGATAAA 25717

Chimpanzee GCTAACTAGTAGGAAAACCAGAGTTTTGTTTTTTTAAAGACTCTTCCCATTTTGGATAAA 34985

Gorilla GCTAACTAGTAGGAAAATCAGAGTTTTTTTTTTTTTAAGACTCTTCCCATTTTGGATAAA 25703

***************** ********* ******* ************************

Human AATAAGATTCAATTTTTCTTTTACATCAGTTTCAGAAGGTTTATTTGATGATTAAACATG 25777

Chimpanzee AATAAGATTCAATTTTTCTTTTACATCAGTTTCAGAAGGTTTATTTGATGATTAAACATG 35045

Gorilla AATAAGATTCAATTTTTCTTTTACATCAGTTTCAGAAGGTGTATTTGATGATTAAACATG 25763

**************************************** *******************

Human GTAATAATAATTAACAACCTTAGAATAATTCTTGGTTTTTCATAGTTACGGTCTATCTCT 25837

Chimpanzee GTAATAATAATTAACAACCTTAGAATAATTCTTGGTTTTTCATAGTTGCGGTCTATCTCT 35105

Gorilla GTAATAATGATTAACAACCTTAGAATAATTCTTGGTTTTTCATAGTTACTGTCTATCTCT 25823

******** ************************************** * **********

Human CTTATTAGTTTTAGTTACTGCTCACCTTCTCTTATTAATATCAAGATATGTTAATGCAAA 25897

Chimpanzee CTTATTAGTTTTAGTTACTGCTCATCTTCTCTTATTAATATCAAGATATGTTAATGCGAA 35165

Gorilla CTTATTTATTTTAGTTAATGCTCATATTCTGTTATTAATATCAAGATATGTTAATGTGAA 25883

****** ********* ****** **** ************************* **

Human GAGAACTAGCCTAATAGAAAGTCTGTACTCTGAAAAAATCCTAGGTCTAAAGCATATGCA 25957

Chimpanzee GAGAACTAGCCTAATAGAAAGTCTGTACTCTGAAAAAATCCTAGGTCTAAAGCATATGCA 35225

Gorilla GAGAACTAGCCTAATAGAAAGTCTGTACTCTGAAAAAATCCTAGGTCTAAAGCATATGCA 25943

************************************************************

Human TTTTAAAATAGCAACAAAATGAATCTTCTTGCCCATTGCAACAGATTGCTTCTAGTATTG 26017

Chimpanzee TTTTAAAATAGCAACAAAATGAATCTTCTTGCCCATTGCAACAGATTGCTTCTAGTATTG 35285

Gorilla TTTTAAAATAGCAACAAAATGAATCTTCTTGCCCATTGCAACAGATTGCTTCTAGTATNN 26003

**********************************************************

Human TATTTGAAGAGAATGAATTAGTTATTTCAGTCTAAATATATACAATGTATACATAAATAT 26077

Chimpanzee TATTTGAAGAGAATGAATTAGTTATTTCAGTCTAAATATATACAATGTATACATAAATAT 35345

Gorilla NATTTGAAGAGAATGAATTAGTTATTTCAGTCTAAATATATACAATGTATACATAAATAT 26063

***********************************************************

Human ATATATATATATATATATATATATATATGTCTAGGCGATTATGTATGTTATTATTTTCTG 26137

Chimpanzee ATATATATATATAT--------------CTCTAGGTGATAATGTATGTTATTATTTTCTG 35391

Gorilla ATATACATATATATATACNNNNNNNNNNNNCTAGGTGATAATGTATGTTATTATTTTCTG 26123

***** ******** ***** *** ********************

Human TCAACTTCATGGAAACTAATCCAGTCTAAGATTTTTTCATGGGCTAATTTGTTAAAAATT 26197

Chimpanzee TGAACTTCATGGAAACTAATCCAGTCTAAGATTTTTTTATGGGCTAATTTGTTAAAAATT 35451

Gorilla TCAACTTCATGGAAACTAATCCAGTCTAAGATTTTTTTGTGGGCTAATTTGTTAAAAATT 26183

* *********************************** *********************

Human GCATTTATCTCATAAGATACCAAAAGAGAGTAAGTCAATTAGAAATCTACATGATTTTAA 26257

Chimpanzee GCATTTATCTCATAAGATACCAAAAGAGAGTAAGTCAATTAGAAATCTACATAATTTTAA 35511

Gorilla GCATTTATCTCATAAGATACAAAAAGAGAGTAAGTCAATTAGAAATCTACATAATTTTAA 26243

******************** ******************************* *******

Human AGTCATATTATTGTGAGAGGCTTTAAAACCTTAAAATATTTTTAAAAGCAAACAATAGAG 26317

Chimpanzee AGTCATATTATTGTGAGAGGCTTTAAAACCTTAAAATATTTTTAAAAGCAAACAATAGAG 35571

Gorilla AGTCATATTATTGTGAGAGACTTTAAAACCTTAAAATATTTTTAAAAGCAAACAATAGAG 26303

******************* ****************************************

Human AACATATAATTGTATAACATGCTAAGAGACTACTCTTCCTGTTTACCCTAACAATAATTT 26377

Chimpanzee AACATATAATTGTGTAACATGCTAAGAGACTACTCTTCCTGTTTACCCTAACAATAATTT 35631

Gorilla AACATATAATTGTATAACATGCTAAGAGACTACTCTTCCTGTTTACCCTAACAATAATTT 26363

************* **********************************************

Human TCTGCCTATCTTCTATACTATTCCTATTGTTAAGAAACCCAGTAAGTTTTTTACAGTGAC 26437

Chimpanzee TCTGCCTATCTTCTATACTATTCCTATTGTTAAGAAACCAAGCAAGTTTTTTACAGTGAC 35691

Gorilla TCTGCCTGTCTTCCATACTATTCCTATTGTTAAGAAACCCAGTAAGTTTTTTACAGTGAC 26423

******* ***** ************************* ** *****************

Human TTTCTTCCTTTTTTTTTTTTTTTTTTTTTTTTTTTTTTTGATTCAGAGTCTCACTGTTTC 26497

Chimpanzee TTTCTTC------TTTTTTTTTTTTTTTTTTTTTTTTTTGATTCAGAGTCTCACTGTTTC 35745

Gorilla TTTCTTCCTTTTTTTTTTTTTTTTTTTTTTTTTTTTTTTGACTCAGAGTCTCACTGTTTC 26483

******* **************************** ******************

Human ACCCAGGCTTTAGTGCAGTGGTATTAATACAATCATAGATTACTGTAGCTTCAACCTCTT 26557

Chimpanzee ACCCAGGCTTTAGTGCAGTGGTATTAATACAATCATAGATTACTGTAGCTTCAACCTCTT 35805

Gorilla ACCCAGGCTTTAGTGCAGTGGTATTAATACAATCATAGATTACTGCAGCTTCAACCTCTT 26543

********************************************* **************

Human GGGCTCAAGTAATCCTTCTACTCAGCCTCTTGAGTAGCTGGGCCTATAGGCATGTGCCAC 26617

Chimpanzee GGGCTCAAGTGATCCTTCTACTCAGCCTCTTGAGTAGCTGGGCCTATAGGCATGTGCCAC 35865

Gorilla GGGCTCAAGTGATCCTTCTACTCAGCCTCTTGAGTAGCTGGGCCTATAGGCATGTGCCAC 26603

********** *************************************************

Human CATATCTGACTAACTTTTAAATTTTTTGTAGAGACAAGATCTTGCTGTGTTGCTGAGACT 26677

Chimpanzee CACATCTGACTAACTTTTAAATTTTTTGTAGAGACAAGATCTTGCTGTGTTGCTGAGACT 35925

Gorilla CACACCTGGCTAACTTTTAAATTTTTTGTAGAGACAAGATCTTGCTGTGTTGCTGAGACT 26663

** * *** ***************************************************

Human CATCTTAAATTCCCTACCTCAAGGGATTCTCTCATCTCAGCCACCCAAAGCTCTGGCATC 26737

Chimpanzee CATCTTAAATTCCCTACCTCAAGGGATTCTCTCATCTCAGCCACTCAAAGCTCTGGGATC 35985

Gorilla CATCTTAAATTCCCTACCTCAAGGGATTCTCTCATCTCAGCCACCCAAAGCTCTGGGATC 26723

******************************************** *********** ***

Human TCTGGTGTGAGTCACTGCACTCGGCCTCCCTTCCACATTTAAAGCTAGCAGAGCCTGGTC 26797

Chimpanzee TCTGGTGTGAGTCACTGCACTCGGCCTCCCTTCCACATTTAAAGCTAGCAGAGCCTGGTC 36045

Gorilla TCTGGTGTGAGTCACTGCACTCGGCCTCCCTTCAACATTCAAAGCTAGCAGAGCCTGGTC 26783

********************************* ***** ********************

Human AAATCTTTCTCATGAAAGACCCTATCTATGGTTTTAACTCCTCTGCCTCCCTGTTCCTGT 26857

Chimpanzee AAGTCTTTCTCATGAAAGACCCTATCTATGGTTTTAACTCCTCTGCCTCCCTGTTCCTGT 36105

Gorilla AAGTCTTTCTCATGAAAGACCCTATCTACGGTTTTAACTCCTCTGCCTCCCTGTTCCTGT 26843

** ************************* *******************************

Human TATGTAACATCAACAGAGACATATTGCATTGATGGTTTTGTGCTTTACAATTTTCATTTA 26917

Chimpanzee TATGTAACATCAACAGAGACATATTGCATTGATGGTTTTGTGCTTTACAATTTTCATTTA 36165

Gorilla TATGTAACATCAACAGAGACATATTGCATTGATGGTTTTGTGCTTTACAATTTTCATTTA 26903

************************************************************

Human GTGACGTTTGTCTTACTATTAAGGATTTATTAATATTAAGCAAGGCTTGTTCTGATTTTC 26977

Chimpanzee GTGATGTTTGTCTTACTATTAAGGATTTATTAATATTAAGCAAGGCTTGTTCTGATTTTC 36225

Gorilla GTGATGTTTGTCTTACTATTAAGGATTTATTAATATTAAGCAAGGCTTGTTCTGATTTTC 26963

**** *******************************************************

Human TTTACATGTATTTTCATAGAGGTGTTGACCTCTCTTAAATTAAAAACTCTTCTGATTAGA 27037

Chimpanzee TTTACATGTATTTTCATAGAGGTGTTGACCTCTCTTAAATTAAAAACTCTTCCAATTAGA 36285

Gorilla TTTACATGTATTTTCATAGAGGTGTTGACCTCTCTTAAATTAAAAA--CTTCTCATTAGC 27021

********************************************** **** *****

Human TTTGAGTGTATCTATTGACTGTATAAGTTTACATTTCATTTATTGATATGGTGTCAGAAT 27097

Chimpanzee TTTGAGTGTATGTATTGACTGTATAAGTTTACATTTCATTTATTGATATGGTGTCAGAAT 36345

Gorilla TTTGAGTGTATCTATTGACTGTATAAGTTTACATTTCATTTATTGATATGGTGTCAGAAT 27081

*********** ************************************************

Human TCATAAAATGTTAATCCAGCATTTGATTGTGATAATTTTTTATGATATCTCTTGTTATCA 27157

Chimpanzee TCATAAAATGTTAATCCAGCATTTGATTGTGATAATTTTTTATGATATCTCTTGTTATCA 36405

Gorilla TCATAAAATGTTAATCCAGCATTTGATTGTGATAATTTTTTATGATATCTCTTGTTATCA 27141

************************************************************

Human CATGTCTTTCAATGGGCAGCATGTACATTATTTTAGTACTTTACCTCCCTTATTTGATGC 27217

Chimpanzee CATGTCTTTCAATGGGCAGCATGTACATTATTTTAGTACTTTACCTCCCTTATTTGATGC 36465

Gorilla CATGTCTTTCAATGGGCAGCATGTACATTATTTTAGTACTTTACCTCCCTTATTTTATGC 27201

******************************************************* ****

Human ACCCTTTTGGTGTATGAATGATGTGGTAGTGTTTCATGCAACTATTTCTCTTTTCATAAC 27277

Chimpanzee ACCCTTTTGGTGGATGAATGATGTGGTAGTGGTTCATGCAACTATTTCTCTTTTTGTAAC 36525

Gorilla ACCCTTTTGGTGTATGAATGATGTGGTGGTGGTTCATGCAACTATTTATCTTTTTGTAAC 27261

************ ************** *** *************** ****** ****

Human AAAACAGTCGAGAGTAAGGCTTTGGCTCCTGAGGCGCAGTATTTCTCAAGATAAGGGAAA 27337

Chimpanzee AAAACAGTCGAGAGTAAGGCTTTGGTTCCTCAGGTGCAGTATTTCTCAAGATAAGGGAAA 36585

Gorilla AAAACAGTCGAGAGTAAGGCTTTGGTTCCTCAGGTGCAGTATTTCTCAAGATAAGGGAAA 27321

************************* **** *** *************************

Human AGACCGTTGCAATAAACCAGCTGAGTGATTGAAAAATATGATTTCCCTTTCAGATAACCA 27397

Chimpanzee AGACCATTGCAATAAACCAGCTGAGTGATTGAAAAATATGATTTCCCTTTCAGATAACCA 36645

Gorilla -GACCATTGCAATAAACCAGCTGAGTGATTGAAAAATATGATTTCCCTTTCAGATAACCA 27380

**** ******************************************************

Human CTTAACTAGACTCTCTTAAGGACAGCATTTTGGAATATGTATTTCATAAGAACTCATGTA 27457

Chimpanzee CTTAACTAGACTCTCTTAAGGACAGCATTTTGGAATATGTATTTCATAAGAACTCATGTA 36705

Gorilla CTTAACTAGACTCTCTTAAGGACAGCGTTTTGGAATATGTATTTCATAAGAACTCATGTA 27440

************************** *********************************

Human ATTTTAATACACTGTAATGTCTAAATAACACCCTTCCAGGCATTACCTATTCTGAAACAC 27517

Chimpanzee ATTTTAATACACTGTAATGTCTAAATAACACCCTTCCAGGCATTACCTATTCTGAAACAC 36765

Gorilla ATTTTAATACACTGTAATGTCTAAATAACACCCTTCCAGGCATTACCTGTTCTTAAACAC 27500

************************************************ **** ******

Human TATTTGCCCATTTTCACCATTAGAATATCTTGTACACCTACTGGGGAAACAAACAAACAA 27577

Chimpanzee TATTTGCCCATTTTCACCATTAGAATATCTTGTACACCTACCGGGGAAACAAACAAACAA 36825

Gorilla TATTTGCCCATTTTCACCATTAGAATATCTTGTACACCTACTGGGGAAACAAACAAACAA 27560

***************************************** ******************

Human ACAAAAACTATGAAACTAGCAAGTGTTAAATCTTCCATCTTTCTCAATTGACATCAAAAT 27637

Chimpanzee ACAAAAACTATGAAACTAGCAAGTGTTAAATCTTCCATCTTTCTCAATTGACATCAAAAT 36885

Gorilla ACAAAAACTATGAAACTAGCAAGTGTTAAATCTTCCATCTTTCTCAATTGACATCAAAAT 27620

************************************************************

Human ATTTAAAGGTGCATTATGGGTACTTTCAGTTTTGTAACTGAAGTTTAAAAATTGTGGACA 27697

Chimpanzee ATTTAAAGGTGCATTATAGGTACTTTCAGTTTTGTAACTGAAGTTTAAAAATTGTGGACA 36945

Gorilla ATTTAAAGGTGCATTATAGGTACTTTCAGTTTTGTAACTGAAGTTTAAAAATTGTGGACA 27680

***************** ******************************************

Human GATGGCTATTTATTTCAGTGGATTCAAAGTTCACTGAGAATCGTTTATAGACTTGTTTTC 27757

Chimpanzee GATGGCTATTTATTTCAGTGGATTCAAAGTTCACTGAGAATCGTTTATAGACTTGTTTTC 37005

Gorilla GATGGCTATTTATTTCAGTGGATTTAAAGTTCACTGAGAATCGTTTACAGACTTGTTTTC 27740

************************ ********************** ************

Human ATACATGATGAGATATTTACTTCACTTTCTTTCTTTGGCTGGCTGGAGTTTTATTTTTCC 27817

Chimpanzee ATACATGATGAGATATTTACTTCACTTTCTTTCTTTGGCTGGCTGGAGTTTTATTTTTCC 37065

Gorilla ATACACGATGAAATATTTACTTCACTTTCTTTCTTTGGCTGGCTGGAGTTTTATTTTTCC 27800

***** ***** ************************************************

Human AAAAATTGTCTCAATTATTTGTTTGCCATTTGGGTTTTGATTAGATGATTTATAAAGAGG 27877

Chimpanzee AAAAATTGTCTCAATTATTTGTTTGCCATTTGGGTTTTGATTAGATGATTTATAAAGAGG 37125

Gorilla AAAAATTGTCTCAATTATTTGTTTGCCATTTGTGTTTTGATTAGATGATTTATAAAGAGG 27860

******************************** ***************************

Human TGGTGACCTCTATGATTCCAGTCTTCTTACTTTTCATAATGACTGCTTTCTTCTGATTTA 27937

Chimpanzee TGGTGACCTCTATGATTCCAGTCTTCTTACTTTTCATAATGACTGCTTTCTTCTGATTTA 37185

Gorilla TGGTGACCTCTATGATTCCAGTCTTCTTGCTTTTCATAATGACTGCTTTCTTCTGATTTA 27920

**************************** *******************************

Human AGTAGGAAGTAATCGAGGTATCCAAATTTGAGAATCTAAATGTATTTACTATGTAAAAAT 27997

Chimpanzee AGTAGGAAGTAATCGAGGTATCCAAATTTGAGAATCTAAATGTATTTACTATGTAAAAAT 37245

Gorilla AGTAGGAAGTAATTGAGGTATCCAAATTTGAGAATCTAAATGTATTTACTATGTAAAAAT 27980

************* **********************************************

Human TGTGTTAATCTATTCTCTCAGTTAGTGTAGCTGTGGAGATGTCTGATGGAAATGTTATCA 28057

Chimpanzee TGTGTTAATCTATTCTCCCAGTTAGTGTAGCTGTGGAGATGTCTGAAGGAAACGTTATCA 37305

Gorilla TGTGTTAATCTATTCTCTCAGTTAGTGTAGCTGTGGAGATGTCTGAAGGAAATGTTATCA 28040

***************** **************************** ***** *******

Human TTTTCTGCCAGTAAATTGCTTGCACTCTGTACCTTATTCACATATATATACACATATATA 28117

Chimpanzee TTTTCTGCCAGTAAATTGCTTGCACTCTGAACCTTATTCACATATATATATATATATATA 37365

Gorilla TTTTCTGCCAGGAAATTGCTTGCACCCTGTACCTTATTCACATATATATANATATATATA 28100

*********** ************* *** ******************** * *******

Human ------TATATATGCATAATATATCATATAGATTATATGAGTGTATGTGTCAATAATATA 28171

Chimpanzee CACATATATATATGCATAATATATCATATAGATTATATGAGTGTATGTGTCAATAACATA 37425

Gorilla ------TATATATGCATAATATATCATATAGATTATATGAGTGTATGCGTCAATAATATA 28154

***************************************** ******** ***

Human TGGTTACTCTTTACTGTGGATTGAAATTGAGCTCTATTTTTCCTTGGGAAATAATGTATA 28231

Chimpanzee TGGTTACTCTTTACTGTGGATTGAAATTGAGCTCTATTTTTCCTTGGGAAATAATGTATA 37485

Gorilla TCGTTANNNNNNNNNNNNNNNNNNNNTTGAGCTCTATTTTTCCCTGGGAAATANNNTATA 28214

* **** ***************** ********* ****

Human ATTTTGTATCTGTACTTTGGAAAATTATTTGATTTTCTTTCATGATGTAATTGCCCAATA 28291

Chimpanzee ATTTTGTATCTGTACTTTGGAAAATTATTTGATTTTCTTTCATGATGTAATTGCCCAATA 37545

Gorilla ATTTTGTATCTNNNNNNNNNNNNNNNNNNNNNNNNNNNNNNNNNNNNNNNNNNNNNNNNN 28274

***********

Human GATTCTTCTTGCCTGCTACATAGATAGAGCCAATTCACTGATACAGCAGAAGTGGAAGGG 28351

Chimpanzee GATTCTTCTTGCCTGCTACATAGACAGAGCCAATTCACTGATACAGCAGAAGTGGAAGGG 37605

Gorilla NNNNNNNNNNNNNNNCTGCATAGATAGAGCCAATTCACTGATACAGCAGAAGTGGAAGGG 28334

** ****** ***********************************

Human TTTAATCAATGCAGAGCTAGGCACAGGGAAGACATTAGTTTATTATTCAAATCAGCCTTG 28411

Chimpanzee TTTAATCAATGCAGAGCTAGGCACAGGGAAGACATTAGTTTATTATTAAAATCAGCCTTG 37665

Gorilla TTTAATCAATGCATAGCTAGGCACAGGGAAGACATTAGTTTATTATTCAAATCAGCCTTG 28394

************* ********************************* ************

Human CTGGACATTCAGAGACTAGGTTTTTAAAATAATAATTAGGTGGGCAGGTGGCTAGGAAAT 28471

Chimpanzee CTGGACATTCAGAGACTAGGTTTTTAAAATAATAATTAGGTGGGCAGGTGGCTAGGAAAT 37725

Gorilla CTGGACATTCAGAGACCAGGTTTNNNNANNNATAATTAGGTGGGCAGGTGGCTAGGAAAT 28454

**************** ****** * *****************************

Human GGGGAATACTGACTGCTCAGGTTGGGGTTGAAATGATAGGGAATTTTGGCTTTCTTCTTG 28531

Chimpanzee GGGGAATACTGACTGCTCAGGTTGGGGTTGAAATGATAGGGAATTTTGGCTTTCTTCTTG 37785

Gorilla GGGGAATACTGACTGCTCAGGTTGGGGTTGAAATGATAGGGAATTTTGGCTTTCTTCTTG 28514

************************************************************

Human CTGTCTCCAGTTTCTGTGTGGAAGTCACAAGACTAGTTTGAGCCGGTTTCTTGGTATGGG 28591

Chimpanzee CTGTCTCCAGTTTCTGTGTGGGAGTCACAAGACTAGTTTGAGCCAGTTTCTTGGTATGGG 37845

Gorilla CTGTCTCCAGTTTCTGTGTGGNNGTCACAAGACTAGTTTGAGCCGGTTTCTTGGTATGGG 28574

********************* ********************* ***************

Human TGATGGATCTGACTGGCACCAGCTTTTTCACCAGAATGCAGGGTCTGAAAAATACGTCCA 28651

Chimpanzee TGATGGATCTGACTGGCACCAGCTTGTTCACCAGAATGCAGGGTCTGAAAAATACGT-CA 37904

Gorilla TGATGGATCTGACTGGCACCAGCTTGTTCACCAGAATGCAGGGTCTGAAAAATAAGT-CA 28633

************************* **************************** ** **

Human CTCACCAATCTTAGGTTTTACAATAGTAGTTTCATCTATAGGAGGAACTAGGGAGATTGG 28711

Chimpanzee CTCACCAATCTTAGGTTTTACAATAGTAGTTTCATCTATAGGAGGAACTAGGGAGATTGG 37964

Gorilla CTCACCAATCTTAGGTTTTACAATAGTAGTTNNNNNNNNNNGAGGAACTAGGGNNNNNNN 28693

******************************* ************

Human GAATCTTGTGGCCTCTGGCTACTTGGCTCCTGAGACATAATTTCCAATCTTGTAGTTAAC 28771

Chimpanzee GAATCTTGTGGCCTCTGGCTACTTGGCTCCTGAGACATAATTTCCAATCTTGTAGTTAAC 38024

Gorilla NNANCTTGTGGCCTCTGGCTACTTGGCTCCTGAGACATAATTTCCAATCTTGTAGTTAAC 28753

* ********************************************************

Human TTGTTAGTTTTCCAAAGACAGTGTTAATATATTTATTTTTT-AATTAAACTATAAACTAA 28830

Chimpanzee TTGTCAGTTTTCCAAAGACAGTGTTAATATATTTATTTTTTTAATTAAACTATAAACTAA 38084

Gorilla TTGTTAGTTTTCCAAAGACAGTNNTNNNNNNNNNNNNNNNN-NNNNNNNNNNNNNNNNNN 28812

**** ***************** *

Human ATTCAATAGATTCGTAGACATATACTTATCTTAAAACACATCACAGTGTGTATAATACAT 28890

Chimpanzee ATTCAATAGATTCATAGACATATACTTATCTTAAAACATATCACAGTGTGTATAATACAT 38144

Gorilla NNNNNNNNNNNNNNNNNACNNNNNNNNNNNNNNAAACNNNNNNNNNNNNNNNNNNNNNNN 28872

** ****

Human ATGTACAGCTTTTTGTATGACAACCATACTTCAATAAACTGGTTTTAGAAAAAGAACCAT 28950

Chimpanzee ATGTACAGCTTTTTGTATGACAACCATACTTCAATAAACTGGTTTTAGAAAAAGAACCAT 38204

Gorilla NNNNNNNNNNNNNNNNNNNNNNNNNNNNNNNNNNNNNNNNNNNNNNNNNNNNNNNNNNNN 28932

Human TTTTAGGGTAAAATACTTTAATTTCTTTCAATATTTATGTAATAAATATTAACATTTATT 29010

Chimpanzee TTATAGGGTAAAATACTTTAATTTCTTTCAATATTTATGTAATAAATATTAACATTTATT 38264

Gorilla NNNNNNNNNNNNNNNNNNNNNNNNNNNNNNNNNNNNNNNNNNNNNNNNNNNNNNNNNNNN 28992

Human TTAATATTTTGATATTTATATTTAAATATACTTAATAATTTACTATTATTATGTAACAAT 29070

Chimpanzee TTAATATTTTGATATTTATATTTAAATATACTTAATAATAATTTACTATTATGTAATAAT 38324

Gorilla NNNNNNNNNNNNNNNNNNNNNNNNNNNNNNNNNNNNNNNNNNNNNNNNNNNNNNNNNNNN 29052

Human AACATAACTATTGAAATAATCAAAATATTTTACCCCAAAATGTTGTCAACCAAGAGCAAT 29130

Chimpanzee AACATAACTATTGAAATAATCAAAATATTTTACCCCAAAATGTTGTCAACCAAGAGCAAT 38384

Gorilla NNNNNNNNNNNNNNNNNNNNNNNNNNNNNNNNNNNNNNNNNNNTGTCAACCAAGAGCAAT 29112

*****************

Human AAAAAAATGTCAGAATCTAGTCTCAAGATAGTTTATTCAAGTCTGAGGATGGATGACATT 29190

Chimpanzee AAAAAAATGTCAGAATCTAGTCTCAAGATAGTTTATTCAAGTCTGAGGATGGATGACATT 38444

Gorilla AAAAACAAGTCAGAATCTANNNNNNNNNNNNNNNNNNNNNNNNNNNNNNNNNNNNNNNNN 29172

***** * ***********

Human CCAATCAAAGATTCTGAAGAAGAGAAATCAGGGTTCCAAAGGGTAACTGTTTTGAATTCC 29250

Chimpanzee CCAATCAAAGATTCTGAAGAAGAGAAATCCGGGTTCCAAAGGGTAGATGTTTTGAATTCC 38504

Gorilla NNNNNNNNNNNNNNNNNNNNNNNNNNNNNNNNNNNNNNNNNNNNNNNNNNNNNNNNNNNN 29232

Human TTATGTGGACAAAGTTTAGAAAAGTGTGACAGAATTTCATCATCTTTCTCTGTATCGCTT 29310

Chimpanzee TTATGTGGACAAAGTTTAGAAAAGTGTGACAGAATTTCATCATCTTTCTCTGTATCGCTT 38564

Gorilla NNNNNNNNNNNNNNNNNNNNNNNNNNNNNNNNNNNNNNNNNNNNNNNNNNNNNNNNNNNN 29292

Human GATGCATGGTTAAAACAATCTGGTTACTTAAAGTGGTGTTCTTCTTTTGGGAAAGGTGTA 29370

Chimpanzee GATGCATGGTTAAAACAATCTGGTTACTTAAAGTGGTGTTCTTCTTTTGGGAAAGGTGTA 38624

Gorilla NNNNNNNNNNNNNNNNNNNNNNNNNNCTTAAAGTGNNNNTTTTCTNNNNNNNNNNNNNNN 29352

********* * ****

Human TGCAACATTCCCAACACTATGTAAAAATTGTGTTAATCTATTCTCTCAGTGTAGCTGTGG 29430

Chimpanzee TGCAACATTCCA------------------------------------------------ 38636

Gorilla NNNNNNNNNNNNNNNNNNNNNNNNNNNNNNNNNNNNNNNNNNNNNNNNNNNNNNNNNNNN 29412

Human AGATGTCTGAAGGAAATGTTATCATTTTCTGCCAGTAAATTGCTTGCACTCTGTACCTTA 29490

Chimpanzee ------------------------------------------------------------

Gorilla NNNNNNNNNNNNNNNNNNNNNNNNNNNNNNNNNNNNNNNNNNNNNNNNNNNNNNNNNNNN 29472

Human TTCACATATATGTACACATATATATATGCATAATATATCATATAGATTATATGAGTGTAT 29550

Chimpanzee ------------------------------------------------------------

Gorilla NNNNNNNNNNNNNNNNNNNNNNNNNNNNNNNNNNNNNNNNNNNNNNNNNNNNNNNNNNNN 29532

Human GTGTCAATAATATATGGTTACTCTTTACTGTGGATTGAAATTGAGCTCTATTTTTCCTTG 29610

Chimpanzee ------------------------------------------------------------

Gorilla NNNNNNNNNNNNNNNNNNNNNNNNNNNNNNNNNNNNNNNNNNNNNNNNNNNNNNNNNNNN 29592

Human GGAAATAATGTATAATTTTGTATCTGTACTTTGGAAAATTATTTGATTTTCTTTCATGAT 29670

Chimpanzee ------------------------------------------------------------

Gorilla NNNNNNNNNNNNNNNNNNNNNNNNNNNNNNNNNNNNNNNNNNNNNNNNNNNNNNNNNNNN 29652

Human GTAATTGCCCAATAGATTCTTCTTGCCTGCTACATAGATAGAGCCAATTCACTGAAGATG 29730

Chimpanzee -------------------------------------------------CACTGAAGATG 38647

Gorilla NNNNNNNNNNNNNNNNNNNNNNNNNNNNNNNNNNNNNNNNNNNNNNNNNCACTGAAGATG 29712

***********

Human TAACGGACATCTTGGGCACCATCTAGTCTGAGCTAGGTACAGGATGATAAAGAAGGCAGT 29790

Chimpanzee TAACTGACATCTTGGGCACCATCTAGTCTGAGCTAGGTACAGGATGATAAAGAAGGCAGT 38707

Gorilla TAACTGACATCTTGGGCACCACCTAGTCTGAGCTAGGTACAGGATGATAAAGAAGGCAGT 29772

**** **************** **************************************

Human TAGTCTGTAAGATCGATCAATGATTGTTAGGGGGAAGTCTAGTTTCTGGTCTCTGGTAGG 29850

Chimpanzee TAGTCTGTAAGATCGATCAAGGATTGTTAGGGGGAAGTCTAGTTTCTGGTCTCTGGTAGG 38767

Gorilla TAGTCTGTAAGATCGATCAATGATTGTTAGGGGGAAGTCTAGTTTCTGGTCTCTGGTAGG 29832

******************** ***************************************

Human AATTTACAGAACAACATAAATACGGAAGATAGTTAATCTACAATCTAAAAAGCAAAATTG 29910

Chimpanzee AATTTACAGAACAACATCAATACGGAAGATAGTTAATCTACAATCTAAAAAGCAAAATTG 38827

Gorilla AATTTACAGAACAACATCAGTACGGAAGATAGTTAATCTACAATCTAAAAAGCAAAATTG 29892

***************** * ****************************************

Human CAGACATACTCTGTGTCTAAGTATCCAGGATTTAACTTTTCCCTTGGCATCATGAACATA 29970

Chimpanzee CAGACATACTATGTGTCTAAGTATCCAGGATTTAACTTTTCCCTTGGCATCATGAACATA 38887

Gorilla CANNNNNNNNNNNNNTCNNNNNNTCCAGGATTTAACTTTTCCCTTGGCATCATGAACATA 29952

** ** *************************************

Human GAGGGTTCTGAAATTTTGTTTTCTTTTACAATGTATTTCGTTTGCATATTTCTGCATGGG 30030

Chimpanzee GAGGGTTCTGAAATTTTGTTTTCTTTTACAATGTATTTCGTTTGCATATTTCTGTATGGG 38947

Gorilla GAGGGTTCTGAAATTTTATTTTCTTTTACAATGTATTTCGTTTGCATATTTCTGTATGGG 30012

***************** ************************************ *****

Human TAGTCAGAAGTGCAGTAAATACAAGAATTGTTCAAAAGCTGTCTTTTGTGGGGGAGATTT 30090

Chimpanzee TAGTCAGAAGTGCAGTAAATACAAGAATTGTTCAAAAGCTGTCTTTTGTGGGGGAGATTT 39007

Gorilla TAGTCAGAAGTGCAGTAAATACAAGAATTGTTCAAAAGCTGTCTTTTGTGGGGGAGATTT 30072

************************************************************

Human GCATCTGTAGAAAAAATCTGAGGCCAAATGCAGTGACTCATACCTGTATTCCCTGTACTT 30150

Chimpanzee GCATCTGTAGAAAAAATCTGAGGCCAGATGCAGTGACTCATACCTGTAATCCCTGTACTT 39067

Gorilla GCATCTGTAGAAAAAATCTGAGGCCAGATGCAGTGACTCATACCTGTAATCCCTGTACTT 30132

************************** ********************* ***********

Human TGGGAGGCCCAAGCAGGCAGATCACTTGAAGTCTGGAGTTTGAGACCAGCCTTCCCAACA 30210

Chimpanzee TGGGAGGCCCAAGCAGGCAGATCACTTGAAGTCTGGAGTTTGAGACCAGCCTTCCCAACA 39127

Gorilla TGGGAGGCCCAAGCAGGCAGATCACTTGAAGTCTGGAGTTTGAGACCAGCCTTCCCAACA 30192

************************************************************

Human TGGTGATAGCTTGTCTCTACTAAAAATACAAAAATTAGCTGGGCATGGTGGCAGTGCCAG 30270

Chimpanzee TGGTGATAGCTTTTCTCTACTAAAAATACAAAAATTAGCTGGGCATGGTGGCAGTGCCAG 39187

Gorilla TGGTGATAGCTTGTCTCTACTAAAAATACAAAAATTAGGTGGGCATGGTGGCAGTGCCAG 30252

************ ************************* *********************

Human TAATTCCAGCTAATCAGGAGGCTGAAGCAAGATAATTGCTTTAACCCAAGAGGCAGAGAT 30330

Chimpanzee TAATTCCAGCTAATCAGGAGGCTGAAGCAAGATAATTGCTTTAACCCAAGAGACAGAGAT 39247

Gorilla TAATTCCAGCTAATCAGGAGGCTGAAGCAAGATAATTGCTTTAACCCAAGAGGCAGAGAT 30312

**************************************************** *******

Human TGCAGTGAGCCAAGACTGCACCACCGCACTCCAGCTTGGGGGACAGAGTGAGACTCTGTC 30390

Chimpanzee TGCAGTGAGCCAAGACTGCACCACTGCACTCCAGCTTGGGGGACAGAGTGAGACTCTGTC 39307

Gorilla TGCAGTGAGCCAAGACTGNNNCACTNNACTCCAGCTTGGGGGACAGAGTGAGACTCTGTC 30372

****************** *** *********************************

Human TCAAAAACAACACCACCAACAACAGCAACAACAACAAAAACCAAAAGAAAAATCTGCATT 30450

Chimpanzee TCAAAAACAACCACAACAACAACAGCAACAACAACAAAAACCAAAAGAAAAATCTGCATT 39367

Gorilla TCAAAAACANCAACANNNNNNNNNNNNNNNNNNNCAAAAACCAAAAGAAAAATCTGCATT 30432

********* * ** **************************

Human GATACAATCAGGTTTTCTCTGAGGTCCTCTTTGTCTAATCTAGGAAAGATGAACTGAGAA 30510

Chimpanzee GATACAATCAGGTTTTCTCTGAGGTCCTGTTTGTCTAATCTAGGAAAGATGAACTGAGAA 39427

Gorilla GATACAATCAGGTTTTCTCTGAGGTCCTGTTTGTCTAATCTAGGAAAGATGAACTGAGAA 30492

**************************** *******************************

Human TCAAACACCATTACAGATCTGAAAGAAACATTCACCATCTATTCTCTCTGAGTGCTGCTA 30570

Chimpanzee TCCAACACCATTACCGATCTGAAAGAAACATTCACCATCTATTCTCTCTGAGTGCTGCTA 39487

Gorilla TCAAACACCATTACGGATCTGAAAGAAACATTCACCATCTATTCTCTCTGAGTGCTGCTA 30552

** *********** *********************************************

Human CCTGTGAGGTTTCATCTACATGCCACCACCACCTTTGCTAGCTAGATGCTCTTCTCCCTG 30630

Chimpanzee CCTGTGAGGTTTCATCTGCATGCCACCACCACCTTTGCTAGCTAGATGCTCTTCTCCCTC 39547

Gorilla CCTGTGAGGTTTCATCTGCATGCCACCACCACCTTTGCTAGCTACATGCTCTTCTCCCTC 30612

***************** ************************** **************

Human TTCCACAACCTGTCTGGTGTCCGTAACCTGAGTTACCAGGTTTTATAATCTGTTTTTGCA 30690

Chimpanzee TTCCACAACCTGTCTGGTGTCCATAACCTGAGTTACCAGGTTTTATAATCTGTTTTTGCA 39607

Gorilla TTGCACAACCTGTCTGGTGTCCATAACCTGAGTTACCAGGTTTTATAATCTGTTTTTGCA 30672

** ******************* *************************************

Human CATGCTGTAAGACCCAAGCCCAGGATGGTATAAAAGCATCAATAATCTGGCCACTTCTTT 30750

Chimpanzee CATGCTGTAAGACCCAAGCCCAGGATGGTATAAAAGCATCAATAATCTGGCCACTTCTTT 39667

Gorilla CATGCTGTAAGACCCAAGCCCAGGATAGTATAAAAGCATCAATAATCTGGCCACTTCTTT 30732

************************** *********************************

Human AAGAAATTTTGTACTTTTCCATCCAAATTAATAAATTCGTATAGAAACCTCCTTTGCAAA 30810

Chimpanzee AAGAAATTTTGTACTTTTCCATCCAAATTAATAAATTCGTATAGAAACCTCCTTTGCAAA 39727

Gorilla AAGAAATTTTGTAATTTTCCATCCAAATTAATACCTTCATATAGNNNNNNNNNTTGCAAA 30792

************* ******************* *** ***** *******

Human ATTATGACAGTAAGAGAAACCTGACATAGCTCACTCCATCTTGCTTCTAGCCTCACAGGT 30870

Chimpanzee ATTATGACAGTAAGAGAAACCTGACATAGCTCACTCCATCTTGCTTCTAGCCTCACAGGT 39787

Gorilla ATTATGACAGTAAGAGAAACCTGACATAGCTCACTCCATCTTGCTTCTAGCCTCACAGGT 30852

************************************************************

Human TGGCTATCTTCACTCTTTCCTGGGCATGTGCCAAAGCTAATGTTGAGAGAAATTTAGTTT 30930

Chimpanzee TGGCTATCTTCACTCTTTCCTGGGCATGTGCCAAAGCTAATGTTGAGAGAAATTTAGTTT 39847

Gorilla TGGCTATCTTCACTCTTTCCTGGGCATGTGCCAAAGCTAATGTTGAGAGAAATTTAGTTT 30912

************************************************************

Human ATAGTTTATTTTATTATCTTTATTATTTTTTTAAGAAAGGGACTCCCTCTGTCACCCAGG 30990

Chimpanzee ATAGTTTATTTTATTATCTTTATTATTTTTTTAAGAAAGGGACTCCCTCTGTCACCCAGG 39907

Gorilla ATAGTTTATTTTANNNNNNNNNNNNNNNNNNNNNNNNNNNNNNNNNNNCTGTCACTCAGG 30972

************* ******* ****

Human CTGGAGTGCAGTGGCATGATCTCGGCTCACTGCAACCTTCAACTAGCTTGGGAGTTGAAG 31050

Chimpanzee CTGGAGTGCAGTGGCATGATCTCGGCTCACTGCAACCTTCAACTAGCTTGGGAGTTGAAG 39967

Gorilla CTGGAGNNNNGTGGCATGATCTCAGCTCACTGCAACCTTCAACTAGCNNNNNNNNNGAAG 31032

****** ************* *********************** ****

Human CTATTCTCCCACATCAACCTTTTAAGTAGCTTAGACTACAGGCATGAGCCACCATTGCTG 31110

Chimpanzee CTATTCTCCCACATCAACCTTTTAAGTAGCTTAGACTACAGGCACGAGCCACCATTGCTG 40027

Gorilla CTATTCTCCCACATCAACCTTTTAAGTAGCTTAGACTACAGGCATGAGCCACCATTGNNN 31092

******************************************** ************

Human GCTAATTGTTTTATTTTTTATTTATTGTTTATTTTTTTTTAAGTAGAGGTGGGGTTTTTC 31170

Chimpanzee GCTAATTGTTTTATTTTTTATTTTTTGTTTATTTTTTTTTAAGTAGAGATGGGTTTTTTC 40087

Gorilla NNNNNNNNNNNNNNNNNNNNNNNNNNNNNNNNNNNNNNNNNNNNNNNNNNNNNNNNNNNN 31152

Human CAGGCTGCCCAGGCTAATTTTGAACTCCTGAACTCAAGCAAGCTTCGTGCCTCAGCCTCC 31230

Chimpanzee CATGCTGCCCAGGCTAATTTTGAACTCCTGAACTCAAGCAATCTTCGTGCCTCAGCCTCC 40147

Gorilla NNNNNNNNCCAGGCTAATTTTGAACTCCTGAACTCAAGCAAGCTTCGTGCCTCAGCCTCC 31212

********************************* ******************

Human CATGGTGCTGGGATTACAGGTGTCAGCCAACCACTCCTGGCCTGCAAGTTAAATTATAAA 31290

Chimpanzee CATGGTGCTGGGATTACAGGTGTCAGCCA-CCACTCCTGGCCTGCAAGTTAAATTATAAA 40206

Gorilla CATGGTGCTGGGATTACAGGTGTCAGCC-ACCACTCCTNNNNNNNNNNNNNNNNNNNNNN 31271

**************************** ********

Human AGCATTTTCCAAAACAAAATTGCCTTTATAAAACTAAGGAAAGCCTACCAGGTCAGGAGG 31350

Chimpanzee AGCATTTTCCAAAACAAAATTGCCTGTATAAAGCTAAGGAAAGCCTACCAGGTCAGGAGG 40266

Gorilla NNNNNNNNNCAANNNNAAATTGCCTTTATAAAACTAAGGAAAGCCTACCAGGTCAGGAGG 31331

*** ********* ****** ***************************

Human ATAAAGGTCTGAATTCTACTATGATATAAGCATAGTTAAATAATTACCAGCCATTATTTA 31410

Chimpanzee ATAAAGGTCTGAATTCTACTATGATGTAAGCATAGTTAAATAATTACCAGCCATTATTTA 40326

Gorilla ATAAAGGTCTGAATTCTACTATGACATAAGCATAGTTAAATAATTACCAGCCATTATTTA 31391

************************ **********************************

Human GGTGGTCACAAGATTTTGAACTTCCCCAATTACTCCTTCAGATAACATCACTCTTGTAGA 31470

Chimpanzee GGTGGTCATAAGATTTTGAACTTCCCCAATTACTCCTTCAGATAACATCACTATTGTAGA 40386

Gorilla GGTGGTCACAAGATTTTGAACTTCCCCAATTACTCCTTCAGATAACNNNACTATTGTAGA 31451

******** ************************************* *** *******

Human ACCCAGGATTGACATTTTGAGATAGCTTTTCAGATTTTTTGCATTTCTGACTACCAATAG 31530

Chimpanzee ACCCAGGATTGACATTTTGAGATAGCTTTTCAGATTTTTTGCATTTCTGACTACCAATAG 40446

Gorilla ACCCAAGACTGGCATTTTGAGATAGCTTTTCAGATTTTTTGCATTTCTGACTACCAATAG 31511

***** ** ** ************************************************

Human CTGTACCAGTACTCACCAACTGGTCTTTTGCCTTCTACTCAGGAACTGATGCAGGGCAAG 31590

Chimpanzee CTGTACCAGGACTCACCAACTGGTCCTTTGCCTTCTACTCAGGAACTGATGCAGGGCAAG 40506

Gorilla CCGTACCAGGACTCACCAACTGGTCTTTTGCCTTCTACTCAGGGACTGATGCAGGGCAAG 31571

* ******* *************** ***************** ****************

Human AGGACAGCTTCAACTCCCTATGATTTCATCTCTGACACAACCAGTCGGCACTTCCCACTC 31650

Chimpanzee AGGACAGCTTCAACTCCCTATGATTTCATCTCTGACACAACCAGTGGGCACTTCCCACTC 40566

Gorilla AGGACAGCTTCAACTCCCTATGATTTCATCTCAGACACAACCAGTCGGCCCTTCCCACTC 31631

******************************** ************ *** **********

Human CTGGGCCCCCTGCCCACCAAACTATCTTTGAAAAACCCTGATCTCCAAATTTTTGAGAAG 31710

Chimpanzee CTGGGCCCCCTGCCCACCAAACTATCTTTGAAAAACCCTGATCTCCAAATTTTTGAGAAG 40626

Gorilla CTGGGCCCCCTGCCCACCAAACTATCTTTGAAAAACCCTGACCTCCAAATTTTTGAGAAG 31691

***************************************** ******************

Human ACTGATTTGAATAATAATAAAACTCTGGTCTCTTGTTTATCTGTGTATGCATGAATTAAA 31770

Chimpanzee ACTGATTTGAATAATAATAAAACTCTGGTCTCTTGTTTATCTGTGTATGCATGAATTAAA 40686

Gorilla ACTGATTTGAATAATAATAAAACTCTGGTCTCTTGTTTATCTGTGTATGCATGAATTAAA 31751

************************************************************

Human CTCTTCCTCTATTGCACTTACCTTGTCTTGATCATTTAGTTCTCTCTGGGCAATAGGAAA 31830

Chimpanzee CTCTTCCTCTATTGCACTTACCTTGTCTTGATCATTTAGTTCTCTCTGGGCAATAGGAAA 40746

Gorilla CTCTTCCTCTATTGCACTTACCTTGTCTTGGTCATTTAGTTCTCTCTGGGCAATAGGAAA 31811

****************************** *****************************

Human AAAGATCCCACAGGGCAGTTAGGTTGTGGAAGGTTATTAATCTACCTTTCATCAGGTGAT 31890

Chimpanzee AAAGATCCCACAGGGCAGTTAGGTTGTGGAACGTTATTAATCTACCTTTCATCAGGTGAT 40806

Gorilla AAAGATCACACAGGGCAGTTAGGTTGTGGAACGTTATTAATCTACCTTTCATCAGGTGAT 31871

******* *********************** ****************************

Human TTGTAGCGAACCTTCAGAGAGCAAAGGGCAAGTTTTTCCTTGGCTTCTGTAATATGAACA 31950

Chimpanzee TTGTAGCGAACCTTCAGAGAGCAAAGGGCAAGTTTTTCCTTGGCTTCTATAATATGAACA 40866

Gorilla TTGTAGCGAACCTTCAGAGAGCAAAGGGCAAGTTTTTCCTTGGCTTCTATAATATGAACA 31931

************************************************ ***********

Human GGCATGGTTATAAACATAGACATAGACATTGACATGGAAAATGATGTAAGCTGTTTACAG 32010

Chimpanzee GGCATGGTTATAAACATAGACATAGACATTGACATGGAAAATGATGTAAGCTGTTTACAG 40926

Gorilla GGCATGGTTATAAACATAGACATAGACATTGACATGGAAAATGATGTAAGCTGTTTACAG 31991

************************************************************

Human TTAAGGAGGTGCCATAGACTAAATGTCTGTGTCCCATCACAGTCACACACCGTATGCTGA 32070

Chimpanzee TTAAGGAGGTGCCATAGACTAAATGTCTGTGTCCCATCACAGTCACACACCATATGCTGA 40986

Gorilla TTAAGGAGGTGCCATAGACTAAATGTCTGTGTCCCATCACAGTCACACACCATATGCTGA 32051

*************************************************** ********

Human AACCCTAGCCCCCAGTGGGTTGGTATTTGGAAATGGGGCATTAGAAGGTAATTAGTTTTA 32130

Chimpanzee AACCCTAGCCCCCAGTGGGTTGGTATTTGGAAATGGGGCATTAGAAGGTAACCAGTTTTA 41046

Gorilla AACCCTAGCCCCCAGTGGGTTGGTATTTGGAAATGGGGCATTAGAAGGTAATTAGTTTTA 32111

*************************************************** *******

Human GATGACATCCTGAAAGTGGGTCTCCCATGATGGGATCAATGTTTTTATGAGAAGATGACC 32190

Chimpanzee GATGACATCCTGAAAGTGGGTCTCCCATGATGGGATCAATGTTTTTATGAGAAGATGACC 41106

Gorilla GATGACATCCGGAAAGTGGGTCTCCCATGATGGGATCAATGTTTTTATGAGAAGATGACC 32171

********** *************************************************

Human AGCCCAGTGCTCTTTTTTTTTTCTCTCTGCCTTTGAAGCCACCTTTGCAAAATTATGACT 32250

Chimpanzee AGCCCAGTGCTCTTTTTTTTT-CTCTCTGCCTTTGAAACCACCTTTGCAAAATTATGACT 41165

Gorilla AGCCCAGTGCTCTTTTTTTTNNCTCNNNNNNNNNNNNNNNNNNTTTGCAAAATTATGACT 32231

******************** *** *****************

Human GAGAGTGAAAGACATCTTGTTATAGGTAGGTCTTTGTTCTTAGAGCTCCCAAGATGGTGG 32310

Chimpanzee GAGAGTGAAAGACATCTTGTTATAGGTAGGTCTTTGTTCTTAGAGCTCCCAAGATGGTGG 41225

Gorilla GAGAGTGAAAGACATCTTGTTATAGGTAGGTCTTTGTTCTTAGAGCTCCCAAGATGGTGG 32291

************************************************************

Human TGGGCTGCTCCCAAGATGGTGGCAGCTGCTCCCAAGATTGCAGCCAGCCTTTTATTCTCT 32370

Chimpanzee TGGGCTGCTCCCAAGATGCTGGCAGCTGCTCCCAAGATTGCAGCCAGCCTTTTATTCTCT 41285

Gorilla TGGGCTGCTCCCAAGATGGTGGCAGCTGCTCCCAAGATTGCAGCCAGCCTTTTATTCTCT 32351

****************** *****************************************

Human GACCTGCGTTTCTTGGCCTCTCAGGTTCCAAGGAATGGAACTTTGGGCCACACGGTGAGT 32430

Chimpanzee GACCTGGGTTTCTTGGCCTCTCAGGTTCCAAGGAATGGAACTTTGGGCCACACGGTGAGT 41345

Gorilla GACCTGGGTTTCTTGGCCTCTCAGGTTCCAAGGAATGGAACTTTGGGCCACACGGTGAGT 32411

****** *****************************************************

Human GTGATACCTCTACTACAAGCCATGGGTCATGGAAGAGAACTGTGGAAACTGGTGACTAGT 32490

Chimpanzee GTTATACCTCTACTACAAGCCATGGGTCATGGAAGAGAACTGTGGAAACTGGTGACTAGT 41405

Gorilla GTTATACCTCTACTACAAGCCATGGGTCATGGAAGAGAACTGTGGAAACTGGTGACTAGT 32471

** *********************************************************

Human GTTCAGCTCTATTAGGATGACCCTGGGCACCTAGCCATGCAGGAACAATAGCAAGCCTCT 32550

Chimpanzee GTTCAGCTCTATTAGGATGACCCTGGGCACCTAGCCATGCAGGAACAATAGCAAGCCTCT 41465

Gorilla GTTCAGCTCTATTAGGATGANNNTGGGCANCTAGCCATGCAGGAACAATAGCAAGCCTCT 32531

******************** ****** ******************************

Human AGCTTGAACGGGAACAGCAATAGGTGCCTCATTGGATCAGAAATGCAGCAGACACCCTGC 32610

Chimpanzee AGCTTGAACGGGAACAGCAATAGGGGCCTCATTGGATCAGAAATGCAGCAGACACCCTGC 41525

Gorilla AGCTTGAACGGGAACAGCAATGGGTGCCTCATTGGATCAGAAATGCAGCAGACACCCTGC 32591

********************* ** ***********************************

Human CAGATCTGGAGGGGTGGAAGTCAGTGGCAGGTCTATGATGGTGGTGTACAGCAGTGGTGG 32670

Chimpanzee CAGATCTGGAGGGGTGGAAGTCAGTGGCAGGTCTATGATGGTGGTGTACAGCAGTGGTGG 41585

Gorilla CAGATCTGGAGGGGTGGAAGTCAGTGGCAGGTCTATGATGGTGGTGTACAGCAGTGGTGG 32651

************************************************************

Human ATGATGAGTGAAAACTCAGCTTGAGCTGGAACAAACACAGAACAGAAGAGTGTGCAACCG 32730

Chimpanzee ACAATGAGCGAAAACTCAGCTTGAGCTGGAGCAAACACAGAACAGAAGAGTGTGCAACCG 41645

Gorilla NNNNNNNNNNNAAACTCNNNNNNNNCCGGAACAAACACAGAACAGAAGAGTGTGCAACTG 32711

****** * *** *************************** *

Human TAAGATTTAATAGAGTGAAAACACAGCTCCCATAAAATGGGAGGGGACCCAAAGGGG-TT 32789

Chimpanzee TAAGATTTAATAGAGTGAAAACAGAGCTCCCATACAATGGGAGGGGACCCAAGGGGGGTT 41705

Gorilla TAAGATTTAANNNNNNNNNNNNNNNNNNNNNNTACAATGGGAGGGGACCCAAAGGGG-TT 32770

********** ** ***************** **** **

Human CCCAATCCCAGCTCAAATGCCTGGGGTTTATATCCCAATCATTGTCCCTCCCCCTGTGCT 32849

Chimpanzee CCCAATCCCAGCTCAAATGCCTGGGGTTTATATCCCAATCATTGTCCCTCCCCCTGTGCT 41765

Gorilla CCCAATCCCAGCTCAAATGCCTGGGGTTTATATCCCAATCATTGTCCCTCCCCCTGTGCT 32830

************************************************************

Human CTCAGATGATAGATGATTTGACCATTTCTTTAACTCCTGCTTTTAGCCTGATTGATATTT 32909

Chimpanzee CTCAGATGATAGATGATTTGACCATTTCTTTAACTCCTGCTTTTAGCCTGATTGATATTT 41825

Gorilla CTCAGATGATAGATGATTTGACCATTTCTTTAACTCCTGCTTTTAGCCTGATTGATATTT 32890

************************************************************

Human AGTGAGCTCTCTTTACTACCTGATTGGTCAGGTGTGAGCTGAGTTACAAACCCTGGGTTT 32969

Chimpanzee AGTGAGCTCTCTTTACTACCTGATTGGTCAGGTGTGAGCTGAGTTACAAACCCTGGGTTT 41885

Gorilla AGTGNNNNNNNNNNACTACCTGATTGGTCAGGTGTGAGCTGAGTTACAAACCCTGGGTTT 32950

**** **********************************************

Human AAAGGTGGTTGTGGTCACCTTCCCCAGCTAGGCTTAGGAATTCTTAGTCGGCATAGGAAA 33029

Chimpanzee AAGGGTGGTTGTGGTCACCTTCCCCAGCTAGGCTTAGGAATTCTTAGTCGGCATAGGAAA 41945

Gorilla AAAGGTGGTTGTGGTCACCTTCCCCAGCTAGGCTTAGGAATTCTTAGTTGGCATAGGAAA 33010

** ********************************************* ***********

Human TCCAGCTAGTCCTGTCTCTGAATCTGACTTAACTCCATCTTGCTTTCAACCTCCAAGCTG 33089

Chimpanzee TCCAGCTAGTCCTGTCTCTGAATCTGACTTAACTCCATCTTGCTTTCAACCTCCAAGCTG 42005

Gorilla TCCAGCTAGTCCTGTCTCTGAATCTGACTTAACTCCATCTTGCTTTCAACCTCCAAGCTG 33070

************************************************************

Human TTGTTGCTTATTCCAGGGCGTAGGCTGAACTACTTTTGGGAGGGACTTACGGTTTAAAAC 33149

Chimpanzee TTGTTGCTTATTCCTGGGTGTAGGCTGAACTACTTTTGGGAGGGACTTACGGTTTAAAAC 42065

Gorilla TTGTTGCTTATTCCTGGGTGTAGGCTGAACTACTTTTGGGAGGCACTTACGGTTTAAAAC 33130

************** *** ************************ ****************

Human AAAGATGATAACAGCCCTTTCTCAAAACAAGGCTTATTTCTCCCTTGGGACTAGACTGCC 33209

Chimpanzee AAAGATGATAACAGCCCTTTCTCAAAACAAGGCTTATTTTTCCCTTGGGACTAGACTGCC 42125

Gorilla AAAGATGATCACAGCCCTTTCTCAAAACAAGGCTTATTTTTCCTTGGGGACTAGACTGCC 33190

********* ***************************** *** * **************

Human TTTGTTGGACTAACAAATCAGCCACAAGATTACAAATTATGGTTTAGTAGTCGACTAATC 33269

Chimpanzee TTTGTTGGACTAACAAATCAGCCACAAGATTACAAATTATGGTTTAGGAGTCGACTATTC 42185

Gorilla TTTGTTGGACTAACAAATCAGCCACAAGATTACAAATTATGGTTTAGTAGTCGACTATTC 33250

*********************************************** ********* **

Human AGCTGAAAACTACAAGATTCTGACCTTCTCTAAAATGCTCCTAAGACCAATGCTCGAGAT 33329

Chimpanzee AGCTGAAAACTACAAGATTCTGACGTTCTCTAAAGTGCTCCTAAGACCAATGCTTGAGAT 42245

Gorilla AGCTGAAAAGTACAAGATTCTGACCTTCTCCAAAATGCTTCTAAGACCAATGCTTGAGAT 33310

********* ************** ***** *** **** ************** *****

Human ATTTTGCAGAGCCTACACTTGATTGATCAGCTGGAACCACCCAGACTGATAAACTGGCTC 33389

Chimpanzee ATTTTGCAGAGCCTACACTTGATTGATCAGCTGGAACCACCCAGACTGATAAACTGGCTC 42305

Gorilla ATTTTGCAGAGCCTACACTTGATTGATCAGCTGGAACCACCCAGACTGATAAACTGGCTC 33370

************************************************************

Human ACCTGACGTTGTGGCCCCCACCCAGGAAATGACTCAGCACAAGACGATGGCCACGACTTC 33449

Chimpanzee ACCTGATGTTGTGGCCCCCACCCAGGAAATGACTCAGCACAAGAGGATGGCCACGACTTC 42365

Gorilla ACCTGATGTTGTGGCCCCCACCCAGGAAATGACTCAGCACAAGAGGATGGCCACAACTTC 33430

****** ************************************* ********* *****

Human CCATGATTTCATCTCCAATCCCACCAATCAGCACTCTTGACTCACTGCTGCACTACCCAC 33509

Chimpanzee CCATGATTTCATCTCCAATCCCACCAATCAGCACTCTTGACTCACTGCTGCACTACCCAC 42425

Gorilla CCATGATTTCATCTCCAACCCCACCAATCAGCACTCTTGACTCACTGCTGCACTACCCAC 33490

****************** *****************************************

Human CAAATTATCCTTAAATACTCTGATCCCCAAATGCTCAGGGAGACTAATTTCAGTAATAAT 33569

Chimpanzee CAAATTATCCTTAAATACTCTGATCCCCAAATGCTCAGGGAGACTAATTTCAGTAATAAT 42485

Gorilla CAAATTATCCTTAAATACTCTGATTCCCAAATGCTCAGGGAGACTNNTTTCAGTAATAAT 33550

************************ ******************** *************

Human AAAACTTCAGTTTCCCATACAGCCAGCTCTGCATAAAATACTCTTTCTCTATTGTAAGTC 33629

Chimpanzee AAAACTTCAGTTTCCCATACAGCCAGCTCTGCATAACGTACTCTTTCTCTACTGTAATTC 42545

Gorilla AAAACTTCAGTTTCCCATACAGCCAGCTCTGCATAAAATACGCTTTCTCTATTGTAATTC 33610

************************************ *** ********* ***** **

Human CCCTGTCTTGAGAAATTGGCTCTCTTTAGGCAGCGGGCAAGGTGAACCCACTGGGCAGCT 33689

Chimpanzee CCCTATCTTGAGAAATTGGCTCTGTTTAGGCAGTGGGCAAGGTGAACCCACTGGACAGCT 42605

Gorilla CCCTGTCTTGAGAAATTGGCTCTGTTTAGGCAGTGGGCAAGGTGAACCCACTGGGCAGCT 33670

**** ****************** ********* ******************** *****

Human ATACTATGTGAACTTAGAGTGAGAAACTGACCATCAACAAACCAGGAAGAGAGCTCTCTC 33749

Chimpanzee ATACTATGTGGACTTAGAGTGAGAAACTGACCGTCAACAAACCAGGAAGAGAGCTCTCTC 42665

Gorilla ATACTATGTGAACTTAGAGTGAGAAACTGACCATCAACAAANNNGGAAGAGAGCTCTCTC 33730

********** ********************* ******** ****************

Human CAGAAACCAGGTTGACATCTTGATGAACTTGGACTTCCAAAATTCTGATAAATAATTTTT 33809

Chimpanzee CAGAAACCAGGTTGACATCTTGATGATCTTGGACTTCCAAAATTCTGATAAATAATTTTT 42725

Gorilla CAGAAACCAGGTTGACATCTTGATGATCTTGGACTTCCAAAATTCTGATAAACAATTTTT 33790

************************** ************************* *******

Human GTAAGCCCTCCAGTTCATGGTATCTGTTACAGCAGACTGAGCAGTCCTTCAAGAATCCCT 33869

Chimpanzee GTAAGCCCTCCAGTTCATGGTATCTGTTACAGCAGACTGAGCAGTCCTTCAAGAATCCCT 42785

Gorilla GTAAGCCCTCCAGTTCATGGTGTCTGTTACAGCAGACTGAGCAGTCCTTCAAGAATCCCT 33850

********************* **************************************

Human CACATAGCCATAGATCTACCTACACACATACAAAAATCTTTAACAACCTAGGACATGTCT 33929

Chimpanzee CACATAGCCATAGATCTACCTACACACATACAAAAATCTTTAACAACCTAGGACATGTCT 42845

Gorilla CACATAGCCATAGATTTACCTACACACATACAAAAATCTTTNACAACCTAGGACATGTCT 33910

*************** ************************* ******************

Human TTCAGGTACCTATGAATGTGTCACAGATACAAAGATAGTGTCAACTTTAAATAACCAAAA 33989

Chimpanzee TTCAGGTACCTATGAAAGTGTCACAGATACAAAGATAGTGTCAACTTTAAATAACCAAAA 42905

Gorilla TTCAGGTACCTATGAAAGTGTCACAGATACAAAGATAGTGTCAACTTTAAATAACCAAAA 33970

**************** *******************************************

Human AGGTTAGAGTATAATTCAAACAAAGTTTA--TTCAAGACCCAAGTGTGAAGATGGATGGT 34047

Chimpanzee AGGTTAGAGTATAATTCAAACAAAGTTTAAATTCAAGACCCAAGTGTGAAGATGGATGGT 42965

Gorilla AGGTTAGAGTATAATTCAAACAAAGTTTA--TTCAAGACCCAAGTGTGAAGATGGATGGT 34028

***************************** *****************************

Human TCACACCCATCATACCAACTCCAAAATTATGGAGTAGGAAAGTTAAGGTTTCATGTATAG 34107

Chimpanzee TCACACCCATCATACCAACTCCAAAATTATGGAGTAGGGAAGTTAAGGTTTCATGTATAG 43025

Gorilla TCACACCCATCATACCAACTCCAAAATTATGGAGTAGGAAAGTTAAGGTTTCATGTATAG 34088

************************************** *********************

Human AGCCAGAGACAGATGAGTTTTTAGCAAGATTACCACATCATTCCTACAAGATTGGCACAG 34167

Chimpanzee AGCCAGAGACAGATGAGTTTTTAGCAAGATTACCACATCATTCCTACAAGATTGGCACAG 43085

Gorilla AGCCAGAGACAGATGAGTTTTTAGCAAGATTACCACATCATTCCTGCAAGATTGGCACAG 34148

********************************************* **************

Human AGTTGCAGCCATTAGATTGGTTACAGGCAGTTTTTATGGGGGGAAGAGTACATTTAACAA 34227

Chimpanzee AGTTGCAGCCATTAGATTGGTTATAGGCAGTTTTTATGGGGGGAAGAGTACATTTAACAA 43145

Gorilla AGTTGCAGCCATTAGATTGGTTATAGGCAGTGTTTTTGGGGGGAAGAGTACATTTAACAA 34208

*********************** ******* *** ************************

Human TTTTTTTAAACAGAGGCTGTAACAGTCATGGATTTTCTTTATCTACTCTCAGTGAAGCTG 34287

Chimpanzee TTTTTTTAAACAGAGGCTGTAACAGTCATGGATTTTCTTTATCTACTCTCAGTGAAGCTG 43205

Gorilla TTTTTTTAAACAGAGGCTGTAACAGTCACGGATTTTCTTTATCTACTCTCCGTGAAGCTG 34268

**************************** ********************* *********

Human GACAACCCAGGGGGGCTTAATCTATCACAAGTATCATTAATTAAGAAGGGCAGACAGGGG 34347

Chimpanzee GACAACCCAGGGGAGCTTAATCTATCACAAGTATCATTAATTAAGAAGGGCAGACAGGGG 43265

Gorilla GACAACCCAGGGGAGCTTAATCTATCACAAGTATCATTAATTAAGAAAGGCAGAAGGGGG 34328

************* ********************************* ****** ****

Human CCTGGCATGGTGGCTCATGCCTGTAATCTGGAGGCTGAGATGGGCAGATCACAAGGTCAG 34407

Chimpanzee CCTGGCATGGTGGCTCATGCCTGTAATCTGGAGGCTGAGATGGGCAGATCACAAGGTCAG 43325

Gorilla CCTGGCGTGGTGGCTCATGCCTGTAATCTGGAGGCTGAGATGGGCAGATCACAAGGTCAG 34388

****** *****************************************************

Human GAGAGCAAGACCATCCTGGCTAACATGGTGAAACCATGTTAAAAATCTACTGGAAAAAAA 34467

Chimpanzee GAGAGCAAGACCATCCTGGCTAACATGGTGAAACCATGTTAAAAATCTACTG-AAAAAAA 43384

Gorilla GAGAACAAGACCATCCTGGCTAACATGGTGAAACCATGTTAAAAATCTACTGAAAAAAAA 34448

**** *********************************************** *******

Human AAAAAAAAAAATAGCCAGGCATGGTTGTGGGTGCCTATAGTCCAAGCTACTTGGGAGGCT 34527

Chimpanzee AAAAAAAAAATTAGCCAGGCATGGTTGTGGGTGCCTGTAGTCCAAGCTACTTGGGAGGCT 43444

Gorilla AAAAAAAAAATTNNNNNNNNNNGGTNNNNNNNNNNNNNNNNNNNNNNNNNNNNNNNNNNN 34508

********** * ***

Human GAGGCAGGAGAATGGTGTGGACCTGGGAGGAGAAGCTTGCAGTGAGCTGAGATCATGCCA 34587

Chimpanzee GAGGCAGGAGAATGGTGTGGACCTGGGAGGAGAAGCTTGCAGTGAGCTGAGGTCATGCCA 43504

Gorilla NNNNNNNNNNNATGGTGTGGACCTGGGAGGAGAAGCTCGCAGTGAGCTGAGATCATGCCA 34568

************************** ************* ********

Human CTGCACTCAGCCTGATGTTGCTTAATTCTCCCTAGTCTTTTTACAGAACACGGAGAATTA 34647

Chimpanzee CTGCACTCAGCCTGATGTTGCTTAATTCTCCCTAGTGTTTTTACAGAACACGGAGAATTA 43564

Gorilla CTGCACTCAGCCTGATGTTGCTTAATTCTCCCTAGTCTTTTTACAGAACACGGAGAATTA 34628

************************************ ***********************

Human AAAAGTGAGTTAATCTATAATCTGAGAACAGAAGTTGCTACCATATGTGACTCAGATGAC 34707

Chimpanzee AAAAGTGAGTTAATCTATAATCTGAGAACAGAAGTTGCTACCATATGTGACTCAGATGAC 43624

Gorilla AAAAGTGAGTTAATCTATAATCTGAGAACAGAAGTTGCTACCATATGTGACTCAGATAAC 34688

********************************************************* **

Human GGTCACATCTCTCTCATGGCTTAAAGTGCTCTTGTGGGGGACTGCCATAGCTTTTAAATT 34767

Chimpanzee AGTCACATCTCTCTCATGGCTTAAAGTGCTCTTGTGGGGGAGTGCCATAGCTTTTAAATT 43684

Gorilla AGTCNNNNNTCTCTCATGGCTTAAAGTGCTCTTGTGGGGGAGTGCCATAGCTTTTAAATT 34748

*** ******************************** ******************

Human ATATTTATTTTCACAATAAACATAGTCATAGGCATAGATGCAAACTGTTAACAATAAATG 34827

Chimpanzee ATACTTATTTTCACAATAAACATAGTCATAGGCATAGATGCAAACTGTTAACAATAAATG 43744

Gorilla ATATTTATTTTCACAATAAACATAGTCATAGGCATAGATGCAAACTGTTAACAATAAATG 34808

*** ********************************************************

Human AGATATCCTTCAGGAATCAAGGATATCGACATGACAAGGATGAGGTTAGATCAGAAATTT 34887

Chimpanzee AGATATCCTTCAGGAATCAAGGATATAGACATGACAAGGATGAGGTTAGCTCAGGAGTTT 43804

Gorilla AGATATCCTTCAGGAATCAAGGATATAGACATGACAAGGATGAGGTTAGATCAGAAGTTT 34868

************************** ********************** **** * ***

Human AATAAAGGAAAGAAAGCCCTATGCCGCAGAGAGGCATCCCAAAAAAGGGTTGCAATTTCA 34947

Chimpanzee AATAAAGGAAAGAAAGCCCTATGCCGCAGAGAGGCATCCCAAAAAAGGGTTGCAATTTCA 43864

Gorilla ANNNNNNNNNNNAAAGCCCTATGCCACAGAGAGGCATCCCAAAAAAGGGTTGCAATTTCA 34928

* ************* **********************************

Human CAGTTGAATATAGAGCCTTTTATAAGAAATCGTTGAGGGATGAGTGTTTCATTTGCATAA 35007

Chimpanzee CAGTTGAATATAGAGCCTTTTATAAGAAATCGTTGAGGGATGAGTGTTTCATTTGCATAA 43924

Gorilla CAGCTGAATATAGAGCCTTTTATAAGAAATCATTGAGGGATGAGTGTTTCATTTGCATAA 34988

*** *************************** ****************************

Human GGTGTGAAGTTCTGGTAGCTCCACCCCATCCTCCTAGTGCACCCACAGGCCTTTAGCTTG 35067

Chimpanzee GGTGTGAAGTTCTGGTAGCTCCACCCCATCCTCCTAGTGCACCCACAGGCCTTTAGCTTG 43984

Gorilla GGTGTGAAGTTCTGGTAGCTCCACCCCATCCTCCTAGTGCACCCACAGGCCTTTAGCTTG 35048

************************************************************

Human AGTTACTCCATATTGTTTTGTTCCCCTTATGATGCCTGTGCCAGGGGATGGAATTTTCCA 35127

Chimpanzee AGTTACTCCATATTGTTTTGTTCTCCTTACTATGCCTGTGTCAGGGGATGGAATTTTCCA 44044

Gorilla AGTTACTCCATATTGTTTTGTTCNNNNNNNNNNNNNNNTGTCAGGGGATGGAATTTTCCA 35108

*********************** ** *******************

Human TTGCAGGCAAGTCTAGTCAAATCAGCTATATAGACTTTCTTATCTGTATGGCTGTGGGCA 35187

Chimpanzee TTGCAGGCAAGTCTAGTCAAATCAGCTATATAGACTTTCTTATCTGCATGGCTGTGGGCA 44104

Gorilla TTGCAGGCAAGTCTAGTCAAATCAGCTATATAGACCTTCTTATCTGCACGGCTGTGGGCA 35168

*********************************** ********** * ***********

Human TGTCTCAGTCAAGCTCCCCTGTGTAAGTTCCCTTATGTGTGCCTGCAGGCTATTCTTTTG 35247

Chimpanzee TGTCTCAGTCAAGCTCCCCTCTGTAAGTTCCCTTATGTGTGCCTGCAGGCTATTCTTTTG 44164

Gorilla TGTCTCAGTCAAGCTCCCCGGTGTAAGTTCCCTTATCTGTGCCTGCAGGCTATTCTTTTG 35228

******************* *************** ***********************

Human TTTAAAAGAATTCAACCATTTAACCTTGGAACAACTCTAACTGGCTGCTTAATGGGTTTC 35307

Chimpanzee TTTAAAAGAATTCAACCATTTAACCTTGGAACAACTCTAACTGGCTGCTTAATGGGTTTC 44224

Gorilla TTTAAAAGAATTCAACCATTTAACCTTGGAACAACTCTAACTGGCTGCTTAATGGGTTTC 35288

************************************************************

Human TTCCTCTTTTCCCTCTCAGTACTAGATGATCTAGGCAGAAACCTTGACCTCCACTAGATG 35367

Chimpanzee TTCCTTTTTTCCCTCTCAGTACTAGATGATCTAGGCAGAAATCTTGACCCCTACTAGATG 44284

Gorilla TTCCTTTTTTCCCTCTCAGTACTAGATGCTCTAGGCAGAAATCTTGACCCCCACTAGATG 35348

***** ********************** ************ ******* * ********

Human ATCTAGGCAGAAATCTTGACCCCCAAGCTCATCTCCTATCTTCAACATATGTAAGGATAA 35427

Chimpanzee ATCTAGGCAGAAATCTTGACCCCCAAGCTCATCTCCTATCTTCAACATATGTAAGGATAA 44344

Gorilla ATCTAGGCAGAATTCTTGACCCCCAAGCTCATCTCCTATCTTCAACATATGTAAGGATAA 35408

************ ***********************************************

Human GCTCAGTATTCAGCAAAATAACTGAGAGCTGGTGAGGGCTACTTTGCGGGACATTTTCAA 35487

Chimpanzee GCTTAGTATTCAGCAAAATAACTGAGAGCTGGTGAGGGCTACTTTGCGGGACATTTTCAA 44404

Gorilla GCTTAGTATTCAGCAAAATAACTGAGAGCTGGTGAGGGCTACTTTGCGGGACATTTTCAA 35468

*** ********************************************************

Human GTTCTGTGCATTTCTCTTCTGTTAGGAGAGTATCAAGGAGATTCCACGTGAAATTGATTG 35547

Chimpanzee GTTCTGTGCATTTCTCTTCTGTTAGGAGAGTATCAAGGAGATTCCACGTGAAATTGATTG 44464

Gorilla GTTCTGTGCATTTCTCTTCTGTTAGGAGAGTATCAAGGAGATTCCACGTGAAATTGATTG 35528

************************************************************

Human AAGGGACGTCAAGTGTCTCATCCACTTTGGGCTGCAATAATGAAAATACCATAGACTGCG 35607

Chimpanzee AAGGGACGTCAAGTGTCTCATCCAGTTTGGGCTGCAATAATGAAAATACCATAGACTGCG 44524

Gorilla AAGGGACGTCAAGTGTCTCATCCAGTTTGGGCTGCAATAATGAAAATACCATAGACTGCG 35588

************************ ***********************************

Human TAGCTTCTAAAAAGGATAAATTGATTGCATACTCTTCTGAAAGCTGGAAGTCCAAGATCA 35667

Chimpanzee TAGCTTCTAAAAAGGATAAATTGATTGCATACTCTTCTGAAAGCTGGAAGTCCAAGATCA 44584

Gorilla TAGCTTCTAAAAAGGATAAATTGATTGCATACTCTTCTGAAAGCTGGAAGTCCAAGATCA 35648

************************************************************

Human AAACGTGGTAGATTTAGTGTCCCGTGGGGACTTAGGTCCGGTGAGGACTTAGGTCCATAG 35727

Chimpanzee AAACGTGGTAGATTTAGTGTCCCGTGGGGACTTAGGTCCGGTGAGGACTTATGTCCATAG 44644

Gorilla AAACGTGGTGGATTTAGTGTCCCGTGGGGACTTAGGTCCGGTGAGGACTTAGGTCCATAG 35708

********* ***************************************** ********

Human ACAGTATCTTCTAGCTGTATTCTCACACAGTGAAAGAGGTGAAGAAGATCTATTGGGTCC 35787

Chimpanzee ACAGTATCTTCTAGCTGTATTCTCACACAGTGAAAGAGGTGAAGAAGATCTACTGGGTCC 44704

Gorilla ACAGTATCTTCTAGCTGTATTCTCACACAGTGAAAGAGGTGAAGAAGATCTATTGGGTCC 35768

**************************************************** *******

Human CTTTTATAATAGCACTAATTCCATTCATGTGGGCTCCACTCTCATCACCTTCCATCTCCT 35847

Chimpanzee CTTTTATAACAGCACTAATTCCATTCAGGTGGGCTCCACTCTCTTCACCTTCCATCTCCT 44764

Gorilla CTTTTATAATAGCACTAATTCCATTCATGTGGGCTCCACTCTCATCACCTTCTATCTCCT 35828

********* ***************** *************** ******** *******

Human AATACCATCACCTTGGAAGTTAGGACTCAAGATGTTATTCTGGAAAAACACAAACATTTG 35907

Chimpanzee AATACCATCACCTTGGAAGTTAGGACTCAAGATGTTATTCTGGAAAAACACAAACATTTG 44824

Gorilla AATACCATCACCTTGGAAGTTAGGACTCAAGATGTTATTCTGGAAAAACACAGATATTTG 35888

**************************************************** * *****

Human GAGCATAGCATCAAGAGACCTTCTCAGTGAATCTGGGGGTCACAGAATAGAGAGACTATA 35967

Chimpanzee GAGCATAGCATCAAGAGACTTTCTCAGTGAATCTGGGGGTCACAGAATAGAGAGACTATA 44884

Gorilla GAGCATAGCATCAAGAGACCTTCTCAGTGAATCTGGGGGTCACAGAATAGAGAGACTATA 35948

******************* ****************************************

Human GGCTGACTCTTCTTTGAAGAAGGTGAATGATACTAGGTTGTCTGCAAGTTTAGTGAAGGG 36027

Chimpanzee GGCTGACTCTTCTTTGAAGAAGGTGAATGATACTAGGTTGTCTGCAAGTTTAGTGAAGGG 44944

Gorilla GGCTG-CTCTTCTTTGAAGAAGGTGAATGATACTAGGTTGTCTGCAAGTTTAGTGAAGGG 36007

***** ******************************************************

Human TGAATAAAGAGGTGAGACTACAAGAACACAGGCTTGGCCATCTGGTGTTGTTGGGCAGCA 36087

Chimpanzee TGAATAAAGAGGTGAGACTGCAAGAACACAGGCTTGGCCATCTGGTGTTGTTGGGCAGCA 45004

Gorilla TGAATAAAGAGGTGAGACTGCAAGAACACAGGCTTGGCCATCTGGTGTTGTTGGGCAGCA 36067

******************* ****************************************

Human CAGAATGATTGACAATTTCTTTTGGCCAAGTGTGCTCTGAAAGATGACAGCCTTGATCAC 36147

Chimpanzee GAGAATGATTGACAATTTCTTTTGGCCAAGTGTGCTCTGAAAGATGACAGCCTTGATCAC 45064

Gorilla CAGAATGATTGCCAATTTCTTTTGGCCAAGTGGGCTCTGAAAGATGACAGCCTTGATCAC 36127

********** ******************** ***************************

Human CCAGATGGGACACTGACAAAGTTTCTCCCTTCTGAGAATGTGACACTACAGGGAAAATAG 36207

Chimpanzee CCAGATGGGACACTGACAAAGTTTCTCCCTTCTGAGAATGTGACACTACAGGAAAAATAG 45124

Gorilla CCAGATGGGACACTGACAAAGTTTCTCCCTTCTGAGAATGTGACACTACAGGAAAAATAG 36187

**************************************************** *******

Human AACCAGAAGTATAGTCAGAAGCTAGAAACCAGAGATGTGGGTGTTGCAATAAGTCCTATA 36267

Chimpanzee AACCAGAAGTATAGTCAGAAGCTAGAAGCCAGAGATGTGGGTGTTGCAATAAGTCCTATA 45184

Gorilla AACCAGAAGTATAGTCAGAAGCTAGAAGCCAGAGATGTGGGTGTTGCAATAAGTCCTATA 36247

*************************** ********************************

Human AAAGCAACAATGCCCCAAGGAAAAAAAAAA----TTA----TGTAAGTGAGTATGACATC 36319

Chimpanzee AAAGCAACAATGCCCCAAGGAAAAAAAAAAAAAATTA----TGTAAGTGAGTATGACATC 45240

Gorilla AAAGCAACAATGCCCCAAGGGAAAAAAAAAA----NA----TGTAAGTGAGTATGACATC 36299

******************** ********* * *******************

Human CTTCTTCAAATATCCTTGCAAACTTGATAAAGTTTGACAGATGAATGGAAAGTGAGATTG 36379

Chimpanzee CTTCTTCAAATATCTTTGCAAACTTGATAAAGTTTGACAGATGAATGGAAAGTGAGATTG 45300

Gorilla CTTCTTGAAATATCTTTGCAAACTTGATAAAGTTTGACAGATGAATGGAAAGTGAGATTG 36359

****** ******* *********************************************

Human TTTTTGTTTGTTTTGAGACAGGGTCTCATTCTGTTGCCCAGGCTGAAGTGCAATGGCTTG 36439

Chimpanzee TTTTTGTTTGTTTTGAGACAGGGTCTCATTCTGTTGCCCAGGCTGAAGTGCAATGGCTTG 45360

Gorilla TTTTTGTTTGTTTTGAGTTAGGGTCTCATTCTGTTGCCCAGGCTGAAGTGCAATGGCTCG 36419

***************** *************************************** *

Human CTCTTGGTTCACTGCAACCTCTACATCCCAGGCTCTAGTGATCTTCTCATCTTAGCTTCC 36499

Chimpanzee CTCTTGGTTCACTGCAACCTCTACATCCCAGGCTCTAGTGATCTTCTCATCTTAGCTTCC 45420

Gorilla CTCT-GGTTCACTGCAACCTCTACATCCCAGGCTCTAGTGATCTTCTCATCTTAGCTTCC 36478

**** *******************************************************

Human TGAGTAGCTGGGACTATCGGTGTGCACCACCTTGCCAAGCTAATTCTTTTATTTTATTGT 36559

Chimpanzee TGAGTAGCTGGGACTATCGGTGTGCACCACCTTGCCAAGCTAATTCTTTTATTTTATTGT 45480

Gorilla TGAGTAGCTGGCACTATCGGTGTGCACCACCTTGCCAAGCTAATTCTTTTATTTTATTGT 36538

*********** ************************************************

Human ATAGGTGGGTTCTCACTGTGCTGCCCAGGCTGGTCTTGAAATCCTGAGCTCAAGCAATCC 36619

Chimpanzee ATAGGTGGGCTCTCACTGTGCTGCCCAGGCTGGTCTTGAAATCCTGAGCTCAAGCAATCC 45540

Gorilla ATAGGTGGGCTCTCACTGTGCTGCCGAGGCTGGTCTTGAAATCCTGAGCTCAAGCAATCC 36598

********* *************** **********************************

Human ATCCCAAAATGTTGGGACTACAGATGTGAGCCACTGCATCTGGTCTGGAATTTTCAAATA 36679

Chimpanzee ATCCCAAAATGTTGGGACTACAGATGTGAGCCACTGCATCTGGTCTGGAATTTTCAAATA 45600

Gorilla ATCCCAAAATGTTGGGACTACAGATGTGAGCCACTGCATCTGGTCTGGAATTTTCAAATA 36658

************************************************************

Human TAATGAGATATTGAAATTATACTTTAGGTTGCATTGGTCATTTAAACATACATGAATCAG 36739

Chimpanzee TAATGAGATATTGAAATTATACTTTAGGTTGCATTGGTCATTTAAACATACGTGAATCAG 45660

Gorilla TAATGAGATATTGAAATTATACTTTAGATTGCATTGGCCATTTAAACATACATGAATCAT 36718

*************************** ********* ************* *******

Human ATTATTCACCAATATCTTGAATAGTCCAGAAAATATTTGAAGTGTGCCTGAAATATCACC 36799

Chimpanzee ATTATTCACCAATATCTTGAATAGTCCAGAAAATATTTGAAGTGTGCCTGAAATATCACC 45720

Gorilla ATTATTCACCAATATCTTGAATAGTCCAGAAAATATTTGAAGTGTGCCTGAAATATCACC 36778

************************************************************

Human TACAGAAAAGATATGAAAATGTGATAGCATGACAAACTTATATTTTCCCATCATTTTATC 36859

Chimpanzee TACAGAAAAGATATGAAAATGTGATAGCATGACAAACTTATATTTTCCCATCATTTTATC 45780

Gorilla TACAGAAAAGATATGAAAATGTGATAGCATGACAAACTTATATTTTCCCATCATTTTATC 36838

************************************************************

Human CAGTGACTCAAAGAAAGAGAGAAGCCTGCTGAGAGACATACTGAGAAGAAAAATGTAAAA 36919

Chimpanzee CAGTGACTCAAAGAAAGAGAGAAGCCTGCTGAGAGACATACTGAGAAGAAAAATGTAAAA 45840

Gorilla CAGTGACTCAAAGAAAGAGAGAAGCCTGCTGAGAGACATACTGAGAAGAAAAATGTAAAA 36898

************************************************************

Human AGACTATTCAAAGAAGGTTGAGTGTCAACAAGGAACTCTTATTGGGACAAGAGAGGAAGA 36979

Chimpanzee AGACTATTCAAAGAAGGTTGAGTGTCAACAAGGAACTCTTATTGGGACAAGAGAGGAAGA 45900

Gorilla AGACTATTCAAAGAAGGTTGAGTGTCAACAAGGAACTCTTATTGGGACAAGAGAGAAAGA 36958

******************************************************* ****

Human ATAGACGTCTCTTCTTGCAACAAGAAATAGAAGAGTAGTTGTGAACTCCAGGGAAAATCA 37039

Chimpanzee ATAGATGTCTCTTCTTGCAACAAGAAATAGAAGAGTAGTTGTGAACTCTAGGGAAAATCA 45960

Gorilla ATAGATGTCTCTTCTTGCAACAAGAAATAGAAGAGTAGTTGTGAACTCCAGGGAAAATCA 37018

***** ****************************************** ***********

Human CAATCATGCAATGTTTTCAAGTAATGTGCTAGACTTTAAGAAAGAAGGATATGACTCTCC 37099

Chimpanzee CAATCATGCAATGTTTTCAAGTAATGTGCTAGACTTTAACAAAGAAGGATATGACTCTCC 46020

Gorilla CAATCATGCAATGTTTCCAAGTAATGTGCTGGACTTTAAGAAAGAAGGATATGACTGTCC 37078

**************** ************* ******** **************** ***

Human TGATACATGAACTCTTCTTAAAGCAGTTCATGTTGAATATCGTGGGACTAAATCACATGG 37159

Chimpanzee CGATACATGAACTCTTCTTAAAGCAGTTCATGTTGAATATCGTGGGACCAAATCACATGG 46080

Gorilla TGATACATGAACTCTTCTTAAAGCAGTTCATGTTGAATATCGTGGGACTAAATCACATGG 37138

*********************************************** ***********

Human GTTTCTGTAGATTCATTCATACTCTCTGGGGTCCACTGGTAAATATCCACACAAGGATTA 37219

Chimpanzee GTTTCTGTAGATTCATTCATACTCTCTGGGGTCCACTGGTAAATATCCACACAAGGATTA 46140

Gorilla GTTTCTGTAGATTCATTCATACTCTCTGAGGTCCACTGGTAAATATCCACACAAGGATTA 37198

**************************** *******************************

Human TTCTCAATTCTATTGATTGGTTTTTAATTTTCAGAATTAACTTTATTGGAAACCAATAGG 37279

Chimpanzee TTCTCAATTCTATTGATCGGTTTTTAATTTTCAGAATTAACTTTATTGGAAACCAATAGT 46200

Gorilla TTCTCAATTCTATTGATTGGTTTTTAATTTTCAGAATTAACTTTATTGGAAACCAATAGG 37258

***************** *****************************************

Human AAGTCATTATTTTTACGGATTAAAAAACATCAGTTTCAAAGGTTAATTAATTAATGAATG 37339

Chimpanzee AAGTCATTATTTTTACGGATTAAAAAACATCAGTTTCAAACGTTAATTAATTAATGAATG 46260

Gorilla AAGTCATTATTTTTACGGATTAAAAGACATCAGTTTCAAACATTAATTAATTAATGAATG 37318

************************* ************** ******************

Human GAAAAGCCAATGTAAATTAAGGAAGGAAAGAGGGGATATCAAGGAAAATATATAATGCTT 37399

Chimpanzee GAAAAGCCAATGTAAATTAAGGACGGAAAGAGAGGATATCAAGGAAAATATATAATGCTT 46320

Gorilla GAAAAGCCAATGTAAATTAAGGAAGGAAAGAGGGGATATCAAGGAAAATATATAATGCTT 37378

*********************** ******** ***************************

Human ACTTTGCTAATCACTTAAGAGTAAGATGTTATATCCTACAGTCCAAATGTAATGGTTCAA 37459

Chimpanzee ACTTTGCTAATCACTTAAGAGTAAGATGTTATATCCTACAGTCCAAATGTAATGGTTCAA 46380

Gorilla ACTTTGCTAATCACTTAAGAGTAAGATGTTATATCCTACAGTCCAAATGTAATGGTTCAA 37438

************************************************************

Human GAAATAAACATAGAAAAAATTACTTTTAGATTCAGTCTTTCAAAAAATGCAGAATTATAA 37519

Chimpanzee GAAATAAACATAGAAAAAATTACTTTTAGTTTCAATCTTTCAAAAAATGCAGAATTATAA 46440

Gorilla GAAATAAACATAGAAAAAATTACTTTTAGATTCAATCTTTCAAAAAATGCAGAATTATAA 37498

***************************** **** *************************

Human AAGTTATGATATTATGAGCAAGTATTATTTGTCATTGTTATGTTGGAGTAAATAAATATA 37579

Chimpanzee AAGTTATGATATTATGAGCAAGTATTATTTGTCATTGTTATGTTGGAGTAAATAAATATA 46500

Gorilla AAGTTATGATATTATGAGCAAGTATTATTTGTCATTGTTATGTTGGAGTAAATAAATATA 37558

************************************************************

Human CCTTAACGTTTGTAAAGATGTTGGAAGTATCATCTTGGCTCAAGAAGACTTAAAGAAAAA 37639

Chimpanzee TCTTAATGTTTGTAAAGATGTTGGAAGTATCATCTTGGCTCAAGAAGACTTAAAGAAAAA 46560

Gorilla CCTTAATGTTTGTAAAGATGTTGGAAGTATCATCTTGGCTCAAGAAGACTTAAAGAAAAA 37618

***** *****************************************************

Human ATGATGAGAAATGATTAACGTGGTGAGAGAACTTGGCATGAGGGCAGGTGAACAATTTTC 37699

Chimpanzee ATGATGAGAAATGATTAACGTGGTGAGAGAACTTGGCCTGAGGGCAGGTGAACAATTTTC 46620

Gorilla ATGATGAGAAATGATTAACGTGGTGAGAGAACTTGGCATGAGGGCAGGTGAACAGTTTTC 37678

************************************* **************** *****

Human CATTCCATTATTAGTCTTTTGTGTGGCTAAGCTTTTTTTTTTTTT----CCCATGGACAT 37755

Chimpanzee CATTCCATTATTAGTCTTTTGTGTGGCTAAGCTTTTTTTTTTTTTTTTTCCCATGGACAT 46680

Gorilla CATTCCATTATTAGTCTTTTGTGTGGCTAAGCNNNNNNTTTTTTT----CCCATGGACAT 37734

******************************** ******* ***********

Human CCCAAAATATTAGTTTTAAATGGTATTTGAAGTCATTCTCCTTTAAGAATTTTCCTCCCT 37815

Chimpanzee CCCAAAATATTAGTTTTAAATGGCATTTGAAGTCATTCTCCTTTAAGAATTTTCCTCCCT 46740

Gorilla CCCAAAATATTAGTTTTAAATTGTATTTGAAGTCATTCTCCTTTAAGAATTTTCCTCCCT 37794

********************* * ************************************

Human CCAGTGAGTAAAGAGGGGCTTAGAGGACATAAGTCACTTGCAAGTAACTTGCCTGTATTT 37875

Chimpanzee CCAGTGAGTAAAGAGGGGCTTAGAGGACATAAGTCACTTGCAAGTAACTTGCCTGTATTT 46800

Gorilla CCAGTGAGTAAAGAGGGGCTTAGAGGACATAAGTCACTTGCAAGTAACTTGCCTNNNNNN 37854

******************************************************

Human AAGGTCACAGTGTGATATCCTACCTTGTTTTAACCTGAATTGACTCTCCCTTGGCTGAGA 37935

Chimpanzee AAGGTCACAGTGTGATATCCTACCTTGTTTTAACCTGAATTGACTCTCCCTTAGCTGAGA 46860

Gorilla NNNNNCACCGTGTGATATCCTACCTTGTTTTAACCTGAATCGACTCTCCCTTAGCTGAGA 37914

*** ******************************* *********** *******

Human GAAGTGGACAGACTCCATTTTGGCTCCTTCACTTGCAGCCCCTTACCCACCCCCTTCCTC 37995

Chimpanzee GAAGTGGACAGACTCCATTTTGGCTCCTTCACTTGCAGCCCCTTACCCACCCCCTTCCTC 46920

Gorilla GAAGTGGACAGACTCCATTTTGCCTCCTTCACTTGCAGCCCCTTACCCACTCCCTTCCTC 37974

********************** *************************** *********

Human AAAGACTTAACTTGTGCAAGCTGACTCCCAGCACATCCAGGAATGCAATTACTGATAAGA 38055

Chimpanzee AAAGACTTAACTTGTGCAAGCTGACTCCCAGCACATCCAGGAATGCAATTACTGATAAGA 46980

Gorilla AAAGACTTACCTTGTGCAAGCTGACTCCCAGCACATCCAGGAATGCAATTACTGATAAGA 38034

********* **************************************************

Human TACTGTGGCAAGCTATATCCGCAGTTCCCAGGAATTCACCTGGTTGATAGTACTCAAAGC 38115

Chimpanzee TACTGTGGCAAGCTATATCCACAGTTCCCAGGAATTCACCTGGTTGATAGTACTCAAAGC 47040

Gorilla TACTGTGGCAAGCTATATCCGCAGTTCCCAGGAATTCACCTGGTTGATAGTACTCAAAGC 38094

******************** ***************************************

Human CCCAGTGTTTGTGTCCAGTTGATAGCGCCCAAAGCCCCCGCATCTATCACCTTGTGATAG 38175

Chimpanzee CCCAGTGTTTGTGTCCAGTTGATAGCGCCCAAAGCCCCCACATCTATCACCTTGTGATAG 47100

Gorilla CCCAGTGTTTGTGTCCAGTTGATAGCGCCCAAAGCCCCCGCATCTATCGCCTTGTGATAG 38154

*************************************** ******** ***********

Human ATTTAAAGCCCCTGCACCTGGACCTGTTTGTTTTCCCGTAACCGTTCGTCTTTTTAACTT 38235

Chimpanzee ATTAAAAGCCCCTGCACCTGGAACTGTTTGTTTGCCCGTAACCGTTCATCTTTTTAACTT 47160

Gorilla ATTTAAAGCCCCTGCACCTGGAACTGTTTGTTTTCCCGTAACCGTTCATCTTTTTAACTT 38214

*** ****************** ********** ************* ************

Human TTTTTGTCTGTTGTGCTTCTGTAAGATTGCTTCAGCTAGGCTCCCCCTTCCCTTTCTAAA 38295

Chimpanzee TTTTTGTCTGTTGTGCTTCTGTAAGATTGCTTCAGCTAGGCTCCCCCTTCCCTTTCTAAA 47220

Gorilla TTTTTGTCTGTTGTGCTTCTGTAAGATTGCTTCAGCTAGGCTCCCCCTTCCCTTTCTAAA 38274

************************************************************

Human CCAAAGTATAAAAGAAAATCTAGCCCCTTCTTTGGGGCAGAGAGAATTTTGAGCACTAGC 38355

Chimpanzee CCAAAGTATAAAAGAAAATCTAGCCCCTTCTTTGGGGCAGAGAGAATTTTGAGCACTAGC 47280

Gorilla CCAAAGTATAAAAGAAAATCTAGCCCCTTCTTTGCGGCAGAGGGAATTTTGAGCACTAGC 38334

********************************** ******* *****************

Human CATCTCTCGGTCACCAGCAAATAAAGGACTGCTAAATTAGTCTCAAAGTGTGGCGTTTCT 38415

Chimpanzee CGTCTCTCGGTCACCAGCAAATAAAGGACTGCTAAATTAGTCTCAAAGTGTGGCGTTTCT 47340

Gorilla CATCTCTCGGTCACCAGCAAATAAAGGACTGCTAAATTAGTCTCAAAGTGTGGCGTTTCT 38394

* **********************************************************

Human CTACAACTCGCTCAGTTACAACATTTGGAAGTCCCGGCGAGATTTATTTGCCATGAGATG 38475

Chimpanzee CTACAACTCGCTCAGTTACAACATTTGGAAGTCCCGGCGAGATTTATTTGCCATCAGATG 47400

Gorilla CTACAACTCGCTCAGTTACAACATTTGGAAGTCCCGGCGAGATTTATTTGCCATCAGATG 38454

****************************************************** *****

Human AGTGCTGAATTCATTCCAGGCTCCCCTGGACAGACAGTGGTCTTATAGGGGAGGTGCCAC 38535

Chimpanzee AGTACTGAATTCATTCCAGGCTCCCCTGGACAGACAGTGGTCTTATAGGGGAGGTGCCAC 47460

Gorilla AGTGCTGAATTCATTCCAGGCTCCCCTGGACAGACAGTGGTCTTATAGGGGAGGTGCCAC 38514

*** ********************************************************

Human CTGAAGACTTTCCAAGGCACCATAGGCCACCGTATTCCGGAGGGGGAACGGATCGACTGC 38595

Chimpanzee CTGAAGACTTTCCAAGGCACCATAGGCCACCGTATTCCGGAGGGGGAACGGATCGACTGC 47520

Gorilla CTGAAGACTTTCCAAGGCACCATAGGCCACCGTATTCCNNNNNGGGAANNNNNNNACTGC 38574

************************************** ***** *****

Human TGGTGTGTGCCCACCAAATTCAACTCCTGAGTCCTCAGTCTCTGGTCCTGGGACGGTAAG 38655

Chimpanzee TGGTGTGTGCCCACCAAATTCAACTCCTGAGTCCTCAGTCTCTGGTCCTGGGAAGGTAAG 47580

Gorilla TGGTGCATGCCCACCAAATTCAACTCCTGAGTCCTCAGTCTCTGGTCCTGGGAAGGTAAG 38634

***** ********************************************** ******

Human TCAGATCTGACTCTGTTTCTCTGGGAGGGAAATGGCCCTATTGAGGGCCCTCCCTCAGAC 38715

Chimpanzee TCAGATCTGACTCTGTTTCTCTGGGAGGGAAATGGCCCTATTGAGGGCCCTCCCTCAGAC 47640

Gorilla TCAGATCTGACTCTGTTTCT-TGGGAGGGAAATAGCCCTATTGAGGGCCCTCCCTCAGAC 38693

******************** ************ **************************

Human TCTGTCCACACTCCAGGACACTGGAGGGCAAAGTCCTGGTTTCTGTCAGGCTTCTCTGTT 38775

Chimpanzee TCTGTCCACACTCCAGGACACTGGAGGGCAAAGTCCTGGTTTCTGTCAGGCTTCTCTGTT 47700

Gorilla TCNNNCCACACTCCAGGACACTGGAGGGCAAAGTCCTGGTTTCTNNNAGGCTTCTNTGTT 38753

** *************************************** ******** ****

Human AAGACTCTCACACTCCCTCTCTGTCTCTTCTTCTTCTCTCATTCAGGTCTCAAGGAGACC 38835

Chimpanzee AAGACTCTCACACTCCCTCTCTGTCTCTTCTTCTTCTCTCATTCAGGTCTCAAGGAGACC 47760

Gorilla AAGACTCTCACACTCCCTCTCTGTCTCTTCTTCTTCTCTCATGGAAGTCTCAAGGAGNNN 38813

****************************************** * ***********

Human CCTGTTTAAATGGGTATGAAAAATTATAATAAACTCTGAAGGAGTGAGTGAGTGAATGTG 38895

Chimpanzee TCTGTTTAAATGGGTATAAAAAATTATAATAAACTCTGAAGGAGTGAGTGAGTGAATGTG 47820

Gorilla NCTNNNNNNNNNNNNNNNNNNNNNTATAATAAACTCTGAAGGAGTGAGTGAGTGAATGTG 38873

** ************************************

Human GAGTTCAAGGGCTTTTGCTTGAATTTCAAGTTTGTAGCTCCATGGCAAAAGCTACAGAGT 38955

Chimpanzee GAGTTCAAGGGCTTTTGCTTGAATTTCAAGTTTGTAGCTCCATGGCAAAAGCTACAGAGT 47880

Gorilla GAGTTCAAGGGCTTTTCCTTGAATTTCAAGTTTGTAGCTCCATGGCAAAAGCTACAGAGT 38933

**************** *******************************************

Human TTGAGTGGGCCCTCACCTGTGGTTCCGGGTGACCTCATAAGGCTTAGGGTAGCAAAGGGC 39015

Chimpanzee TTGAGTGGGCCCTCACCTGCGGTTCCAGGTGACCTCATAAGGCTTAGGGTAGCAAAGGGC 47940

Gorilla TTGAGTGGGCCCTCACCTGCGGTTCCGGGTGACCTCATAAGGCTTAGGGTAGCAAAGGGC 38993

******************* ****** *********************************

Human ATAGCTCGACCTGAGCTGGGGGTTTATACTGGCCTGCCAATGTCAAGAGGAGCCTAAGTC 39075

Chimpanzee ATAGCTCGACCTGAGCTGGGGGTTTATACTGGCCTGCCAATGTCAAGAGGAGCCTAAGTC 48000

Gorilla ATAGCTCGACCTGAGCTGGGGGTTTATACTGGCCTGCCAATGCCAAGAGGAGCCTAAGTC 39053

****************************************** *****************

Human CCTGCAAGGGGAGTGGCCAGGCAGGCATCTGACTAATCACATCACAGAAGCCCCTCATCT 39135

Chimpanzee CCTGCAAGGGGAGTGGCCAGGCAGGCATCTGACTGATCACATCACAGAAGCCCCTCATCT 48060

Gorilla CCTGCAAGGGGAGTGGCCAGGCAGGCATCTAACTAATCACATCACAGAAGCCCCTCATCT 39113

****************************** *** *************************

Human TGTCTGTTTATAAAAACTTTCATAACTGTTTATATACCCCAGTGTCTATTCTCCTGTCTG 39195

Chimpanzee TGTCTGTCTATAAAAACTTTCATAACTGTTTATATACCCCAGTGTCTATTCTCCTGTCTG 48120

Gorilla TGTCTGTCTATAAAAACTTTCAAAACTGTTTATATACCCCAGTGTCTATTCTCCTGTCTG 39173

******* ************** *************************************

Human GTGTTTGCCTAAGTTTCACATGTCAGGTCTTCAATACTGCCCAAGATGACTGGGCAAGGA 39255

Chimpanzee GTGTTTGCCTAAGTTTCACATGTCAGGTCTTCAGTACTGCCCAAGATGACTGGGCAAGGA 48180

Gorilla GTGTTTGCCTAAGTTTCACATGTCAGGTCTTCAATACTGCCCAAGATGACTGGGCAAGGA 39233

********************************* **************************

Human CTTCTTCAAGGTCGTTAGTACAGATTTTCTATCCCAGGAGTTCAAATCTTTCATCAGTCA 39315

Chimpanzee CTTCTTGAAGGTCCTTAGTACAGATTTTCTATCCCAGGAGTTCAAATCTTTCATTAGTCA 48240

Gorilla CTTCTTCAAGGTCCTTAGTACAGATTNNNNNNNNNANNNGTTCAAATCTTTCATTAGTCA 39293

****** ****** ************ * *************** *****

Human TTTGGGCTGGCCATCCGAGTCCTGTCTTTTCTGTCGGAAAAAAGTCAGGTGTTGTTATGG 39375

Chimpanzee TTTGGGCTGGCCATCCCAGTCCTGTCTTTTCTGTCAGAAAAAAATCAGGTGTTGTTATGG 48300

Gorilla TTTGGGCTGGCCATCNNNNNNNNNNCTTTTCTGTCNNNNNAAAATCAGGTGTTGTTATGG 39353

*************** ********** *** ****************

Human GGAGGTGTATGGGAAACAGTCTCTCGTTTGGGATTTCTGGCACCATAAAGGTTGCTGGCA 39435

Chimpanzee GGAGGTGTATGGGAAACAGTCTCTCGTTTGGGATTTCTGGCACCATAAAGGTTGCTGGCA 48360

Gorilla GGAGGTGTATGGGAAACAGTCTCTCGTTTGGGATTTCTGGCACCATAAAGGTTGCTGGCA 39413

************************************************************

Human TTTGGATTGCCATACCTCAAACCCCAGTGACTGAAACACCTCCTCCTTAGATCAGTGGGG 39495

Chimpanzee TTTGGATTGCCATACCCCAAACCCCAGTGACTGAAACACCTCCTCCTTAGATCAGTGGGG 48420

Gorilla TTTGGATTGCCATACCCCAAACCCCAATGACTGAAACACCTCCTCCTTAGATCAGTGGGG 39473

**************** ********* *********************************

Human GATTCAAAATAGCCACCCTGCAGATTTCCTTGCTCACCTTTTCTGTCATCCTGTAACTTT 39555

Chimpanzee GATTCAAAATAGCCACCCTGCAGATTTCCTTGCTCACCTTTTCTGTCATCCTGTAACTTT 48480

Gorilla GATTCAAAATAGCCACCCTGCAAATTTCCTTGCTCACCTTTTCTGTCATCCTGTAACTTT 39533

********************** *************************************

Human TCCCATGCCCTTGGATAAGGCACTGTGCAGAGAAACCTACGCCTGTACTGTCTTATTCCA 39615

Chimpanzee TCCCATGCCCTTGGATAAGGCACTGTGCAGAGAAACCTACGCCTGTACTGCCTTATTCCA 48540

Gorilla TCCCATGCCCTTGGATAAGGCACTGTGCAGAGAAACCTACGCCTGTACTGCCTTATTCCA 39593

************************************************** *********

Human TCTGGACTCTTATTCTATCCCTCTGTAGCTACTCTCCTACCTTAGGAAAGATCCAAGTGG 39675

Chimpanzee TCTGGACTCTTATTCTATCCCTCTGTAGCTACTCTCCTACCTTAGGAAAGATCCAAGTGG 48600

Gorilla TCTGGACTCTTATTCTATCCCTCTGTAGCTACTCTCCTACCTTAGGAAAGATCCAAGTGG 39653

************************************************************

Human CCCCTTTCCTACTCATCCCCATCCCTTACCCCGTACATTTCATTTTCCTGTGTCACAGCA 39735

Chimpanzee CCCCTTTCCTACTCATCCCCATCCCTTACCCCGTACATCTCATTTTCCTGTGTCACAGCA 48660

Gorilla CCCCTTTCCTACTCATCCCCATCCCTTACCCCGTACATCTCATTTTCCTGTGTCACAGCA 39713

************************************** *********************

Human AGTCCAGCACCTCCAAGACTTGGCTCTGCTCACCCTCCTAAAACCCTTAAAAGAAAAAGC 39795

Chimpanzee AGCCCAGCACCTCCAAGACTTGGCTCTGCTCACCCTCCTAAAACCCTTAAAAGAAAAAGC 48720

Gorilla AGTCCAGCACCTCCAAGACTTGGCTCTGCTCGCCCTCCTAAAACCCTTAAAAGAAAAAGC 39773

** **************************** ****************************

Human CAAGTTTGAACTGTTTGCCTTTGAGTCATGAAGACACCAAAAATATTCAGGCTATAAGTC 39855

Chimpanzee CAAGTTTGAACTTTTTGCCTTTGAGTCATGAAGACACCAAAAATATTCAGGCTATAAGTC 48780

Gorilla CAAGTTTGAACTTTTTGCCTTTGAGTCATGAAGACACCAAAAATATTCAGGCTATAAGTC 39833

************ ***********************************************

Human AAAAAGGAAGGAGGGATCATGTAGGTCCCACAGGCCTCAGACCCACCTCTTGTCCTCTCC 39915

Chimpanzee AAAAAGGAAGGAGGGAACATGTAGGTCCCACAGGCCTCAGACCCACCTCTTGTCCTCTCC 48840

Gorilla AAAAAGGAAGGAGGGATCATGTAGGTCCCACAGGCCTCAGACCCACCTCTTGTCCTCTCC 39893

**************** *******************************************

Human CTAGATCTCAAAACCTAAAGAGAAAGAGCTTATGTGGCAAGAAGTGTTGGCTATAGTTGC 39975

Chimpanzee CTAGATCTCAACACCTAAAGAGAAAGAGCTTATGTGGCAAGAAGTGTTGGCTATAGTTGC 48900

Gorilla CTAGATCTCAAAACCTAAAGAGAAAGAGCTTATGTGGCAAGAAGTGTTGGCTATAGTTGC 39953

*********** ************************************************

Human TTTCCTACTTCTTCTGGTCGTAATATTTCTGTTCTTCTGATACTACGGGCCCCCAGAGAG 40035

Chimpanzee TTTCCTACTTCTTCTGGTCGTAATATTTCTGTTCTTCTGATACTACAGGCCCCCAGAGAG 48960

Gorilla TTTCCTACTTCTTCTGGTTGTAATATTTCTGTTCTNNNNATACTACAGGNNNNNNNNNNN 40013

****************** **************** ******* **

Human TGAATTGCTCTGTCCATGCTGGATTTAATATTTCTGCTCAATCCTTGTTAAATTGCTTCC 40095

Chimpanzee TGAATTTCTCTGTCCATGCTGGATTTAATATTTCTGCTCAATCCTTGTTAAATTGCTTCC 49020

Gorilla NNNNNNNNNNNNTCCATGCTGGATTTAATATTTCTGCTCAATCCTTGTTCAATTGCTTCC 40073

************************************* **********

Human AGAATGGGAAGCTCTTCTTCCTGGCCTCATAGAGATTGGAGCCCTCTCCAATGTATGTTA 40155

Chimpanzee AGAATGGGAAGCTCTTCTTCCTGGCCTCATAGAGATTGGAGCCCTCTCCAATGTATGTTA 49080

Gorilla AGAATGGGAAGCTCTTCTTCCTGGCCTCATAGAGATTGGAGCCCTCTCCAATGTATGTTA 40133

************************************************************

Human CAAAATTTCTCCCTGGGCTTCTCAGAGGATTATGGGGTTTGCCTTAAAAAAGGCAAATTC 40215

Chimpanzee CAAAATTTCTCCCTGGGCTTCTCAGAGGATTATGGGGTTTGCCTTAAAAAAGGCAAATTC 49140

Gorilla CAAAATTTCTCCCTGGGCTTCTCAGAGGATTATGGGGTTTGCCTTAAAAAAGGCAAATTC 40193

************************************************************

Human TGGACACTCTGTGAAGTAGAACGGCTACAGTTTGGAACCGGGTGGCCCCCAGAAGGGTCA 40275

Chimpanzee TGGACACTCTGTGAAGTAGAACAGCTACAGTTTGGAACCGGGTGGCCCCCGGAAGGGTCA 49200

Gorilla TGGACACTCTGTGAAGTAGAACGGCTACAGTTTGGAACTGGGTGTCCCCCAGAAGGGTCA 40253

********************** *************** ***** ***** *********

Human CTGAATGTGTGTGGCAGTTCATTGCTGGAACTCCCAGTCACCCTGATCAGTTTCCCTACA 40335

Chimpanzee CTGAATGTGTGTGGCAGTTCGTTGCTGGAACTCCCAGTCACCCTGATCAGTTTCCCTACA 49260

Gorilla CTGAATGTGTGTGGCAGTTCGTTGCTGGAACTCCCAGTCACCCTGATCAGTTTCCCTACA 40313

******************** ***************************************

Human TTGATCAATGGCTGAGCTTGGTCTGGGGCCCTTCCCCTTGGCTCCACTCATGTGCTGTTC 40395

Chimpanzee TTGATCAATGGCTGAGCTTGGTCTGGGGCCCTTCCCCTTGGCTCCACTCATGTGCTGTTC 49320

Gorilla TTGATCAATGGCTGAGCTTGGTCTGGGGCCCTTCCCCTTGGCTCCACTCATGTGCTGTTC 40373

************************************************************

Human ATAATCCTACCTCCAAGGTGCTTTTGAGCCAGGCCTCACTGTCATTCTGACCCTCAGCCC 40455

Chimpanzee ATAATCCTACCTCCAAGGTCCTTTTGAGCCAGGCCTCACTGTCATTCTGACCCTCAGCCC 49380

Gorilla ATAATCCTACCTCCAAGGTCCTTTTGAGCCAGGCCTCACTGTCATTCTGACCCTCAGCCC 40433

******************* ****************************************

Human CCTCAGCTCCTCCTGTACTGCCTTTTTCTGAAGAAGAGGAAAGTTTTTCTCACGCATTTC 40515

Chimpanzee CCTCAGCTCCTCCTGTACTGCCTTTTTCTGAAGAAGAGGAAAGTTTTTCTCACGCATTTC 49440

Gorilla CCTCAGCTCCTCCTGTACTGCCTTTTTCTGAAGAAGAGGAAAGTTTTTCTCATGCATTTC 40493

**************************************************** *******

Human CACCACCCCATAACCCTCCTGCTCCACCAAAATCTTGCCTCGTTTCTTTGACTACATCCC 40575

Chimpanzee CACCACCCCATAACCCTCCTGCTCCACCAAAATCTTGCCTCGTTTCTTTGACTACATCCC 49500

Gorilla CACCACCCCATAACCCTCCTGCTCCACCAAAATCTTGCCTCGTTTCTTTGACTACATCCC 40553

************************************************************

Human CTGTGGCCTCTCTGCCTACAGCCTCCTGATTATGACCGCTGGAGAATAAAGCTAATCCTC 40635

Chimpanzee CTGTGGCCTCTCTGCCTACAGCCTCCTGATTATGACCGCTGGAGAATAAAGCTAATCCTC 49560

Gorilla CTGTGGCCTCTCTGCCTACAGCCTCCTGATTATGACCGCTGGAGAATAAAGCTAATCCTC 40613

************************************************************

Human TCCTATTGTATCTACAGATTGTGTTTCCTTGAGTCTGGGCCAAGTCAAAATCCCCAGGAC 40695

Chimpanzee TCCTATTGTATCTACAGATTGTGTTTCCTTGAGTCTGGGCCGAGTCAAAATCCCCAGGAC 49620

Gorilla TCCTATTGTATCTACAGATTGTGTTTCCTTGAGTCTGGGCTGAGTCAAAATCCCCAGGAC 40673

**************************************** ******************

Human TAGCAAAGCATCATCCACTGTTAGTTGTAGAACTCCTGGTCACTGCCCTGCCAGTCCAGG 40755

Chimpanzee TAGCAAAGCATCATCCACTGTTAGTTGTAGAACTCCTGGTCACTGCCCTGCCAGTCCAGG 49680

Gorilla TAGCAAAGCATCATCTGCTGTTAGTTGTAGAACTCCTGGTCACTGCCCTGCCAGTCCAGG 40733

*************** *******************************************

Human TAAAAAAAACTATCCTATGAGTCAGCGGGCTAGAGAGGGAATCAATCCCCATATTCAGGG 40815

Chimpanzee TAAAAAAAACTATCCTATGAGTCAGCGGGCTAGAGAGGGAATCAATCCCCATATTCAGGG 49740

Gorilla TNGAAAANNCTATCCTATGAGTCAGCTGGCTAGAGAGGGAATCAATCCCCATATTCAGGG 40793

* **** ***************** *********************************

Human GCTATTACAAGCTGGTATACTCACAGCGTGTTAGTTGGCCTGGAATACTCCATTTTT-GT 40874

Chimpanzee GCTATTACAAGCTGGTATACTCACAGCGTGTTAGTTTGCCTGGAATACTCCATTTTTTGT 49800

Gorilla GCTATTACAAGCTGATATACTCACAGCGTGTTAGTTTGCCTGGAATACTCCATTTTT-GT 40852

************** ********************* ******************** **

Human CGGTCCAGAAACCTGGAACAAATAATTACCAGCCTGTACAGGACTTGCAGGAAGTTAACA 40934

Chimpanzee CGGTCCAGAAACCTGGAACAAATAATTACCAGCCTGTACAGGACTTGCAGGAAGTTAACA 49860

Gorilla CAGTCCAGAAACCTGGAACAAATAATTACCAGCCTGTACAGGACTTGCAGCAAGTTAACA 40912

* ************************************************ *********

Human AGTGGACAGTCACTGTCCATCCAACTGTCCCTAACCCTTATACTTTATTCAGCCTGCTTT 40994

Chimpanzee AGTGGACAGTCACTGTCCATCCAACTGTCCCTAACCCTTATACTTTATTCAGCCTGCTTC 49920

Gorilla AGTGGACAGTCACTGTCCATCCAACTGTCCCTAACCCTTACACTTTATTCAGCCTGCTTC 40972

**************************************** ******************

Human TGCCAGAACATACAGTATGTGCTGTCCTGGACTTAAAAGAGGCTTTCTTTGCTATTTCTC 41054

Chimpanzee TGCCAGAACATACAGTATGTGCTGTCCTGGACTTAAAAGAGGCTTTCTTTGCTATTTCTC 49980

Gorilla TGCCAGAACATCCAGTATGTGCTGTCCTTGACTTAAAAGAGGCTTTATTTGCTATTTCTC 41032

*********** **************** ***************** *************

Human TGGTCCCCAAATGCCAATCTATCTTTGCTTTTGAATGGACAGATCCTGGCTTGGGAGACA 41114

Chimpanzee TGGTCCCCAAATGCCAACCTATCTTTGCTTTTGAATGGACAGATCCTGGCTTGGGAGACA 50040

Gorilla TGGTCCCCAAATGCCAACCTATCTTTGCTTTTGAGTGGACAGATCCTGGCTTGGGAGACA 41092

***************** **************** *************************

Human CCACTCAATTAACCTGGACTCAGCTACCCCAGGGTTTTAAAAATTCCCCCACACTTTTTG 41174

Chimpanzee CCACTCAATTAACCTGGACTCAGCTACCCCAGGGTTTTAAAAATTCCCCCACACTTTTTG 50100

Gorilla CCACTCAATTAACCTGGACTCAGCTACCCCAGGGTTTTAAAAATTCCCCCACACTTTTTG 41152

************************************************************

Human GAGAAGCCCTCCAACAAGATCTCATACCATTCTGAGCCAGTCACCCTAACTGCACTTTTC 41234

Chimpanzee GAGAAGCCCTCCAACAAGATCTCATACCATTCTGAGCCTGTCACCCTAACTGCACTTTTC 50160

Gorilla GAGAAGCCCTCCAACAAGATCTCATACCATTCTGAGCCAGTCACCCTAACTGCACTTTTC 41212

************************************** *********************

Human TCCAGTACGTGGATGACCTTTTATTAGCTGCTAAAACTACTGACAGCCGCCTGCAATATA 41294

Chimpanzee TCCAGTACGTGGATGACCTTTTATTAGCTGCTAAGACTACTGACAGCCGCCTGCAATATA 50220

Gorilla TCCAGTACGTGGATGACCTTTTATTAGCTGCTAAAACTACTGACAGCCGCCTGCAATATA 41272

********************************** *************************

Human CTAGAGACCTGCTTTGCCTTCTCCAGAAACTTGGGTACTGGGTTTCAGCTAAGAAGACCA 41354

Chimpanzee CTCGAGACCTGCTTTGCCTTCTCCAGAAACTTGGGTACTGGGTTTCAGCTAAGAAGTCCA 50280

Gorilla CTAGAGACCTGCTTTGCCTTCTCCAGAAACTCGGGTACTGGGTTTCAGCTAAGAAGTCCA 41332

** **************************** ************************ ***

Human AGCTTTATCTCCCCAGGGTTTCCTATCTGGGGTATGAGATAGACAAGGGAAAAAGGGCAC 41414

Chimpanzee AGCTTTATCTCCCCAGGGTTTCCTATCTGGGGTATGAGATGGACAAGGGAAAAAGGGCAC 50340

Gorilla AGCTTTATCTCCCCAGGGTTTCCTACCTGGGGTATGAGACAGACAAGGGAAAAAGGGCAC 41392

************************* ************* *******************

Human TCACCAGTGCTCAAAAGGAAACCATCCTGTGGATCCCCACTCCCATCACCAAGAGACAGG 41474

Chimpanzee TCACCAGTGCTCAAAAGGAAACCATCCTGTGGATCCCCACTCCCATCACCAAGAGACAGG 50400

Gorilla TCACCAGTGCTCAAAAGGAAACCATCCTGCAGATCCCCACTCCCATCACCAAGAGACAGG 41452

***************************** *****************************

Human TACATGAATTCCTGGGGTCTGTAGGATACTGTCATCTATGGATATCAGGGTTTGTGGAAA 41534

Chimpanzee TACATGAATTCCTGGGGTCTGTAGGATACTGTCATCTATGGATATCAGGGTTTGCGGAAA 50460

Gorilla TACATGNNNNNNTNNNNNNNNNNNNNNACTGTCATCTATGGATATCAGGGTTTGCAGAAA 41512

****** * *************************** ****

Human TCACCAAGCCCCTAAACTCTGTTATAGGAGAAAATGACCTGCTAGCTTGGACTTACACAG 41594

Chimpanzee TCACCAAGCCCCTAAACTCTGTTATAGGAGAAAATGACCTGCTAGCTTGGACTTACACAG 50520

Gorilla TCACCAAGCCCCTAAACTCTGTTATAGGAGAAAATGACCTGCTAGCTTGGACTTACACAG 41572

************************************************************

Human AGGAACAGGCTTTTCAATACTGGAAAAAACCATTAACTGAGGACCCTGCTCTAGCCCTCC 41654

Chimpanzee AGGAACAGGCTTTTCAATACTGGAAAAAACCATTAACTGAGGACCCTGCTCTAGCCCTCC 50580

Gorilla AGGAACAGGCTTTTCAATACTGGAAAAAACCATTAACTGAGGACCCTGCTCTAGCCCTCC 41632

************************************************************

Human CAAATAATCTCAAAACCATTTCACCTTTTTGTTCATGAAAGCCAGGGAGTCACTAGACGG 41714

Chimpanzee CAAATAATCTCAAAACCATTTCACCTTTTTGTTCATGAAAGCCAGGGAGTCACTAGACGG 50640

Gorilla CAAATAATCTCAAAACCATTTCACCTTTTTGTTCATGAAAGCCAGGGAGTCACTAGACGG 41692

************************************************************

Human GTACTCACTCAAACTTTGGGGCCATGGTGATGCCCAGTGGCCTATTTGTCTAAGAAACTG 41774

Chimpanzee GTACTCACTCAAACTTTGGGGCCATGGTGATGCCCAGTGGCCTATTTGTCTAAGAAACTG 50700

Gorilla GTACTCACTCAAACTTTGGGGCCATGGTGATGCCCAGTGGCCTATTTGTCTAAGAAACTG 41752

************************************************************

Human GACCCCTGGACCCTGTGGCCTCCAGGTGGCCAAGATGTCTGCGAGCCATAGCAGCCACAG 41834

Chimpanzee G--------ACCCTGTGGCCTCCAGGTGGCCAAGATGTCTGCGAGCCATAGCAGCCCCAG 50752

Gorilla GACNNNNNNNNNNNGTGGCCTCCAGGTGGCCAAGATGTCTGCGAGCCATAGCAGCCGCAG 41812

* ****************************************** ***

Human CAAGCCTGGTTCAGGAGTCTGATAAACTGACTCTAGGTAAAAATTTAACCCTTATGGCTC 41894

Chimpanzee CAAGCCTGGTTCAGGAGTCTGATAAACTGACTCTAGGTAAAAATTTAACCCTTATGGCTC 50812

Gorilla CAAGCCTGGTTCAGGAGTCTGATAAACTGACTCTAGGTAAAAATTTAACCCTTATGGCTC 41872

************************************************************

Human CTCGTGCCATAGAGACATTGCTACAAAGTGCTTCTGCCAAATAGATGTCGAACGCTTGCA 41954

Chimpanzee CTCGTGCCATAGAGACATTGCTACAAAGTGCTTCTGCCAAATAGATGTCGAACGCTTGCA 50872

Gorilla CTCATGCTATAGAGACATTGCTACAAAGTGCTTCTGCCAAATAGATGTCAAACGCTTGCA 41932

*** *** ***************************************** **********

Human TCCTGCAGTATCAAAATTTACTGTTAGATCAGCCTCGTGTAACTTTCTCTCCCACAAGGT 42014

Chimpanzee TCCTGCAGTATCAAAATTTACTGTTAGATCAGCCTCGTTTAACTTTCTCTCCCACAAGGT 50932

Gorilla TCCTGCAGTATCAAAATTTACTGTTAGATCAGNNNNNNNNNNNNNNNNNNNNCACAAGGT 41992

******************************** ********

Human GTTTAAATCCATCTACCTTGCTCCCTGATCCAGACCTTACCACACCTATCCATGACTGTG 42074

Chimpanzee GTTTAAATCCATCTACCTTGCTCCCTGATCCAGACCTTACCACACCTATCCATGACTGTG 50992

Gorilla GTTTAAATCCATCTACCTTGCTCCCTAATCCAGACCCTACCACACCTATCCATGACTGTG 42052

************************** ********* ***********************

Human AGGAACTGTTAGTGACTATAGAAATTGGCTGACCTGATCTCCAAGATGTGCCTTTAAAGG 42134

Chimpanzee AGGAACTGTTAGTGACTATAGAAATTGGCTAACCTGATCTCCAAGATGTGCCTTTAAAGG 51052

Gorilla AGGAACTGTTAGTGACTATAGAAATTGGCTGACCTGATCTCCAAGATGTGCCTTTAAAGG 42112

****************************** *****************************

Human AGGCAGACACCACCACATTTACAGAGAGTAGCAGCTTCCTCAAACAAGGAGTATGAAAGC 42194

Chimpanzee GGGCAGACACCACCACATTTACAGAGAGTAGCAGCTTCCTCAAACAAGGAGTATGAAAGG 51112

Gorilla AGGCAGACACCACCACATTTACAGAGAGTAGCAGCTTCCTGAAACAAGGAGTATGAAAGG 42172

*************************************** ******************

Human CTGCTCCAGCCATTACTATGGAAACAGATATACTGTGGGCCCAGGTGCTGCTGGCAGGCA 42254

Chimpanzee CTGCTCCAGCCATTACTATGGAAACAGATATACTGTGGTCCCAGGTGCTGCTGGCAGGCA 51172

Gorilla CTGCTCCAGCCATTACTATGGAAACAGATATACTGTGGGCCCAGGTGCTGCTGGCAGGCA 42232

************************************** *********************

Human ACTTGGCAAAGAGGGCTGATTAGTTGCCCTCACTCAAGCTTCCGATAGGGCAAAAATAAA 42314

Chimpanzee ACTTGGCAAAGAGGGCTGATTAGTTGCCCTCACTCAAGCCTCCGATAGGGTAAAAATAAA 51232

Gorilla ACTTGGCAAGGAGGGCTGATTAGTTGCCCTCACTCAAGCCTCTGATAGGGTAAAAATAAA 42292

********* ***************************** ** ******* *********

Human TGCATTAACATCTACACTGACAGCAGATATACGTTTGCTACTGTCCATGTACACAGAGCC 42374

Chimpanzee TGCATTAACATCTACACTGACAGCAGATATACGTTTGCTACTGTGCATGTACACAGAGCC 51292

Gorilla TGCATTAACATCTACACTGACAGCAGATATACGTTTGCTACTGTGCATGTACACAGAGCC 42352

******************************************** ***************

Human ATCTATCAGGAGCATGGGCTACTCACCTCAGAAGGAAAAACTATCAAAAATAAAGAAGAA 42434

Chimpanzee ATCTATCAGGAGCATGGGCTACTCCCCTCAGAAGGAAAAACTATCAAAAATAAAGAAGAA 51352

Gorilla ATCTATCAGAAGCATGGGCTACTCACCTCAGAAGGAAAAACTATCAAAAATAAAGAAGAA 42412

********* ************** ***********************************

Human ATTTTGGCCTTGCTTGAAGCTGTTTGGTTCCCTCAACAGGTGGCTGTAATTCACTGCGAA 42494

Chimpanzee ATTTTGGCCTTGCTTGAAGCTGTTTGGTTCCCTCAACAGGTGGCTGTAATTCACTGCGAA 51412

Gorilla ATGTTGGCCTTGCTTGAAGCTGTTTGGTTCCCTCAACAGGTGGCTGTAATTCACTGTGAA 42472

** ***************************************************** ***

Human GGACAACAAAAAGAAGATATGGTCATTGCTTGTGGTAACCAAAGAGCAGATTCTGCAGCT 42554

Chimpanzee GGACAACAAAAAGAAGATATGGTCGTTGCTTGTGGTAACCAAAGAGCAGATTCTGCAGCT 51472

Gorilla GGACAACAAAAAGAAGATATGGTCGTTGCTTGTGGTAACCAAAGAGCAGATTCTGCAGCT 42532

************************ ***********************************

Human AAAAAGACAGCTCAGTTTCCAGTCATGCCTTTGACTCTGCTGCCCTCTATATCCTTTCTG 42614

Chimpanzee AAAAAGACAGCTCAGTTTCCAGTCATGCCTTTGACTCTGCTGCCCTCTATATCCTTTCGG 51532

Gorilla AAANNGACAGCTCAGTTTCCAGTCATGCCTTTGACTCTGCTGCCCTCTATATCCTTTCTG 42592

*** ***************************************************** *

Human CAGCCTGACTTTCGAGACCACCCAGAATACTCCCTAGAGGAGGAAAAAGAGGCTTCAGAT 42674

Chimpanzee CAGCCTGACTTTCGAGACCACCCAGAATACTCCCTAGAGGAGGAAAAAGAGGCTTCAGAT 51592

Gorilla CAGCCTGACTTTCGAGACCACCCAGAATACTCCCTAGAGGAGGAAAAAGAGGCTTCAGAT 42652

************************************************************

Human CTTCAGGCCAGTAAAAATCAGGAAGGTTGGTGAATTCTTCCTGATCCCAGAATCTTCATG 42734

Chimpanzee CTTCAGGCCAGTAAAAATCAGGAAGGTTGGTGAATTCTTCCTGATCCCAGAATCTTCATG 51652

Gorilla CTTCAGGCCAGTAAAAATCAGGAAGGTTGGTGAATTCTTCCTGATCCCAGAATCTTCATG 42712

************************************************************

Human CCCCAAGTCCTCGGGGAAACTTTAACCAGTCATCTGCATTCTACCACACATTTGAGAGCA 42794

Chimpanzee CCCCAAGTCCTCGGGGAAACTTTAATCCGTCATCTGCATTCTACCACACATTTGAGAGCA 51712

Gorilla CCCCAAGTCCTGGGGGAGACTTTAATCAGTCATCTGCATTCTACCACACATTTGAGAGCA 42772

*********** ***** ******* * ********************************

Human ATAAAACTGGCCCAGCTTCTAAAGAGCCATTTCAAGATCCTCCACCTTCAGGGCTTAGCT 42854

Chimpanzee ATAAAACTGGCCCAGCTTCTAAAGAGCCATTTCAAGATCCTCCACCGTCAGGGCTTAGCT 51772

Gorilla ATANAACTGGCCCAGCTTCTAAAGAGCCATTTCAAGATCCTCCACCTTCAGGGCTTAGCT 42832

*** ****************************************** *************

Human AACCAAGCAGCCCTCTGGTGTATAGGTTGTGCTCAGGTAAATGACAAGGAAGGTCCTAAA 42914

Chimpanzee AACCAAGCAGCCCTCTGGTGTATAGGTTGTGGTCAGGTAAATGCCAAGGAAGGTCCTAAA 51832

Gorilla AACCAAGCAGCTCTCTGGTGTATAGGTTGTGCTCAGGTAAATGCCAAGGAATGTCCTAAA 42892

*********** ******************* *********** ******* ********

Human CCCAGCCCAGGCCACCGCCTCTGGGGAGACTCACCAGGAGAAAGGTGGGAATTGACTTTA 42974

Chimpanzee CCCAGCCCAGGCCACCGCCTCTGGGGAGACTCACCAGGAGAAAGGTGGGAATTGACTTTA 51892

Gorilla CCCAGCCCAGGCCACCGCCTCTGGGGAGATTCACCAGGAGAAAGGTGGGAATTGACTTTA 42952

***************************** ******************************

Human CAGAGATAAAACCACACTGGGCAGTGTATAAATACCTCCTGGTGCTAGTAGACACCTTTT 43034

Chimpanzee CAGAGATAAAACCACACCGGGCAGTGTATAAATACCTCCTGGTGCTAGTAGACACCTTTT 51952

Gorilla CAGAGATAAAACCACACCGGACAGTGTATAAATACCTCCTGGTGCTAGTAGACACCTTTT 43012

***************** ** ***************************************

Human CCAGATGGACTGAGGCATTTGCCACCAAAAACGAGACTGCCACCATGGTAGTTAAGCTAT 43094

Chimpanzee CCAGATGGACTGAGGCATTTGCCACCAAAAACGAGACTGCCACCATGGCAGTTAAGCTAT 52012

Gorilla CCAGATGGACTGAGGCATTTGCCACCAAAAACGAGATTGCCACCATGGTAGTTAAGCTAT 43072

************************************ *********** ***********

Human TACTCAATGAAATCACTCCTTGACATGGGCTGCCCGCTGCCATAGGGTCTGATAATGGAC 43154

Chimpanzee TACTCAATGAAATCATTCCTTGACATGGGCTGCCTGCTGCCATAGGGTCTGATAATGGAC 52072

Gorilla TACTCAATGAAATCATCCCTTGACATGGGCTGCCTGCTGCCATAGGGTGTGATAATGGAC 43132

*************** ***************** ************* ***********

Human CAGCCTTCACCTCATCTGTAGCTCAATCAGTTAGTGAGACATTAAACATTCAATGGAAAC 43214

Chimpanzee CAGCCTTCACCTCATCTGTAGCTCAATCAGTTAGTGAGACATTAAACATTCAATGGAAAC 52132

Gorilla CGGCCTTCACCTCATCTGTAGCTCAATCAGTTAGTGAGACATTAAACATTCAATGGAAAC 43192

* **********************************************************

Human TCCATTGTGCCTATCAACCCCAGAGCTCTAGGCAGGTAGAATGCATGAACTGCATCCTAA 43274

Chimpanzee TCCATTGTGCCTATCAACCCCAGAGCTCTAGGCAGGTAGAATGCATGAACTGCATCCTAA 52192

Gorilla TCCATTGTGCCTATCAACCCCAGAGCTCTAGGCAGGTAGAATGCATGAACTGCATCCTAA 43252

************************************************************

Human AAAGTAATCTTACAATATTAATCCTAGAGACCAGTGAGAATTGGGTAAAACTCCTTTCTC 43334

Chimpanzee AAAGTAATCTTACAATATTAATCCTAGAGACCAGTGAGAATTGGGTAAAACTCCTTTCTC 52252

Gorilla AAAGTAATCTTACAATATTAATCCTAGAGACCAGTGAGAATGGGGTAAAACTCCTTTCTC 43312

***************************************** ******************

Human TAGACCTTCTTAAAGTAAGATGCACCCCTTACCAGGCTAGGTTTTCACCTTTTGAAATCA 43394

Chimpanzee TAGACCTTCTTAAAGTAAGATGCACCCCTTACCAGGCTAGGTTTTCACCTTTTGAAATCA 52312

Gorilla TAGACCTTCTTAAAGTAAGATGCACCCCTTACCAGGCTAGGTTTTCACCTTTTGAAATCA 43372

************************************************************

Human TGTCTGGGAGGGCTCCTCCTACCTTGTCCAAACTAAGAGATACCCATTTAACAGAAATCT 43454

Chimpanzee TGTCTGGGAGGGCTCCTACTACCTTGTCCAAACTAAGAGATACCCATTTAGCAGAAATCT 52372

Gorilla TGTCTGGGAGGGCTCCTCCTACCTTGTCCAAACTAAGAGATACCCATTTAGCAGAAATCT 43432

***************** ******************************** *********

Human CACAAGCTAATTTGTTACAGTACCAGCAGTCTCTGCAACAGGTACAAGACATTATCCAGC 43514

Chimpanzee CACAAGCTAATTTGTTACAGTACCAGCAGTCTCTGCAACAGGTACAAGACCTTATCCAGC 52432

Gorilla CACAGGCTAATTTGTTACAGTACCAGCAGTCTCTCCAACAGGTATAAGACATTATCCAGC 43492

**** ***************************** ********* ***** *********

Human CACATGTCTGAGGAGCACATCCCAATCCAGTTCCCGACCACAAGGGGCTCTGCCACTCTT 43574

Chimpanzee CACATGTCTGAGGAGCACATCCCAATCCAGTTCCTGACCACAAGGGGCTCTGCCACTCTT 52492

Gorilla CACATGTCTGAGGAGCACATCCCAATCCAGTTCCTGACCACAAGGGGCTCTGCCACTNNN 43552

********************************** **********************

Human TCCAGCCAGGTGACCTGGTGTATGTTAAAAAGTTCCAGAAAGAAGGACTTACTCCTGCCT 43634

Chimpanzee TCCAGCCAGGTGACCTGGTGTATGTTAAAAAGTTCCAGAAAGAAGGACTTACTCCTGCCT 52552

Gorilla NNNNGCCAGNTGACCTGGTGTATGTTAAAAAGTTCCAGAAAGAAGGACTTACTCCTGCCT 43612

***** **************************************************

Human GAAAAGGACCTGATACTGTCATCCTCACCACACCAAGAGCTCTAAAAGTAGATAACATTC 43694

Chimpanzee GAAAAGGACCTGATACTGTCATCCTCACCACACCAAGAGCTCTAAAAGTAGATAACATTC 52612

Gorilla GAAAAGGACCTGATACTGTCATCCTCACCACACCAACAGCTCTAAAAGTAGATAACATTC 43672

************************************ ***********************

Human CTGCTTGGATTCATCACTCTTGCATCAAGAAGACTAACAAAACCCAGCAAGAAACATGGG 43754

Chimpanzee CTGCTTGGATACATCACTCTTGCATCAAGAAGACTAACAAAACCCAGCAAGAAACATGGG 52672

Gorilla CTGCTTGGATTCATCACTCTTGCATCAAGAAGACTAACAAAACCCNGCAAGAAACATGGG 43732

********** ********************************** **************

Human TCCCCAAGACTGGTAGAGGCCCCTTACAACTGCACCTAAGTCAAGTGAAGCCAGTAGATT 43814

Chimpanzee TCCCCAAGACTGGGAGAGGCACCTTACAACTGCACCTAAGTCAAGTGAAGCCAGGAGATT 52732

Gorilla TCCCCAAGACTGGGAGAGGCCCCTTACAACTGCACCTAAGTCAAGTGAAGCCAGTAGATT 43792

************* ****** ********************************* *****

Human AATTCTTTATCTCTTTTGTTTGTTTCTGCCTGTCGTGCCCTCTGCTTCTTCCTACTCTTT 43874

Chimpanzee AATTCTTTTTCTCTTTTGTTTGTTTCTGCCTGTCGTGCCCTCTGCTTCTTCCTACTCTTT 52792

Gorilla AATTCTTTATCTCTTTTGTTTGTTTCTGCCTGTTGTGCCCTCTGCTTCTTCCTACTCTCT 43852

******** ************************ ************************ *

Human ATTCCTGACTTCTTTCATGACAGGAAGTGTGTTTGCAAACACCACCTGGAAGTCGGGGAC 43934

Chimpanzee ATTCCTGACTTCTTTCATGACAGGAAGTGTGTTAGCAAACACCACCTGGAAGTCGGGGAC 52852

Gorilla ATTCCTGACTTCTTTCATGACAGGAAGTATGTTTGCAAACACCACCTGGAAGTCGGGGAC 43912

**************************** **** **************************

Human CTACAAGGAAGTCTCTTTGGCAGTCGATTTATGTGCTCTGTTTCCAGAGCCTGCTCATAC 43994

Chimpanzee CTACAAGGAAGTCTCTTTGGCAGTCGATTTATGTGCTCTGTTTCCAGAGCCTGCTCATAC 52912

Gorilla CTACAAGGAAGTCTCTTTGGCAGTCGATTTATGTGCTCTGTTTCCAGAGCCTGCTCATAC 43972

************************************************************

Human GTTCGAAGAGCAACACAATCTGCCAATCATAGGAGCAGGGAACATCGACCTTGCTGCAGA 44054

Chimpanzee GTTCGAAGAGCAACACAATCTGCCAATCATAGGAGCAGGGAACATCGACCTTGCTGCAGA 52972

Gorilla GTTCGAAGAGCAACACAATCTGCCAATCCTAGGAGCAGGGAACATCAACCTTGCTGCAGA 44032

**************************** ***************** *************

Human GTTTGGACACTCCAGAGGCTAGACTGGATGTGGAAGCTCCAAAGGTGCAGAAAAAGGACT 44114

Chimpanzee GTTTGGACACTCCAGAGGCTAGACTGGATGTGGGAGCTCCAAAGGTGCAGAAAAAGGACT 53032

Gorilla GTTTGGACACTCCAGAGGTTAGACTGGATGTGGAAGNNNNAAANNNNCNNNAAAAGGACT 44092

****************** ************** ** *** * *********

Human CCAGAACGTTGAACTTTACTTCTGTCCTGGAAATCACCTTGACTCTAGGTGTCAAGATTC 44174

Chimpanzee CCAGAACGTTGAACTTTACTTCTGTCCTGGAAATCACCCTGACTCTAGGTGTCAAGATTC 53092

Gorilla CCAGAACATTGACCTTTACTTCTGTCCTGGAAATCACCCTGACTCTAGGTGTCAAGATTC 44152

******* **** ************************* *********************

Human TTACCAATTTTTCTGCCCTGACTAGACATGTATAACTTTGGCCACCTACTCTGGGGGATC 44234

Chimpanzee TTACCAATTTTTCTGCCCTGACTAGACATGTATAACTTTGGCCACCTACTCTGGGGGATC 53152

Gorilla TTACCAATTTTTCTGCCCTGACTAGACATGTATAACTTTGGCCACCTACTCTGGGGGATC 44212

************************************************************

Human AATCCGACCTTCAACCTTTTCCATAGCTCATGCTTCCCATCCTAAACTGTGTGCAAGGGG 44294

Chimpanzee AATCCGACCTTCAACCTTTTCCATAGCTCATGCTTCCCATCCTAAACTGTGTGCAAGGGG 53212

Gorilla AATCCGACCTTCAACCTTTTCCATAGCTCATGCTTCCCATCCTAAACTGTGTGCAAGGGG 44272

************************************************************

Human AAATTGCAATCTTCTTACTATAACCATCCGTAACCCCAATTTAGCTCAATGGCATTATGG 44354

Chimpanzee AAATTGCAATCTTCTTACTATAACCATCCATAACCCCAATTTAGCTCAATGGCATTATGG 53272

Gorilla AAACTGCAATCTTCTTACTATAACCATCCATAACCCCAATTTAGCTCAATGGCATTATGG 44332

*** ************************* ******************************

Human CATGTCATGGGGATTAAGGCTTTATATCGCTGGATTTGATGTTGAAACTGTGTTCAGCAT 44414

Chimpanzee CATGTCATGGGGATTAAGGCTTTATATCGCTGGATTTGATGTTGAAACTGTGTTCAGCAT 53332

Gorilla CATGTCACGGGGATTAAGGCTTTATATCGCGGGATTTGATGTTGAAGCTGTGTTCACCAT 44392

******* ********************** *************** ********* ***

Human CCTATGTTCCAAAAACACCCAGACAGGGTCAATTTAACTGTTCCACCACCACTCCTGGTT 44474

Chimpanzee CCTATGTTCCAAAAACACCCAGACAGGGTCAATTTAACTGTTACACCACCACTCCTGGTT 53392

Gorilla CCTATGTTCCAAAAACACCCAGACAGGGTCAATTTAACTGTTCCACCACCACTCCTGGTT 44452

****************************************** *****************

Human GCTAAACCTCAGCTGCAATGACAAGACCTCCAGCTCAGCCTGATATCCATTCTGGGTGGG 44534

Chimpanzee GCTAAACCTCAGCTGCAATGACAAGACCTCCAGCTCAGCCTGATATCCATTCTGGGTGGG 53452

Gorilla GCTAAACCTCAGCTGCAATGACAACACCTCCAGCTCAGCCTGATATCCATTCTGGGTGGG 44512

************************ ***********************************

Human GTACATCAGCAAATAAAGGACTGCTAAATTAGTCTCAAAGTGTGGCATTTCTCTATAACT 44594

Chimpanzee GTACATCAGCAAATAAAGGACTGCTAAATTAGTCTCAAAGTGTGGCATTTCTCTATAACT 53512

Gorilla GTACATCAGCAAATAAAGGACTGCTAAATTAGTCTCAAAGTGTGGCATTTCTCTATAACT 44572

************************************************************

Human AGCTCAGTTACAACAACAGGACCAGTTTAAAAGGGGTCAATGAAATAAGCCAGGTATGTT 44654

Chimpanzee AGCTCAGTTACAACAACAGGACCAGTTTAAAAGGGGGCAATGAAATAAGCCAGGTATGTT 53572

Gorilla AGCTCAGTTACAACAACAGGACCAGTTTAAAAGGGGGCAATGAAATAAGCCAGGTATGTT 44632

************************************ ***********************

Human TAATTTCAACATCTGTTTTTTTTCTTTTCATCCACAATTATATCATCAATAAAAATATCT 44714

Chimpanzee TAATTTCAACGTCCGTTTTTTTTCTTTTCATCCACAATTATATCATCAATAAAAATATCT 53632

Gorilla TAATTTCAACGTCCGTTTTTTTTCTTTTCATCCACAATTATATCATCAATAAAAATATCT 44692

********** ** **********************************************

Human TCAGAGATTCAAAAAAAAGCCATTGATTTTTGTTATATTTCCTGAATAAATTCTGAGATC 44774

Chimpanzee TCAGAGATTCAAAAAAAAGCCATTGATTTTTGTTATATTTCCTGAACAAATTCTGAGATC 53692

Gorilla TCAGAGATTCAAAAAAAAGCCATTGATTTTTGTTATATTTCCTGAATAAATTCTGAGATC 44752

********************************************** *************

Human AGGATAGCCCTTGTTCTCCTTACTTGAATTTTGGCATATGGGCTGGGCACAGTGGCTCAC 44834

Chimpanzee AGGATAGCCCTTGTTCTCCTTACTTGAATTTTGGCATATGGGCTGGGCACAGTGGCTCAC 53752

Gorilla AGGTTAGCCCTTGTTCTCCTTACTTGAATTTTGGCATATGGGCTGCGCACAGTAGCTCAC 44812

*** ***************************************** ******* ******

Human GCCATAATCTCAGCACTTTGGGAGGCCAAGGTGGACAGACCACTTGAGGTCAGGAGTTCA 44894

Chimpanzee GCCGTAATCTCAGCACTTTGGGAGGCCAAGGTGGACAGACCACTTGAGGTCAGGAGTTCA 53812

Gorilla GCCGTAATCTCAGCACTTGGGGAGGCCAAGGTGGACAGACCACTTGAGGTCAGGAGTTCA 44872

*** ************** *****************************************

Human AGACCAGCCTTGCAAACATGGCAAACGAAGTATCTACTAAAAATACATAAAGTTGGCCAG 44954

Chimpanzee AGACCAGCCTTGCAAACATGGCAAACCAAGTATCTACTAAAAATACATAAAGTTGGCCAG 53872

Gorilla AGACCAGCCTTGCAAACATGGCAAACCAAGTATCTACTAAAAATACATAAAGTTGGACAG 44932

************************** ***************************** ***

Human GCATGGTGGTGGGCACCTGTAGTCCCAGCTACTCAGGAGGCTGAGGCAGGAAAATTGCTT 45014

Chimpanzee GCATGGTGGTGGGCACCTGTAGTCCCAGCTACTCACGACGCTGAGGCAGGAAAATTGCTT 53932

Gorilla GCATGGTGGTGGGCACCTGTAGTCCCAGCTACTCAGGANNNNNNNNNNNNNNNNNNNNNN 44992

*********************************** **

Human GAACCTGGGAGGCAGAGGTTGCAGTGAGCTGAGATCATGCTACTGAACTCTCCAGCCTGA 45074

Chimpanzee GAACCTGGGAGGCAGAGGTTGCAGTGAGCTGAGATCATGCTACTGAACT--CCAGCCTGA 53990

Gorilla NNNNNNNNNNNNNNNNNNNNNNNNNNNNNNNNNNNNNNNNNNNNNNNNNNNNNNNNNNNN 45052

Human GTGACAGACTGAAACTCTGTCACCAAAAAAAAAAAAAAAAAAAAAAAAAAAGGAAATGTT 45134

Chimpanzee GTGACAGACTGAAACTCTGTCACAAAAAAAAAAAAAAAAAAAAAAAAAAAAGGAAATGTT 54050

Gorilla NNNNNNNNNNNNNNNNNNNNNNNNNAANNNNNNNNNNNAAGAAAAAAAAAAAGAAATGTT 45112

** ** ********** ********

Human GGTGTGTGTGAAAAGAAAATTTGGATTTAGGTAAGGAGAC-------------TTTTTTT 45181

Chimpanzee GGTGTGCGTGAAAAGAAAATTTGGATTTAGGTAAGGAGAGTGTTTTTTTTTTGTTTTTTT 54110

Gorilla GGTGTGTGTGAAAAGAAAATTTGGATTTAGGTAAGGAGAC-------------NNNNNNT 45159

****** ******************************** *

Human TTTTTTTTTTCTGGATAGGATGATTGCAACAGGAGAGAGAAGATGACTATAGCAGTCAGG 45241

Chimpanzee TTTTTTTTTTCTGGATAGGATGATTGCCACAGGAGAGAGAAGAGGACTATAGCAGTCAGG 54170

Gorilla TTATNNNNNNNNNGATAGGATGATTGCAACAGGAGAGAGAAGATGACAATAGCAGTCAGG 45219

** * ************** *************** *** ************

Human TAAACTCTCCTTTTATGATATCTGCTGGCAGTTCACAGGTTAAGTGGAAAATAAGCTTTT 45301

Chimpanzee TAAACTCTCCTTTTATGATATCTGCTGGCAGTTCACAGGTTAAGTGGAAAATAAGCTTTT 54230

Gorilla TAAACTCTTCTTTTATGATATCTGCTGGCAGTTCACAGGTTAAGTGGAAAATAAGCTTTT 45279

******** ***************************************************

Human CTTTTATGGGTATATAGGATGAAAAGGTAACTGTACCAACTAGTAGGTGAGAGGATGTTT 45361

Chimpanzee CTTTTATGGGTATGTAGGATGAAAAGGTAACTGTACCAACTAGTAGCTGAGAGGATGTTT 54290

Gorilla CTTTTATGGGTATGTAGGATGAAATGGTAACGGTACCAACTAGTAGGTGAGAGGATGTTT 45339

************* ********** ****** ************** *************

Human TAACCCAGGCCACCCTTTTCTCAAAAGGGCCTCTTAACAGTAGGCAATGTGCTTGTTAAC 45421

Chimpanzee TAACCCAGGCCACCCTTTTCTCAAAAGTGCCTCTTAACAGTAGGCAATGTGCTTGTTAAC 54350

Gorilla TAACCCAGGCCACCCTTTTCTCAAAAGGGCCTCTTAACAGTAGGCAATGTGCTTGTTAAC 45399

*************************** ********************************

Human AAATGTTCCTTTAGCAAGCATTTTGCTTGACTAGTGTATGGGGAAGAAACCATTCAGTTA 45481

Chimpanzee --------------------------------------------------CATTCAGTTA 54360

Gorilla AAATGTTCCTTTAGCAAGCATTTTGCTTGACTAATGTATGGGGAAGAAACCATTCAGTTA 45459

**********

Human GTCATTGAAAAGTCAATGAATTCCCAACAAATGGATCCACTAGCATGTAGATCCCCATCA 45541

Chimpanzee GTCATTGAAAAGTCAATGAATTCCCACCAAATGGATCCACTAGCATGTAAACCCCCATCA 54420

Gorilla GTCATTGAAAAGTCAATGAATTCCCACCAAATGGTTCCACTAGCATGTAGANNNNNNTCA 45519

************************** ******* ************** * ***

Human GATATGGGGGAACTAAGGACTTAACTCTTGCCAGTATTCTTTGTTTTACATTTCTTCCTG 45601

Chimpanzee GATATGGGAGAACTAAGGACTTAACTCTTGCCAGTATTCTTTGTTTTAAATTTCTTCCTG 54480

Gorilla GATATGGGGGAACTAAGGACATAACTCTTGCCAGTATTCTTTGTTTTAAATTTCTTCCTG 45579

******** *********** *************************** ***********

Human ATGAGATTGGAGGAAGTCACACCCACAGACCAGAGTTAACGTTCTTTTCTGCTGAAGGCA 45661

Chimpanzee ATGAGATTGGAGGAAGTCACACCCACAGACCAGAGTTAACATTCTTTTCTGCTGAAGGCA 54540

Gorilla ATGAGATTGGAGGAAGTCACACCCACAGACCAGAGTTAACATTCTTTTCTGCTGAAGGCA 45639

**************************************** *******************

Human ATTTTTTAGATAAAACTTTCCCTCCTTAACCAATCAGAAATCAGAAAATATTTCAATCCA 45721

Chimpanzee AGTTTTTAGATAAAACTTTCCCTCCTTAACCAATCAGAAATCAGAAAATATTTCAATCCA 54600

Gorilla ATTTTTTAGATAAAACTTTCCCTCCTTAACCAATCAGAAATCAGAAAATATTTCAATCCA 45699

* **********************************************************

Human GCTGTCATTTTTGGGTGGCCCACTTCAAAATATCCTGCCTTTTTAATTCAAACTGTGGGA 45781

Chimpanzee GCTGTCATTTTTGGGTGGCCCACTTCAAAATATCCTGCCTTTTTAATTCAAACTGTGGGA 54660

Gorilla GCTGTCATTTTTGGGTGGCCCACTTCAAAATATCCTGCCTTTTTAATTCAAACTATGGGA 45759

****************************************************** *****

Human TATGAGGAGGTTTCTCTTTAAATAGCCTGATCAATCCTTTATTGTTTAATTTATAGCATC 45841

Chimpanzee TATGAGGAGGTTTCTCTTTAAATAGCCTGATCAATCCTTTATTGTTTAATTTATAGCATC 54720

Gorilla TATCAGGAGGTTTCTCTTTAAATAGCCTGATCAATCCTTTATTGTTTAATTTATAGCATT 45819

*** *******************************************************

Human CCCCCTACATCCCTTTTTCCTTTTTCTCTCTTTTTTCTTCCTTTCTCCCTTTTTTATATG 45901

Chimpanzee CCCCCTATATCCCTTTTTCCTTTTTCTCTCTTTTTTCTTCCTTTCTCCCTTTTTTACATG 54780

Gorilla CCCCCTACATCCCTTTTTCCTTTTTCTCTCTTTTTTCTTCCTTTCTCCCTTTTTTACATG 45879

******* ************************************************ ***

Human CCCAGACATGCCACAGTACCAGGCGTTGCCAGCTCACATTCCTTTCACTATTTAGAAACA 45961

Chimpanzee CCCAGACATGCCGCAGTACCAGGCGTTGCCAGCTCACATTCCTTTCACTATTTAGAAACA 54840

Gorilla CCCAGACATGCCACAGTACCAGGCGTTGCCAGCTCACATTCCTTTCACTATTTAGAAACA 45939

************ ***********************************************

Human AGACTAGCTGTCTAGCTCATTACAGACACCGTTTTTTTCCCCTCTCTCCTTTATGTGCCC 46021

Chimpanzee AGACTAGCTGTCTAGCTCATTACAGACACTGTTTTTTCCCCCTCTCTCCTTTATGTGCCC 54900

Gorilla AGACTAGCTGTCTAGCNNATCACAGACACTGTTTNTTNNNNNNNTCTCCTTTATGTGCCC 45999

**************** ** ******** **** ** ****************

Human AACTTATTTAAAAA-AAAAAAAAAAGTTCAGATGTTTAGCCAACCAGGATTAGTTTAGAG 46080

Chimpanzee AACTTATTTAAAAAGAAAAAAAAAAGTTCAGATGTTTAGCCAACCAGGATTAGTTTAGAG 54960

Gorilla AACTTNTTTTAAAA-ANNNNNAAAAGTTCAGATGTTTAGCCAACCAGGATTAGTTTAGAG 46058

***** *** **** * ***************************************

Human AGTATGACCCGACCCTGGCCAATGGGGAAAGGGTACATATGGGGCAGGACTTGTCACACA 46140

Chimpanzee AGTATGACCCGACCCTGGCCAATGGGGAAAGGGTACATACGGGGCAGGACTTGTCACACA 55020

Gorilla AGTATGACCCGACCCTGGCCAATGGGGAAAGGGTACATACGGGGCAGGACTTGTCACACA 46118

*************************************** ********************

Human CATCTGTGTGAAAAGAACAATTAAACAGGCTTTGCGTGAGCAATAAAGCTGTTTTTTTCC 46200

Chimpanzee CATCTGTGTGAAAAGAACAATTAAACAGGCTTTGCGTGAGCAATAAAGCTGTTTTTTTTC 55080

Gorilla CNNNNNNNNNNNNNNNNNNNNNNANNNNNNNNNNNNNNNNNNNNNNNNNNNNNNNNNNNN 46178

* *

Human ACCTGGGTGCAGGCAGGCTGAGTCTGAAAAGGAGTTGGCAAAGAGAGTTATGGGTGGGGC 46260

Chimpanzee ACCTGGGTGCAGGCAGGCTGAGTCTGAAAGGGAGTCGGCAAAGAGAGTTATGCGTGGGGC 55140

Gorilla ACCTGGGTGCAGGCAGGCTGAGTCTGAAAAGGAGTAGGCAAAGAGAGTTATGGGTGGGGC 46238

***************************** ***** **************** *******

Human AGTTTTATAGGATTTGGGTAGGTAGTGCAAAATTATAGTCAAAGGGTGTTTGCTCTCTTG 46320

Chimpanzee AGTTTTATAGGATTTGGGTAGGTAGTGCAAAATTATAGTCAAAGGGTGTTTGCTCTCTTG 55200

Gorilla AGTTTTATAGGATTTGGGTAGGTATTGCAAAGTTATAGTCAAAGGGTGTTTGCTCTCTTG 46298

************************ ****** ****************************

Human CAGGAAGGGGTGGGGGTCACAAGGTGCACAGTGGGGGAGCTCCTGAGCCAGGAGAAGGAA 46380

Chimpanzee CAGGAAGGGGTGGGGGTCACAAGGTGCACAGTGGGGGAGCTCCTGAGCCAGGAGAAGGAA 55260

Gorilla CAGGAAGGGGTGGGGGTCACAAGGTGCACAGTGGGGGAGCTCCTGAGCCAGGAGAAGGAA 46358

************************************************************

Human TTTCACAAGATAATGTTATCAGTTAAACAGGAACGGGCCATTTTCACTTCTTTTGTAATT 46440

Chimpanzee TTTCACAAGATAATGTTATCAGTTAAACAGGAATGGGCCATTTTCACTTCTTTTGTAATT 55320

Gorilla TTTCACAAGATAAGGTTATCAGTTAAACAGGAACGGGCCATTTTCACTTCTTTTGTAATT 46418

************* ******************* **************************

Human CTTCAGTTGCTTCAGGTCATCTGGATGTATACGTGTAGGCTTAGACTCAGAGGCCTGACA 46500

Chimpanzee CTTCAGTTGCTTCAGGTCATCTGGATGTATACGTGTAGGCTTAGACTCAGAGGCCTCACA 55380

Gorilla CTTCAGTTGCTTCAGGTCATCTGGATGTATACGTGTAGGCTTAGACTCAGAGGCCTGACA 46478

******************************************************** ***

Human TGACTTGCATCAGGAATGATGTCTCTTGTGCCCCTTTATTCAGGTGTGCTCTCATGGCTA 46560

Chimpanzee TGACTTGCACCAGGAATGATGTCTCTTGTGCCCCTTTATTCAGGTGTGCTCTCATGGCTA 55440

Gorilla TGACTTGCATCAGGAATGATGTCTCTTGTGCCCCTTTATTCAGGTGTGCTCTATTGGCTA 46538

********* ****************************************** ******

Human CTGGCCAAGGAGAAGCACCCCTCTGTGCAGAAATAAAATGGCTTTGCTAAGAATCCTTTG 46620

Chimpanzee CTGGCCAAGGAGAAGCACCCCTCTGTGCAGAAATAAAATGGCTTTGCTAAGAATCCTTTG 55500

Gorilla CTGGCCAAGGAGAAGCACCCCTCTGTGCAGAAATAAAATGGCTTTGCTAAGAATCCNNNN 46598

********************************************************

Human TTTGAGTGTCCAATTTCCTTAGGATTTTGAATATTATTCCCATTAAAACCAATGTATGAT 46680

Chimpanzee TTTGAGTGTCCAATTTCCTCAGGATTTTGAATATTATTCCCATTAAAACCAATGTGTGAT 55560

Gorilla NNNGAGTGTCCAGTTTCCTTAGGATTTTGAATATTATTCCCATTAAAACCAACGTATGAT 46658

********* ****** ******************************** ** ****

Human CTCCACGTATTCATTTACAATGTTACCTGTGACTTCTGCATTCCTGAAATGTACTACTGC 46740

Chimpanzee CTCCACGTATTCATTTACAATGTTACCTGTGGCTTCTCCATTCCTGAAATGTACTACTGC 55620

Gorilla CTCCATGTATTCATTTACAATGTTACCTGTGACTTCTGCATTCCTGAAATGTACTACTGC 46718

***** ************************* ***** **********************

Human CTTTAAAAACCATTACTTGCAAGCCACCGGGAGGTTAGGTCTTCAGTGAGAACTGCCCAA 46800

Chimpanzee CTTTAAAAACCATTACTTGCAAGCCACTGGGAGGTTAGGTCTTCAGTGAGAACTGCCCAA 55680

Gorilla CTTTAAAAACCATTACTTGCAAGCCACCGGGAGGTTAGGTCTTAAGTGAGAACTGCCCAA 46778

*************************** *************** ****************

Human TTCTCTTTGCTTGGTGTCCTGCAAATATAAACACCCTCCTTTCCGCTGCTTCAAAACCTC 46860

Chimpanzee TTCTCTTTGCTTGGTGTCCTGCAAATATAAACACGCTCCTTTCCGCTGCTTCAAAACCTC 55740

Gorilla TTCTCTTTGCTTGGTGTCCTGCAAATATAAACACCCTCCTTTCTGCTGCTTCAAAACCTC 46838

********************************** ******** ****************

Human AGTGTGAATGTTTGGCCTTACTGTGCCAGGCAAGGAACCCCTAGATGGGTCCAGTAACAC 46920

Chimpanzee AGTGTGAATGTTGGCCCTTACTGTGCCAGGCAAGGAACCCCTAGATGGGTCCAGTAACAC 55800

Gorilla AGTGTGAATGTTTGGCCTTACTGTGCCAGGCAAGGAACCCCTAGATGGGTCCAGTAACAC 46898

************ * *********************************************

Human CACCAGCATTTTGTTGGAATCATAAAGACTGAGTAATCTTCAAGGTCAAGACTGCAGAGC 46980

Chimpanzee CTCCAGCATTTTGTTGGAATCATAAAGACTGAGTAATCTTCAAGGTCAAGACTGCAGAGC 55860

Gorilla CACCAGCATTTTGTTGGAATCATAAAGACTGAGTAATCTTCAAGATCAAGACTGCAGAGC 46958

* ****************************************** ***************

Human CTCAGAGCTTGTCTGCTGCCGATTACAATTAAACCCTACTCACAATCCCTCCTTTCCCGT 47040

Chimpanzee CTCAGAGCTTGTCTGCTGCCGATTACAATTAAACCCTACTCACAATCCCTCCTTTCCCGT 55920

Gorilla CTCAGAGCTTGTCTGCTGCCGATTATAATTAAACCCTACTCACAATCCCTCCTTTCCCGT 47018

************************* **********************************

Human GCTTAAATCATGGAATGAGAGACATTTGAGACGCCAGGACAAAAATCTTGGAAATGCCAA 47100

Chimpanzee GCTTAAATCATGGAATGAGAGACATGTGAGACACCAGGACAAAAATCTTGGAAATGCCAA 55980

Gorilla GCTTAAATCATGGAATGAGAGACATTTGAGACACCAGGACAAAAATCTTGGAAATGCCAA 47078

************************* ****** ***************************

Human ATACCTGTAGTTATCTACTTCTCATATGCTCTTTCCAAAACAGAAAGTGAAAAATTTTAA 47160

Chimpanzee ATACCTGTAGTTATCTACTTCTCATATGCTCTTTCCGAAACAGAAAGTGAAAAATTTTAA 56040

Gorilla ATACATGTAGTTATCTACTTCTCATATGCTCTTTCCAAAACAGAAGGTGAAAAATTTTAA 47138

**** ******************************* ******** **************

Human AGTGGTTGATATTCCCAGTAGATTCCAAGTTTATCATATCTGTCTGAAAATGTTAATCTC 47220

Chimpanzee AGTGGTTGATATTCCCAATAGATTCCAAGTTTATCATATCTGTCTGAAAATGTTAATCTG 56100

Gorilla AGTGGTTGATATTCCCAATAGATTCCAAGTTTATCATATCTGTCTGAAAATGTTAATCTG 47198

***************** *****************************************

Human TGATTACCCTATCAAATGTGCCCATAAGTCTGCAGTATTTTATTTATTTATTTTTTAGGG 47280

Chimpanzee TGATTACCCTATCAAATGTGCCCATAAGTCTGCAGTATTTTATTTATTTATTTTTTAGGG 56160

Gorilla TGATTACCCTATCAAATGTGCCCATAAGTCTGCAGTATTTTATTTATTTATTTTTTAGGG 47258

************************************************************

Human CACTTTCCTTCATAATTCTTCTATTGTGTGAATAATTCTCCAAAAATAAAATGAAAGATG 47340

Chimpanzee CACTTTCCTTCATAATTCTTCTATTGTGTGAATAATTCTCCAAAAATAAAATAAAAGATG 56220

Gorilla CACTTTCCTTCATAATTCTTCTATTGTGTGAATAATTCTCCAAAAATAAAATAGAAGATG 47318

**************************************************** ******

Human GAAAAATAAAACCTCAGTAAAATACAGGAAAGTAATTGTTTCTGTGATTCTCAATACATA 47400

Chimpanzee GAAAAATAAA-CCTCAGTAAAATACAGGAAAGTAATTGTTTCTGTGATTCTCAATACATA 56279

Gorilla GAAAAATAAA-CCTCAGTAAAACACAGGAAAGTAATTGTTTCTGTGATTCTCAATGCATA 47377

********** *********** ******************************** ****

Human TTAAACAGAAAAAAAAAAAGCAGTTGAGTTTTGAAGAAAACAGATGTTGAAGATATGTGA 47460

Chimpanzee TTAAACAGAAAAAAAAA--GCAGTTGAGTTTTGAAGAAAACAGATGTTGAAAATATGTGA 56337

Gorilla TTAAACAGNAAAAAAAAANGCAGTTGAGTTTTGAAGAAAACAGATGTTGAAAATATGTGA 47437

******** ******** ******************************** ********

Human CTTCATTAAGCCAGAGATCAAATTAATTGCTATGAAGGCAGTCCACTGAGGTAGCCAGGA 47520

Chimpanzee CTTCATTAAGCCAGAGATCAAATTAATTGCTATGAAGGCAGTCCACTGAGGTAGCCAGCA 56397

Gorilla CTTCATTAAGCCAGAGATCAAATTAATTGCTATGAAGGCAGTCCACTGAGGTAGCCAGGA 47497

********************************************************** *

Human AATTAGTGAAAGCAGGTTTTCAGTATTGAAAAAAAGGAAATGAAAATTATGACTTAGGCA 47580

Chimpanzee AATTAGTGAAAGCAGGTTTTCAGTATTGAAAAAAAGGAAATGAAAATTATGACTTAGGCA 56457

Gorilla AATTAATGAAAGCAGGTTTTCAGTATTGAAAAAAAGAAAATGAAAATTATGAGTTAGGCA 47557

***** ****************************** *************** *******

Human CAATTCTGTAAGTACGGTATTTTAAAAATACATTATAAAATTTACAGTGTATATTTTCAG 47640

Chimpanzee CAATTCTGTAAGTACGGTATTTTAAAAATACATTATAAAATTTACAGTGTATATTTTCAG 56517

Gorilla CAATTCTGTAAGTACGGTATTTTAAAAATACATTATAAAATTTACAGTGTATATTTTCAG 47617

************************************************************

Human GTTTCATCAATGCATGTGTCAAGATGCTGGCATAATTTTATTGATCCTAAACATCAAACT 47700

Chimpanzee GTTTCATCAATGCATGTGTCAAGATGCTGGCATAATTTTATTGATCCTAAACATCAAACT 56577

Gorilla GTTTCATCAATGCATGTGTCAAGATGCTGGCATAATTTTATTGATCCTAAACATCAAACT 47677

************************************************************

Human ACTTTGGAAAAATATCAAAACCTGTAGAAAAATACATTAATTTAGAAAAATGAATTAGAA 47760

Chimpanzee ACTTTGGAAAAATATCAAAACCTGTAGAAAAATACATTAATTTAGAAAAATGAATTAGAA 56637

Gorilla ACTTTGGAAAAATATCAAAACCTGTAGAAAAATACATTAATTTAGAAAAATGAATTAGAA 47737

************************************************************

Human AAATGAATAAATTTTCCATCATGTCTCAGGGTTACAATAATTTTATCTTCAAAGAAAAGC 47820

Chimpanzee AAATGAATAAATTTTCCATCATGTCTCAGGGTTACAAAAATTTTATCTTCAAAGAAAAGC 56697

Gorilla AACTGAATAAATTTTCCATCATGTCTCAGGGTTACAATAATTTTATCTTCAAAGAAAAGC 47797

** ********************************** **********************

Human TATCATTACACCCTGAAATAATGGATGGATACCCAAGGCATATGATAGTTTGCCTCGTAT 47880

Chimpanzee AATCATTACACCCTGAAATAACGGATGGATAACCAAGGCATATGATAGTTTGCCTCGTAT 56757

Gorilla AATCATCACACCCTGAAATAATGGATGGATACCCAAGGCATATGATAGTTTGCCTCGTAT 47857

***** ************** ********* ****************************

Human GTCTTTCAGAAAATGAATAAAATTACTGAAAATTGTCCGGTTATATACAACTTAGAGTTA 47940

Chimpanzee TTCTTTCAGAAAATGAACAAAATTACTGAAAATTGTCCTGTTATATACAACTTAGAGTTA 56817

Gorilla TTCTTTCAGAAAATGAATAAAATTACTGAAAATTGTCCTGTTATATACAACTTAGAGTTA 47917

**************** ******************** *********************

Human TGTTGAAAAAGACAATAGTTTCTCCTTCATAGACTCAGTAGCAGAGGGATGTCATAGAAT 48000

Chimpanzee TGTTGAAAAAGACAATACTTTCTCCTTCATAGACTCAGTAGCAGAGGGATGTCATAGAAT 56877

Gorilla TGTTGAGAAAGACAATACTTTCTCCTTCATAGACTCAGTAGCAGAGGGATGTCATAGAAT 47977

****** ********** ******************************************

Human TGTCAATAAAGTCATAAGGGTTCCCTTCTATAAGTGTAATGCCAAAGGGTCTTGTCTTAG 48060

Chimpanzee TGTCAATAAAGTCATAAGGGTTCCCTTCTATAAGTGTAATGCCAAAGGGTCTTGTCTTAG 56937

Gorilla TGTCGATAAAGTCATAAGGGTTCCCTTCTATAAGTGTAATGCCAAAGGGTCTTGTCTTAG 48037

**** *******************************************************

Human CCATGCCAAAGGATTGGTGTGGCAGCAGCCTGTGGTGAGAGAGAGATACGGATAGGACTG 48120

Chimpanzee CCATGCCAAAGTGTTGGTGTGGCGGCAGCCTGTGGTGAGAGAGAGATACAGATAGGACTG 56997

Gorilla CCATGCCAAAGGATTGGTGTGGCGGCAGCCTGTGGTGAGAGAGAGATACGGATAGGACTG 48097

*********** ********** ************************* **********

Human AGAGAAAGAGGTTGTAGGCTTTATTAAGCAGAGTAACAGTACAAAGCTTCCACAGCATGG 48180

Chimpanzee AGAGAAAGAGGTTGTAGGCTTTATTAAGCAGAGTAACAGTACTAAGCTTCCACAGCATGG 57057

Gorilla AGAGAAAGAGGTTGTAGGCTTTATTAAGCAGAGTAACAGTACTAAGCTTCCACAGCATGG 48157

****************************************** *****************

Human AAGGGCTCCCAAGCGGGTAGCCAGTGTTAGATTTTTCAATCACCTTTTAAACTCTTCAAG 48240

Chimpanzee AAGGGCTCCCAAGCGGGTAGCCAGTGTTAGATTTTTCAATCACCTTTTAAACTCTTTAAG 57117

Gorilla AAGGGCTCCCAAGCGGGTAGCCAATGTTAGATTTTTCAATCACCTTTTAAACTCTTTAAG 48217

*********************** ******************************** ***

Human GCGCGAAATACGTGTGGACGGAAGATGTTACCAGAGCGAGAAACAAAGACAATTAACGTG 48300

Chimpanzee GCGGGAAATACGTGTGGACGGAAGATGTTACCAGAGCGAGAAACAAAGACAATTAACGTG 57177

Gorilla GCGGGAAATACATGTGGACGGAAGATGTTACCAGAGCGAGAAACAAAGACAATTAACGTG 48277

*** ******* ************************************************

Human TCTCAGATCTTGAAGAAAACCAGAACTGCCACTTGTTTTGTCTACTTTATGACCTTGCAG 48360

Chimpanzee TCTCAGATCTTGAAGAAAACCAGAACTGCCACTTGTTTTATCTACTTTATGACCCTGCAG 57237

Gorilla TCTCAGATCNNNNNNNAAANNNNNNNNNNNNNNNNNNNNNNNNNNNTGATGACCTTGCAG 48337

********* *** * ****** *****

Human AGTCAAGGCAAAGGAGTCAGTATCTCACAGGATTTTACAAATTGTGTTTCCAAATAATTG 48420

Chimpanzee AGTCAAGGCAAAGGAGTCAGTATCTCACAGGATTTTACAAATTGTGTTTCCAAATAATTG 57297

Gorilla AGTCAAGGCAANNNAGTCAGTACCTCACAGGATTTTACAAATTGTGTTTCCAAATAATTG 48397

*********** ******** *************************************

Human GAATTGGGAGAATAGATAACCTCTGCTTGTCACAGAAAAACAGGCTTTTAATATTCCTTT 48480

Chimpanzee GAATTGGGAGAATAGATAACCTCTGCTTGTCACAGAAAAACAGGCTTTTAATATTCCTTT 57357

Gorilla GAATTGGGAGAATAGATAACGTCTGCTTGTCACAGAAAAACAGGCTTTTAATATTCCTTT 48457

******************** ***************************************

Human TAGTTTCAGGGGATGGGGAAGGGAGAGAGGACACAGGGAAGCTTACAACAACATTTTCAC 48540

Chimpanzee TAGTTTCAGGGGATGGGGAAGGGAGAGAGGACACAGGGAAGCTTACAACAACATTTTCAC 57417

Gorilla TAGTTTCAGGGGATGGGGAAGGGAGAGAGGACACAGGGAAGCTTACAACAAAATTTTCAC 48517

*************************************************** ********

Human TGTTTCTAGCTTTCTTGGGGAAGAAAACACATGTACAAATTCTGATGTTAGGGATATTTT 48600

Chimpanzee TGTTTCTAGCTTCCTTGGGGAAGAAAACACATGTACAAATTCTGATGTTAGGAATATTTT 57477

Gorilla TNNNNNNAGCNTTCTTGGGGAAGAAAACACATGTACAAATTCTGATGTTAGGAATATTTT 48577

* *** * *************************************** *******

Human AAACATATATCTTCAATATTATTCATCCAGGACCAAAGTATGTCCTGATGCAGGAAATAA 48660

Chimpanzee AAACATATATCTTCAATATTATTCATCCAAGACCAAAGTATGTCCTGATGCAGGAAATAA 57537

Gorilla AAACATATGTCTTCAATATTATTCATCCAGGACCAAAGTATGTCCTGATGCAGGAAATAA 48637

******** ******************** ******************************

Human GTGAGTTTCACAGCTTTCTGAGCCCCTACTCTACCCAGGAAGACCAGATGGCACCTCCTC 48720

Chimpanzee GTGAGTTTCACAGCTTTCTGAGCCCCTACTCTACCCAGGAAGCCCAGATGGCACCTCCTC 57597

Gorilla GTGAGTTTCACAGCTTTCTGAGCCCCTACTCTACCCAGGAAGCCCAGATGGCACCTCCTC 48697

****************************************** *****************

Human TCATAAGCATCAACTCCCCTTGAAAGTGGCACAGTTACATATACAAACAAGAATAATATT 48780

Chimpanzee TCATAAGCATCAACTCCCCTTGAAAGTGGCACAGTTACATATACAAACAAGAATAATATT 57657

Gorilla TCATAAGCATCAACTCCCCTTGAAAGTGGCACAGTTAAATATACAAACAAGAATAATATT 48757

************************************* **********************

Human AATGCCAATTAATAGAGATATTGAATGGAATTTTGCAAATAAAATACATAACCTAAATTG 48840

Chimpanzee AATGCCAATTAATAGAGATATTGAATGGAATTTTGCAAATAAAATACGTAACCTAAATTG 57717

Gorilla AATGCCAATTAATAGAGATATTGAATGGAATTTTGCAAATAAAATACATAACCTAAATTG 48817

*********************************************** ************

Human ACAGCTTCCATAGGCCCTGGGGAATGACTTCAAAATACACTGCCTACTTGCTTTTCTCTT 48900

Chimpanzee ACAGCTTCCATAGGCCCTGGGGAATGACTTCAAAATACACTGCCTACTTGCTTTTCTCTT 57777

Gorilla ACAGCTTCCATAGGCCCTGGGGAATGACTTCAAAATACACTGCCTACTTGCTTTTCTCTT 48877

************************************************************

Human TTCTTTTCTGTTAACAGACTAAGTTGCAGTAAACACTCAGCCTTCTCTGTCTCACAGAAC 48960

Chimpanzee TTCTTTTCTGTTAGCAGACTAACTGGCAGTAAACACTCAGCCTTCTCTGTCTCACAGAAC 57837

Gorilla TTCTTTTCTGTTAACAGACTAAGTTGCAGTAAACACTCAGCTTTCTCTGTCTCACAGAGC 48937

************* ******** * **************** **************** *

Human CACACCATCTAGTCCCATTCTGGAAAATGGGGGTAGAATACTGGGAAGAGGGTACCTTGG 49020

Chimpanzee CACACCATCTAGTCCCATTCTGGAAAATGTGGGTAGAATACTGGGAAGAGGGTATCTTGG 57897

Gorilla CACACCATCTAGTCCCATTCTGGAAAATGTGGGTAGAATACTGGGAAGAGGGTACCTTGG 48997

***************************** ************************ *****

Human AAATATGCCTCGGGTTTTTACTCTTCTCCTTCTCTTTCTCCTTGAAATTTACAATCTGTG 49080

Chimpanzee AAATATGCCTCAGGTTTTTACTCTTCTCCTTCTCTTTCTCCTTGAAATTTACAATCTGTG 57957

Gorilla AAATATGCCTCGGGTTTTTACTCTTCTCCTTCTCTTTCTCCTTGAAATTTACAATCTGTG 49057

*********** ************************************************

Human GACAGTCAGTACCACTCAGATGTTTGTGTATGTTTCTGTAGATGGATACGCCAGATAGAT 49140

Chimpanzee GACAGTCAGTACTACTCAGATGTTTGTGTATGTTTCTGTAGATGGATACGCCAGATAGAT 58017

Gorilla GACAGTCAGTACTACTCAGATGTTTGTGTATGTTTCTGTAGATGGATATGCCAGATAGAT 49117

************ *********************************** ***********

Human AAGATTGATAGAAGATAGACAGGTGAGATTGATAGATGATGGATAGATGGATAATAGAAG 49200

Chimpanzee AAGATTGATAGATGATAGATAGGTGAGATTGATAGATGATGGATAGATGGATAATAGAAG 58077

Gorilla AAGATTGATAGATGATAGATAGGTGAGATTGATAGATGATGGATAGATGGATAATAGAAG 49177

************ ****** ****************************************

Human ATATATTTAAATAATAGATATAAAATATGGATAATTGATAATCGATGTATATAAGTTGGA 49260

Chimpanzee ATATATTTAAATAATAGATATAAAATATAGATAATTGATAATCGATGTATATAAGTTGGA 58137

Gorilla ATATATTTAAATAATAGATATAAAATATAGATAATTGATAATCGATGTATATAAGCTGGA 49237

**************************** ************************** ****

Human TGATAGATGGGTACCTAAATGGGAGAGATAGATGTTGGATGGATGGATAGACAGTGGAAA 49320

Chimpanzee TGATAGATGGGTACATAAATGGGAGAGACAGATGTTGGATGGATGGATAGACAGTGGAAA 58197

Gorilla TGATAGATGGGTACATAAATGAGAGAGATAGATGTTGGATGGATGGATAGACAGTGGAAA 49297

************** ****** ****** *******************************

Human TGCTATTTCAATAATAGATATGACACGTAGATATTTGAAAAAAGTAATAGATATAGATGA 49380

Chimpanzee TGCTATTTCAATAATAGATATGACATATAGATATTTGAAGAAAGTAATAGATATACATGA 58257

Gorilla TGCTATTTCAATAATAGATATGACATATAGATATTTGAAGAAAGTAATAGATATAGATGA 49357

************************* ************ *************** ****

Human TGAATGCATACATGAAGAAATGATAGAAAGTATGTTTAGATGCCAGCGATGAATATAGCT 49440

Chimpanzee TGAATGGATACATGAAGAAATGATAGAAAATATGTTTAGATGCCAGCGATGAATATAGCT 58317

Gorilla TGAATGGATACATGAAGAAATGATAGAAAATATGTTTAGATGCCAGCGATGAATATAGCT 49417

****** ********************** ******************************

Human TATTGATAATAGATAATTGATAAATGGATAAATAGGATAGATAGGTAGTTTAGCTAGACA 49500

Chimpanzee TATTGATAATAGATAATTGATAAATGGATAAATAGGATAGATAGGTAGTTTAGCTAGATA 58377

Gorilla TATTGATAATAGATAATTGATAAATGGATAAATAGGATAGATAGGTAGTTTAGCTAGATA 49477

********************************************************** *

Human ATATAAAAGACACATAAATAATATATATATAATTTAGGATATTAATAAATATTTAGTAAC 49560

Chimpanzee ATATAAAAGACACATAAATAATATATATATAATTTAGGATATTAATAAATATTTAGTAAC 58437

Gorilla ATATAAAACACAC----ATAATATATATATAATTTAGGATATTAATAAATATTTAGTAAC 49533

******** **** *******************************************

Human AAGTGATGCTGGATTGAGGGACAGAGAGGTAACAGAAAAGATACTTAAGTTTTATATAAA 49620

Chimpanzee AAGTGATGCTGGATTGAGGGACAGAGAGGTAACAGAAAAGATACTTAAGTTTTATATAAA 58497

Gorilla AAGTGATGCTGGATTGAGGGACAGAGAGATAACAGAAAAGATACTTAAGTTTTATATAAA 49593

**************************** *******************************

Human ATATTGATTATTGATGATAGAGAATATTTGATGAACTAGAGGATGAACGATGGATGAACA 49680

Chimpanzee ATATTGATTATTGATGATAGAGAATATTTGATGAAATAGAGGATGAACGATGGATGAACA 58557

Gorilla ATATTGATTATTGATGATAGAGAATATTTGATGAAATAGAGGATGAATGATGGATGAACA 49653

*********************************** *********** ************

Human GGAGATATGAATGAAAAGTTAGATGGATGGATAACAAGATAGGTGGTAAATATTCACTTC 49740

Chimpanzee GGAGATATGAATGAAAAGTT----GGATGGATAACAAGATAGGTG-TAAATATTCACTTC 58612

Gorilla GGAGATATGAATGAAAAGTTAGATGGATGGATAACAAGATAGGTGGTAAATATTCACTTC 49713

******************** ********************* **************

Human TATAGCTCACTCTTTTTCTATGCAAACAAACATAGACGTACACACGTACACACTATGTAT 49800

Chimpanzee TATAGCTCACTCTTTTTCTATGCAAACA----TACACATACACACGTACACACTAT---- 58664

Gorilla TATAGCTCACTCTTTTTCTATGCAAACAAACGTACACATACACACATACACACTATATAT 49773

**************************** ** ** ******* **********

Human GTGTATGTATGTGTATACCTCTCTATATGCAAATTTTTTTTCTGTTTTTTTTTTT---TT 49857

Chimpanzee ------GTATGTGTATACCCCTCTATATGCAAATTTTTTTTCTGTTTTTTTTTTTATGTT 58718

Gorilla GTGTATNNNNNNNNNNNNNNCTNNATATGCAAATTTTTTTTCTNNNNTTTTTTTT---TT 49830

** ******************* ******** **

Human TTTTTT-------AAGATGGAGTCTCACTCACTGTCTTGCCCAGGCTGGAGTGCAGTGGC 49910

Chimpanzee TTTTTTTTTA---AAGATGGAGTCTCACTCACTGTCTTGCCCAGGCTGGAGTGCAGTGGC 58775

Gorilla TCNNNN-------AAGATGCAGTCTCACTCACTGTCTTGCCCAGGCTGGAGTGCAGTGGC 49883

* ****** ****************************************

Human TTGATCTCAGCTCACTGCAGCCTCCACCTCCTGGGTTCAACTTGTTCTCCTGCCTCAGCC 49970

Chimpanzee TTGATCTCAGCTCACTGCAGCCTCCACCTCCTGGGTTCAACTTGTTCTCCTGCCTCAGCC 58835

Gorilla TTGATCTCAGCTCACTGCAGCCTCCACCTCCTGGGTTCAACTTGTTCTCCTGCCTCAGCC 49943

************************************************************

Human TCCAGAGTAGCTGGAATTACTGACAGGCACCACAATTTCCAGCAATTTTTTTTTTTTTTT 50030

Chimpanzee TCCAGAGTAGCTGGAATTACTGACAGGCACCACAATTTCCAGCAATTTTTTTTTTTTTTT 58895

Gorilla TCCAGAGTAGCTGGAATTACCAACAGGCACCACAATTTCCAGC--TTTTTTTTTTTTTTT 50001

******************** ********************* ***************

Human T---------GGTGTGTGTGTATTTTTAGTAGATATGGGGTTTCACCATGTTGGCCAGGC 50081

Chimpanzee TTTTTTTTTTGGTGTGTGTGTATTTTTAGTAGATATGGGGTTTCACCATGTTGGCCAGGT 58955

Gorilla T---------GGTGTGTGTGTATTTTTAGTAGATATGGGGTTTCACCATGTTGGCCAGGC 50052

* *************************************************

Human TGGTCTTGAACTCCTGGTTTCAAGTGATCTGCCCGCCTTGGCCTCCCAAATTGCTGAAAT 50141

Chimpanzee TGGTCTTGAACTCCTGGTTTCAAGTGATCTGCCTGCCTTGGCCTCCCAAATTGCTGAAAT 59015

Gorilla TGGTCTTGAACTCCTGGTTTCAAGTGATCTGCCCGCCTTGGCCTCCCAAATTGCTGAAAT 50112

********************************* **************************

Human TAGAGGTATGAGCCACTGTGCCTGGCCAATATTTTTCTTGTTATGTTTTTGGTAAAATAC 50201

Chimpanzee TAGAGGTATGAGCCACTGTGCCTGGCCAATATTTTTCTTGTTATGTTTTTGGTAAAATAC 59075

Gorilla TAGAGGTATGAGCCACTGTGCCTGGCCAATATTTTTCTTGTTATGTTTTTGGTAAAACAC 50172

********************************************************* **

Human ACTTGGGCCAAAAATTGTATGCAATTACAATATTTTCAGATTCCATTTCATTACTTGCTG 50261

Chimpanzee ACTTGGGTCAAACATTGTATGCAATTACGATATTTTCAGATTCCATTTCATTACTTGCTG 59135

Gorilla ACTTGGGTCAAAAATTGTATGCAATTACAATATTTTCAGATTCCATTTCACTACTTGCTG 50232

******* **** *************** ********************* *********

Human TAACATTCTAGCATTGAATTTCCACCTATTTATTTAAAGATGTTTTCCCTTGCATCACTG 50321

Chimpanzee TAACATTCTAGCATTGAATTTCCACCTATTTATTTAAAGATGTTTTCCCTTGCATCACTG 59195

Gorilla TAACATTCCAGCATTGAATTTCCACCTATTTATTTAAAGATGTTTTCCCTTGCATCACTG 50292

******** ***************************************************

Human CTCTCTTTAAACCTTTTGAACCTTGCAAGACTGAGTGCAGTAGTAAAGTGGCCTGTTCTC 50381

Chimpanzee CTCTCTTTAAACCTTTTGAACCTTGCAAGACTGAGTGCAGTAGTAAAGTGGCCTGTTCTC 59255

Gorilla CTCTCTTTAAACCTTTTGAACCTTGCAAGACTGAGTGCAGTAGTAAAGTGGCCTGTTCTC 50352

************************************************************

Human AGTGTTTGGGCAGGTGTTTGAATGGATCTGCCTGATGCACAATGAAGAGGCACTGCATCT 50441

Chimpanzee AGTGTTTTGGCAGGTGTTTGAATGGATCTGCCTGAGGCACAATGAAGAGGCACTGCCTCT 59315

Gorilla AGTGTTTTGGCTGGTGTTTGAATGGATCTGCCTGATGCACAATGAAGAGGCATTGCATCT 50412

******* *** *********************** **************** *** ***

Human CACATGGTTTCCAAGAAGGCAGTGACTACCTGAGTCACTA-TAAACAGGAAGGGAAAAAA 50500

Chimpanzee CACATGGTTTCCAAGAAGGCAGTGACTACCTGAGTCACTAATAAACAGGAAGGGAAAAAA 59375

Gorilla CACATGGTTTCCAAGAAGGCAGTGACTACCTGAGTCACTA-TAAACAGGAAGGGAAAAAA 50471

**************************************** *******************

Human TACGTTTGTCCTGACAGATTCCTAGAGAATTCAGCATATCATTGCTCAGATTCAATTAAA 50560

Chimpanzee TACGTTTGTCCTGACAGATTCCTAGAGAATTCAGCATATCATTGCTCAGATTCAATTAAA 59435

Gorilla TACGTTTGTCCTGACAGATTCCTAGAGAATTCAGCGTATCATTGCTCAGATTCAATTAAA 50531

*********************************** ************************

Human GATAAAAATCTTTTTCCTCTTACAGGAACACACAGTCTCAGAATTTATATATATTTTTCC 50620

Chimpanzee GATAAAAATCTTTTTCCTCTTACAGGAACACACAGTCTCAGAATTTATATATATTTTTCC 59495

Gorilla GACAAAAATCTTTTTTCTCTTACAGGAACACACAGTCTCAGAATTTATATATATTTTTCC 50591

** ************ ********************************************

Human TATTTGCATTTATTGCTCATTTAGATTTATAATGTTTCTGTTTTGCTTTGTACTTGCCTC 50680

Chimpanzee TATTTGCATTTATTGCTCATTTAGATTTATAATGTTTCTGTTTTGCTTTGTATTTGCCTC 59555

Gorilla TATTTGCATTTATTGCTCATTTAGATTTATAATGTTTCTGTTTTGTTTTGTATTTGCCTC 50651

********************************************* ****** *******

Human TCTTGGTTTTAAGTTTTACTGTGAGAGTAACCCTTCTCATTAACTCATCTATTTGCCCAT 50740

Chimpanzee TCTTGGTTTTAAGTTTTACCGTGAGAGTAACCCTTCTCATTAACTCATCTATTTGCCCAT 59615

Gorilla TCTTGGTTTTAAGTTTTACTGTGCGAGTAACCCTTCTCATTAACTCATCTATTAGCCCAT 50711

******************* *** ***************************** ******

Human TCTCTTTTTTATCTCTTCATACGTTTATGAAATTCCCATTCATTCTCTTCTTCCATGTTC 50800

Chimpanzee TCTCTTTTTTATCTCTTCATACGTTTATGAAATTCCCATTCATTCTGTTCTTCCATGTTC 59675

Gorilla TCTCTTTTTTATCTCTTCATACGTTTATGAAATTCCCATTCATTCTGTTCTTCCATGTTT 50771

********************************************** ************

Human AGCTTCAACACTTAATTTTATAAGAAATATTTTTGTTATTGGTTTTGAAATTATTTTCTC 50860

Chimpanzee AGCTTCAACACTTAATTTTATAAGAAATATTTTTATTATTGGTTTTGAAATTATTTTCTC 59735

Gorilla AGCTTCAACACTTACTTTTATAATAAATATTTTTATTATTGGTTTTGAAATTATTTTCTC 50831

************** ******** ********** *************************

Human CTTGAGTCAATAACTTTTAGGCAAATCTTTTGTCAGAGTACACCATCGAGAGATTTAATC 50920

Chimpanzee CTTGAGTCAATAACTTTTAGGCAAATCTTTTGTCAGAGTACACCATCGAGAGATTTAATC 59795

Gorilla CTTGAGTCAATAACTTTTAGGCAAATCTTTTGTCAGAGTACACCATCAAGAGATTTAATC 50891

*********************************************** ************

Human AGTGGGGATAACCGAGGTGGTAGATTTTCAGAGAAGTTGGCTGTACTAAGTGTTTTCTTC 50980

Chimpanzee AGTGGGGATAACCGAGGTGGTAGATTTTCAGAGAAGTTGGCTGTACTAAGTGTTTTCTTC 59855

Gorilla AGTGCGGATAACTGAGGTGGTAGATTTTCAGAGAAGTTGGCTGTACTAAGTGTTTTCTTC 50951

**** ******* ***********************************************

Human ACCTTCTAGAAAACCATTAAACAGAATTCTATCCATTGAACAGAAATCCAGCCCTTGAAT 51040

Chimpanzee ACCTTCTAGAAAACCATTAAACAGAATTCTATCCATTGAACAGAAATCCAGCCCTTGAAT 59915

Gorilla ACCTTCTAGAAAACCATTAAACAGAATTCTATCCATTGAACAGAAATCCAGCCCTTGAAT 51011

************************************************************

Human ATTCACTACTGATTTTTATTTATGAAATTTTTAAAAATTAGGAAATAAAAATATAAAAAT 51100

Chimpanzee ATTCACTACTGATTTTTATTTATGAAATTTTTTAAAATTAGGAAATAAAAATATAAAAAT 59975

Gorilla ATTCACTACTGATTTTTATTTATGAAATTTTTAAAAATTAGGAAATAAAAATATAAAAAT 51071

******************************** ***************************

Human AAATTTTAGTTTCAACTCTACCCACAATCAAAAATTAATTTCAAAATTTAATTTACATTC 51160

Chimpanzee AAATTTTAGTTTCAACTCTACCCACAATCAAAAATTAATTTCAAAATTTAATTTACATTC 60035

Gorilla AAATTTTAGTTTCAACTCTACCCACAATCAAAAATTAATTTCAAAATTTAATTTACATTC 51131

************************************************************

Human ATCTGTGCATTGCTGCCTTTATATAGATGATGAATTAACAGAACTTAGTTAATTCTGTGC 51220

Chimpanzee ATCTGTGCATTGCTGCCTTTATATAGACGATGAACTAAGAGAACTTAGTTAATTCTGTGC 60095

Gorilla ATCTGTGCATTGCTGCCTTTATACAGATGATGAATTAAGAGAACTTAGTTAATTCTGTCC 51191

*********************** *** ****** *** ******************* *

Human TTTAGTCATGGATTTTTATAGTGTTATTGTCTCTACCTCCCAACTGGCCCTCTTACCTGT 51280

Chimpanzee TTTAGTCATGGATTTTTATAGTGTTATTGTCTCTACTTCCCAATTGGCCCTCTTACCTGT 60155

Gorilla TTTAGTCATGGATTTTTATAGTGTTATTGTCTCTACTTCCCAATTGGCCCTCTTACCTGT 51251

************************************ ****** ****************

Human TTTATTTTTAATTGACTTTTAAATGAAATAGCACATTTTATTTGAGGACAGAAATTGGTC 51340

Chimpanzee TTTATTTTTAATTGATTTTTAAATGAAATAGCCCATTTTATTTGAGGACAGAAATTGGTC 60215

Gorilla TTTATTTTTAATTGATTTTTAAATGAAATAGCACATTTTATTTGAGGACAGAAATTGGTC 51311

*************** **************** ***************************

Human CTAAGAATAACATTACTTGCCTGAATCTTTGGCATAGGTACAAGGGCCCTACCTGTGAAG 51400

Chimpanzee CTAAGAATAACATTACTTGCCTGAATCTTTGGCATAGGTACAAGGGCCCTACCTGTGAAG 60275

Gorilla CTAAGAATAACATTACTTGCCTGAATCTTTGGCATAGGTACAAGGGCCCTACCTGTGAAG 51371

************************************************************

Human AGAAAAATATATGGAGGTTCAAAGGTAAGAAGAATATTTTTCCTGATCCTATAAATGCCC 51460

Chimpanzee AGAAAAATATATGGAGGTTCAAAGGTAAGAAGAATATTTT-CCTGATCCTATAAATGCCC 60334

Gorilla AGAAAAATATATGGAGGTTCAAAGGTAAGAAGAATATTTT-CCTGATCCTATAAATGCCC 51430

**************************************** *******************

Human ATTTTTTTT------CCAAATAAAGGTGTTTTATAGAGCAATGTATTAACCTTCTTTATG 51514

Chimpanzee ATTTTTTTTTTTTTTCCAAATAAAGGTGTTTTATAGAGCAATGTATTAACCTTCTTTATG 60394

Gorilla ATTTTTTTT------CCAGATAAAGGTGTTTTATACAGCAATGTATTAACCTTCTTTATG 51484

********* *** **************** ************************

Human TCTTCGGGTTGACATGCATAAGTGTGTTAATACTGACTATTAGGATATTTATAAACATTC 51574

Chimpanzee TCTTCGGGTTGACATGCATAAGTGTGTTAATACTGACTATTAGGATATTTATAAACATTC 60454

Gorilla TCTTCGGGTTGACATGCATAAGTGTGTTAATACTGACTATTAGGGTATTTATAAACATTC 51544

******************************************** ***************

Human AGTCATGGGGTATTTTGTACAGTGGTAGTGCCATGGGGTATCGAGTGGCATGGGGATTGA 51634

Chimpanzee AGTCATGGGGTATTTTGTACAGTGGTAGTGCCATGGGGTATCGAGCGGCATGAGGATTGA 60514

Gorilla AGTCATGGGGTATTTTGTACAGTGGTAGTGCCATGGGGTATCGAGTGGCATGGGGATTGA 51604

********************************************* ****** *******

Human GTGCAGTGGCACAATCATGGCTCACTGCAGCCTCAATCTCCCAGGCTCAAGCAGTCCTCC 51694

Chimpanzee GTGCAGTGGCACAATCATGGCTCACTGCAGCCTCAATCTCCCAGGCTCAAGCAGTCCTCC 60574

Gorilla GTGCAGTGGCACAATCATGGCTCACTGCAGCCTCAATCTCCCAGGCTCAAGCAGTCCTCC 51664

************************************************************

Human TGAAGCTGGCTACCAAGTATCTGGGACCACGGGTGTGCACCACCATACCTGCCTAATCAT 51754

Chimpanzee TGAAGCTGGCTACCAAGTATCTGGGACCACAGGCGTGCACCACCATACCTGCCTAATCAT 60634

Gorilla TGAAGCTGGCTACCAAGTATCTGGGACCACAGGTGTGCACCACCATACCTGCCTAATCAT 51724

****************************** ** **************************

Human TGTTATTTTGTATCTCTGTGTCATCCAGGCTGGTCTCGAAATTCCTGGCCTTGAGCAATC 51814

Chimpanzee TGTTATTTTGTATCTCTGTGTCATCCAGGCTGGTCTCGAAATTCCTGGCCTTGAGCAATC 60694

Gorilla TGTTATTTTGTATCTCTGTGTCATCCAGGCTGGTCTCGAAATTCCTGGCCTTGAGCAATC 51784

************************************************************

Human CTCTTGCCTTAGCCTCCCAAAGTACTAAAATTACAGGTATAAGCCTTTACGCCATGT-GA 51873

Chimpanzee CTCTTGCCTTAGCCTCCCAAAGTACTAAAATTACAGGTATAAGCCTTTACGCCTTGTTAA 60754

Gorilla CTCTTGCCTTAGCCTCCCAAAGTACTAAAATTACAGGTATAAGCCTTCACGCCATGT-GA 51843

*********************************************** ***** *** *

Human AAAAATGCTTTTCATTCAACTTAATTCAATGAGATTTTATAGAGTCTAAACACCGTTCCT 51933

Chimpanzee AAAAATGCTTTTCATTCAACTTAATTCAATGAGATTTTATAGAGTCTAAACACCGTTCCT 60814

Gorilla AAAAATGCTTTTCATTCAACTTAATTCAATGAGATTTTATAGAGTCTAAACACCACTCCT 51903

****************************************************** ****

Human AAAACAACCTCCACCACAAAAAGATAAACCACTTCAGTGTAATATAGAAAGATAGAAAGA 51993

Chimpanzee AAAACAACCTCCACCACAAAAAGATAAACCACTTCAGTGTAATATAGAAAGATAGAAAGA 60874

Gorilla AAAACAACCTCCACCACGAAAAGATATACTACTTCAGTGTAATATAGAAAGATAGAAAGA 51963

***************** ******** ** ******************************

Human TATATAGATAAGGTAAATAGATAGAATGCATAGGTATGGTAATGAGAAAGATAGGTAATA 52053

Chimpanzee TATATAGATAAGGTAAATAGATAGAATGCATAGGTATGGTAATGAGAAAGATAGGTAATA 60934

Gorilla TATATAGATAAGGTAAATAGATAGAATGCATAGGTATGGTAATGAGAAAGATAGGTAATA 52023

************************************************************

Human CCTAAGAATGGATGATAGGTATAGTACATGATAGCTAGATAATTTGATGACAGATATACA 52113

Chimpanzee CCTAAGAATGGATGATAGGTATAGTAGATGATAGCTACATAATTTGATGACGGATATACA 60994

Gorilla CCTAAGAATAGATGATAGGTATAGTAGATGATAGCCAGATAATTTGATGACAGATATACA 52083

********* **************** ******** * ************* ********

Human TCATGATGATGATGGATAATATATAGAGACATATGGTAGATAAATATAGATATAGACAAT 52173

Chimpanzee TCATGATGATGATGGATAATATATAGAGACATATGGTAGATAAATATCGATATAGACAAT 61054

Gorilla TCATGATGGTGATGGATAATATATGGAGACATACGGTAGATAAATATAGATATAGACAAT 52143

******** *************** ******** ************* ************

Human AGATATACAGATATTGTCTGTGTAATAATTAAAACAGCACATACTAAATTTTCAAGATAC 52233

Chimpanzee AGATATACAGATATTGTCTGTGTAATAATTAAAACAGCACATACTAAATTTTCAAGATAC 61114

Gorilla AGATATACAGATATTGTCTGTGTAATAATTAAAACAGCACATACTAAATTTTCAAGATAC 52203

************************************************************

Human AATACTAAACTAATGACAAACAAAAGTGACAATATGTGATTGTTTAAATAGAAAATCTCA 52293

Chimpanzee AATACTAAACTAATGACAAACAAAAGTGACAATATGTGATTGTTTAAATAGAAAATCTCA 61174

Gorilla AATACTAAACTAATGACAAACAAAAGTGACAATATGTGATTGTTTAAATAGAAAATCTCA 52263

************************************************************

Human TAAGTAACATTTAGCAGAATATTGATCAACTGTTTACCCACATACTCTGTCACTAGGCAA 52353

Chimpanzee TAAGTAACATTTAGCAGAATATTGATCAACTGTTTACCCACATAGTCTGTCACTAGGCAA 61234

Gorilla TAAGTAACATTTAGCAGAATATTGATCAACTGTTTACCCACATACTCTGTCACTAGGCAA 52323

******************************************** ***************

Human GTAACATAAGGGGTTCGTTGAAGTATAATTAACACGAACCAAACTGCACTATTAAATGTT 52413

Chimpanzee GTAACATAAGGGGTTCGTTGAAGTATAATTAACACGAACCAAACTGCACTATTAAATGTT 61294

Gorilla GTAACACAAGGGGTTCATTGAAGTATAATTAACATGAACCAAACTGCACGATTAAATGTT 52383

****** ********* ***************** ************** **********

Human TTACTTAAAAAGAAATTATTTTACCAAATGTATTATGAATTAAATAATGAAAAAATAATA 52473

Chimpanzee T-ACTTAAAAAGAAATTATTTTGCCAAATGTATTATGAATTAAATAATGAAAAAATAATA 61353

Gorilla TTACTTAAAAAGAAATTATTTTACCAAATGTATTATGAATTAAATAATGAAAAAATAATA 52443

* ******************** *************************************

Human ATTGTATATCCAATTTAAGTAAAAAATACAAGAATCAGGGAAACTTAAAGTGAGGGGAAA 52533

Chimpanzee ATTGTATATCCAATTTAAGTAAAAAATACAAGAATCAGGGAAACTTAAAGGGAGGGGAAA 61413

Gorilla ATTGTATATCCAATTTAAGTAAAAAATACAAGAATCAGGGAAACCTAAAGTGAGGGGAAA 52503

******************************************** ***** *********

Human CCTTGGGAATAATTTATTCTAACTCTGATACTTAGATAGCAATTATTTTTAACTTTGTTC 52593

Chimpanzee CCTTGGGAATAATTTATTCTACCTCTGATACTTAGATAGCAATTATTTTTAACTTTGTTC 61473

Gorilla CCTTGGGAATAATTTATTCTAACTCTGATACTTAGATAGCAATTATTTTTAACTTTGTTC 52563

********************* **************************************

Human TAACTTTATGAGTTCATTGTAAGAGTTTACAATGCTGAACTGAGATGTCAAATTCAAGTA 52653

Chimpanzee TAACTTTATGGGTTCATTGTAAGAGTTTACAATGCTGAATTGAGATGTCAAATTCAAGTA 61533

Gorilla TAACTTTATGGGTTCATTGTAAGAGTTTACAATGCTGAATTGAGATGTCAAATTCAAGTA 52623

********** **************************** ********************

Human ACCTAGAAAAGAATGTAATGGACTGATTTACTCCACACCTGTCTCACTCATCACCACAGA 52713

Chimpanzee ACCTAGAAAAGAATGTAATGGACTGATTTACTCCACACCTGTCTCACTCATCACCACAGA 61593

Gorilla ACCTAGAAAAGAATGTAATGGACTGATTTACTCCACACCTGTCTCACTCATCACCACAGA 52683

************************************************************

Human AATGAAACTACAGATACACACACGTAAAAAAGTTGATTGCATAAAATGTAATATCATGAT 52773

Chimpanzee AATGAAATTAGAGATACACACATGTAAAAAAGTTGATTGCATAAAATGTAATATCATGAT 61653

Gorilla ACTGAAATTACAGATACACATATGTAAAAAAATTGATTGCATAAAATGTAATATCATGAT 52743

* ***** ** ********* * ******** ****************************

Human CTCTTGAAGTTGATTCCAGGAACACATAATATTTTTCTTAGTGCTGGAATATTTTTATAT 52833

Chimpanzee CTCTTGAAGTTGATTCCAGGAACACATAATATTTTTCTTAGTGCTGGAATATTTTTATAT 61713

Gorilla CTCTTGAAGTTGATTCCAGGAACACATAATATTTTTCTTAGTGCTGGAATATTTTTATAT 52803

************************************************************

Human ATAATTTACACTCACTAATATATACACCATCATCTCAATATGTTCACTTATTTATAGATA 52893

Chimpanzee ATAATTTACACTCACTAATGTATGCACCATCATCTCAATATGTTCACTTATTTATAGATA 61773

Gorilla ATAATTTACACTCACTAATATATACACCATCATCTCAATATGTTCACTTATTTATAGATA 52863

******************* *** ************************************

Human CAAATAAATTCATAATATATTATAAACTTTCATGCACAGAGCTCTTAGAAAACTGGAATT 52953

Chimpanzee CAAATAAATTCATAATATATTATAAACTTTCATGCGCAGAACTCTTAGAAAACTGGAATT 61833

Gorilla CAAATAAATTCATAATATATTATAAACTTTCATGCACAGAACTCTTAGAAAACTGGAATT 52923

*********************************** **** *******************

Human CAAAGGTGCTTTCTTGATGAGGGACAGGACACTAAGAAAACCTATAGCAAACAATGTTTG 53013

Chimpanzee CAAAGGTGCTTTCTTGATGAGGGACAGGACACTAAGAAAACCTATAGCAAACAACGTTTG 61893

Gorilla CAAAGGTACTTTCTTGATGAGGGACAGAANACTAAGAAAACCTATAGCAAACAATNTTTG 52983

******* ******************* * ************************ ****

Human TTGTATTTAAGATCAGGAAATGCAATAATGCTTTTTATCAGAGCCTCTCTTCTTTATTTT 53073

Chimpanzee TTGTATTTAAGATCAGGAAATGCAATAATGCTTTTTATCAGAGCCTCTCTTCTTTATTTT 61953

Gorilla TNNNNNNNNAGATCNNNNNNNNNNNNNNNNNNNNNNNNNNNNNNNNNNNNNNNNNNNNNN 53043

* *****

Human GCTGGATAGAAGAGGAAAGAAAGAACAGTTTTATGAATGGAAAAGGAGGAAAATGTCACT 53133

Chimpanzee GCTGGATAGAAGAGGAAAGAAAGAACAGTTTTATGAATGGAAAAGGAGGAAAATGTCACT 62013

Gorilla NNNNNNNNNNNNNNNNNNNNNNNNNNNNNNNNNNNNNNNNNNNNNNNNGANNNNNNNNNN 53103

**

Human ATTTATCAATCATATGATAATACATAGAGAAAATCTACACAAATATACTGTCAAACTCAT 53193

Chimpanzee ATTTATCAATCATATGATAATACATAGAGAAAATCTACACAAATATACTGTCAAACTCAT 62073

Gorilla NNNNNNNNNNNNNNNNNNNNNNNNNNNNNNNNNNNNNNNNNNNNNNNNNNNNNNNNNNNN 53163

Human ATTGGTCAAATCAACATGAATATTTAATAGTGTTGTTAGATATAAAATCAGCTTTTAAAA 53253

Chimpanzee ATTGGTCAAATCAACATGAATATTTAATAGTGTTGTTAGATACAAAATCAGCTTTTAAAA 62133

Gorilla NNNNNNNNNNNNNNNNNNNNNNNNNNNNNGTGTTGTTAGACATAAAATCAGCTTTTAAAA 53223

*********** * *****************

Human ATCAATTTTCTTATCTTCAATAGTAATAATCATTTAGAAAATGCAGATTATCATATAATT 53313

Chimpanzee ATCAATTTTCTTATCTTCAATAGTAATAATCATTTAGAAAATGCAGATTATCATATAATT 62193

Gorilla ATCAATTTTCTTATCTTCAATAGTAATAATCATTTAGAAAATGCAGATTATCATATAATT 53283

************************************************************

Human TATAAAGACAACAAACAAAAATTTTACTTGACAAAATATAATTAGACTCAATAAATAATG 53373

Chimpanzee TATAAAGACAACAAACAAAAATTTTACTTGACAAAATATACTTAGACTCAATAAATAATG 62253

Gorilla TATAAAGACAACAAACAAAAATTTTACTTGACAAAATATAATTAGACTCAATAAATAATG 53343

**************************************** *******************

Human ATGACAATTGTCTCTTAATTAACCTATGGAGTCAATAAAATTTAAATAAAATTCCCAAAA 53433

Chimpanzee ATGTCAATTGTCTCTTAATTAACCTATGGAGTCAATAAAATTTAAATAAAATTCCCAAAA 62313

Gorilla ATGTCAATTGTCTCTTAATTAACCTATGGAGTCAATAAAATTTAAATAAAATTCCCAAAA 53403

*** ********************************************************

Human AGATGTTTTGTAGAAATCAATAAGCTAATCCAAAAATTTATATAGAGAGACAATTGCATG 53493

Chimpanzee AGAGGTTTTGTAGAAATCAATAAGCTAATCCAAAAATTTATATAGAGAGACAATTGCATG 62373

Gorilla AGATGTTTTGTAGAAATCAATAAGCTAATCCAAAAATTTATATAGAGAGACAATTGCATG 53463

*** ********************************************************

Human AGATACACCAAGAAACCCTTTATGGAAATGATGAAGGTGGAAGATGTGTTCCACGAGATT 53553

Chimpanzee AGATACACCAAGAAACCCTTTATGGAAATGATGAAGGTGGAAGATGTGTTCCACGAGATT 62433

Gorilla AGATACACCAAGAAACCCTTTATGGAAATGATGAAGGTGGAAGATGTGTTCCACGAGATT 53523

************************************************************

Human CCATATGCCTTACTGTTAAATCTTAGAAGAGCATAATACTGATGCTAGGATAGGTTTACC 53613

Chimpanzee CCATATGCCTTACTGTTAAATCTTAGAAGAGCATAATATTGATGCTAGGATAGGTTTACC 62493

Gorilla CCATATGCCTTACTGCTAAATCTTAGAAGAGCATAATATTGATGCTAGGATAGGTTTACC 53583

*************** ********************** *********************

Human TACCAACAAAAAAAAAGTGAGACCAAACAGACATTTATGGAAACATAATTTATTCATGAA 53673

Chimpanzee TACCAACAAAAAAAAAGTGAGACCAAACAGACATTTATGGAAACATAATTTATTCATGAA 62553

Gorilla TACCAACAAAAAAAAAGTGAACCCAAACAGACATTTATGGAAACATAATTTATTCATGAA 53643

******************** **************************************

Human TGAAGAAAGGTAAACATTTTAGTAAAGTATCTTGTACAAATTATAAAATTTAAATAAGTA 53733

Chimpanzee TGAAGAAAGGTAAACATTTTAGTAAACTATCTTGTACAAATTATAAAATTTAAATAAGTA 62613

Gorilla TGAAGAAAGGTAATCATTTTAGTAAAGTATCTTGTACAAATTATAAAATTTAAATAAGTA 53703

************* ************ *********************************

Human ATTACATTAAAAAATAATTACAGGTTTGCTTAGACCTTAAAGCCTGATATTAGAACTTTA 53793

Chimpanzee ATTAAATTAAAAAATAATTACAGGTTTGCTTAGACCTTAAAGCCTGATATTAGAACTTTA 62673

Gorilla ATTAAATTAAAAAATAATTACAGGTTTGCTTAGACCTTAAAGCCTGATATTAGAACTTTA 53763

**** *******************************************************

Human AAACTTAGGAGCAATTAAAAACCTTAAAAATATCAAAGTGTTTGAAAATATCTTTATCAT 53853

Chimpanzee AAACTTAGGAGCAATTAAAAACCTTAAAAATATCAAAGTGTTTGAAAATATCTTTATCAT 62733

Gorilla AAACTTAGGAGCAATTAAAAACCTTAAAAATATCAAAGTGTTTGAAAATATCTTTATCAT 53823

************************************************************

Human ACTGTAGTGGGATTGATAAGGAATCAGAAAGACTGATGGGGTTGAGGAGGATATTTATTT 53913

Chimpanzee ACTGTAGCGGGATTGATAAGGAATCAGAAAGACTGATGGGGATGAGGAGGATATTTATTT 62793

Gorilla ACTGTAGCGGGATTGATAAGGAATCAGAAAGACTGATGGGGTTGAGGAGGATATTTATTT 53883

******* ********************************* ******************

Human TTTAGGTGCACCAGTCCAGTCAGATTAACATGCAAAGGACTGAGCACTGAACAAAGAGGT 53973

Chimpanzee CTTAGGTGCACCCGTCCAGTCAGATTAACATGCAAAGGACTGAGCACTGAACAAAGAGGT 62853

Gorilla TTTAGGTGCACCAGTCCAGTCAGATTAACATGCAAAGGACTGAGCACTGAACAAAGAGGT 53943

*********** ***********************************************

Human AAGTTACATTTTAAGCATTTTGTGGGTTGGGGGTAGATCTGTGCAGGGACAAGCATATTA 54033

Chimpanzee AAGTTACATTTTAAGCATTTTGTGGGTTGGGGGTGGATCTGTGCAGGGACAAGCGTATTA 62913

Gorilla AAGTTACATTTTAAGCATTTTGTGGGTTGGGGGTAGATCTGTGCAGGGATAAGCATATTA 54003

********************************** ************** **** *****

Human CAGAAGTGAGAAACAAAGACAGTTATTCAATTAAGACACGCATTACATCATTTCTACTTT 54093

Chimpanzee CAGAAGTGAGAAACAAAGACAGTTATTCAATTAAGACACGCATTACATAGTTTCTACTTT 62973

Gorilla CAGAAGTGAGAAACAAAGACAGTTATTCAATTAAGACATGCATTACATCATTTCTACTTT 54063

************************************** ********* **********

Human TCAAGGAAAAACATGTTTTATGACTTGAGTTTATCTGTCTAGTGACCTTGCAACTGCACA 54153

Chimpanzee TCAAGGAAAAACATGTTTTATGACTTGAGTTTATCTGTCTAGTGACCTTGCAACTGCACA 63033

Gorilla TCAAGGAAAAACATGTTTTATGACTTGAGTTTATCNNTCTAGTGACCTTGCAACTGCACA 54123

*********************************** ***********************

Human GCTGGAGAAACAGGATCTTCGCCATGCCTGGGAAAGTAGGAGAGATAAGGCTCACTAGTG 54213

Chimpanzee GCTGGAGAAACAGGATCTTCGCCATGCCTGGGAAAGTAGGAGAGATAAGGCTCACTAGTG 63093

Gorilla GCTAGAGAAACAGGATCTTCGCCATGCCTGGGAAAGTAGGAGAGATAAGGCTCACTAGTG 54183

*** ********************************************************

Human ACAGAAAAACAGGCAGTTAATTTATAAAGCGCTCCAGCTCTTTCTCTTTCTCGG----GG 54269

Chimpanzee ACAGAAAAACAGGCAGTTAATTTATAAAGTGTTCCAGCTCTTTCTCTTTCTCGG----GG 63149

Gorilla ACAGAAAAACANNNNNNNNATTTATAAAGTACTCCAGCTCTTNNNCTTTCTCGG----CN 54239

*********** ********** ********** *********

Human ATTTTTTTTTTTTTTTTTTTTTTTTTTT----ACATATAACAGAGTTTCTGCTTACATAT 54325

Chimpanzee ATTTTTTTTTTTTTTTTTTTTTTTTTTTTTTTACATATAACAGAGTTTCTGCTTACATAT 63209

Gorilla NNTTTTTTTTTTTNNNNNNNNNNNNNNN----ACATATAACAGAGTTTCTGCTTACATAT 54295

*********** ****************************

Human TCTTTAATTTCCTTTCATTGCTGCTCCAATACTAGATGATTAAGTATATAAAAAACTGGG 54385

Chimpanzee TCTTTAATTTCCTTTCATTCCTGCTCCAATACTAGGTGATTAAGTATATAAAAAACTGGG 63269

Gorilla TATTTAATTTCCTTTCATTCCTGCTCCAATACTAGATGATTAAGTATATAAAAAACTGGG 54355

* ***************** *************** ************************

Human CCAGACATGGTGGCTCAGACCTGTAATCTCAACATTTTGGGAGGCCAAGGCAGATGGATC 54445

Chimpanzee CCAGACATGGTGGCCCAGACCTGTAATCTCAACATTTTGGGAGGCCAAGGCAGATGGATC 63329

Gorilla CCAGACATGGTGGCTCAGACCTGTAATCTCCACATTTTGGGAGGCCAGGGCAGATGGANN 54415

************** *************** **************** **********

Human ACCTGAGGCCAGGCATTAGAGACCAACCTGGCCAACATGGCAAAAACATATCTACTAAAA 54505

Chimpanzee ACCTGAGGCCAGGCATTAGAGACCAGCCTGGCCAACATGGCAGAAACCTATCTACTAAAA 63389

Gorilla NCCTGAGNNNNNGCATTAGAGACCAGCCTGGCCAACATGGCAAAAACCTATCTACTAAAA 54475

****** ************* **************** **** ************

Human ATACAAATATTAGCTGGCTATGATGATGGGTGCCTGTAATCCCAGCAACTCAGGAGTCTG 54565

Chimpanzee ATACAAATACTAGCTGGCTATGATGACGGGTGCCTGTAATCCCAGCAACTCAGGAGTCTG 63449

Gorilla ATACAAATATTAGCTGGCTATGATGACAGGTGCCTGTAAGCCCAGNNNNNNNNNNNNNNN 54535

********* **************** *********** *****

Human AGGCACGGGAATCACTTGAACCTAGGAGGCAGATGTTGCATTGAGCAGAGATTACAGCAA 54625

Chimpanzee AGGCACGGGAATCACTTGAACCTAGGAGGCAGATGTTGCATTGAGCAGAGATTACAGCAA 63509

Gorilla NNNNACGGGAATCACTTGAACCTAGGAGGCAGATGTTGCATTGAGCAGAGATTACAGCAA 54595

********************************************************

Human CACACTCCAGCCTGGGCCACAGAGTGAAACTCTGTAATCTTAAACAAAGAAAAGGAATAT 54685

Chimpanzee CACACTCCAGCCTGGGCCACAGAGTGAAACTCTGTAATCTTAAACAAAGAAAAGGAATAT 63569

Gorilla CACACTCCAGCCTGGGCCACAGAGTGAAACTCTGTAATCTTAAAAAAAGAAAAGGAATAT 54655

******************************************** ***************

Human AAAAAACTATAACCAAGGTTAAAATTTTGATTAATTCAACTACATTAAAGTTAAAAATTT 54745

Chimpanzee AAAAAACTATAACCAAGGTTAAAAGTTTGATTAATTCAACTACATTAAAGTTAAAAATTT 63629

Gorilla AAAAAACTATAACCAAAGTTAAAAGTTTGATTAATTCAACTACATTAAAGTTAAAAATTT 54715

**************** ******* ***********************************

Human TGCCCATCCAATGTTAAAATTGAGTGATATGTGATGCATCTAATTTGGAGAATGTATCTT 54805

Chimpanzee TGCCCATCCAATGTTAAAATTGAGTGATATGTGATGCATCTAATTTGGAGAATGTATCTT 63689

Gorilla TGCCCATCCAATGTTAAAATAGAGTGGTATGTGATGCATCTAATTTGGAGAATGTATCTT 54775

******************** ***** *********************************

Human CAATATATTTTACCAGAAAATATACCAATAACATATATACATGCATATACTATGCT-ATA 54864

Chimpanzee CAATATATTCTACCAGCAAATATACCAATAACATATATACATGCATATACTATGCTTATA 63749

Gorilla CAATATATTTTATCAGCAAATATACCAATAACATATATACATGCATATACTATGCT-ATA 54834

********* ** *** *************************************** ***

Human ATAATGGGAATTTTTCAAACAGGTGGTGTAGAAAAGTTATCATTTGGATTTTATAAAAAT 54924

Chimpanzee ATAATGGGAATTTTTCAAACAGGTGGTGTAGAAAAGTTATCATTTGGGTTTTATAAAAAT 63809

Gorilla ATAATGGGAATTTTTGAAACAGGTGGTGTAGAAAAGTTATCATTTGGATTTTATAAAAAT 54894

*************** ******************************* ************

Human ATAAAACCATAAAGTTTGTTCATAAAAAATTATAAATTTCACTAACAACGAGGCATCATT 54984

Chimpanzee ATAAAACCATAAAGTTTGTTCATAAAAAATTATAAATTTCATTAACAACGAGGCATCATT 63869

Gorilla ATAAAACCATAAAATTTGTTCATAAAAAATTATAAATTTCATTAACAACGAGGCATCATT 54954

************* *************************** ******************

Human TCTTACCATATAGTTGGCTGAAACACTGTGATGTTGAGGCTTAGATCAGTTTGAACTTTC 55044

Chimpanzee TCTTACCATATAGTTGGCTGAAACACTGTGATGTTGAGGCTTAGATCAGTTTGAACTTTC 63929

Gorilla TCTTACCATATAGTTGGCTGAAACACTGTGATGTTGAGGCTTAGATCAGTTTGAACTTTC 55014

************************************************************

Human TCTCATATCTACTTGGATTATAAATTGTTACCGTGCTTTCAGAATACAGTTTGCTGTCAC 55104

Chimpanzee TCTCATACCTACTTGGATTATAAATTGTTACCGTGCTTTCAGAATACAGTTTGCTGTCAC 63989

Gorilla TCTCATATCTACTTGGATTATAAATTGTTACCGTGCTTTCAGAATACAGTTTGCTGTCAC 55074

******* ****************************************************

Human TCTACAATGTCTGTTCTAAAAATGAACCATAAGTGTAGAACTCTACTTGAATGCATATGT 55164

Chimpanzee TCTACAATGTCTGTTCTAAAAATGAACCATAAGTGTAGAACTCTACTTGAATGCATATGT 64049

Gorilla TCTACAATGTCTGTTCTAAAAATGAACCATAAGTGTAGAATTCTACTTGAATGCATATGT 55134

**************************************** *******************

Human AACAGATACTCAGGAACTCCTTACACCATGAAATGCATATAGGGGTATTTCTAACAACGT 55224

Chimpanzee AACAGATACTCAGGAACTCCTTACACCATGAAATGCATATAGGGGTATTTCTAACAACGT 64109

Gorilla AACAGATACTCAGGAACTCCTTACACCATGAAATGCATATAGGGGTATTTCTAACAACGT 55194

************************************************************

Human CTTTTGACGGAGCTAGAAAATATAATATTCATCACGATCAACTGAAGTTCATTTCAGGAA 55284

Chimpanzee CTTTTGATGGAGCTAGAAAATATAATATTCATCACGATCAATTGAAGTTCATTTCAGGAA 64169

Gorilla CTTTTGACGGAGCTAGAAAATATAATATTCATCACAATCAATTGAAGTTCATTTCAGGAA 55254

******* *************************** ***** ******************

Human TGCACAACATTTTTCCTAGTGCTTGAATATTTTTAAAATATAATTCAAACATGTTAATAT 55344

Chimpanzee TGCACAACACTTTTCCTAGTGCTTGAATATTTTAAAAATATAATTCAAACATGTTAATAT 64229

Gorilla TGCACAACATTTTTCCTAGTGCTTGAATATTTTTAAAATATAATTCNAACNNNNNNNNNN 55314

********* *********************** ************ ***

Human ATCAACTATAATCTAAATAGATTCACTTATCAATAGATGCCAAAATATCAATAAAATATT 55404

Chimpanzee ATCAACTATAATCTAAATAGATTCACTTATCAATAGATGCCAAAATATCAATAAAATATT 64289

Gorilla NNNNNNNNNNNNNNNNNNNNNNNNNNNNNNNNNNNNNNNNNNNNNNNNNNNNNNNNNNNN 55374

Human ACAAACATACATGCACAGAACTCTCATAAAAGTGAAATTCAAAGGAGCTTCAGAATGCTG 55464

Chimpanzee ACAAACATACATGCACAGAACTCTCATAAAAGTGAAATTCAAAGGAGCTTCAGAATGCTG 64349

Gorilla NNNNNNACACATGCACAGNNNNNNNNNNNNNNNNNNNNNNNNNNNNNNNNNNNNNGGCTG 55434

* ********** ****

Human AAAAAAACTCAAAATGCACACATACTTATCAAAAAGAAGATGGATAATCAATTTGTGTTA 55524

Chimpanzee AAAAAAACTCAAAATGCACACATACTTATCAAAAAGAAGATGGATAATCAATTTGTGTTA 64409

Gorilla AAAAAAACTCAAAATGCACACATACTTATCAAAAAGAAGATGGATAATNNNNNNNNNNNN 55494

************************************************

Human AATCAGTTACTCAGAAATATTATACAGCAATGAAGATCACTAGACTTCATGATTATGAAT 55584

Chimpanzee AATCAGTTACTCAGAAATATTATACAGCAATGAAGATCACTAAACTTCATGATTATGAAT 64469

Gorilla NNNNNNNNNNNNNNNNNNNNNNNNNNNNNNNNNNNNNNNNNNNNNNNNNNNNNNNNNNNN 55554

Human CACCCTGCGTGAATCTCTGAAAAATACCGTGTTCAAGGAAAGAACCTAAGGAAGACACTA 55644

Chimpanzee CACCCTGCGTGAATCTCTGAAAAATACCGTGTTCAAGGAAAGAACCTAAGGAAGACATTA 64529

Gorilla NNNNNNNNNNNNNNNNNNNNNNNNNNNNNNNNNNNNNNNNNNNNNNNNNNNNNNNNNNNN 55614

Human TAAACAGTATGATTCTCTTACATAACATTCAAATTCAAGCAAAACTTAAGAATGTATTCT 55704

Chimpanzee TAAACAGTATGATTCTCTTACATAACATTCAAATTCAAGCAAAACTTAAGAATGTATTCT 64589

Gorilla NNNNNNNNNNNNNNNNNNNNNNNNNNNNNNNNNNNNNNNNNNNNNNNNNNAATGTANNNN 55674

******

Human CTAACAATAGAGTAGTGCTAGAAAAGTTAGCAGGACATTCAGTATAGTTGTCTCCTTTGA 55764

Chimpanzee CTAACAATAGAGTACTGCTAGAAAAGTTAGCAGGACATTCAGTATAGTTGTCTCTTTTGA 64649

Gorilla NNNNCAATAGAGTAGTGCTAGAAAAGTTAGCAGGNNATTCANNATAGTTGTCTCCTTTGA 55734

********** ******************* ***** *********** *****

Human TGAGGAAGGAAAATATTGATTGAAAACAGAAATTAGGCAGCATTCATTTACATTTCCATT 55824

Chimpanzee TGAGGAAGGAAAATATTGATTGAAAACAGAAATTAGGCAGCATTCATTTACATTTCCATT 64709

Gorilla TGAGGAAGGAAAATATTGATTGAAAACAGAAATTAGGCAGAATTCATTTACATTTCCATT 55794

**************************************** *******************

Human CCATACCTCAGTGTCTCTTCCACAGGTGTGTGGCCTCAAGCAATCCCCAGGGTCCTGGGG 55884

Chimpanzee CCATACCTCAGTGTCTCTTCCACAGGTGTGTGGCCTCAAGCAATCCACAGGGTCCTGGGG 64769

Gorilla CCATACCTCAGTGTCTCTTCCACAGGTGTGTGGCCTCAAGCAATCCACAGGGTCCCGGGN 55854

********************************************** ******** ***

Human TTCAGAGCGCCCCATGCTAGGTTTAGAGTTCTGCTACTGCTGTCTTGAAATTCTGAA-GA 55943

Chimpanzee TTCAGAGCGCCCCATGCTAGGTTTAGAGTTCTGCTACTGCTGTCTTGAAATTCTGAAAGA 64829

Gorilla NNNNNNNNNNNNNNNNNNNNNNNNNNNNNNNNNNNNNNNNNNNNNNGAAATTCTGAA-GA 55913

*********** **

Human ATTAATCTTTGCACTTTCCTTTCATATGTGGAATGCAATGAAAGAAGAGGAATGCATGCA 56003

Chimpanzee ATTAATCTTTGCACTTTCCTTTCATATGTGGAATGCAATGAAAGAAGAGGAATGCATGCA 64889

Gorilla ATTAATCTTTGCACTTTCCTTTTATATGTGGAATGCANNNNNNNNNNNNNAATGCATGCA 55973

********************** ************** **********

Human CATGGTTAGAGGAGATATGCACCATATGCACTTCTGCCACTGTTCTTTCCTACCCAATTG 56063

Chimpanzee CATGGTTAGAGGAGATATGCACCATATGCACTTCTGCCACTGTTCTTTCCTACCCAGTTG 64949

Gorilla CATGGTTAGAGGAGATATGCAGCATATGCACTTCTGCCACTGTTCTTTCCTACCCAATTG 56033

********************* ********************************** ***

Human CATACAGTGTTCATGAACCCTCTTGGGCACAGAATTCTGCTGGACTGGCAATAAGGGAAA 56123

Chimpanzee CATACAGTGTTCATGAACCCTCTTGGGCACAGAATTCTGCTGGACTGGCAATAAGGGAAA 65009

Gorilla CATACAGTGTTCATGAACCCTCTTGGGCACAGAATTCTGCTGGACTGGCAATAAGGGAAA 56093

************************************************************

Human ATTCAGTAAGATTCACAAAGTGAGGTTATTACTTCTATGACAAAGTGAGTGCTCTTGCAT 56183

Chimpanzee ATTCAGTGAGATTCACAAAGTGAGGTTATTACTTCTATGACAAAGTGAGTGCTCTTGCAT 65069

Gorilla ATTCAGTGAGATTCACAAAGTGAGGTTATTACTTCTATGACAAAGTGAGTGCTCTTGCAT 56153

******* ****************************************************

Human CCCAGAGAGGTCATGCTTTGAGTACAAATTAGAACCTACTTTAAATATAGATAAGTGGCA 56243

Chimpanzee CCCAGAGAGGTCATGCTTTGTGTACAAATTAGAACTTACTTTAAATATAGATAAGTGGCA 65129

Gorilla CCCAGAGAGGTCATGCTTTGTGTANNNNNNNNNNNNTACTTNNNNNNNNNNNNNNNGGCA 56213

******************** *** ***** ****

Human ATGAAATTCTAAAAAATCCCAGAACAAACAAGAACTCTATCCTATCTCTTATTCTTCTTG 56303

Chimpanzee ATGAAATTCTAAAAAATCCCAGAACAAACAAGAATTCTATCCTATCTCTTATTCTTCTTG 65189

Gorilla ATGAAANNNNNNNNNNNNNNNNNNNNAACAAGAACTCTATCCTATCTCTTATTCTTCTTG 56273

****** ******** *************************

Human CTGTGTCAGGCAACCACTTATGTGGAAAATGATGTTATAGAGGAGATGGGGATGAGAGGA 56363

Chimpanzee CTGTGTCAGGCAACCACTTATGCGGAAAATGATGTTATAGAGGAGATGGGGATGAGAGGA 65249

Gorilla CTGTGTCAGGCAACCACTTATGTGGAAAATGATGTTATAGAAGACATGGGGATGAGAGGA 56333

********************** ****************** ** ***************

Human CAACCTGTGGTTCCTGTTAGTTACATGCCTTCCTTATCATCAGTGAGCTGAAGGTACAGT 56423

Chimpanzee CAACCTGTGGTTCCTGTTAGTTACATGCCTTCCTTATCATCAGTGAGCTGAAGGTACAGT 65309

Gorilla CAACCTGTGGTTCCTGTTAGTTACATGCCTCCCCTATCATCAGTGAGCTGAAGGTACAGT 56393

****************************** ** **************************

Human GTTGCTAGAATATGTCAATATCAGGAAGTAAAATAAAATCAGCTGAGTTCATTTTGTGTA 56483

Chimpanzee GTTGCTAGAATATGTCAATATCAAGAAGTAAAATAAAATCAGCTGAGTTCATTTTGTGTG 65369

Gorilla GTTGCTAGAATATGTCAATATNNNNNNNNNNNNNNNNNNNNNNNNNNNNNNNNNNNNGTA 56453

********************* **

Human GCACACTTCCCCTGTTCTGGTAAGAATGCAGTAGATACTTATGCATATGCTACAAAATAA 56543

Chimpanzee GCACACTTCCCCTGTTCTGGTAAGAATGCAGTAGATACTTATGCATATGCTACAAAATAA 65429

Gorilla GCACACTTCCCCTGTTCTGGTAAGAATGCAGTAGATACTTATGCATANNNNNNNNNNTAA 56513

*********************************************** ***

Human CAGCTTGTGTAATTTCAGTGATTCTGAATACATGTTAACTGCTCAGATACTGGAGTTCAA 56603

Chimpanzee CAGCTTGTGTAATTTCAGTGATTCTGAATACATGTTAACTGCTCAGATACTGGAGTTCAA 65489

Gorilla CAGCTTGTGTANNNNNNNNNNNNNNNNNNNNNNNNNNNNNNNTCAGATACTGGAGTTCAA 56573

*********** ******************

Human AATTGGCATTACACAATATAAAGGTAAAAATGGAAAACCATGCCAGTGACTAAATATTTT 56663

Chimpanzee AATTGGCATTACACAATATAAAGGTAAAAATGGAAAACCATGCCAGTGACTAAATATTTT 65549

Gorilla AATTGGCATTACACAATANNNNNNNNNNNNNNNNNNNNNNNNNNNNNNNNNNNNNNNNNN 56633

******************

Human ATTTTTTCCTTACTTAGAACAACATCAAATATAAATTTATTTTGTAAAGACATGACAAGT 56723

Chimpanzee ATTTTTTCCTTACTTAGAACAACAGCAAATATAAATTTATTTTGTAAAGACATGACAAGT 65609

Gorilla NNNNNNNNNNNNNNNNNNNNNNNNNNNNNNNNNNNNNNNNNNNNNNNNNNNNNNNNNNNN 56693

Human TGAGAAAGATTAGAAAAAAAGGGGGAGACAGCTTTATATTTTAGTACTGTTAATAGTTCT 56783

Chimpanzee TGAGAAAGATTAGAAAAAAAGGGGGAGACAGCTTTATATTTTAGTACTGTTAATAGTTCT 65669

Gorilla NNNNNNNNNNNAGAAAAAAAGGGAGAGACAGCTTTATATTTTAGTAGTGTTAATAGTTCT 56753

************ ********************** *************

Human ATGAAATTGGCCTACTTTTAATAAAAAGAGTCAAATAGTAAACATTTTCATCTCTGTGAG 56843

Chimpanzee ATGAAATTGGCCTACTTTTAATAAAAAGAGTCAAATAGTAAATATTTTCATCTCTGTGAG 65729

Gorilla ATGAAATTGGCCTACTTCTAATAAAAAGAGTCAAATAGTAAATATTTTCATCTCTGTGAG 56813

***************** ************************ *****************

Human ATACATAATCTCTGTTGTAGTAACTGAGCTCTTCTATCATACCACAAAAGCAGGCACAGA 56903

Chimpanzee ATACATAAACTCTCTTGTAGTAACTGAGCTCTTCTATCATACCACAAAAGCAGGCACAGA 65789

Gorilla ATACATAAACTCTGTTGTAGTAACTGAGCTCTGCTATCATACCACAAAAGCAGGCACAGA 56873

******** **** ****************** ***************************

Human CAATATACAAATGAATGGGCATGGCTGTTTTCCAGTAAAACTTTATTTACAGAAACAAGT 56963

Chimpanzee CAATATATAAATGAATGGTCATGGCTGTTTTCCAGTAAAACTTTATTTACAGAAACAAGT 65849

Gorilla CAATATATAAATGAATGGGCATGGCTGTTTTCCAGTAAAACTTTATTTACAGAAACAAGT 56933

******* ********** *****************************************

Human AGTGGGCTGGATTTGACCCACGGAACGTAATTAGTCCGTGGATGTCCTAAGGTACGAAGT 57023

Chimpanzee AGTGGGCTGGATTTGACCCACGGAATGTAATTAGTCCGTGGATGTCCTAAGGTAAGAAGT 65909

Gorilla AGTGGGCTGGATTTGGCCCACGGAACGTAATTAGTCCGTGGATGTCCTTAGGTAAGAAGT 56993

*************** ********* ********************** ***** *****

Human CCACTGTAAGACTTCTTAGCAATTCAAACAACATCAGGGGAAACTCTCCTAAAACCTTAT 57083

Chimpanzee CCACTGTAAGACTTCTTAGCAATTCAAACAACATCAGGGGAAACTCCCCTAAAACCTTAT 65969

Gorilla CCACTGTAAGACTTCTTAGCAATTCAAACAACATCAGGGGAAACTCTCCTAAAACCTTAC 57053

********************************************** ************

Human AAATTTTTAAACATTTCTGAGTTTCAGGCTAATGAGTCATTTGCATGATGCTGGGCAGTA 57143

Chimpanzee AAATTTTTAAACATTTCTGAGTTTCAGGCTAATGAGTCATTTGCATGATGCTGGGCAGTA 66029

Gorilla AAATTTTTAAACATTTCTGAGTTTCAGGCTAATGAGTCATTTGCATGATGCTGGGCAGTA 57113

************************************************************

Human ATTGTAGATACATTTACAGCAACATTTTCAGTGTTTCAGAGTTACAGATGAAGGGATTGA 57203

Chimpanzee ATTGTAGATACATTTATAGCAACATTTTCAGTGTTTCAGAGTTACAGATGAAGGGATTGA 66089

Gorilla ATTGTAGATAAATTTATAGCAACATTTTCAGTGTTTCAGAGTTACAGATGAAGGGATTGA 57173

********** ***** *******************************************

Human CTCAGGTAGTTCCACTATGGATCATTGAGTCTATTTTCATGCTGCTCTTTTGGCAAAGTA 57263

Chimpanzee CTCAGGTAGTTCCACTATGGATCATTGAGTCTATTTTCATGCTGCTCTTTTGGCAAAGTA 66149

Gorilla CTCAGGTAGTTCCACTATGGATCATTGAGTCTATTTTCATGCTGCTCTTTTGGCAAAGTA 57233

************************************************************

Human TAGGAATAATGCCAGCAGTCTAGTGATATAGAATGTCTGTTTCTTGAAGCTATGGGTTAT 57323

Chimpanzee TAGGAATAATGCCAGCAGTCTAGTGATATAGAATTTCTGTTTCTTGAAGCTATGGGTTAT 66209

Gorilla TAGGAAAAATGCCAGCAGTCTAGTGATATAGAATTTCTGTTTCTTGAAGCTATGGGTTAT 57293

****** *************************** *************************

Human AACTTTCTGAGAATTGTTTCTCTAAAAAAAAAAAAAAAAAAAAAAAAAACTTGAGAGAGT 57383

Chimpanzee AACTTTCTGAGAATTGTTTCTCTAAAAAAAAAAAAAAAAA---------CTTGAGAGAGT 66260

Gorilla AACTTTCTGAGAATTGTTTCTCTAAAAAAAAAAAAAACANNNNNNNNNNCTTGAGAGAGT 57353

************************************* * ***********

Human CACGTGCAGCTATGGCCAGTGGTATGAATGTTTCATGCTCTCCAACTGCAGAATCTAGAT 57443

Chimpanzee CACGTGCAGCTATGGCCAGTGGTATGAATGTTTCATGCTCTCCAACTGCAGAATCTAGAT 66320

Gorilla CACGAGNANNNNNNNNNNNNNNNNTGAATGTTTCATGCTCTCCAACTGCAGAATCTAGAT 57413

**** * * ************************************

Human ACTTTCTGCTTCTATGTTAGTTATATAGAAAAACTGCAGCATGGTAGGACAATTGAATAC 57503

Chimpanzee ACTTTCTGCTTCTATGTTAGTTATATAGAAAAACTGCAGCATGGTAGGACAATTGAATAC 66380

Gorilla AGTTTCTGCTTCTATGTTAGTTATATAG-AACNCTGCAGCATGGTAGGACAATTGAATAC 57472

* ************************** ** ***************************

Human AGAGATCAAGATTCAGGATAGTAAATCTCTATGCCTTGTGAAGAGTTTTGCTTTTGCTAA 57563

Chimpanzee AGAGATCAAGATTCAGGATAGTAAATCTCTATGCCTTGTGAAGAGTTTTGCTTTTGCTAA 66440

Gorilla AGAGATCAAGATTCAGGATAGTAAATCTCTATGCCTTGTGCAGAGTTTTGCTTTTGCTAA 57532

**************************************** *******************

Human AGGGAAGAGTAAATCATATGGGTGTGTAAAACTCACCACTGGAATCACCTTTTTTTTTTT 57623

Chimpanzee AGGGAAGAGTAAATCATATGGGTGTGTAAAACTCACCACTGGAATCACCTTTTTTTTTTT 66500

Gorilla AGGGAAGAGTAAATCATATGGGTGTGTAAAACTCACCACTGGAATCACCTTTTTTTTTTT 57592

************************************************************

Human TTTTTTCAGGAGTTCAGAACTGATGAAAGGGTCAAAACATAGATGATGTTTTGATCTTCC 57683

Chimpanzee TT-CTTCAGGAGTTCAGAACTGATGAAAGGGTCAAAAAATAGATGATGTTTTGATTTTCC 66559

Gorilla TTTCTTCAGGAGTTCAGAACTGATGAAAGGGTCAAAAAATAGATGATGTTTTGATTTTCC 57652

** ********************************* ***************** ****

Human ACAGGATTACCCAACATTTCTTCTGACAGTATTTGTCAGCTATTTCTTCCTCGGGTGCCC 57743

Chimpanzee ACAGGATTACCCAACATTTCTTCTGACAGTATTTGTCAGCTATTTCTTCCTCGGGTGCCC 66619

Gorilla ACAGGATTACCCAACATTTCTTCTGACAGTATTTGTCAGCTATTTCTTTCTCAGGCGCCC 57712

************************************************ *** ** ****

Human AGAGAAAGTCTTGAGTTTAGCCAAAATTTCTCTATAATATCTTTGTTGTTGCCCCATGGG 57803

Chimpanzee AGAGAAAGTCTTGAGTTTAGCCAAAATTTCTCTATAATATCTTTGTTGTTGCCCCATGGG 66679

Gorilla AGAGAAAGTCTTGAATTTAGCCAAAATTTCTCTATAATATCTTTGTTGTTGCCCCATGGG 57772

************** *********************************************

Human TTTTAGGAAGCAGTATCCATCTTGTGCCAAGAAAGGCTAATCAAGGCAACTTCCTGAAGT 57863

Chimpanzee TTTTAGGAAGCAGTATCCATCTTGTGCCAAGAAAGGCTAATCAAGGCAACTTCCTGAAGT 66739

Gorilla TTTTAGGAAGCAGTATCCATCTTGTGCCAAGAAAGGCTAATCAAGGCAACTTCCTGAAGT 57832

************************************************************

Human ATAAGCTGAGATGAGGGGTGGAAGAGGATTAATGCAGGACTGTTACAGAAATAAAGAAAA 57923

Chimpanzee ATAAGCTGAGATGAGGGGTGGAAGAGGATTAATGCAGGACTGTTACAGAAATAAAGAAAA 66799

Gorilla ATAAGCTGAGATGNGNGGNGGAAGAGGATTAATGCAGGACTGTTACAGAAATAAAGAAAA 57892

************* * ** *****************************************

Human AATCTGCAGCAGAGCAACAACTCCAACCCTTGCTGCAATGTTTCATTCAGACACTGAAAT 57983

Chimpanzee AATCTGCAGCAGAGCAACAACTCCAACCCTTGCTGGAATGTTTCATTCAGACACTGAAAT 66859

Gorilla AATCTGCAGCAGAGCAACAACTCCAACCCTTGCTGGAATGTTTCGTTCAGACACTGAANN 57952

*********************************** ******** *************

Human TTCCAAGGTGCCCAGAAATCAACATTTAATATACTGTGGGATGCATAGAGGAAGCTGTCC 58043

Chimpanzee TTCCAAGGTGCCCAGAAATCAACATTTAATATACTGTGGGATGCATAGAGGAAGCTGTCC 66919

Gorilla NTCCAAGGTGCCCAGAAATCAACATTTAATATACTGTGGGATGCATAGAGGAAGCTGTCC 58012

***********************************************************

Human ATATGTTTCTTCTTTTTGAAGTTTTAGGCA-TTTTTTTTTTTCAGTTCAAAGGTTCTTTT 58102

Chimpanzee CTATGTTTCTTCTTTTTGAAGTTTTAGGCATTTTTTTTTTTTCAGTTCAAAGGTTCTTTT 66979

Gorilla ATATGTTTCTTTTTTTTGAAGTTTTAGGCA---TTTTTTTTTCAGTTCAAAGGTTCTTTT 58069

********** ****************** ***************************

Human AACCTTAATAAGTTCAATTAAACAAAAATACACTTAAGTACTACTAATAACATGACTTGG 58162

Chimpanzee AACCTTAATAAGTTCAATTAAACAAAAATACACATAAGTACTACTAATAACATGACTTGG 67039

Gorilla AACCGTAATAAGTTCAATTAAACAAAAATACACATAAGTACTACTAATAACATGAGTTGG 58129

**** **************************** ********************* ****

Human CAAATTGTGAAATGTAAATTTAAAATGTAACAACAAACACACGTGTATGTTAATTTGATA 58222

Chimpanzee CAAATTGTGAAACGTAAATTTAAAATGTAACAACAAACACACGTGTATGTTAATTTGATA 67099

Gorilla CAAATTGTGAAATGTAAATTTAAAATGTAACAACAAACACACGTGTATGTTAATTTGATA 58189

************ ***********************************************

Human TCCATTTATAAATTTACATACAAGCTTGTATATACATTAATATACATAAAGTTAAAATAT 58282

Chimpanzee TCCATTTATAAATTTACATACAAGCTTGTATATACATTAATATACATAAAGTTAAAATAT 67159

Gorilla TCCATTTATAAATTTACATATAAGCTTGTATATACATTAATATACATAAAGTTAAAATAT 58249

******************** ***************************************

Human ATTATGTATATGTAAATATATATACTTTTTAAATTTTATTATTATTATACTTTAAGTTTT 58342

Chimpanzee ATTATGTATATGTAAATATATATACTTTTTAAATTTTATTATTATTATACTTTAAGTTTT 67219

Gorilla ATTATGTATATGTAAATNNNNNNNNNNNNNNNNNNNNNNNNNNNNNNNNNNNNNNNNNNN 58309

*****************

Human AGGGCACATGTGCACAATGTGCAGGTTTGTTACATATGTATACATGTGCCATGCTGGTGT 58402

Chimpanzee AGGGCACATGTGCACAATGTGCAGGTTTGTTACATATGTATACATGTGCCATGCTGGCAT 67279

Gorilla NNNNNNNNNNNNNNNNNNNNNNNNNNNNNNNNNNNNNNNNNNNNNNNNNNNNNNNNNNNN 58369

Human GCGGCACCCATTAACTTGTCATTTAGCATTAGGTATCTCCTATTGCTATCCCTCCCCCCT 58462

Chimpanzee GCGGCACCCATTAACTTGTCATTTAGCATTCGGTATCTCCTATTGCTATCCCTCCCCCCT 67339

Gorilla NNNNNNNNNNNNNNNNNNNNNNNNNNNNNNNNNNNNNNNNNNNNNNNNNNNNNNNNNNNN 58429

Human CCCCCCACCCCACAACAGTCCCCGAGTGTGATGTTCCCCTTCCTATGTCCATGTGTTCTC 58522

Chimpanzee CCCCGCACCCCACAACAGTCCCCGAGTGTGATGTTCCCCTTCCTATGTCCATGTGTTCTC 67399

Gorilla CCCCCCACCCCACAACAGTCCCCGAGTGTGATGTTCCCCTTCCTATGTCCATGTGNNNNN 58489

**** **************************************************

Human ATTGTTCAATTCACGCCTATGAGTGAGAACATGTGGTGTTTGGTTTTTTGTCCTTGCAAT 58582

Chimpanzee ATTGTTCAATTCACGCCTATGAGTGAGAACATGTGGTGTTTGGTTTTTTGTCCTTGCAAT 67459

Gorilla NNNNNNNNNNNNNNNNNNNNNNNNNNNNNNNNNNNNNNNNNNNNNNNNNNNNNNNNNNNN 58549

Human AGTTTGCTAAGAGTGATGATTTCCAGTTTCATCCATGTCCCTGCAAAGGACATGAACTCA 58642

Chimpanzee AGTTTGCTAAGAATGATGATTTCCAGTTTCATCCATGTCCCTGCAAAGGACATGAACTCA 67519

Gorilla NNNNNNNNNNNNNNNNNNNNNNNNNNNNNNNNNNNNNNNNNNNNNNNNNNNNNNNNNNNN 58609

Human TCATTTTTTATGGCTGCATACTATTCCATGGTGTATATGTGCCACATTTTCTTAATCCGT 58702

Chimpanzee TCATTTTTTGTGGCTGCATACTATTCCATGGTGTATATGTGCCACAGTTTCTTAATCCAT 67579

Gorilla NNNNNNNNNNNNNNNNNNNNNNNNNNNNNNNNNNNNNTGTGCCNCATTTNCTTAATCCGT 58669

****** ** ** ******** *

Human TCTATCATTGTTGGACATTTGGGTTGGTTCCAAGTCTTTGCTATTGTGGATAGCGTCGCA 58762

Chimpanzee TCTATCATTGTTGGACATTTGGGTTGGTTCCAAGTCTTTGCTATTGTGGATAGTGTCGCA 67639

Gorilla TCTATCATTGTTGNNNNNNNNNNNNNNNNNNNNNNNNNNNNNNNNNNNNNNNNNNNNNNN 58729

*************

Human ATAAACATACGTGTGCATGTGTCTTTATAGCAGCATGATTTATAGTCCTTTGGATATATA 58822

Chimpanzee ATAAACATACGTGTGCATGTGTCTTTATAGCAGCATGATTTATAGTCCTTTGGGTATATA 67699

Gorilla NNNNNNNNNNNNNNNNNNNNNNNNNNNNNNNNNNNNNNNNNNNNNNNNNNNNNNNNNNNN 58789

Human CCCAGTAATGGGATGTCTGGGTCAAATGGTATTTCTAGTTCTAGATCCCTGAGGAATCGC 58882

Chimpanzee CCCAGTAATGGGATGTCTGGGTCAAATGGTATTTCTAGTTCTAGATCCCTGAGGAATCGC 67759

Gorilla NNNNNNNNNNNNNNNNNNNNNNNNNNNNNNNNNNNNNNNNNNNNNNNNNNNNNNNNNNNN 58849

Human CACACTGATTTCCACAATGGTTGAACTAGTTTACAGTCCAACCAGCAGTGTAAAAGTGTT 58942

Chimpanzee CACACTGATTTCCACAATGGTTGAACTAGTGTACAGTCCCACCAGCAGTGTAAAAGTGTT 67819

Gorilla NNNNNNNNNNNNNNNNNNNNNNNNNNNNNNNNNNNNNNNNNNNNNNNNNNNNNNNNNNNN 58909

Human CCTATTTCTCCACATCCTCTCCAGCACCTGTTGTTTCCTGACTTTTTAATGATCGCCATT 59002

Chimpanzee CCTATTTCTCCACATCCTCTCCAGCACCTGTTGTTTCCTGACTTTTTAATGATCGCCATT 67879

Gorilla NNNNNNNNNNNNNNNNNNNNNNNNNNNNNNNNNNNNNNNNNNNNNNNNNNNNNNNNNNNN 58969

Human CTAACTGGTGTGAGATGGTATCTCATTGTGGTTTCAATTTGCATTTCTCTGATGGCCAGT 59062

Chimpanzee CTAACTGGTGTGAGATGGTATCTCATTGTGGTTTCAATTTGCATTTCTCTGATGGCCAGT 67939

Gorilla NNNNNNNNNNNNNNNNNNNNNNNNNNNNNNNNNNNNNNNNNNNNNNNNNNNNNNNNNNNN 59029

Human GATGATGAGCATTTTTTCATGTGTTTTTTGGCTGCATAAATGTCTTCTTCTGAGAAGTGT 59122

Chimpanzee GATGATGAGCATTTTTTCATGTGTTTTTTGGCTGCATAAATGTCTTCTTCTGAGAAGTGT 67999

Gorilla NNNNNNNNNNNNNNNNNNNNNNNNNNNNNNNNNNNNNNNNNNNNNNNNNNNNNNNNNNNN 59089

Human CTGTTCATATCCTTGGCCCACTTTTTGATGGGATTGTTTGTTTTTTTCTTGTAAATTTGT 59182

Chimpanzee CTGTTCATATCCTTGGCCCACTTTTTGATGGGATTGTTTGTTTTTTTCTTGTAAATTTGT 68059

Gorilla NNNNNNNNNNNNNNNNNNNNNNNNNNNNNNNNNNNNNNNNNNNNNNNNNNNNNNNNNNNN 59149

Human TTGAGTTCATTGTAGATTCTGGATGTTAGCCCTTTGTCAGATGAGTAGGTTGCAAAAATT 59242

Chimpanzee TTGAGTTCATTGTAGATTCTGGATGTTAGCCCTTTGTCAGATGAGTAAGTTGCAAAAATT 68119

Gorilla NNNNNNNNNNNNNNNNNNNNNNNNNNNNNNNNNNNNNNNNNNNNNNNNNNNNNNNNNNNN 59209

Human TTCTCCCATTTTGTAGGTTGCCTGTTCACTCTGATGGTAGTTTCTTTTGCTGTGCAGAAG 59302

Chimpanzee TTCTCCCAATTTGTAGGTTGCCTGTTCACTCTGATGGTAGTTTCTTTTGCTGTGCAGAAG 68179

Gorilla NNNNNNNNNNNNNNNNNNNNNNNNNNNNNNNNNNNNNNNNNNNNNNNNNNNNNNNNNNNN 59269

Human CTCTTTAGTTTAATTAGATCCCATTTGCCAATTTTGGCTTTTGTTGCCATTGCTTTTGGT 59362

Chimpanzee CTCTTTAGTTTAATTAGATCCCATTTGCCAATTTTGGCTTTTGTTGCCATTGCTTTTGGT 68239

Gorilla NNNNNNNNNNNNNNNNNNNNNNNNNNNNNNNNNNNNNNNNNNNNNNNNNNNNNNNNNNNN 59329

Human GTTTTAGGCACGAAGTCCTTGCCCATGCCTATGTCCTGAATCATATTGCCTAGGTTTTCT 59422

Chimpanzee GTTTTAGGCATGAAGTCCTTGCCCATGCCTATGTCCTGAATCATATTGCCTAGGTTTTCT 68299

Gorilla NNNNNNNNNNNNNNNNNNNNNNNNNNNNNNNNNNNNNNNNNNNNNNNNNNNNNNNNNNNN 59389

Human TCTAGGGTTTTTATGGTTTTAGGTCTAACATGGAAGTCTTTAATCCATCTTGAATTAATT 59482

Chimpanzee TCTAGGGTTTTTATGGTTTTAGGTCTAACATGGAAGTCTTTAATCCATCTTGAATTAATT 68359

Gorilla NNNNNNNNNNNNNNNNNNNNNNNNNNNNNNNNNNNNNNNNNNNNNNNNNNNNNNNNNNNN 59449

Human TTTGTATAAGGTGTAAGAAAGGGATCCAGTTTCGGCTTTCTACATATGGCTAGCCTGTTT 59542

Chimpanzee TTTGTATAAGGTGTAAGAAAGGGATCCAGTTTCGGCTTTCTACATATGGCTAGCCTGTTT 68419

Gorilla NNNNNNNNNNNNNNNNNNNNNNNNNNNNNNNNNNNNNNNCTACGTATGGCTGGCCTGTTT 59509

**** ******* ********

Human CCCCAGCACCATTTATCAAATAGGGAATCCTTTCCCCATTGCTTGTTTTTCTCAGGTTTG 59602

Chimpanzee CCCCAGCACCGTTTATCAAATAGGGAATCCTTTCCCCATTGCTTGTTTTTGTCAGGTTTG 68479

Gorilla CCCCAGCACCATTTATCAAATAGGGAATCNNNNNNNNNNNNNNNNNNNNNNNNNNNNNNN 59569

********** ******************

Human CCAAAGATCAGATGGTTGTAGATATGCAGCATTATTTCTGAGGGCTCTGTTCTGTTCCAT 59662

Chimpanzee CCAAAGATCAGATGGTTGTAGATATGCAGCATTATTTCTGAGGGCTCTGTTCTGTTCCAT 68539

Gorilla NNNNNNNNNNNNNNNNNNTAGATNNNNNNNNNNNNNNNNNNNNNNNNNNNNNNNNNNNNN 59629

*****

Human TGATCTATGTCTCTGTTTTGGTACCAGTACCATGCTGTTTTGGTTACCATAGCCTTGTAG 59722

Chimpanzee TGATCTATGTCTCTGTTTTGGTACCAGTACCATGCTGTTTTGGTTACCATAGTCTTGTAG 68599

Gorilla NNNNNNNNNNCTNNNNNNNNNNNNNNNNNNNNNNNNNNNNNNNNNNNNNNNNNNNNNNNN 59689

**

Human TATAGTTTGAAGTCAGGTAGCGTGATGCCTCTGGCTTTGTTCTTTTGGTTTAGGATTGAC 59782

Chimpanzee TATAGTTTGAAGTCAGGTAGCATGATGCCTCTGGCTTTGTTCTTTTGGTTTACGATTGAC 68659

Gorilla NNNNNNNNNNNNNNNNNNNNNNNNNNNNNNNNNNNNNNNNNNNNNNNNNNNNNNNTTGAC 59749

*****

Human CTTGCAATGCGGGCTCTTTTTTGGTTCCATATGAACTTTAAAGTAGTTTTTTCCAATTCT 59842

Chimpanzee CTTGCAATGCGGGCTCTTTTTTGGTTCCATATGAACTTTAAAGTAGTTTTTTCCAATTCT 68719

Gorilla CTTGCAATGCGGGCTCTTTTTTGGTTCCATANNNNNNNNNNNNNNNNNNNNNNNNNNNNN 59809

*******************************

Human GTGAAGAAAGTCATTGGTAGCTTCATGGGGATGGCATTTAATCTATAAATTACCTTGGGC 59902

Chimpanzee GTGAAGAAAGTCATTGGTAGCTTCATGGGGATGGCATTGAATCTATAAATTACCTTGGGC 68779

Gorilla NNNNNNNNNNNNNNNNNNNNNNNNNNNNNNNNNNNNNNNNNNNNNNNNNNNNNNNNNNNN 59869

Human AGTATGGCCATTTTCACAATATTGATTCTTACTACCCATGAGCATGGAATGTTCTTCCAT 59962

Chimpanzee AGTATGGCCATTTTCACGATATTGATTCTTACTACCCATGAGCATGGAATGTTCTTCCAT 68839

Gorilla NNNNNNNNNNNNNNNNNNNNNNNNNNNNNNNNNNNNNNNNNNNNNNNNNNNNNNNNNNNN 59929

Human TTCTTTGTATCCTCTTTTATTTCATTGAGCAGTGGTTTGTAGTTCTCCTTGAAGAGGTCC 60022

Chimpanzee TTCTTTGTATCCTCTTTTATTTCATTGAGCAGTGGTTTGTAGTTCTCCTTGAAGAGGTCC 68899

Gorilla NNNNNNNNNNNNNNNNNNNNNNNNNNNNNNNNNNNNNNNNNNNNNNNNNNNNNNNNNNNN 59989

Human TTCATGTCCCTTGTAAGTTGGATTCTTAGGTATGTTATTCTCTTTGAAGCAATTGTGAAT 60082

Chimpanzee TTCATGTCCCTTGTAAGTTGGATTCTTAGGTATTTTATTCTCTTTGAAGCAATTTTGAAT 68959

Gorilla NNNNNNNNNNNNNNNNNNNNNNNNNNNNNNNNNNNNNNNNNNNNNNNNNNNNNNNNNNNN 60049

Human GGGAGTTCACTCATGATTTGGCTCTCTGTTTGTCTGTTGTTGTGTATAAGAATGCTTGTG 60142

Chimpanzee GGGAGTTCACTCATGATTTGGCTCTCTGTTTGTCTGTTGTTGTGTATAAGAATGCTTGTG 69019

Gorilla NNNNNNNNNNNNNNNNNNNNNNNNNNNNNNNNNNNNNNNNNNNNNNNNNNNNNNNNNNNN 60109

Human ATTTTTGCACATTGATTTTGTATCCTGAGACTTTGCTGAAATTGCTTATCAGCTTAAGGA 60202

Chimpanzee ATTTTTGCACATTGATTTTGTATCCTGAGACTTTGCTGAAATTGCTTATCAGCTTAAGGA 69079

Gorilla NNNNNNNNNNNNNNNNNNNNNNNNNNNNNNNNNNNNNNNNNNNNNNNNNNNNNNNNNNNN 60169

Human GATTTTGGGCTGAGACAATGGGGTTTTCTAGATGTACAGTCATGTCATCTGCAAACAGGG 60262

Chimpanzee GATTTTGGGCTGAGACAATGGGGTTTTCTAGATATACAGTCATGTCATCTGCAAACAGGA 69139

Gorilla NNNNNNNNNNNNNNNNNNNNNNNNNNNNNNNNNNNNNNNNNNNNNNNNNNNNNNNNNNNN 60229

Human ACAATTTGACTTCCTCTTTTCCTAATTGAATACTTTTTTTCCTTCTCCTGCCTGATTGCC 60322

Chimpanzee ACAATTTGACTTCCTCTTTTCCTAATTGAATACTTTTTTTCCTTCTCCTGCCTGATTGCC 69199

Gorilla NNNNNNNNNNNNNNNNNNNNNNNNNNNNNNNNNNNNNNNNNNNNNNNNNNNNNNNNNNNN 60289

Human CTGGCCAGAACTTCCAACACTATGTGGAATAGGAGTGGTGTGAGAGGGCATCCCTGTCTT 60382

Chimpanzee CTGGCCAGAACTTCCAACACTATGTGGAATAGGAGTGGTGTGAGAGGGCATCCCTGTCTT 69259

Gorilla NNNNNNNNNNNNNNNNNNNNNNNNNNNNNNNNNNNNNNNNNNNNNNNNNNNNNNNNNNNN 60349

Human GTGCCCATTTTCAAAGGGAATGCTTCCAGTTTTTGCCCATTCAGTATGATATTGGCTGTG 60442

Chimpanzee GTGCCCATTTTCAAAGGGAATGCTTCCAGTTTTTGCCCATTCAGTATGATATTGGCTGTG 69319

Gorilla NNNNNNNNNNNNNNNNNNNNNNNNNNNNNNNNNNNNNNNNNNNNNNNNNNNNNNNNNNNN 60409

Human GGTTTGTCATAGATATCTCTTATTATTTGGAGATACGTCCCATCAATACAGAATTTATTG 60502

Chimpanzee GGTTTGTCATAGATATCTCTTATTGTTTGGAGATACGTCCCATCAATACAGAATTTATTG 69379

Gorilla NNNNNNNNNNNNNNNNNNNNNNNNNNNNNNNNNNNNNNNNNNNNNNNNNNNNNNNNNNNN 60469

Human AGAGATTTTAGCATGAAGGGTTGTTGAATTTTGTCAAAGGCCTTTTCTGCATCTATTGAG 60562

Chimpanzee AGAGATTTTAGCATGAAGGGTTGTTGAATTTTGTCAAAGGCCTTTTCTGCATCTATTGAC 69439

Gorilla NNNNNNNNNNNNNNNNNNNNNNNNNNNNNNNNNNNNNNNNNNNNNNNNNNNNNNNNNNNN 60529

Human ATAATCAGGTGGTTTTTGTCTTTGGTTCTGTTTATAAGCCGGATTACATTTTTTGATTTG 60622

Chimpanzee ATAATCATGTGGTTTCTGTCTTTGGTTCTGTTTATAAGCTGGATTACATTTTTTGATTTG 69499

Gorilla NNNNNNNNNNNNNNNNNNNNNNNNNNNNNNNNNNNNNNNNNNNNNNNNNNNNNNNNNNNN 60589

Human GGTATGTTGTACCAGCCTTGCATCCCAGGGAGGAAGCCCACTTGATCATGGTGGATAAGC 60682

Chimpanzee GGTATGTTGTACCAGCCTTGCATCCCAGGGATGAAGCCCACTTGATCATGGTGGATAAGC 69559

Gorilla NNNNNNNNNNNNNNNNNNNNNNNNNNNNNNNNNNNNNNNNNNNNNNNNNNNNNNNNNNNN 60649

Human TTTTTGATGTGCTGCTGGATTCGGTTTGCCAGTATTTTACTGAGGATTTTTACATCGATA 60742

Chimpanzee TTTTTGATGTGCTGCTGGATTTGGTTTGCCAGTATTTTACTGAGGATTTTTACATCGATA 69619

Gorilla NNNNNNNNNNNNNNNNNNNNNNNNNNNNNNNNNNNNNNNNNNNNNNNNNNNNNNNNNNNN 60709

Human TTCATCAGGGATATTGGTCTAAAATTCTCTTTTTTGGTTGTGTCTCTGCCAGGCTTTGGT 60802

Chimpanzee TTCATCAGGGATATTGGTCTAAAATTCTCTTTTTTGGTTGTGTCTCTGCCAGGCTTTGGT 69679

Gorilla NNNNNNNNNNNNNNNNNNNNNNNNNNNNNNNNNNNNNNNNNNNNTCTGCCAGGCTTTGGT 60769

****************

Human ATCAGGATGTTGTTGCCCTCATAAAATGAGTTAGGGAGGATTCTCTCTTTTTCTATTGAT 60862

Chimpanzee ATCAGGATGTTGTTGCCCTCATAAAATGAGTTAGGGAGGATTCTCTCTTTTTCTATTGAT 69739

Gorilla ATCAGGATGTTGTTGCCCTCNNNNNNNNNNNNNNNNNNNNNNNNNNNNNNNNNNNNNNNN 60829

********************

Human TGGATTGGTTTCAGAAGGAATGGTACAAGCTCCTCCTTGTACCTCTGGTAGAATTGGGCT 60922

Chimpanzee TGGATTGGTTTCAGAAGGAATGGTACAAGCTCCTCCTTGTACCTCTGGTAGAATTGGGCT 69799

Gorilla NNNNNNNNNNNNNGAAGGAATGGTNNNAGNTCCTCCTTGTACCTCTGGTAGAATTGGGCT 60889

*********** ** ******************************

Human GTGAATCCATCTGGTCCTTGACTTTTTTTGGTTGGTAAGCTATTGATTATTGCCACAATT 60982

Chimpanzee GTGAATCCATCTGGTCCTTGACTTTTTTTGGTTGGTAAGCTATTGATTATTGCCACAATT 69859

Gorilla GTGAANNNNNNNNNNNNNNNNNNNNNNNNNNNNNNNNNNNNNNNNNNNNNNNNNNNNNNN 60949

*****

Human TCAGATCCTGTTATTGGTCTATTCAGAGATTCAACTTCTTCCTGGTTTACCCTTGGGAGG 61042

Chimpanzee TCAGATCCTGTTATTGGTCTATTCAGAGATTCAACTTCTTCCTGGTTTACCCTTGGGAGG 69919

Gorilla NNNNNNNNNNNNNNNNNNNNNNNNNNNNNNNNNNNNNCTTNNNNNTNNNCCCTTGGGAGG 61009

*** * ***********

Human GTGTAGGTGTCGAGGAATTTTTCCATTTCTTCTAGATTTTCTAGTTTATTTGCATAGAGA 61102

Chimpanzee GTGTATGTGTCGAGGAATTTTTCCATTTCTTCTAGATTTTCTAGTTTATTTGCATAGAGA 69979

Gorilla GTGTATGTGTCGAGGAAATTTTCCNNNNNNNNNNNNNNNNNNNNNNNNNNNNNNNNNNNN 61069

***** *********** ******

Human TGTTTGTAGTATTCTCTGATAGTAGATTGTATTTCTGTGGGATCGGTGGTGATATCCCTT 61162

Chimpanzee TGTTTGTAGTATTCTCTGATAGTAGATTGTATTTCTGTGGGATCGGTGGTGATATCCCTT 70039

Gorilla NNNNNNNNNNNNNNNNNNNNNNNNNNNNNNNNNNNNNNNNNNNNNNNNNNNNNNNNNNNN 61129

Human TTATTGTTTTTTATTACATCTATTTGATTCTTCTCTGTGTTCTTCTTTATTAGTCTTGCT 61222

Chimpanzee TTATTGTTTTTTATTGCATCTATTCGATTCTTCTCTGTGTTCTTCTTTATTAGTCTTGCT 70099

Gorilla NNNNNNNNNNNNNNNNNNNNNNNNNNNNNNNNNNNNNNNNNNNNNNNNNNNNNNNNNNNN 61189

Human AGCGGTCTATCAATTTTGTTGATCTTTTCAAAAAACCAGCTCCTGGATTCATTAATTTTT 61282

Chimpanzee AGCGGTCTATCAATTTTGTTGATCTTTTCAAAAAACCAGCTCCTGGATTCATTAATTTTT 70159

Gorilla NNNNNNNNNNNNNNNNNNNNNNNNNNNNNNNNNNNNNNNNNNNNNNNNNNNNNNNNNNNN 61249

Human TGAAGAGTTTTTTTGTGTCTCTATTTCCTTCAGTTCTGCTCTGATGTTAGTTATTTCTTG 61342

Chimpanzee TGAAGAGTTTTTTTGTGTCTCTATTTCCTTCAGTTCTGCTCTGATGTTAGTTATTTCTTG 70219

Gorilla NNNNNNNNNNNNNNNNNNNNNNNNNNNNNNNNNNNNNNNNNNNNNNNNNNNNNNNNNNNN 61309

Human CCTTCTGCTAGCTTTTGAATGTGTTTGCTCTTGCTTTTCTAGTTCTTTTAATTATGATGT 61402

Chimpanzee CCTTCTGCTAGCTTTTGAATGTGTTTGCTCTTGCTTTTCTAGTTCTTTTAATTGTGATGT 70279

Gorilla NNNNNNNNNNNNNNNNNNNNNNNNNNNNNNNNNNNNNNNNNNNNNNNNNNNNNNNNNNNN 61369

Human TAGGGTGTCAATTTTGGATCTTTCCTGCTTTGTCTTGTGGGCATTTAGTGCTATAAATTT 61462

Chimpanzee TAGGGTGTCAATTTTGGATCTTTCCTGCTTTGTCTTGTGGGCATTTAGTGCTATAAATTT 70339

Gorilla NNNNNNNNNNNNNNNNNNNNNNNNNNNNNNNNNNNNNNNNNNNNNNNNNNNNNNNNNNNN 61429

Human CCCTCTACAGACTGCTTTGCATGTGCCCCAGAGATTCTGGTATGTTGTGTCTTTATTCTC 61522

Chimpanzee CCCTCTACAGATTGCTTTGAATGTGTCCCAGCGATTCTGGTATGTTGTGTCTTTATTCTC 70399

Gorilla NNNNNNNNNNNNNNNNNNNNNNNNNNNNNNNNNNNNNNNNNNNNNNNNNNCTTTATTCTC 61489

**********

Human ATTGGTTTCAAAGAACATCTTTATTTCTGCCTTCATTTCATTATGTACCCAGTAGTCATT 61582

Chimpanzee ATTGGTTTCAAAGAACATCTTTATTTCTGCCTTCATTTCATTATGTACCCAGTAGTCATT 70459

Gorilla ATTGGTTTCAAAGAACATCTTTATTNNNNNNNNNNNNNNNNNNNNNNNNNNNNNNNNNNN 61549

*************************

Human GAGGAGCAGGTTGTTCAGTTTCCATGTAGTTGAGCGGTTTTGAGTGAGTTTCTTAATCCT 61642

Chimpanzee CAGGAGCAGGTTGTTCAGTTTCCATGTAGTTGAGCGGTTTTGAGTGAGTTTCTTAATCCT 70519

Gorilla NNNNNNNNNNNNNNNNNNNNNNNNNNNNNNNNNNNNNNNNNNNNNNNNNNNNNNNNNCCT 61609

***

Human GAGTTCTAGTTTGATTGCACTGTGGTTGGAGAGACAGTTTGCTATAATTTCTGTGCTTTT 61702

Chimpanzee GAGTTCTAGTTTGATTGCACTGTGGTTGGAGAGACAGTTTGCTACAATTTCTGTGCTTTT 70579

Gorilla GAGTTCTAGTTTGATTGCACTGTGGTTGGAGAGACAGTTTGCTATAATTTCTGTGCTTTT 61669

******************************************** ***************

Human ACATTTGCTGAGGAGTGCTTTACTTCCAACTATGTGGTCAATTTTGGAATAGGTGAGATG 61762

Chimpanzee ACATTTGCTGAGGAGTGCTTTACTTCCAACTATGTGGTCAATTTTGGAATAGGTGAGATG 70639

Gorilla ACATTTGCTGAGGAGTGATTTNCTTNNNNNNNNNNNNNNNNNNNNNNNNNNNNNNNNNNN 61729

***************** *** ***

Human TGGTGTTGAAAAAAAATGTATATTCTGTTGTTTTGGGGTGGAGAGTTCTGTAGATGTCTA 61822

Chimpanzee TGGTGTTGAAAAAAA-TGTATATTCTGTTGTTTTGGGGTGGAGAGTTCTGTAGATGTCTA 70698

Gorilla NNNNNNNNNNNNNNNNNNNNNNNNNNNNNNNNNNNNNNNNNNNNNNNNNNNNNNNNNNNN 61789

Human TTAGGTCTGTTTGGTGCAGAGCTGAGTTCAATTCCTGGGTATCCTTTTTAACTTTCTGTC 61882

Chimpanzee TTAGGTCTGTTTGGTGCAGAGCTGAGTTCAATTCCTGGGTATGCTTTTTAACTTTCTGTC 70758

Gorilla NNNNNNNNNNNNNNNNNNNNNNNNNNNNNNNNNNNNNNNNNNNNNNNNNNNNNNNNNNNN 61849

Human TCATTGATCTGTCTAATGTTGACAGTGGGGTGTTAAAGTCTCCCATTATTATTGCATGGG 61942

Chimpanzee TCATTGATCTGTCTAATGTTGACAGTGGGGTGTTAAAGTCTCCAATTATTATTGCATGGG 70818

Gorilla NNNNNNNNNNNNNNNNNNNNNNNNNNNNNNNNNNNNNNNNNNNNNNNNNNNNNNNNNNNN 61909

Human AGTCTAAGTCTCTTTGTGGGTCACTGAGGGCTTGCTTTATGAATCTGGGTGCTCCTGTAT 62002

Chimpanzee AGTCTAAGTCTGTTTGTGGGTCACTGAGGGCTTGCTTTATGAATCTGGGTACTCCTGTAT 70878

Gorilla NNNNNNNNNNNNNNNNNNNNNNNNNNNNNNNNNNNNNNNNNNNNNNNNNNNNNNNNNNNN 61969

Human TGGGTGCATATATATATTTAGGATAGTTAGCTCTTCTTGTTGAATTGATCCCTTTATCAT 62062

Chimpanzee TGGGTGCATATATAT--TTAGGATAGTTAGCTCTTCTTGTTGAATTGATCCCTTTACCAT 70936

Gorilla NNNNNNNNNNNNNNNNNNNNNNNNNNNNNNNNNNNNNNNNNNNNNNNNNNNNNNNNNNNN 62029

Human TATGTAATGGCCTTCTTTGTCTCTTTTGATCTTTGTTGGTTTAAAATCTGTTTTATCAGA 62122

Chimpanzee TATGTTATGGCTTTCTTTGTCTCTTTAGATCTTTGTTGGTTTAAAGTCTGTTTTATCAGA 70996

Gorilla NNNNNNNNNNNNNNNNNNNNNNNNNNNNNNNNNNNNNNNNNNNNNNNNNNNNNNNNNNNN 62089

Human GACTAGGATTGCAACCCCTGCCTTTTTTTGTTTTCCATTTGCTTGGTAGATCTTCCTCCG 62182

Chimpanzee GACTAAGATTGCAACCCCTATCTTTTTTTGTTTTCCATTTGCTTGGTAGATCTTCCTCCA 71056

Gorilla NNNNNNNNNNNNNNNNNNNNNNNNNNNNNNNNNNNNNNNNNNNNNNNNNNNCTTCCTCCG 62149

********

Human TCCCTTTATTTTGAGCTTATGTGTGTCTCTGCATGTGAGATGGGTTTTCTGAATAAAGCA 62242

Chimpanzee TCCCTTTATTTTGAGCTTATGTGTGTCTCTGCATGTGAGATGGGTTTCCTGAATAAAGCA 71116

Gorilla TCCCTTTATTTTGAGCTTATGTGTGTCTCTNNNNNNNNNNNNNNNNNNNNNNNNNNNNNN 62209

******************************

Human CACTGATGGGTCTTGTCTCTTTATCCAATTTGCCAGTCTGTGTCTTTTAATTGGAGCATT 62302

Chimpanzee CACTGATGGGTCTTGTCTCTTTATCCAATTTGCCAGTCTGTGTCTTTTAATTGGAGCATT 71176

Gorilla NNNNNNNNNNNNNNNNNNNNNNNNNNNNNNNNNNNNNNNNNNNCTTTTAATTGGAGCATT 62269

*****************

Human TAGCCCATTTACATTTAAAGTTAATATGGTTATGTGTGAATTTGATCCTGTCATTATGAT 62362

Chimpanzee TAGCCCATTTACATTTAAAGTTAATATGGTTATGTGTGAATTTGATCCTGTCATTATGAT 71236

Gorilla TAGCCCATTTACATTTAAAGTTAATGTGGTNNNNNGTNNNNNNNNNNNNNNNNNNNNNNN 62329

************************* **** **

Human GTTAGCTGGTTATTTTGCTCATTAGTTGATGCAGTTTCTTCCTGGCCTCGATGGTCTTTA 62422

Chimpanzee GTTAGCTGGTTATTTTGCTCATTAGTTGATGCAGTTTCTTCCTGGCCTCGATGGTCTTTA 71296

Gorilla NNNNNNNNNNNNNNNNNNNNNNNNNNNNNNNNNNNNNNNNNNNNNNNNNNNNNNNNNNTA 62389

**

Human CAATTTGGCATGATTTTGCAGTGGCTGTTACCGGTTGTTCCTTTCCATGTTTAGCGCTTC 62482

Chimpanzee CAATTTGGCATGATTTTGCAGTGGCTGTTACTGGTTGTTCCTTTCCATGTTTAGTGCTTC 71356

Gorilla CAATTTGGNNNNNNNNNNNNNNNNNNNNNNNNNNNNNNNNNNNNNNATGTTTAGTGCTTC 62449

******** ******** *****

Human CCTCAGGAGGTCTTTTAAGGCAAGCCTGGTGGTGACAAAATCTCTCAGCATTTGCTTGTC 62542

Chimpanzee CCTCAGGAGGTCTTTTAAGGCAAGCCTGGTGGTGACAAAATCTCTCAGCATTTGCTTGTC 71416

Gorilla CCTCAGGAGGTCTTTTAAGGCAAGCCTGGTGGTGACNNNNNNNNNNNNNNNNNNNNNNNN 62509

************************************

Human TGTAAAGTATTTTATTTCTCCTTCACTTATGAAGCCTAGTTTGGCTGGATATGAAATTCT 62602

Chimpanzee TGTAAAGTATTTTATTTCTCCTTCACTTATGAAGCCTAGTTTGGCTGGATATGAAATTCT 71476

Gorilla NNNNNNNNNNNNNNNNNNNNNNNNNNNNNNNAAGCCTAGTTTGGCTGGATATGAAATTCT 62569

*****************************

Human GGGTTTAAGAATGTTCAATATTGGCCCCCACTCTCTTCTGGCTTGTAGAGTTTGGGCTGA 62662

Chimpanzee GGGTTTAAGAATGCTGAATATTGGCCCCCACTCTCTTCTGGCTTGTAGAGTTTGGGGTGA 71536

Gorilla GGGTTTAAGAATGTTGAANNNNNNNNNNNNNNNNNNNNNNNNNNNNNNNNNNNNNNNNNN 62629

************* * **

Human GAGATCCGCTGTTAGTTTGATGGGCTTCCATTTGTGGGTAACCCGACCTTTCTCTCTGGC 62722

Chimpanzee GAGATCCGCTGTTAGTTTGATGGGCTTCCATTTGTGGGTAACCCGACCTTTCTCTCTGGC 71596

Gorilla NNNNNNNNNNNNNNNNNNNNNNNNNNNNNNNNNNNNNNNNNNNNNNNNNNNNNNNNNNNN 62689

Human TGCCCTGAGCAATTTTTCCTTCATTTCACCTTTGATGAATCTGACAATTATGTGTCTTGG 62782

Chimpanzee TGCCCTGAACAATTTTTCCTTCATTTCGCCTTTGATGAATCTGACAATTATGTGTCTTGG 71656

Gorilla NNNNNNNNNNNNNNNNNNNNNNNNNNNNNNNNNNNNNNNNNNNNNNNNNNNNNNNCTTGG 62749

*****

Human AGTTGCTCTTCTCTAGGAGTATCTTTGTGGAGTTCTCTGTATTTCCTGAATCTGAATGTT 62842

Chimpanzee AGTTGCTCTTCTCTAGGAGTATCTTTGTGGAGTTCTCTGTATTTCCTGAATCTGAATGTT 71716

Gorilla AGTTGCTCTTCTCTAGGAGTATCTTTGTGGAGTTCTCTGTATTTCCTGANNNNGAANNNN 62809

************************************************* ***

Human GGCCTGCCTTGCTAGATTGGGTAAGTTCTCCTGGATAATATCTTGCAGAGTGTTTTCCAA 62902

Chimpanzee GGCCTGCCTTGCTAGATTGGGTAAGTTCTCCTGGATAATATCTTGCAGAGTGTTTTCCAA 71776

Gorilla GGCCTGCCTTGCTAGATTGNNNNNNNNNNNNNNNNNNNNATCTNNNNGAGTGTTNNNNNN 62869

******************* **** *******

Human CTTGGTTCCATTCCCCCCCGTCACTTTCAGGTATACCAATCAGATGTAGATTTGGTCTTT 62962

Chimpanzee CTTGGTTCCATTCCCCCCCGTCACTTTCAGGTATACCAATCAGATGTAGATTTGGTCTTT 71836

Gorilla NNNGGTTCCATTCCCNNNNNNNNNNNNNNNNNNNNNNNNNNNNNNNNNNNNNNNNNNNNN 62929

************

Human TCACATAGTCCCATATTTCTTGAAGGCTTTGTTCGTTTCTTTGTATTCTTTTTTCTCTAA 63022

Chimpanzee TCACATAGTCCCATATTTCTTGAAGGCTTTGTTCGTTTCTTTGTATTCTTTTTTCTCTAA 71896

Gorilla NNNNNNNNNNNNNNNNNNNNNNNAGGCTTTGTTCGTTTCTTTGTATTCTTTTTTCTCTAA 62989

*************************************

Human ACTTCCCTTCTCGCTTCATTTCATTCATTTCATCTTCCATCGCTGATATCCTTTCTTCCA 63082

Chimpanzee ACTTCCCTTCTCGCTTCATTTCATTCATTTCATCTTCCATCACTGATATCCTTTCTTCCA 71956

Gorilla ACNNNNNNNNNNNNNNNNNNNNNNNNNNNNNNNNNTCCATNNNNNNNNNNNTTTCTTCCA 63049

** ***** *********

Human GATAATCGCATCAGCTCCTGAGGCTTCTGCATTCTTCACATAGTTCTCGAGCCTTGCCTT 63142

Chimpanzee GATAATCGCATCAGCTCCTGAGGTTTCTGCATTCTTCACATAGTTCTCGAGCCTTGCCTT 72016

Gorilla GATANNNNNNNNNNNNNNNNNNNNNNNNNNNNNNNNNNNNNNNNNNNNNNNNNNNNNNNN 63109

****

Human TCAGCTCCATCAGCTCCTTTAAGGACTTCTCTGCATTGATTATTCTGGTTATCCATTCAT 63202

Chimpanzee TCAGCTCCATCAGCTCCTTTAAGGACTTCTCTGCATTGATTATTCTGGTTATCCATTCAT 72076

Gorilla NNNNNNNNNNNNNNNNNNNNAAGGACTTCTCTNCATTGATTNTTCTGGTTATCCNNNNNN 63169

************ ******** ************

Human CTAATTTTTTTTCAAAGTTTTTAACTTCTTTGCCATTGGTTTAAATTTCTCCTGTAGCTC 63262

Chimpanzee CTAATTTTTTTTCAAAGTTTTTAACTTCTTTGCCATTGGTTTAAATTTCTCCTGTAGCTC 72136

Gorilla NNNNNNNNNNNNNAAAGTTTTTAACTTATTTGCCATNNNNNNNNNNNNNNNNNNNNNNNN 63229

************** ********

Human GGAGTAGTTTGATCATCTGAAGCCTTCTTTTCTCAACTCATCAAAGTCATTCTCCGTCCA 63322

Chimpanzee GGAGTAGTTTGATCATCTGAAGCCTTCTTTTCTCAACTCATCAAAGTCATTCTCCGTCCA 72196

Gorilla NNNGTAGTTNNNNNNNNNNNNNNNNNNNNNNNNNNNNNNNNNNNNNNNNNNNNNNNNNNN 63289

******

Human GCTTTTTTCCATTGCTGGTGAGGAGCTGCATTCCTTTGGAGGAGGAGAAGCACTCTGCTT 63382

Chimpanzee GCTTTTTTCCATTGCTGGTGAGGAGCTGCATTCCTTTGGAGGAGGAGAAGCACTCTGCTT 72256

Gorilla NNNNNNNNNNNNNNNNNNNNNNNNNNNNNNNNNNNNNNNNNNNNNNNNNNNACTCTGCTT 63349

*********

Human TTTAGAGTTTCCAGTTTTTCTGCTCTGTTTTTTCCCCATGTTTGTGGTTTTATCTACTTT 63442

Chimpanzee TTTCGGGTTTCCAGTTTTTCTGCTCTGTTTTTTCCCCATGTTTGTGGTTTTATCTACTTT 72316

Gorilla TNNAGAGTTTCCAGTTTTTCTGCTCTGTTTTTTCCCCATGTTTGNNNNNNNATCTACTTT 63409

* * ************************************** *********

Human TCGTCTTTGATGATGGTGACATACAGATGGGTTTTTGGTGTGGGTGTCCTTTCTGTTTGT 63502

Chimpanzee TCGTCTTTGATGATGGTGACATACAGATGGGTTTTTGGTGTGGATGTCCTTTCTGTTTGT 72376

Gorilla TTGTCNTNNATGATGNNNNNNNACAGATGGGNNNNNNNNNNNNANNNNNNNNNNNNNNNN 63469

* *** * ****** *********

Human TAGTTTTCCCTCTAACAGACAGGACCCTCAGCTGCAGGTCTGTTGGAGTTTGCTAGAGGT 63562

Chimpanzee TAGTTTTCCCTCTAACAGACAGGACCCTCAGCTGCAGGTCTGTTGGAGTTTGCTAGAGGT 72436

Gorilla NNNNNNNNNNNNNNNNNNNNNNNNNNNNNNNNNNNNNNNNNNNNNNNNNNNNNNNNNNNN 63529

Human CCACTCCAGACCCTGTTTGCCTGGGTATCAGCAGCCATGGCTGCAGAACAGTGGTGGCAG 63622

Chimpanzee CCACTCCAGACCCTGTTTGCCTGGGTATCAGCAGCCATGGCTGCAGAACAGTGGTGGCGG 72496

Gorilla NNNNNNNNNNNNNNNNNTGCCTGGGNNNCAGCAGCCATGGCTGCAGAACAGTGGTGGCGG 63589

******** ****************************** *

Human TAGAACAGTGGATATTTGTGAACCGCAAATGCTGCTGCCTGATCGTTCCCCTGGAAGTTT 63682

Chimpanzee TAGAACAGTGGATATTTGTGAACCGCAAATGCTGCTGCCTGATCGTTCCCCTGGAAGTTT 72556

Gorilla TAGAACAGTGGCTATTTGTGNNNNGCAAATGCTGCTGCCTGATCGTTCCCCTGGAAGTTT 63649

*********** ******** ************************************

Human TGTCTCAGAGGAGTACCCGGCCGTGTGAGGTGTCAGTCTGCCCTTACTGCGGGGTGCCCC 63742

Chimpanzee TGTCTCAGAGGAGTACCCGGCCGTGTGAGGTGTCAGTCTGCCCTTACTGCAGGGTGCCTC 72616

Gorilla TGTCNNAGAGGAGTACCCGGCCNNNNNNNNNNNNNNNNNNNNNNNNNNNNNNNNNNNNNN 63709

**** ****************

Human CCAGTTAGGCTGCTCGGGGGTCAGGGACCCACTTGAGGAGGCAGTCTGCCATTCTCAGAT 63802

Chimpanzee CCAGTTAGGCTGCTCGGGGGTCAGGGACCCACTTGAGGAGGCAGTCTGCCATTCTCAGAT 72676

Gorilla NNNNNNNNNNNNNNNNNNNGTCAGGGACCCACTTGAGGAGGCAGTCTGCCATTCTCAGNN 63769

***************************************

Human CTCCAGCTGCGTGTTGGGAGAACCACTACTCTCTTCAAAGGTGTCAGACATGGACATTTA 63862

Chimpanzee CTCCAGCTGCGTGTTGGGAGAACCACTACTCTCTTCAAAGGTGTCAGACATGGACATTTA 72736

Gorilla NNNNNNNNNNNNNNNNNNNNNNNNNNNNNNNNNNNNNNNNNNNNNNNNNNNNNNNNNNNN 63829

Human AGTCTGCAGAGGTTACTGCTGTCTTTTTGTTTGTCTGTGCCCTGCCCCGAGAGGTGGAGC 63922

Chimpanzee AGTCTGCAGAGGTTACTGCTGTCTTTTTGTTTGTCCGTGCCCTGCCCCCAGAGGTGGAGC 72796

Gorilla NNNNNNNNNNNNNNNNNNNNNNNNNNNNNNNNNNNNNNNNNNNNNNNNNNNNNNNNNNNN 63889

Human CTACAGAGGCAGGCAGGCCTCCTTGAGCTGTGGTGGGCTCCACCCAGTTGGAGCTTCCCA 63982

Chimpanzee CTACAGAGGCAGGCAGGCCTCCTTGAGCTGTGGTGGGCTCCACCCAGTTGGAGCTTCCCA 72856

Gorilla NNNNNNNNNNNNNNNNNNNNNNNNNNNNNNNNNNNNNNNNNNNNNNNNNNNNNNNNNNNN 63949

Human GCTGCTTTGTTTACCTAATCAAGCCTGGGCAATGGCAGGCACCCTTCCCCCAGCCTTACT 64042

Chimpanzee GCTGCTTTGTTTACCTAATCAAGCCTGGGCAATGGCAGGCACCCTTCCCCCAGCCTTACT 72916

Gorilla NNNNNNNNNNNNNNNNNATCAAGCCTGGGCAATGGCAGGCACCCTTCCCCCAGCCTTACT 64009

*******************************************

Human GCCACCTTGCAGTTTGATCTCAGACTGCTGTGCGAGCAATCAGCAAGACTCCGTGGGTGT 64102

Chimpanzee GCCACCTTGCAGTTTGATCTCAGACTGCTGTGCTAGCAATCAGCAAGACTCCGTGGGTGT 72976

Gorilla GCCACCTTNNNNNNNNNNNNNNNNNNNNNNNNNNNNNNNNNNNNNNNNNNCCGTGGGTGT 64069

******** **********

Human AGGACCCTCTGAGCCAGGTGCAGGACATAATCTCCTGGTGTGCTGTTTTTCAAGCCCGTT 64162

Chimpanzee AGGACCCTCTGAGCCATGTGCAGGATATAATCTCCTGGTGTGCTGTTTTTCAAGCCCGTT 73036

Gorilla AGGACCCTCCGAGCCAGGTGCAGGATATAATCTCCTGGTGTGCTGTTTTTCAAGCCCGTT 64129

********* ****** ******** **********************************

Human GGAAAAGCTCAGTATTAGGTTGAGAGTGACCCGATCTTCCAGGTGCCGTCTGACACCCCT 64222

Chimpanzee GGAAAAGCTCAGTATTAGGTTGAGAGTGACCCGATCTTCCAGGTGCCATCTGTCACCCCT 73096

Gorilla GGAAAAGCTCAGTATTAGGTTGAGAGTGNCCCGNNNNNNNNNNNNNNNNNNNNNNNNNNN 64189

**************************** ****

Human TTCTTTGACTAGGAAAGGGAACTCCCTGACCCCTTGCACTTCCCGAGTGAGGCAATGCCT 64282

Chimpanzee TTCTTTGACTAGGAAAGGGAACTCCCTGACCCCTTGCACTTCCCGAGTGAGGCAATGCCT 73156

Gorilla NNNNNNNNNNNNNNNNNNNNNNNNNNNNNNNNNNNNNNNNNNNNNNNNGAGGCAATGCCT 64249

************

Human AGCCCTGCTTCAGCTCCTGCATGGGGCACTGCACCCACTGTC--------------TGGC 64328

Chimpanzee AGCCCTGCTTCAGCTCCTGCATGGGGCACTGCACCCACTGTCCTGCACCCACTGTCTGGC 73216

Gorilla AGCCCTGCTTCAGCTCCTGCATAGGGCACTGCACCNNNTGTC--------------NGGC 64295

********************** ************ **** ***

Human ACTCCCTAGTGAGATGAACCCGGTACCTCAGATGGAAATGCAGAAATCACCCATCTTCTG 64388

Chimpanzee ACTCCCTAGTGAGATGAATCCGGTACCTCAGATGGAAATGCAGAAATCACCCATCTTCTG 73276

Gorilla ACNCCCTAGTGAGATGAACCCGGTACCTCAGATGGAAATGCAGAAATCACCCATCTTCTG 64355

** *************** *****************************************

Human CGTCACTCACACTGGGAGCTGTGGACCGGAGCTGTTCTTATTCGGCCATCTTGGCTCCAC 64448

Chimpanzee CGTCGCTCACACTGGGAGCTGTGGACCGGAGCTGTTCTTATTCGGCCATCTTGGCTCCAC 73336

Gorilla CNNNNNNNNNNNNNNNNNNNNNNNNNNNNNNNNNNNNNNNNNNNNNNNNNNNNNNNNNNN 64415

*

Human CCCCATATATACTTTTTTATAATATATAAATGAATACCCATCTCCATATTTAAATTACAG 64508

Chimpanzee CCCCATATATACTTTTTTATAATATATAAATGAATACCCATCTCCATATTTAAATTACAG 73396

Gorilla NNNNNNNNNNNNNNNNNNNNNNNNNNNNNNNNNNNNNNNATCTCCATATTTAAATTACAG 64475

*********************

Human CTTTTTGTT-GGGCACGGCAGATCATGCCTGTAAGTCCCAGGACTTGGCGAGTTTGGGAT 64567

Chimpanzee CTTTTTTTTTGGGCACGGCAGATCATGCCTGTAAGTCCCAGGACTTGGCGAGTTTGGGAT 73456

Gorilla CTTTATGTT-GGGCACGGCAGATCNNNNNTGNNANNNNNNNNNNNNNNCGAGTNNNNNNN 64534

**** * ** ************** ** * *****

Human GGAAAGACTGCGAGAGCTCAGGAATTGGAGAGCAGCCTGCACAACATGGTAAATCCCTGT 64627

Chimpanzee GGAAAGACCGCGAGAGCTCAGGAATTGGAGAGCAGCCTGCACAACATGGTAAATCCCTGT 73516

Gorilla NNNNNNNNTGCTAGAGCTCAGGANNNNNNNNNNNNNNNNNNNNNNNNNNNNNATCCCTGT 64594

** *********** ********

Human CTCTACCAAAAATACAAAAATTATATAGGCATCACAGTGCATGCCTGTGGTCCCAGCTAC 64687

Chimpanzee CTCTACCAAACATACAAAAATTATATAGGCATCACAGTGCATGCCTGTGGTCCCAGCTAC 73576

Gorilla CTNNNNNNNNNNNNNNNNNNNNNNNNNNNNNNNNNNNNNNNNNNNNNNNNNNNNNNCTAC 64654

** ****

Human TGGAGACAGCGGCTGAGGTAAGAGGATTGCTTGAACCCCAGATGTTTAGGTTGCAGTGAG 64747

Chimpanzee TGGAGACAGCGGCTGAGGTAAGAGGTTTGCTTGAACCCCAGATGTTTAGGTTGCGGTGAG 73636

Gorilla TGGAGACAGCGGCTGAGGTAAGAGGATTGCTTGAACCCCAGATGTTTAGGTTACAGTGAG 64714

************************* ************************** * *****

Human TCCAGATCACACCACTGCACCATAGCCTAGGCCACAGAGCAAGATCCAGTCTCAAATAAA 64807

Chimpanzee TCCAGATCACACCACTGCACCATAGCCTAGGCCACACAGCAAGATCCAGTCTCAAATAAA 73696

Gorilla TCCAGATCACACCACTGCACNNTAGCCTAGGCCACAGAGCAAGATCCAGTCTCNAANNNN 64774

******************** ************** **************** **

Human TAAATAAATAAA------------------ATAAAATAAAATATATTACATCTTTATATG 64849

Chimpanzee TAAATAAATAAATAAATAAAATAAAATAAAATAAAATAAAATATATTACATCTTTATATG 73756

Gorilla NNNNNNNNNNNN------------------NNNNNNNNNNNNNNNNNNNNNNNNNNNNNN 64816

Human TGTGCCTAGTTCTGTTTAGTAAAATTATTATAAATAATAATAATATATACAATATACTTA 64909

Chimpanzee TGTACCTAGTTCTGTTTAGTAAAATTATTATAAATAGTAATAATATATACAATATACTTA 73816

Gorilla NNNNNNNNNNNNNNNNNNNNNNNNNNNNNNNNNNNNNNNNNNNNNNNNNNNNNNNNNNNN 64876

Human CATATCACTGTATTAGTTCATTTTCAGACTGTTTTAAAGAACTACTGGAGACTGGGTAAT 64969

Chimpanzee CATATCACTGTATTAGTTCATTTTCAGACTGTTTTAAAGAACTACTGGAGACTGGGTAAT 73876

Gorilla NNNNNNNNNNTNTTAGTTCATTTTCAGACTGTTTTAAAGAACTACTGGAGACTGGGTAAT 64936

* ************************************************

Human TTTTAAAGAAAAGAGGTTTAATAACTTAGAGTGCTGCAGGGCCTGGGAGGCCTCACGGAA 65029

Chimpanzee TTTTAAAGAAAAGAGGTTTAATAACTTAGAGTGCTGCAGGGCCTGGGAGGCCTCACGGAA 73936

Gorilla TTTTAAAGAAAAGAGGTTTAATAACTNNNNGTGCTGCAGGGCCTGGGAGGCCTCATGGAA 64996

************************** ************************* ****

Human CTTATAATCATGGCGGAAGGGGAAGCGGAAGCAAGGCACATCTTCTCATGGCAGCAGGAG 65089

Chimpanzee CTTATAATCATGGCGGAAGGGGAAGCGGAAGCAAGGCACATCTTCTCATGGCAGCAGGAG 73996

Gorilla CTTATAATCATGGCGGAAGGGGAAGCGGAAGCAAGGCACATCTTCTCATGGCAGCAGGAG 65056

************************************************************

Human GGAGAGGGAGCAAGGAAGTGCCACATTTTTAAACCATTAGATCTCGTGAGAACTCACTAT 65149

Chimpanzee GGAGAGGGAGCAAGGAAGTGCCACATTTTTAAACCATTAGATCTCGTGAGAACTCACTAT 74056

Gorilla GGAGAGGGAGCAAGGAAGTGCCACATTTTTAAACCATTAGATCTCGTGAGAACTCACTAT 65116

************************************************************

Human CTTGAGAACAGCAAGGGAAAAATCCCCTAGGATCCAATCACCTCCCATCAGGCACCTCCC 65209

Chimpanzee CTTGAGAACAGCAAGGGAAAAATCCCCTAGGATCCAATCACCTTCCATCAGGCACCTCCC 74116

Gorilla CATGAGAACAGCAAGGGAAAANNNNNNNNNNNNNNNNNNNNNNNNNNNNNNNNNNNNNNN 65176

* *******************

Human TGGACATGTGGGGATTACACTTCACAGTGAGGTTTTAGTGGGAACGCAGAGCCAAATGCC 65269

Chimpanzee TGGACACGTGGGGATTACACTTCACAGTGAGGTTTTAGTGGGAACGCAGAGCCAAATGCC 74176

Gorilla NNNNNNNNNNNNNNNNNNNNNTCACAATGAGGTTTTAGTGGGAATGCAGAGCCAAATGCC 65236

***** ***************** ***************

Human ATCAATAATATGTAATTACATTTATTTATACATGTACACTGATATATTTATATATAGACT 65329

Chimpanzee ATCAATAATATGTAATTACATTTATTTATACATGTACACTTATATATTTATATATACACT 74236

Gorilla ATCAATAATATGTAATTACATTTATTTATACATGTACACTTATATATTTATATATACACT 65296

**************************************** *************** ***

Human TGTATATATGTAAACATACATTTATGTATTTATTTACAAACTTGTATACATATAAATTGA 65389

Chimpanzee TGTATATATGTAAACATAGATTTGTGTATTTATTTACAAACTTGTATACATATAAATTGA 74296

Gorilla TGTATATATGTAAACATACATTTATGTNNNTNNNNNCAAACTTGTATACATCTAAATTGA 65356

****************** **** *** * *************** ********

Human TATACAGATGGTCTTTCACTTGAGATGGTCCCATTTACAACTGCTGGACTTAATGGTGGT 65449

Chimpanzee TATACAGATGGTCTTTCACTTAAGATGGTCCCATTTACAACTTCTGGACTTAATGGTGGT 74356

Gorilla TATACAGATGGTCTT-CACTNAAGANNNNNNNNNNNNNNNNNNNNNNNNNNNNNNNNNNN 65415

*************** **** ***

Human GTGAGAATAGCATGCATGCAGTAGAAAGTGCTGTTTTCCCCTCTTGCCAGGCTACAGGGA 65509

Chimpanzee GTGAGAATAGCATGCATGCAGTGGAAAGTGCTGTTTTCCCCTCTTGCCAGGCTACAGGGA 74416

Gorilla NNNNNNNNNNNNNNNNNNCAGTAGAAAGTTCTGTTTTCCCCTCTTGCCAGGCTACAGGGA 65475

**** ****** ******************************

Human GCCAGTTCCCACTCAGCCTTGCAATCACAAAGGTTCATAGGCAAAACTCTACAGTGTTCT 65569

Chimpanzee GCCAGTTCCCACTCAGCCTTGCAATCACAAAGGTTCATAGGCAAAACTCTACAGTGTTCT 74476

Gorilla GCCAGTTCCCACTCAGCCTTGCAATCACAATGGTTCATAGGCAAAACTCTACANNNNNNN 65535

****************************** **********************

Human GTGTTGCTAGAGGAGTTTGCTCCATGATAGGTTATACAAGTGCTTTGAGCATATTTAAGG 65629

Chimpanzee GTGTTGCTAGAGGAGTTTGCTCCATGATAGGTTATACAAGTGCTTTGAGCATATTCAAGG 74536

Gorilla NNGNNNNNNNNNGAGTTTGCTCCATGATAGGTTATACAAGCGCTTTGAGCNNNNNNNNNN 65595

* **************************** *********

Human TATGCTAGGCAAAGCTATGATGTTTAGTAGGTTAGGTGTATTCAATGCATTTTCAAATTT 65689

Chimpanzee TATGCTAGGCAAAGCTATGATGTTTAGTAGGTTAGGTGTATTCAGTGCATTTTCAAATTT 74596

Gorilla NNNNNNNNNNNNNNNNNNNNNNNNTAGTAGGTTAGGTGTATTCAAAGCATTTTCAAATTT 65655

******************** **************

Human TGGTATTTTCAACTTGCTATGGGTTTATTGGGACATAACCCCATCATAAGTTAAGGAGCG 65749

Chimpanzee TGGTATTTTCAACTTGCTATGGGTTTATTGGGACATAACCCCATCATAAGTTAAGGAGCA 74656

Gorilla TGGTATTTTCAACTTGCTATGGGTTTATTGGGACATAACCCCATCATAAGTTAAGGAGCA 65715

***********************************************************

Human TTTGTACATAAACTTGTGTTTGTGTGTGTGAGTGTGTGTGTGTGTGTGTGTGTGTGTATA 65809

Chimpanzee TTTGTACATAAACTTGTGTTTGTGTGTGTGAGTGTGTGTGTGTGTGTG------TGTATA 74710

Gorilla TTTGTACATAAACTTGTGTTTGTGTGTGTGANNNNNNNNNNNNGTGTGTNNNNNNNNNNA 65775

******************************* ***** *

Human TACACCTACACATATATATGTATATGGTACATATCTATAACTTGGATCTATGCATATTTC 65869

Chimpanzee CACACCTACACATATATATGTATATGGTACATATCTATAACTTGGATGTACGCATATTTC 74770

Gorilla CACACCTACACATATATATGTATATNNNNNNNNNNNNNNNNNNNNNNNNNNNNNNNNNNN 65835

************************

Human ATATGTTTTTGAATATTTAATATATACATTTTAAACCAATACATACATTTATATGAACAT 65929

Chimpanzee ATATGTTTTTGAATATTTAATATATACATTTTAAACCAATACATACATTTATATGAACAT 74830

Gorilla NNNNNNNNNNNNNNNNNNNNNNNNNNNNNNNNNAGCCAATACATACATTTATATGAACAT 65895

* *************************

Human TTAACATATACAGTTAGCCGTTGAGCAACATGGGAGGTAAGGAACAAACCTCCATCCGAC 65989

Chimpanzee TTAACATATACAGTTAGCCGTTGAGCAACATGGGAGGTAAGGAACAAAGCTCCATCCGAC 74890

Gorilla TTAACATATACNGTTAGCCGTTGAGCANNNNNNNNNNNNNNNNACAAATNNNNNNNNNNN 65955

*********** *************** *****

Human TCTCATGTATAGTTGAAAATCCATGTATAATTTTTACTCCCGCAAAACTGAAGTATTAAT 66049

Chimpanzee TCTCATGTATAGTTGAAAATCCATGTATAATTTTTACTCCCGCAAAACTGAAGTATTGAT 74950

Gorilla NNNNNNNTATNNNNNNNNNNNNNNNNNNNNNNNNNNNNNNNNNNNNNNNNNNNNNNNNNN 66015

***

Human AGTATACTGTAGATTGGCTATTACCAATGATATGAATGGTCAATTAACACACATTTTGTA 66109

Chimpanzee AGTATACTGTAGATTGGCTATTACCAATGATATGAATGGTCAATTAACACACATTTTGTC 75010

Gorilla NNNNNNNNNNNNNNNNNNNNNNNNNNNNNNNATGAATGGTCAATTAACACACATTTTGTA 66075

****************************

Human CATTATATGTACTATATACTGTATTTTTACAATAACGTAAGATATAGAAAATAAAATGTC 66169

Chimpanzee CATTATATGTATTATATACTGTATTTTTACAATAACGTAAGATATAGGAAAGAAAATGTC 75070

Gorilla CATTATATGTANNNNNNNNNNNNNNNNNNNNNNNNNNNNNNNNNNNNNNNNNNNNNNNNN 66135

***********

Human ATTAAGAATATTCCAAGAAAGAGAAAATACATTTATTATTCACAAAGTGGAAGTGGATCA 66229

Chimpanzee ATTAAGAATATTCCAAGAAAGAGAAAATACATTTATTATTCACAAAGTGGAAGTGGATCA 75130

Gorilla NNNNNNNNNNNNNNNNNNNNNNNNNNNNNNNNNNNNNNNNNNNNNNNNGGAAGTGGATCA 66195

************

Human TCTTGAAGGCCTTCAGCCCCATTGTCTTCACCTGGAGTGGACTGAGGAGGAAGAAGGGAA 66289

Chimpanzee TCTTGAAGGCCTTCAGCCCCATTGTCTTCACCTGGAGTGGACTGAGGAGGAAGAAGGGAA 75190

Gorilla TCTTGAAGGCCTTCAGCCCCATTGTCTTCACCTGGAGTGGACTGAGGAGGAAGAAGGGAA 66255

************************************************************

Human AGGGTTGGTCTTGCTATCTCAATGGTGGCAGAGGTGGAAGGAAATCCACATATTAGTGAC 66349

Chimpanzee AGGGTTGGTCTTGCTATCTCAATGGTGGCAGAGGTGGAAGGAAATCCACATATTAGTGAC 75250

Gorilla AGGGTTGGTCTTGCTATCTCAATGGTGGCAGAGGTGGAAGGAAATCCACATATTAGTGAC 66315

************************************************************

Human TCACACAGTTCAAACCCATGTTGTTCAAGGGGCAGCTGTATATGCATATACACACATACA 66409

Chimpanzee TCACACAGTTCAAACCCATGTTGTTCAAGGGTCAGCTGTATATGCATATACACACATACA 75310

Gorilla TCACACGGTTCAAACCCATGTTGTTCAAGGGTCAGCTGTATATGCATATACACACATACA 66375

****** ************************ ****************************

Human TACACATTTATATTTAAATTATATCATTATATATGTAGCTACTTGTGTTCAGTAAAATTA 66469

Chimpanzee TACACATTTATATTTAAATTATATCATTATATATGTAGCTACTTGTGTTCAGTAAAATTA 75370

Gorilla TACACATTTATATTTAAATTATATCATTATATACGTAGCTACTTGTGTTCAGTAAAATTA 66435

********************************* **************************

Human ATTTTGTTACACTCTTGAATATTTTTTGTTATTTACATTTCTTCCATATGTTTCATGATT 66529

Chimpanzee ATTTTGTTACACTCTTGAATATTTTTTGTTATTTACATTTCTTCCATATGTTTCATGATT 75430

Gorilla ATTTTGTTACACTCTTGAATGTTTTTTGTTATTTACATTTCTTCCATATGTTTCATGATT 66495

******************** ***************************************

Human TCAAGACACAAAAAAATAGACACAATATTCTGACTGAATAATTATGTTATGGGCATCCAG 66589

Chimpanzee TCAAGACACAAAAAAATAGACACAATATTCTGACTGAATAATTATGTTATGGGCATCCAG 75490

Gorilla TCAAGACACAAAAAAATAGACACAATATTCTGACTGAATAATTATGTTATGGGCATCCAG 66555

************************************************************

Human GTAGTTCTGTTTAGACAGTTGCTAAACCACCAGCTACACAGACACATTTTATAGAATTGC 66649

Chimpanzee GTAGTTCTGTTTAGACAGTTGCTAAACCACCAGCTACACAGACACATTTTATAGAATTGC 75550

Gorilla GTAGTTCTGTTTAGACAGTTGCTAAACCACCAGCTACACAGACACATTTTATAGAATTGC 66615

************************************************************

Human CATATAAAAGGAGATACACTAGATTATTTTTATGTCATTAATTGATATACAACATGGGCA 66709

Chimpanzee CATATAAAAGGAGATACACTAGATTATTTTTATGTCATTAATTGATATACAACATGGGCA 75610

Gorilla TATATAAAAGGAGATACATTAGATTATTTTTATGTCATTAATTGATATACAACATGGGCA 66675

***************** *****************************************

Human TTTTTACCCAAGGGCTTCCTCTGTCATAGGATGTTTACTCAATTTATGTGAACAAATTCC 66769

Chimpanzee TTTTTACCCAAGGGCTTCCTCTGTCATAGGATGTTTACTTAATTTATGTGAACAAATTCC 75670

Gorilla TTTTTACCCAAGGGCTTCCTCTGTCATAGGATGTTTACTCAATTTATGTGAACAAATTCC 66735

*************************************** ********************

Human AATTTGCAGTGGTGTGCCCTAAAAGCCAAAAAGAATGCAGTGAATTTTAAATCTAGTGTG 66829

Chimpanzee AATTTGCAGTGGTGTGCCCTAAAAGCCAAAAAGAATGCAGTGAATTTTAAATCTAGTGTG 75730

Gorilla AATTTGCAGTGGTGTGCCCTAAAAGCCAAAAAGAATGCAGTGAATTTTAAATCTAGTGTG 66795

************************************************************

Human TTTGATGTTCTAAATTTGGAAAATATTTTAAAAATGTTCATTGTTACAGCAATTCTTACA 66889

Chimpanzee TTTGATGTTCTAAATTTGGAAAATATTTTAAAAATGTTCATTGTTACAGCAATTCTTACA 75790

Gorilla TTTGATGTTCTAAATTTGGAAAATATTTAAAAAATGTTCATTGTTACAACAATTCTTACA 66855

**************************** ******************* ***********

Human TGTGTGTATATATGTGTGTGTGTGTCTGTGTGTGTGTATAAAATAAATGCCATATATATA 66949

Chimpanzee TGTGTGTATATACATGTGTGTGTGTCTGTGTGTGTGTATAAAATAAATGTCATATATATA 75850

Gorilla TGTGTGTATATA--TGTGTGTTTGTCTGTGTGTGTGTATAAAATAAATGTCATATATATA 66913

************ ******* *************************** **********

Human CATATACATTTATATATTCGTTGCATTTGTGCTTTTTATTAAATATAATGATTATTGGTA 67009

Chimpanzee CATATACATTTATATATTCGTTGCATTTGTGCTTTTTATTAAATATAATGATTATTGGTA 75910

Gorilla CATATACATTTATATATTCATTGCATTTGTGCTTTTTATTACATATAATGATTATTGGTA 66973

******************* ********************* ******************

Human AATTTGAAGCTTGTTTGATGGCAGTCCCCTCCCTATAGCTGCCAAGGTGATGGTGGATTG 67069

Chimpanzee AATTTGAAGCTTGTTTGATGGCAGTCCCCTCCCTATAGCTGCCAAGGTGATGGTGGATTG 75970

Gorilla AATTTGAAGCTTGTTTGATGGCAGTCCCCTCCCTATAGCTGCCAAGGTGATGGTGGATTG 67033

************************************************************

Human TTGCTTTCAACAAATTTCACTTGGTAAACTTCAACTTTACCAATAATAATTTTATTTAAT 67129

Chimpanzee TTGCTTTCAACAAATTTCACTTGGTAAACTTCAACTTTACCAATAATAATTTTATTTAAT 76030

Gorilla TTGCTTTCAACAAATTTCACTTGGTAAACTTCNNNNNNNNNNNNNNNNNNNNNNNNNNNN 67093

********************************

Human GAAAAAAAGTAATGTAAATGTAATATTTTAAAAATTCATCTCTAATCTCTGTTTTTTTCC 67189

Chimpanzee GAAAAAAAGTAATGTAAATTTAATATTTTAAAAATTCATCTCTAATCTCTGTTTTTTTC- 76089

Gorilla NNNNNNNNNNNNNNNNNNNNNNNNNNNNNNNNNNNNNNNNNNNNNNNNNNNNNNNNNNNN 67153

Human AAGGGTCAACTGTACATGTTAAATGCTTATTAAAATGTATATATTGTTCAAAGGTATATA 67249

Chimpanzee AAGGGTCAACTGTACATGTTAAATGCTTATTTAAATGTATATATTGTTCAAAAGTATATA 76149

Gorilla NNNNNNNNNNNNNNNNNNNNNNNNNNNNNNNNNNNNNNNNNNNNNNNNNNNNNNNNNNNN 67213

Human TAAATGTGTATGACATTTGTTTTATTATATATACACAAATCTATATACATACATTTACAC 67309

Chimpanzee TAAATGTGTATGACATTTGTTTTATTATGTATACACAAATCTATATACATACATTTACAC 76209

Gorilla NNNNNNNNNNNNNNNNNNNNNNNNNNNNNNNNNNNNNNNNNNNNNNNNNNNNNNNNNNNN 67273

Human ATACATACTGATTATTCTAACAGTGCATATGTACATATTGATATAA-TATGTATATATAA 67368

Chimpanzee ATACATACTGATTATTCTAACAGTGCATATGTACATATTGATATAAATATGTATATATAA 76269

Gorilla NNNNNNNNNNNNNNNNNNNNNNNNNNNNNNNNNNNNNNNNNNNNNN-NNNNNNNATATAA 67332

******

Human ATGTATATGACATTTTTCTTTATGTATATACACATATAAGCATACCTATATGTAACATGC 67428

Chimpanzee ATGTATATGACATTTTTCTTTATGTATATACACATATAAGCATACCTATATGTAACATGC 76329

Gorilla ATGTATATGACATTTTTCTTTATGTATATACACACATAAGCATACCTATATGTAACATGC 67392

********************************** *************************

Human TGATTTACTATAACAGAGCACATGTATATGTTGATATAAAATATATATAAATGTATAGGA 67488

Chimpanzee TGACTTACTATAACAGAGCACATGTATATGTTGATATAAAATATATATAAATGTATAGGA 76389

Gorilla TGATTTACTATAACAGAGCACATGTATATGTTGATATAAAATATATATAAATGTATAGGA 67452

*** ********************************************************

Human CATTAGTTTTACACATATATACACACATACACATACACAACACACACATGCTAATTATAA 67548

Chimpanzee CATTAGTTTTACACATATATACACACATACACATACACAACACACACATGCTAATTATAA 76449

Gorilla CATTAGTTTTACACATATATACACACATACACATACACAACACACACATTCTAATTATAA 67512

************************************************* **********

Human CAGTGCACATCTTTTAAATGTTTTCCAAATTAAGAACATCAAGCACACTATATTTAAAAT 67608

Chimpanzee CAGTGCACATCTTTTAAATGTTTTCCAAATTAAGAACATCAAGCACACTATATTTAAAAT 76509

Gorilla CAGTGCACGTCTTTTAAATGTTTTCCAAATTAAGAACATCAAGCACACTATATTTAAAAT 67572

******** ***************************************************

Human TCACTCTATTCTATTTGCCTTCTCGGGCATTCCTTTGCAAATTGAAACTTGTTCACATAC 67668

Chimpanzee TCACTCTATTCTATTTGCCTTCTAGGGCATTCCTTTGCAAATTGAAACCTGTTCACATAC 76569

Gorilla TCACTCTATTCTATTTGCCTTCTAGGGCATTCCTTTGCAAATTGAAACTTGTTCACATAC 67632

*********************** ************************ ***********

Human ACTGTGTAAACATCTTGTGAGAGAGAAAACCACTGGTGACAAATGCTGAAGTTGTGTATC 67728

Chimpanzee AGTGTGTAAACATCTTGTGAGAGAGAAAACCACTGGGGACAAATGCTGAAGTTGTGTATC 76629

Gorilla ACTGTGTAAACATCTTGTGAGAGAGAAAACCACTGGGGACAAATGCTGAAGTTGTGTATC 67692

* ********************************** ***********************

Human AATCAATTATATTCTCATAATGTCTTCCAAAGTACATTTTTATATTGTCAGTAAAAATAT 67788

Chimpanzee AATCAATTATATTCTCATAATGTCTTCCAAAGTACATTTTTATATTGTCAGTAAAAATAT 76689

Gorilla AATCAATTATATCCTCATAATGTCTTCCAAAGTACATTTTTATATTGTCAGTAAAAATAT 67752

************ ***********************************************

Human ATGTATCACTAAACTACAGCTGGTGAATATTTTTTAAATTTACTTTAGAAGTACATTATT 67848

Chimpanzee ATGTATCAATAAACTATAGCTGGTGAATATTTTTTAAATTTACTTTAGAAGTACATTATT 76749

Gorilla ATGTATCACTAAACTATAGCTGGTGAATATTTTTAAAATTTACTTTAGAAGTACATTATT 67812

******** ******* ***************** *************************

Human ATATACATCTTTTTGTAATTATTTTATTGCCCTTTTAGATAGAGCCAATTTGTATAACTG 67908

Chimpanzee ATATACATCTTTTTGTAATTATTTTATTGCCCTTTTAGATAGAGCCAATTTGTATAACTG 76809

Gorilla ATGTACATCTTTTTGTAATTATTTTATCGCCCTTTTAGATAGAGCCAATTTGTATAACTG 67872

** ************************ ********************************

Human TAATATACCTGGTATGCATACACTATGAGCTATTAATTCTTAATTACTCCCTTTTCCTTG 67968

Chimpanzee TAATATACCTGGTATGCATACACTGTGAGCTATTAATCCTTAATTACTCCCTTTTCCTTG 76869

Gorilla TAATATATCTGGTATGCATACACTATGAGCTATTAATCCTTAATTACTCCCTTTCCCTTG 67932

******* **************** ************ **************** *****

Human ATCTAAGTCATTTCCACTTTTCCTCTTCTACAAACAATATGGCAAGAAACAAAATTATTA 68028

Chimpanzee ATCTAAGTCATTTCCACTTTTCCTCTTCTACAAACAATATGGCAAGAAACAAAATTATTA 76929

Gorilla ATCTAAGTCATTTCCACTTTTCCTCTTCTACAAACAATATGGCAAGAAACAAAATTATTA 67992

************************************************************

Human ATGCCTCTTTGTGTACATATGTGAGCATTTATTTGGCGGTATTTTGTTAATGCTCTTTAT 68088

Chimpanzee ATGCCTCTTTGTGTACACATGTGAGCATTTATTTGGCAGTATTTTGTTAATGCTCTTTAT 76989

Gorilla ATGCCTCTTTGTGTACATATGTGAGCATTTATTTGGTGGCATTTTGTTAATGCTCTTTAT 68052

***************** ****************** * ********************

Human AAATATTAAGCTTATTTGCCTCAACAAAAGTCTAGTTGCCTTAATTCATAAAATGAAAAT 68148

Chimpanzee AAATATTAAGCTTATTTGCCTCAACAAAAGTCTAGTTGCCTTAATTCATAAAATGAAAAT 77049

Gorilla AAATATTAAGCTTATTTGCCTCAACAAAAGTCTAGTTGCCTTAATTCATAAAATTAAAAT 68112

****************************************************** *****

Human CTAAAGCCTAGTTACTAAATTGCTCAAATCACACACCTAGCAGGAAAAAAGCTGAGATCA 68208

Chimpanzee CTAAAGCCTAGTTACTAAATTGCTCAAATCACACACCTAGCAGGAAAAAAGCTGAGATCA 77109

Gorilla CTAAAGCCTAGTTACTAAATTGCTCAAATCACACACCTAGCAGGAAAAAAGCTGAGATCA 68172

************************************************************

Human AATCCAGCCTGTCTGTCCCAAAAACCCCAATAATTAAACATGTCTTGCCTTCTTAAAGGT 68268

Chimpanzee AATCCAGCCTGTCTGTCCCAAAAACCCCAATAATTAAACATGTCTTGCCTTCTTAAAGGT 77169

Gorilla AATCCAACCTGTCTGTCCCAAAAACCCCAATAATTAAACATGTCTTGCCTTCTTAAAGGT 68232

****** *****************************************************

Human ACTGTTGAGTAACAAATGCCATTGCAGACTTTCCATTTCATTAGACAATACCAAATGGCT 68328

Chimpanzee ACTGTTGAGTAACAAATGCCATTGCAGACTTTCCATTTCATTAGACAATACCAAATGGCT 77229

Gorilla ACTGTTGAGTAACAAATGCCATTGCAGACTTTCCATTTCATTAGACAATACCAAATGACT 68292

********************************************************* **

Human TTTCCAAAATTGGGTACAAATTCCCTTTTGCTACAATTCTGCAACAATGAATACAGCAGA 68388

Chimpanzee TTTCCAAAATTGGGTACAAATTCCCTTTTGCTACAATTCTGCAACAATGAATACAGCAGA 77289

Gorilla TTTCCAAAATTGGGTACAAATTCCCTTTTGCTACAATTCTGCAACAATGAATACAGCAGA 68352

************************************************************

Human CATTTGAATTTTTGCTAAGCTAATGGCTGGGAAATTCCCTTTCTTTGTTTAAATTTTTCA 68448

Chimpanzee CATTTGAATTTTTGCTAAGCTAATGGCTGGGAAATTCCCTTTCTTTGTTTAAATTTTTCA 77349

Gorilla CATTTGAATTTTTGCTAAGCTAATGGCTGGGAAATTCCCTTTCTTTGTTTAAATTTTTCA 68412

************************************************************

Human TGGCTTTGATTTCTGGTGGATTGGAATTTAATGAACTATGTTTATTGAATGATCTGTCAG 68508

Chimpanzee TGGCTTTGATTTCTGGTGGATTGGAATTTAATGACCTATGTTTATTGAATGATCTGTCAG 77409

Gorilla TGGCTTTGATTTCTGGTGGATTGGAATTTAATGACCTGTGTTTATTGAATGATCTGTCAG 68472

********************************** ** **********************

Human ATTTTCCACATTAGGGGAATTAGGCTTGTCTTGTTTTGTTTGTTTGTTTGTTTCTTTGTT 68568

Chimpanzee ATTTTCCACATTAGGGGAATTAGGCTTGTCTTGTTTTGTTTGTTTGTCTGTTTGTTTGTT 77469

Gorilla GTTTTCCACATTAGGGGAATTAGGCTTGTCTTGTTTTGTTTGTTTGTTTGTTTCTTTGTT 68532

********************************************** ***** ******

Human TTAAGACCCAGAGACTGGAGTTCCTGTCACCCACCCTAATTTAGTATTATTTTTTTAGTT 68628

Chimpanzee TTAAGACCCAGAGACTGGAGTTCCTGTCACCCACCCTAATTTAGTATTATTTTTTTAGTT 77529

Gorilla TTAAGACCCAGAGACTGGAGTTCCTGTCACCCACCCTAATTTAGTGTTTTTTTTTTAGTT 68592

********************************************* ** ***********

Human GAATGGGTCGAAATGAAATTTGACAATGATACATATTTACTTTTATTTTCTAATTATTAA 68688

Chimpanzee GAATGGGTCGAAATGAAATTTGACAATGATACATATTTACTTTTATTTTCTAATTATTAA 77589

Gorilla GAATGGGTCGAAATGAAATTTGACAATGATACATATTTACTTTTATTTTCTAATTATTAA 68652

************************************************************

Human AAGTGGCTCTTGCAACAGGCATTCCCAGAGGAAATGGGAAAGCTTTGTGGAAATATGAGA 68748

Chimpanzee AAGTGGCTCTTTCAACAGGCATTCCCAGAGGAAATGGGAAAGCTTTGTGGAAATATGAGA 77649

Gorilla AAGTGGCTCTTTCAACAGGCATTCCCAGAGGAAATGGGAAAGCTTTGTGGAAATATGAGA 68712

*********** ************************************************

Human ACAAGGCTGTGAAAATCTGATTTTGCTTCATTGAAAGCTGACACGAGATAGATAGTACCT 68808

Chimpanzee ACAAGGCTGTGAAAATCTCATTTTGCTTCATTGAAAGCTGACACGAGATAGATAGTACCT 77709

Gorilla ACAAGGCTGTGAAAATCTCATTTTGCTTCATTGAAAGCTGACACGAGATAGATAGTACCT 68772

****************** *****************************************

Human ACTTTTAAATTTAATTTTCCTCATACTTGTCAGTAAAAAATGTTTCTTATGCTTGAAAAG 68868

Chimpanzee ACTTTTAAATTTAATTTTCCTCATACTTGTCAGTAAAAAATGTTTCTTATGCTTGAAAAG 77769

Gorilla ACTTTTAAATTTAATTTTCCTCATACTTGTCAGTAAAAAATGTTTCTTATGCTTGAAAAG 68832

************************************************************

Human TTCCTTGCCAGAATATGTCATCTGTGAAG---GTGCAAAACTCATTATAAAATGGATGGC 68925

Chimpanzee TTCCTTGCCAGAATATGTTATCTGTGAAGTTTGTGCAAAAGTCATTATAAAATGGATGGC 77829

Gorilla TTCCTTGCCAGAATATGTTATCTGTGAAG---GTGCAAAACTCATTATAAAATGGATGGC 68889

****************** ********** ******** *******************

Human TCAGCATTATACTTGTATAATCATGTCACCCCAGCAAAAGCTTTAAAGCCGTGAGAAATG 68985

Chimpanzee TCAGCATTATACTTGTATAATCATGTCACCCCAGCAAACGCTTTAAAGCCGTGAGAAATG 77889

Gorilla TCAGCATTATACTTGTATAATCATGTCACCCCAGCAAAAGCTTTAAAGCCGTGAGAAATG 68949

************************************** *********************

Human CTATTTTACTTGCCCACTATAAAACTTGAATGGCATAAATTGAAACCATTTATATGTATA 69045

Chimpanzee CTATTTTACTTGCCCACTACAAAACTTGAATGGCATAAATTGAAACCATTTATATGTATA 77949

Gorilla CTATTTTACTTGCCCACTACAAAACTTGAATGACATAAATTGAAACCATTTATATGTATA 69009

******************* ************ ***************************

Human CAGACACACACATACACACACACACACACACACACACAAATATATAAGACTTCTATTCAT 69105

Chimpanzee ----CACACACACACACACACACACACACACACACACAAATATATAACACTTCTATTCAT 78005

Gorilla AAGACACACACACACACACACACACACACACACACACNNNNNNNNNACACTTCTATTCAT 69069

******** ************************ * ************

Human TTTAATTTAATTTTATTTTATTAATTAATTTTGAGACAGCCTGTTGTCCAGGCTGGAATG 69165

Chimpanzee TTTAATTTTATTTTATTTTATTAATTAATTTTGAGACAGCCTGTTGTCCAGGCTGGAATG 78065

Gorilla TTTAATTTTATTTTATTTTATTAATTAATTTTGAGACAGCCTGTTGTCCAGGCTGGAGTG 69129

******** ************************************************ **

Human CAGTGGAATGATCCCTGCTCAGTGCAGCCTCTGCCGCCCAGGTTCAATTGATTCTTCTGC 69225

Chimpanzee CAGTGGAATGATCCCTGCTCAGTGCAGCCTCTGCCGCCCAGGTTCAATTGATTCTTCTGC 78125

Gorilla CAGTGGAATGATCCCTGCTCAGTGCAGCCTCTGCCGCCCAGGTNCANNTGATTCTTNNNN 69189

******************************************* ** ********

Human TTCAGCTGGGAGTAGATGGGATTACAGGAACCCACCATCATGCCCAGCTAACTTTTGTAT 69285

Chimpanzee TTCAGCTGGGAGTAGATGGGATTACAGGCACCCACCATCATGCCCAGCTAACTTTTGTAT 78185

Gorilla NNNAGCTNNNNNNNNNNNNNNCTACAGGCACCCACCATNNNNNNNNNNNNNNNNNNNNNN 69249

**** ****** *********

Human TTTTAGTAAAGATGGTTTTTTTTTTTTTCATGTTAGCCAGGATGGTTGGTGTCAGACTCC 69345

Chimpanzee TTTTAGTAAAGATGGTTTTTTTTTTTTTCATGTTAGCCAGGATGGTTGGTGTCAGACTCC 78245

Gorilla NNNNNNNNNNNNNNNNNNNNNNNNNNNNNNNNNNNNNNNNNNNNNNNGGTGTCAGACTCC 69309

*************

Human TGGCCTCAAGTGATTCGCCTGCTTCAGACTCCCAAAGTGCTGTGATTACAGGCACGAGCC 69405

Chimpanzee TGGCCTCAAGTGATTCGCCTGCTTCAGACTCCCAAAGTGCTGTGATTACAGGCACGAGCC 78305

Gorilla TGGCCTCAAGTGATTCGCCTGCTTCAGACTCCCAAAGTGCTGTGATTNNNNNNNNNNNNN 69369

***********************************************

Human ACCGCGCCCAGCTTCTACTTGTTTTAAAACACCTCATTTGCTCATTTTATACCTGAGATA 69465

Chimpanzee ACCGCGCCCAGCTTCTACTTGTTTTAAAACACCTCATTTGCTCATTTTATACCTTAGATA 78365

Gorilla NNNNNNNNNNNNNNNNNNNNNNNNNNNNNNNNNNNNNNNNNNNNNNNNNNNNNNNNNNNN 69429

Human TAAATTTTATCAAGGACTGCTTTTTTTGTAAGCAGTCCTAGACAGTCGTAGATAGCATTT 69525

Chimpanzee TAAATTTTATCAAGGACTGCTTTTTT-GTAAGCAGTCCTAGACAGTCGTAGATAGCATTT 78424

Gorilla NNNNNNNNNNNNNNNNNNNNNNNNNNNNNNNNNNNNNNNNNNNNNNNNNNNNNNNNNNNN 69489

Human GTATTTATATTAAAAATAATATATGTATGTAATATAAATAAATTTATATTTATAATATAT 69585

Chimpanzee GTATTTATATTAAAAATAATATATGTATGTAATATAAATAAATTTATATTTATAATATAT 78484

Gorilla NNNNNNNNNNNNNNNNNNNNNNNNNNNNNNNNNNNNNNNNNNNNNNNNNNNNNNNNNNNN 69549

Human AATATAATATATAAATATAATTATGTTATTTAATATATAATATAGTATATTTATAGTATA 69645

Chimpanzee AATATAATATATAAATATAATTATGTTATTTAATATATAATATAGTATATTTATAGAATA 78544

Gorilla NNNNNNNNNNNNNNNNNNNNNNNNNNNNNNNNNNNNNNNNNNNNNNNNNNNNNNNNNNNN 69609

Human TATAATAATTTACTATAAGTATATTTATAGTATATATTATATTTTTATTATATAAATATG 69705

Chimpanzee TATAATAATTTACTATAAGTATATTTATAGTATATATTATATTTTTATTATATAAATATG 78604

Gorilla NNNNNNNNNNNNNNNNNNNNNNNNNNNNNNNNNNNNNNNNNNNNNNNNNNNNNNNNNNNN 69669

Human TTTCATATGATAGATAATATGAGTATTGTATTTATATTCTCCATTTATAGAGAATATAAA 69765

Chimpanzee TTTCATATGATAGATAATATGAGTATTGTATTTATATTCTCCATTTATAGAGAATATAAA 78664

Gorilla NNNNNNNNNNNNNNNNNNNNNNNNNNNNNNNNNNNNNNNNNNNNNNNNNNNNNNNNNNNN 69729

Human TATTATCTATTATTTATATAATAGAAATATAATTATAGTAATAGATGTTTATATTATAGA 69825

Chimpanzee TATTATCTATTATTTATATAATAGAAATATAATTATAGTAATAGATGTTTATATTATAGA 78724

Gorilla NNNNNNNNNNNNNNNNNNNNNNNNNNNNNNNNNNNNNNNNNNNNNNNNNNNNNNNNNNNN 69789

Human GACCATATAATTACCGTTCAAGGAACTGAGACTACAGGGGTGAACTACCAAGCCTGGTTA 69885

Chimpanzee GACCATATAATTACCGTTCAAGGAACTGAGACTACAGGGGTGAACTACCAAGCCTGGTTA 78784

Gorilla NNNNNNNNNNNNNNNNNNNNNNNNNNNNNNNNNNNNNNNNNNNNNNNNNNNNNNNNNNNN 69849

Human ATTAAAATTATTGTTATTATTTATATCTATACATAAATAATATAAATATAATAGATATAC 69945

Chimpanzee ATTAAAATTATTGTTATTATTTATATCTATACAAAAATAATATAAATATCATAGATATAC 78844

Gorilla NNNNNNNNNNNNNNNNNNNNNNNNNNNNNNNNNNNNNNNNNNNNNNNNNNNNNNNNNNNN 69909

Human ATAAAAATAGAACTATATTTATAAATAATATAAATATATTCATATTTATATTAGCTATAT 70005

Chimpanzee ATAAAAATAGAACTATATTTATAAATAATATAAATATATTCATATTTATATTAGCTATAT 78904

Gorilla NNNNNNNNNNNNNNNNNNNNNNNNNNNNNNNNNNNNNNNNNNNNNNNNNNNNNNNNNNNN 69969

Human TTATAGATAATATGTATACACA--CATCAATAATTAAGAAAATAATTATTTTAGTAATAT 70063

Chimpanzee TTATAGATAATATGTATACACATCCATCAATAATTAAGACAATAATTATTTTAGTAATAT 78964

Gorilla NNNNNNNNNNNNNNNNNNNNNN--NNNNNNNNNNNNNNNNNNNNNNNNNNNNNNNNNNNN 70027

Human ACTAATACTATAAAAGTAATAGTCTTGCAAATAATTGCTTATGTATTTGGAACCTTATTT 70123

Chimpanzee ACTAATACTATAAAAGTAATAGTCTTGCAAATAATTGCTTATGTATTTGGAACCTTATTT 79024

Gorilla NNNNNNNNNNNNNNNNNNNNNNNNNNNNNNNNNNNNNNNNNNGTATTNNNNNNNNNANNN 70087

***** *

Human AATCTTCTTAGCTTTCATTTGAAAACATGTTTGTCTGTGTCCTGTGAGCGTGTTTTCCTA 70183

Chimpanzee AATCTTCTTAGCTTTCATTTGAAAACATGTTTGTCTGTGTCCTGTGAGCGTGTTTTCCTA 79084

Gorilla NNNNNNNNNNNNNNNNNNNNNNNAACATGTTTGTCTGTGTCCTGTGAGCGTATTTTCCTA 70147

**************************** ********

Human TTCTATTTTTATGTACAGATCAACTCTGTCTCAGTCTGCTTTGCATTGCTATAAAGAAAT 70243

Chimpanzee TTCTATTTTTATGTACAGATCAACTCTGTCTCAGTCTGCTTTGCATTGCTATAAAGAAAT 79144

Gorilla TTCTATTTTTATGTACAGATCAACTCTGTCTCAGTCTGCTTTGCATTGCTATAAAGAAAT 70207

************************************************************

Human ACCTGAGCCTGGGTAATTTACCAATTTATAAAGCCTGGTGTTTACCTGAGTCACGATTTT 70303

Chimpanzee ACCTGAGCCTGGGTAATTTACCAATTTATAAAGCCTGGTGTTTACCTGAGTCACGATTTT 79204

Gorilla ACCTGAGCCTGGGTAATTTACCCATTNNNAAAGCCNNNNNNTTACCTNNNNNNNNNNNNN 70267

********************** *** ****** ******

Human TCAGGCTGTATAAGAAGACTTTTCTTCTTGTAAACACCTCAGGATGCTTCCAGTAATGGC 70363

Chimpanzee TCAGGCTGTATAAGAAGACTTTTCCTCTTGTAAACACCTCAGGATGCTTCCAGTAATGGC 79264

Gorilla NNAGGNNNNNTAAGAAGACTTTTCTTCTTGTAAACACCTCAGGATGCTTCCAGTAATGGC 70327

*** ************** ***********************************

Human AAATGTGAAAGGTAAATGGAGAGTCATATGCAAGGGAGGAAAAGACAGAGGAAGAGGGGT 70423

Chimpanzee AAATGTGAAAGGTAAATGGAGAGTCATATGCAAGGGAAGAAAAGACAGAGGAAGAGGGGT 79324

Gorilla AAATGTGAAAGGTAAATGGAGAGTCATATGCAAGGGAGGAAAAGACAGAGGAAGAGGGGT 70387

************************************* **********************

Human GCCATGCTCAGATGAACTATACAGTAGGGACTCACTCCTTCCAAAGGGCACCAAGTCATT 70483

Chimpanzee GCCATGCTCAGATGAACTATACAGTAGGGACTCACTCCTTCCAAAGGGCACCAAGTCATT 79384

Gorilla GCCATGCTCAGATGAACTATACAGTAGGGACTCACTCCTTGCAAAGGGCACCAAGTCATT 70447

**************************************** *******************

Human CATGAGGAATCCACCCCCATGACTCAAACACCTCTCGCCAGGCCCCACATACAACGTGGG 70543

Chimpanzee CATGAGGAATCCACCCTCATGACTCAAACACCTCTCGCCAGGCCCCACATACAACGTGGG 79444

Gorilla CATGAGGAATCCACCCCCATGACGCAAACACCTCTCACCGNNNNNNNNNNNNNNNGTGGG 70507

**************** ****** ************ ** *****

Human GATAAAATTTCAATATGAGTTATGGCGGATCCAATATCCAAACTCTACCGATGCCTAACT 70603

Chimpanzee GATAAAATTTCAATATGAGTTATGGCGGATCCAATATCCAAACTCTACCGATGCCTAACT 79504

Gorilla GATAANNNNNNNNNNNNAGTTACGGCGGATCCAATATCCAAACTCTANNNNNGNNNNNNN 70567

***** ***** ************************ *

Human CAGTCTTGTAGAAGAGCAATTGTTTTATTATGCTAAAGATTTTGTGCTTTAGGGATTCTG 70663

Chimpanzee CAGTCTTGTAGAACAGCAATTGTTTTATTATGCTAAAGATTTTGTGCTTTAGGGATTCTG 79564

Gorilla NNNNNTTGTAGAACAGCAATTGTTTTATTATGCTAAAGATTTTGTGCTTTAGGGATTCTG 70627

******** **********************************************

Human AAAGGGTTCGTTGTATATGGCTGTCTTTGCCTGTGGTGTTTGGAGTTTACAAAGTCGGGG 70723

Chimpanzee AAAGGGTTCGTTGTATATGGCTGTCTTTGCCTGTGGTGTTTGGAGTTTACAAAGTTGGGG 79624

Gorilla AAAGGGTTCGTTGTATATGGCTGTCTTTGCCTGTGGTGTTTGGAGTTTACAAAGTTGGGG 70687

******************************************************* ****

Human AGGCACTCTGTGGCTGAGGAATAGAATGAAGTGAAGGTTTCATCACTCCTATGTGTGTGG 70783

Chimpanzee AGGCACTCTGTGGCTGAGGAATAGAATGAAGTGAAGGTTTCATCACTCCTATGTGTGTGG 79684

Gorilla AGGCACTCTGTGGCTGAGGAATAGAATGAAGTGAAGGTTTCATCACTCCTNNNNNNNNNN 70747

**************************************************

Human ATGGATGCTGGCTCAGTAAGAGCTCTCAGATGAAATGTACAAAGCACCTCCCCGAAGTGG 70843

Chimpanzee ATGGATGCTGGCTCAGCAAGAGCTCTCAGATGAAATGTACAAAGCACCTCCCCGAAGTGG 79744

Gorilla ATGGATGCTGGCTCAGTAAGAGCTCTCAGATGAAATGTACAAAGCACCTCCCCGAAGTGG 70807

**************** *******************************************

Human CTTAGGCTTCATTTACAATCCAGCCTCAGAATGTACATAGTGTCACTTCCTCTGTTTTCA 70903

Chimpanzee CTTAGGCTTCATTTACAATCCAGCCTCAGAATGTACATAGTGTCACTTCCTCTGTTTTCA 79804

Gorilla CTTAGGCTTCATTTACAATCCAGCCTCAGACTGTACATAGTGTCACTTCCTCTGTTTTCA 70867

****************************** *****************************

Human CAGCCTTGCTCAGATTCCAGAGGAGAGAGCGTCTTTTTATTTATTTATGTATTTATTTAT 70963

Chimpanzee CAGCCTTGCTCAGATTCCAGAGGAGAGAGCGTCTTTTTATTTATTTATGTATTTATTTAT 79864

Gorilla CATCCTTGCTCAGATTCCAGNNNAGAGAGCNNNNNNNNNNNNNNNNNNNNNNNNNNNNNN 70927

** ***************** *******

Human TTACTTATTTATTCAATTAATTTATTTTTGAGACATGGTCTTGCTCTGTCTCCTAGACTG 71023

Chimpanzee TTACTTATTTATTCAATTAATTTATTTTTGAGACATGGTCTTGCTCTGTCTCCTAGACTG 79924

Gorilla NNNNNNNNNNNNNNNNNNNNNNNNNNNNNNNNNNNNNNNNNNNNNNNNNNNNNNNNNNNN 70987

Human GAGTGCAGTGGTTCTATCATGGCTCACTGCAGACTCAACTTCCTGGACTGAAGTGATCCT 71083

Chimpanzee GAGTGCAGTGGTTCTATCATGGCTCACTGCAGACTCAACTTCCTGGACTGAAGTGATCCT 79984

Gorilla NNNNNNNNNNNNNNNNNNNNNNNNNNNNNNNNNNNNNNNNNNNNNNNNNNNNNNNNNNCT 71047

**

Human CCTGCCTGAGCCTCCCAAGTAACTGGGACTACAGAGGTGAACTGCCATGCATGGTTATAT 71143

Chimpanzee CCTGCCTGAGCCTCCCAAGTAACTGGGACTACAGAGGTGAACTGCCATGCATGGTTATAT 80044

Gorilla CCTGCCTGAGCCTCCCAAGTAACTGGGACTACAGAGGTGAACTGCCATGCATGGTTATAT 71107

************************************************************

Human TTTAAAATAATAATAGTTATTATTATTACATTGTAGAAAAAGGGTCTCACTATGTTGCTG 71203

Chimpanzee TTTAAAATAATAATAGTTATTATTATTACATTGTAGAAAAAGGGTGTCACTATGTTGCTG 80104

Gorilla TTTAAAATAATAATAGTTATTATTATTACATTGTAGAAAAAGGGTCTCACTATGTTGCTG 71167

********************************************* **************

Human GTCTCCAATTCCCGGCCTCAACAATCCTCCCACCCTGGCCTCCAAAATGCTATTATTTCA 71263

Chimpanzee GTCTCCAATTCCTGGCCTCAACAATCCTCCCACCCTGGCCTCCAAAATGCTATTATTTCA 80164

Gorilla GTCTCCAGTTCCCGGCCTCAACAATCCTCCCACCCTGGCCTCCCAAATGCTATTATTTCA 71227

******* **** ****************************** ****************

Human GGCTTGAGCACACACACCTGCCAGGAGAATCTTTTGTCTGAGAAGAATCTTAAAGCTTTA 71323

Chimpanzee GGCTTGAGCACACACACCTGCCAGGAGAATCTTTTGTCTGAGAAGAATCTTAAAGCTTTA 80224

Gorilla GGCTTGAGCACACACACCTGCCAGGAGAATCTTTTGTCTGAGAAGAATCTTAAAGTTTTA 71287

******************************************************* ****

Human AAGTTGCTCTATATGAAGGGTCTGTGGAATGGAAGAAATGCTCTGAACACCTTTGGTAAA 71383

Chimpanzee AAGTTGCTCTATATGAAGGGTCTGTGGAATGGAAGAAATGCTCTGAACACCTTTGGTAAA 80284

Gorilla AAGTTGCTCTATGTGAAGGGGCTGTGGAATGGAAGAAATGCTCTGAACACCTTTGGTAAA 71347

************ ******* ***************************************

Human TAAAATTGATCACAAGGAATATGTTTGTGAAGTTTTGAAAAATGAGTTTATTTGAGACAT 71443

Chimpanzee TAAAATTGATCACAAGGAATATGTTTGTGAAGTTTTGAAAAATGAGTTTATTTGAGACAT 80344

Gorilla TAAAATTGATCACAAGGAATATGTTTGTGAAGTTTTGAAAAATGAGTTTATTTGAGACAT 71407

************************************************************

Human GCATGTGATTCTAACATTCTGATTTCTTTCAGCTCCACTTGCAAATTTTTCTGTTCAGAA 71503

Chimpanzee GCATGTGATTCTAACATTCTGATTTCTTTCAGCTCCACTTGCAAATTTTTCTGTTCAGAA 80404

Gorilla GCATGTGATTCTAACATTCTGATTTCTTTCAGCTCCACTTGCAAATTTTTCTGTTCAGAA 71467

************************************************************

Human TAAAAGTAGGTAATGATAGATAATCTATAACACTATAAAATAGTGGTAATTTTTCCCATC 71563

Chimpanzee TAAAAGTAGGTAATGATAGATAATCTATAACACTATAAAATAGTGGTAATTTTTCCCATG 80464

Gorilla TAAAAGTAGGTAATGATAGATAATCTATAACACTGTAAAATAGTGGTAATTTTTCCCATC 71527

********************************** ************************

Human ATGATGTTAAAATTTCCTCAAGTAAAGGATAGTTTCATCAAAATAATGGCCCTATCATTT 71623

Chimpanzee ATGATGTTAAAATTTCCTCAAGTAAAGGATAGTTTCATCAAAACAGTGGCCCTATCATTT 80524

Gorilla ATGATGTTAAAATTTCCTCAAGTAAAGGATAGTTTCATCAAAATAGTGGCCCTATCATTT 71587

******************************************* * **************

Human ATTCTGTATTATTACATAGCCCTTAGTTGAAACTTACATGGAAGAAATTTCTCCATTCCA 71683

Chimpanzee ATTCTGTATTATTACATAGCCCTTAGTTGAAACTTACATGGAAGAAATTTCTCCATTCCA 80584

Gorilla ATTCTGTATTATTACATAGCCCTTAGTTGAAACTTACATGGAAGAAATTTCTCCATTCCA 71647

************************************************************

Human ATCACATTCTTGTCCTAGCAGGCTACTGAAAACCTTTGTTTCAAACTGAGAAATCAATCT 71743

Chimpanzee ATCACATTCTTGTCCTAGCAGGCTACTGAAAACCTTTGTTTCAAACTGAGAAATCAATCT 80644

Gorilla ATCACATTCTTGTCCTAGCAGGCTACTGAAAACCTTTGTTTCAAACTGAGAAATCAATCT 71707

************************************************************

Human TAAAAATATATATAAAATAAGCATAAAATGTTAAACCAGAAAAAATGACCAACGGCTTTT 71803

Chimpanzee TAAAAAAA-ATATAAAATAAGCATAAAATGTTAAACCAGAAAAAATGACCAACGGCTTTT 80703

Gorilla TAAAAATATATATAAAATAAGCATAAAATGTTAAACCAGAAAAAATGACCAACGGCTTTT 71767

****** * ***************************************************

Human TCTTAATGAAAAATTAAGAAAACTTAGGCAATAAACACTGATGTTTGTTTTTGAGATAAG 71863

Chimpanzee TCTTAATGAAAAATTAAGAAAACTTAGGCAATAAACACTTATGTTTGTTTTTGAGACAAG 80763

Gorilla TCTTAATGAAAAATTAAGAAAACTTNNNNNNNNNNNNNNNNNNTTTGTTTTTGAGACAAG 71827

************************* ************* ***

Human ATTTCACTCTATGACCCAGGCTGGAGTGCAAATGGGATGATTATGGTTCACTACAGCCTT 71923

Chimpanzee ATTTCACTCTATGACCCAGGCTGGAGTGCAAATGGGATGATTATGGTTCACTACAGCCTT 80823

Gorilla ATTTCACTCTATGACCCAGGCTGGAGTGCAAATGGGATGATTATGGTTCACTACAGCCTT 71887

************************************************************

Human TTTCTTTGGAGATCAGCAATCATCACACTTCAGCCTCCCAAACAGCTAGAACCACAGGCA 71983

Chimpanzee TTTCTTTGGAGATCAGCAATCATCACACTTCAGCCTCCCAAACAGCTAGAACCACAGGCA 80883

Gorilla TTTCTTTGGAGNNNNNNNNNCATCACACTTCAGCCTCCCAAATAGCTAGAACCGCAGGCA 71947

*********** ********************** ********** ******

Human TGTGCCACCACACCCAGCTAATTTTCAAAAATAAATTATTTTTAGAGATGAAGTGTCACT 72043

Chimpanzee TGTGCCACCACGCCCAGATAATTTTCAAAAATAAATTATTTTTAGAGATGAAGTGTCACT 80943

Gorilla TGTGCCACCACGCCCAGCTAATTTTCAAAAATAAATTATTTTTAGAGATGAAGTGTCACT 72007

*********** ***** ******************************************

Human ATGTTGTCTAGGCTGTTCTCAAATGCCTGGCCGCAAGCGACCCTCCCACCTTGATTTTCT 72103

Chimpanzee ATGTTGTCTAGGCTGTTCTCAAATGCCTGGCCTCAAGCGACCCTCCCACCTTGATTTTGT 81003

Gorilla ATGTTGTCTAGGCTGTTCTCAAATGCCTGGCCTCAAGCGACCCTCCCACCTTGATTTTCT 72067

******************************** ************************* *

Human AAATTGTTGGGATTACAGGTGTGACCCAACATGCTTGACCAAAACTAATTTTAATATTAT 72163

Chimpanzee AAATTGTTGGGATTACAGGTGTGACCCAACATGCTTGACCAAAACTAATTTTAATATTAT 81063

Gorilla AAATTGTTGGGATTACAGGTGTGACCCACCATGCTTGGCCAAAACTAATTTTAATATTAT 72127

**************************** ******** **********************

Human CACCCTGCTTTTTTTTTTTTTTTTTTTTTTTTTCTGTAGGAAACTTTTGCCTAACCTAGA 72223

Chimpanzee CACCCTGCTTTTTTTTTTTTTTTTTTT------CTGTAGGAAACTTTTGCCTAACCTAGA 81117

Gorilla CACCCTGCTTTTTTTTTTTTTTTTTTTTTTTTTNNNNAGGAAACTTTTGCCTAACCTAGA 72187

*************************** ***********************

Human AATAGCATCAACATTTCTTAAAAAAAAAAAAA--------TCAAGCTATATCCTTGTCAA 72275

Chimpanzee AACAGCATCAACATTTCTTAAAAAAAAAAAAAAAAAAAAATCAAGCTATACCCTTGTCAA 81177

Gorilla AACAGCATCAACATTTCTTAAAAAAAAAA-----------TCAAGCTATACCCTTGTCAA 72236

** ************************** ********** *********

Human TATGGGCAGATATACACTAATGATCTAGACCAAGAGGTCACTTATTAAAGAATATCCTAT 72335

Chimpanzee TATGGGCAGATATACACTAATGATCTAGACCAAGAGGTCACTTATTAAAGAATATCCTAT 81237

Gorilla TATGGGCAGATATACACTAATGATCTAGACCAAGAGGCCACTTATTAAAGAATATCCTAT 72296

************************************* **********************

Human TATTGGGAAAAAAGAAACTTTAAGGTAACATGGATAAGAATCCCATTCGGAAACACATTT 72395

Chimpanzee TATTGGGAAAAAAGAAACTTTAAGGTAACATGGACAAGAATCCCATTCGGAAACACATTT 81297

Gorilla TATTGGGAAAAAAGAAACTTTAAGGTAACATGGACAAGAATCCCATTCGGGAACACATTT 72356

********************************** *************** *********

Human TGAAGTTTGATGGTATTTAAGCGCACGATTAACCACATTATACATAGCGGAAGCCAAAGT 72455

Chimpanzee TGAAGTTTGATGGTATTTAAGCGCACGGTTAACCACATTATACATAGCGGAAGCCAAAGT 81357

Gorilla TGAAGTTTGATGGTATTTAAGCGCACGATTAACCACATTATACATAGCGGAAGCCAAAGT 72416

*************************** ********************************

Human TCCTAGTATGGAAAGAATGGAGATACAAGTATGTCCCATTATAATGAGGAAAGGCCCAAG 72515

Chimpanzee TCCTAGTATGGAAAGAATGGAGATACAAGTATGTCCCATTATAACGAGGAAAGGCCCAAG 81417

Gorilla TCCTAGTATGGAAAGAATGGAGATACAAGTATGTCCCATTATAATGAGGAAAGGCCCAAG 72476

******************************************** ***************

Human ACAAGGAAACAGTGTGCAAGGTACAAGAAAAGTGTGATTTTTCACACCCATCAGATGCTG 72575

Chimpanzee ACAAGGAAACAGTGTGCAAGGTACAAGAAAAGTGTGATTTTTCACACCCATCAGATGCTG 81477

Gorilla ACAAGGAAACAGTGTGCAAGGTTCAAGAAAAGTGTAATTTTTCACACCCATCAGATGCTG 72536

********************** ************ ************************

Human ACATTCTTGGAGTGATACAGAGAACAGGAAAAGCACATGATGGATTAGGCTAAATCCATT 72635

Chimpanzee ACATTCTTGGAGTGATGCAGAGAACAGGAAAAGCACATGATGGATTAGGCTAAATCCATT 81537

Gorilla ACATTCTTGGAGTGATACAGAGAACAGGAAAAGCACATGATGGATTAGGCTAAATCCGTT 72596

**************** **************************************** **

Human TTCTCCTTTCTAGCCTTAATTATTTTTGCTAAAATCAAACTCTGTGATGTCAGTCTCTCC 72695

Chimpanzee TTCTCCTTTCTAGCCTTAATTATTTTTGCTAAAATCAAACTCTGTGATGTCAGTCTCTCC 81597

Gorilla TTCTCCTTTCTAGCCTTAATTATTTTTGCTAAAATCAAACTCTGGGATGTCAGTCTCTCC 72656

******************************************** ***************

Human TAATTCTACCTGGGAAATGCTGTTCATGCATTTAGCACCCAGTTAAGGGACAATGTGGTG 72755

Chimpanzee TAATTCTACCTGGGAAATGCTGTTCATGCATTTAGCATCCAGTTAAGGGACAATGTGGTG 81657

Gorilla TAATTCTACCTGGGAAATGCTGTTCATGCATTTAGCATCCAGTTAAGAGACAATGTGGTG 72716

************************************* ********* ************

Human GTTGGGGATGTTGCCCAAGGTTGGCACCCAGATATCAAAATCCATATTGTACCTGGATTC 72815

Chimpanzee GTTGGGGATGTTGCCCAAGGTTGGCACCCAGATATCAAAATCCATATTGTACCTGGATTC 81717

Gorilla GTTAGGGATGTTGCCCAAGGTTGGCACACAGATATCAAAATCCATATTGTACCTGGATTC 72776

*** *********************** ********************************

Human CTGGTTGATGTTTTAACATTTCTTCTCACTTTAGTGTCCTCATCTATAGATCAACCCAGC 72875

Chimpanzee CTGGTTGATGTTTTAACATTTCTTCTCACTTTAGTGTCTTCATCTATAGATCAACCCAGC 81777

Gorilla CTGGTTGATGTTTTAACATTTCTTCTCACTTTAGTGTCCTCATCTATAGATCAACCCAGC 72836

************************************** *********************

Human CAAATTTCTCCTCCATCAGCTTGCTGTGGTAATTTTTTTTTT----AAAAAAATATTTAT 72931

Chimpanzee CAAATTTCTCCTCCATCAGCTTGCTGTGGTAATTTTTTTT------AAAAAAATATTTAT 81831

Gorilla CAAATTTCTCCTCCATCAGCTTGCTGTGGTAATTTTTTTT-------AAAAANNNNNTAT 72889

**************************************** ***** ***

Human TTATTTATTTTATTTTTAGAGACAATTTCTCATTCTGTTGCCCAGGATGGATTGTAGTGT 72991

Chimpanzee TTAT----TTTATTTTTAGAGACAATTTCTCATTCTGTTGCCCAGGATGGATTGTAGTGT 81887

Gorilla TTATTTNNNNTATTTTTAGAGACAATTTCTCATTCTGTTGCCCAGGATGGATTGTAGTGT 72949

**** **************************************************

Human CCCAGATTTCACTGCAGCCTTGACATCCTGGGCTCAAGAAATCCTCCCATGTCAACACCC 73051

Chimpanzee CCCAGATTTCACTTCAGCCTTGACATCCTGGGCTCAAGAAATCCTCCCATGTCAACACCC 81947

Gorilla CCTAGATTTCACTGCAGCCTTGACATCCTGGGCTCAGGCAATCCTCCCATGTCAACACCC 73009

** ********** ********************** * *********************

Human CCAGAGTAGCTGAGTCTACAGACATACACCGTCATGCACGGCTAATT-----TGTGTGTG 73106

Chimpanzee CCAGAGTAGCTGAGTCTACAGACATACACCATCATGCATGGCTAATTGTGTGTGTGTGTG 82007

Gorilla CCAGAGTAGCTGAGTCTACAGACATACACCATCATGCATGGCTAATT-----TGTGTGTG 73064

****************************** ******* ******** ********

Human TGTGTGTGTGTGTGTGTGTAAGACAGGTGTTTGTTATGTTGCCCAGGAGGGTCTCAGATT 73166

Chimpanzee TGTGTGTGTGTGTGTGTGTAAGACAGGTGTTTGTTATGTTGCCCAGGAGGGTCTCAGATT 82067

Gorilla TGTGTGTGTGTGTGTGTGTAAGACAGGTGTTTGTTATGTTGCCCAGGAGGGTCTCAAATT 73124

******************************************************** ***

Human CTTGGCCTCAAATGATCCCGCCTCCTTTCTCTACCAAAGCAGTGGGATTACAGACATAAG 73226

Chimpanzee CTTGGCCTCAAATGATCCCGCCTCCTTTCTCTACCAAAGCAGTGGGACTACAGGCATAAG 82127

Gorilla CTTGGCCTCAAATGATCCCGCCTCCTTTCTCTACCAAAGCAGTGGGATTACAGGCATAAG 73184

*********************************************** ***** ******

Human CCACTGCATCTGGCCAGTTTATAGTGATGATTAATCTCCTAACTGATGCCTTAAACTGCC 73286

Chimpanzee CCACTGCATCTGGCCAGTTTATAGTGATGATGAATCTCCTAACTGATGCCTTAAACTGCC 82187

Gorilla CCACTGCATCTGGCCAGTTTATAGTGATGATTAATCTCCTAACTGATGCCTTAAACTGCC 73244

******************************* ****************************

Human TGACACATAGTAAACCTTCCGGTCATGTTGCTTATTATTACTCCTATAATTATTTGTATT 73346

Chimpanzee TGACACATAGTAAACCTTCCGGTCATGTTGCTTATTATTACTCCTATAATTATTTGTATT 82247

Gorilla TGACACATAGTTAACCTTCCGGTCATGTTGCTTATTATTACTCCTATAATCATTTGTATT 73304

*********** ************************************** *********

Human GTTATTTATGCCATTAATTTTTCTTTTACTTGAAGTCTTTCTCTTCAATTACAAAATAAA 73406

Chimpanzee GTTATTTATGCCATTAATTTTTCTTCTACTTGAAGTCTTTCTCTTCAATTACAAAATAAA 82307

Gorilla GTTATTTATGCCATTAATTTTTCTTCTACTTGAAGTCTTTCTCTTCAATTACAAAATAAA 73364

************************* **********************************

Human TGTCATTTCATGTTAAAGAATTTCAAAGCATTGTCGATATGACATATGATTTTCTCAGTG 73466

Chimpanzee TGTCATTTCATGTTAAAGAATTTCAAAGCATTGTCGATATGACATATGATTTTCTCAGTG 82367

Gorilla TGTCATTTNATGTTAAAGAATTTCAAAGCACTGTCGATATGACATATGATTTTCTCAATG 73424

******** ********************* ************************** **

Human GGTATCAAAGAAATAATACATACTTATATAATCATATTTAACAATCTATTACTATAAAAT 73526

Chimpanzee GGTATCAAAGAAATAGTATATACTTATATAATCATATTTAACAATCTATTACTATAAAAT 82427

Gorilla GGTATCAAAGAAATAATATATACTTATATAATCATATTTAACAATCTATTACTATAAAAT 73484

*************** ** *****************************************

Human AGTTGTTTCAGAATTCTGAAAAAGTTATCTTTAACTAGAGGAAAATTTTAGAGAAATGGT 73586

Chimpanzee AGTTGTTTCAAAATTCTGAAAAAGTTATCTTTAACTAGAGGAAAATTTTAGAGAAATGGT 82487

Gorilla AGTTGTTTTAGAATTCTGAAAAAGTTATCTTTAACTAGAGGAAAATTTTATAGAAATGGT 73544

******** * *************************************** *********

Human GAAATTAAGTTGGGCTGTATAAAAGAAAATTAATATGTTTTTAGTAAACTAATATAAAAA 73646

Chimpanzee GAAATTAAGTTGGGCTGTATAAAAGAAAATTAATATGTTTTTAGTAAACTAATATAAAAA 82547

Gorilla GAAATTAAGTTGGGCTGTATAAAAGAAAATTAATATGTTTTTAGTAAACTAATATAAAAA 73604

************************************************************

Human TTCCAACTGACAATGTTTAAAATACTAAATGAATGTTGCATATAACTATAACACCTAAAA 73706

Chimpanzee TTCCAACTGACAATGTTTAAAATACTAAATGAATTTTGCATATAACTATAACACCTAAAG 82607

Gorilla TTCCAACGGACAATGTTTAAAATACTAAATGAATTTTGCATATAACNNNNNNACCTAAAA 73664

******* ************************** *********** *******

Human CATACATAACATGTTTAATGGTGATTGTAAAATGTTATGATTTTCCAAGTGATTGGCATT 73766

Chimpanzee CATATACAACATGTTTAATGGTGATTGTAAAATGTTATGATTTTCCAAGTGATTGGCATT 82667

Gorilla CATACATAACATGTTTAATGGTGATTGTAAAATGTTATGATTTTCCAAGTGATTGGCATT 73724

**** * *****************************************************

Human AAAATGTTTCACCGTTAAAGTCATTCATTTATGTAGCCCTCATATTCAGGGATTGAAAAT 73826

Chimpanzee AAAATGTTTCACCGTTAAAGTCATTCATTTATGTAGCCCTCATATTCAGGGATTGAAAAT 82727

Gorilla AAAATGTTTCACCGTTAAAGTCATTCATTTATGTAGCCCTCATGTTCAGGGATTGAAAAT 73784

******************************************* ****************

Human AATTAGCTACAATGCAAAAACGTGAATCAATTTTATTTTCTTTCATTAAATTTAGGTAGA 73886

Chimpanzee AATTAGCTACAATGCAAAAACGTGAATCAATTTTATTTTCTTTCATTAAATTTAGGTAGA 82787

Gorilla AATTAGCTACAGTGCAAAAACGTGAATCAATTTTATTTTACTTCATTAAATTTAGGTAGA 73844

*********** *************************** *******************

Human ACAGGAGACAGGACTGTTGTTATTAATGAGAATACTACTACTGAACTGGTACAATTGTAG 73946

Chimpanzee ACAGGAGACAGGACTGTTGTTATTAATGAGAATACTACTACTGAACTGGTACAATTGTAG 82847

Gorilla ACAGGAGACAGGACTGTTGTTATTAATGAGAATACTACTACGGAACTGGTACGATTGTAG 73904

***************************************** ********** *******

Human ACAAAAAGCCCAGGGAATTTGTGGTATTGCTAGCTTCAGGAAAATTTTTAAACATCATTT 74006

Chimpanzee ACAAAAAGCCCAGGGAATTTGTGGTATTGCTAGCTTCAGGAAAATTTTTAAACATAATTT 82907

Gorilla ACAAAAAGCGCAGGAAATTTGTGGTATTGCTAGCTTCAGGAAAATTTTTAAAAATCATTT 73964

********* **** ************************************* ** ****

Human AGGACTGTTAAGTCCATATGTAAAATGCTATACCTCTTCTTTCAAACCATCACAAAAAGT 74066

Chimpanzee AGGACTGTTAAGTCCATACGTAAAATGCTATACCTCTTCTTTCAAACCATCACAAAAAGT 82967

Gorilla AGGACTGTTAAGTCCATATGTAAAATGCTATACCTCTTCTTTCAAACCATCACAAAAAGT 74024

****************** *****************************************

Human ACACCATGAAGTCTAATGTTGAAGGCAAGTACATTTGGGATAGGTGTGTTCTCGGTGTGC 74126

Chimpanzee ACACCATGAAGTCTAATGTTGAAGGCAAGTACATTTGGGATAGGTGTGTTCTCAGTGTGC 83027

Gorilla ACACCATGAAGTCTAATGTTGAAGGCAAGTACATTTGGGATAGGTGTGTTCTCGGTGTGC 74084

***************************************************** ******

Human TATTTGAAATACAAGTAACATCAATGTTAGCAGAAGCAGTAACCTTTCATTTCTTTGGTG 74186

Chimpanzee TATTTGAAATACAAGTAACATCAATGTTAGCAGAAGCAGTAACCTTTCATTTCTTTGGTG 83087

Gorilla TATTTGAAATACGAGTAACATCAATGTTAGCAGAAGCAGTAACCTTTCATTTCTTTGGTG 74144

************ ***********************************************

Human TTCAATTCTACCACTATCACTCTTTTTTGCGTCACTACACATGGAGGGTGTATTAGTTCA 74246

Chimpanzee TTCAGTTCTACCACTATCACTCTTTTTTGTGTCACTACACATGGAGGGTGTATTAGTTCA 83147

Gorilla TTCAATTCTACCACTATCACTCTTTTTTGTGTCACTACACATGGAGGGTGTATTAGTTCA 74204

**** ************************ ******************************

Human TTCTTATGCTGCTATAACGAGATGCCTGAGACTGGGTAATTTATAACGGAAAGGTTTAAT 74306

Chimpanzee TTCTTATGCTGCTATAACGAGATGCCTGAGACTGGGTAATTTATAACAGAAAGGTTTAAT 83207

Gorilla TTCTTATGCTGCTATAACGAAATGCCTGAGACTGGGTAATTTATAACGGAAAGGTTTAAT 74264

******************** ************************** ************

Human TCACTCACTATTCTGCATGGATGGAGAGGCCTCAGGAAACTTACAGTACTGGTGGAAGGC 74366

Chimpanzee TCACTCACTATTCTGCATGGATGGAGAGGCCTCAGGAAACTTACAGTACTGGTGGAAGGC 83267

Gorilla TCACTCACTATTCCGCATGGATGGAGAGGCCTCAGGAAACTTACAGTACTGGTGGAAGGC 74324

************* **********************************************

Human AAAGGAGAAGCAAATACCTTCTTCACAAAGTGTCAGGCTGAGGCAGGAGAATTGCTTGAA 74426

Chimpanzee AAAGGAGAAGCAAATACCTTCTTCACAAAGTGTCAGGCCGAGGCAGGAGAATTGCTTGAA 83327

Gorilla AAAGGAGAAGAAAATACCTTCTTCACAAAGTGTCAGGCTGAGGCAGGAGAATTGCTTGAA 74384

********** *************************** *********************

Human CCCAGGAGGTGGAGGTTGCAGTGAGCCAAGATCATGCCACTGCACTCCAACATGAGCAAC 74486

Chimpanzee CCCAGGAGGTAGAGGTTGCAGTGAGCCAAGATCATGCCACTGCACTCCAACATGAGCAAC 83387

Gorilla CCCAGGAGGTGGAGGTTGCAGTGAGCCAAGATGATGCCACTGCACTCCAACATGAGCAAC 74444

********** ********************* ***************************

Human ACAGTGATACGCCATCTCAAAAATAAATACATACATACATACATACATACATACATACAT 74546

Chimpanzee ACAGTGATACGCCATCTCAAAAATAAATACATACATACATACATACATACATACATACAT 83447

Gorilla ACAGTGATACGCCATCTCAAAAANNNNNNNNNNNNNNNNNAAATAAATAAACACATACAT 74504

*********************** * *** *** * ********

Human ACATAATAAGGAGGAACTGTCAAACACTTATAAAGCCATCAGATCTCCAGAGAACTCACT 74606

Chimpanzee A----ATAAGGAGGAACTGTCAAACACTTATAAAGCCATCAGATCTCCAGAGAACTCACT 83503

Gorilla CCATAATAAGGAGGAACTGTCAAACACTTATAAAGCCATCAGATCTCCAGAGAACTCACT 74564

*******************************************************

Human CACATCATGAGAATGGCATGGGGGAGCCTCCACCATGATACAATCACCTCCCACCAGTCC 74666

Chimpanzee CACATCATGAGAATGGCATGGGGGAGCCTCCACCATGATACAATCACCTCCCACCAGTCC 83563

Gorilla CACATCATGAGAATGGCATGGGGGAGCCTCCACCGTGATNNNNNNNNNNNNNNNNNNNNN 74624

********************************** ****

Human TTTCCTCAACATATGGGGATTATGAGAATTACAATCCCAGATGAGATTTGGGTGAGGACA 74726

Chimpanzee TTTCCTCAACATATGGGGATTATGAGAATTACAATCCCAGATGAGATTTGGGTGAGGACA 83623

Gorilla NNTCCTCAACATATGGGGATTATGAGAATTACAATCCCAGATGAGATTTGGGTGAGGACA 74684

**********************************************************

Human CAGAGCCAAACCATATCAAAGAATTAACTATGAGCAATGATGACCCTGATAGCTCATTAT 74786

Chimpanzee CAGAGCCAAACCATATCAAAGAATTGACTATAAGCAATGATGACCCTGATAGCTCATTAT 83683

Gorilla CAGAGCCAAACCATATCAAAGAATTAACTGTGAGCAATAATGACCCTGATAGCTCATTAT 74744

************************* *** * ****** *********************

Human TATTTAAAAAAAGAAAACACA---AAACTGATGCTTCTCTATACTTACTCCAGGACAATA 74843

Chimpanzee TATTTAAAAAAAAAACACACACAAAAACTGATGCTTCTCTATACTTACTCCAGGACAATA 83743

Gorilla TATTTAAAAAAANNNNNCACA---AAACTGATGCTTCTCTATACTTACTCCAGGACAATA 74801

************ **** ************************************

Human CAATTGTTTCACCTCCAGAGTTACAAGGCTATTTAATATGCATATATTCACATATTTAAT 74903

Chimpanzee CAATTGCTTCACCTCCAGAGTTATAAGGCTATTTAACATGCATATATTCACATATTTAAT 83803

Gorilla CCATTGTTTCACCTCCAGAGCTATAAGGCTATTTAATATGCATATATTCACATATTTAAT 74861

* **** ************* ** ************ ***********************

Human CATTTTTTTCACTTATGTGCATGCATAATGATGGGCACACACAGAGAGATAAATCTACCT 74963

Chimpanzee CATTTTTTTCACTTATGTGCATGCATAATGATGCGCACACACAGAGAGATAAATCTACCT 83863

Gorilla GATTTTTTTCACTTACATGCATGCATAATGATGCGCACACACAGAGAGATAAATCTACCT 74921

************** **************** **************************

Human TCATTTCTCATGCTACAAGTTCAAATCTGTATGAGAACTTTGATGCTATGTTTTTATGAC 75023

Chimpanzee TCATTTCTCATGCTACAAGTTCAAATCTGTATGAGAACTTTGATGCTATGTTTTTATGAC 83923

Gorilla TCATTTCTCATGCTACAAGTTCAAATCTATATGAGAACTTTGATGCTATGTTTTTATGAC 74981

**************************** *******************************

Human ATGAGTATTTGCTAATTCCTGTGTGTGTGTGTGTGTGTGTGTGTGTGTGTGTGTGTGTGT 75083

Chimpanzee ATGTGTATTTGCTAATTCCTGTGTGTGTGTGTGTGTGTGTGT------------------ 83965

Gorilla ATGTGTATTTGCTAATTCCNNNNNNNNNNNNTGTTTGTGTGTTTGTTTGTGTGTTTGTAT 75041

*** *************** *** *******

Human GTGTATTTATACATATGTGTGTTTTCCTGTGTCTTTTGGTGTGTTTCTTGTTATTCTTCT 75143

Chimpanzee GTGTATTTATACATATGTGTGTTTTCCTGTGTCTTTTGGTGTGTTTCTTGTTATTCTTCT 84025

Gorilla GTGTATTTATACATATGTGTGTTTTCCTGTGTCATTGGGTGTGTTTCTTGTTATTCTTCT 75101

********************************* ** ***********************

Human ACTTTTTAAATTCCAGCTTGTTCCAACATTATTCTCTTGAATCTAATGTGTCTGAGGTCC 75203

Chimpanzee GCTTTTTAAATTCCAGCTTGTTCCAACATTATTCTTTTGAATCTAATGTGTCTGAGGTCC 84085

Gorilla ACTTTTTTAATTCCAGCTTGTTCCAACATTATTCTTTTGAATCTAATGTGTCTGAGGTCC 75161

****** *************************** ************************

Human CTCTACAATCTGAAGTTAGAAATGGATATGCCCGCCTTAGGATAGTGAGATTTATTCAAG 75263

Chimpanzee CTCTAAAATCTGAAGTTAGAAATGGATATGCCTGCCTTAGGATAGTGAGATTTAGTCAAG 84145

Gorilla CTCTAAAATCTGAAGTTAGAAATGGATATGCCCGCCTTAGGATAGTGAGATTTATTCAAG 75221

***** ************************** ********************* *****

Human ATTTCCAAATTGCATAGCCAATGGGAAAAAGAAAAATACAATGACTCTTTTGCTGCTTGC 75323

Chimpanzee ATTTCCAAATTGCATAGCCAATGGGAAAAAGAAAAATACAATGACTCTTTTGCTGCTTGC 84205

Gorilla ATTTCCAAATTGCATAGCCAATGGGAAAAAGAAAAATACAGTGACTCTTTTGCTGCTTGC 75281

**************************************** *******************

Human CAACTTGACCTTCACAGAATTGAAGACTGCCTTCTTGGAAGCATTTCCTTTTCCATATGA 75383

Chimpanzee CAACTTGACCTTCACAGAATTGAAGACTGCCTTCTTGGAAGCATTTCCTTTTCCATATGA 84265

Gorilla CAACTTGACCTTCACAGAATTGAAGACTGCCTTCTTGGAAGCATTTCCTTTTCCATATGA 75341

************************************************************

Human AAAAGCTCTGCATTTCTCCCCAATTTCACAATAATTAAATAAACCTAGGCATCTTCTAGG 75443

Chimpanzee AAAAGCTCTGCATTTCTCCCCAGTTTCACAATAATTAAATAAACCTAGGCATCTTCTAGG 84325

Gorilla AAAAGCTCTGCATTTCTCCCCAATTTCACAATAATTAAATAAACCTAGGCATCTTCTAGG 75401

********************** *************************************

Human TCTATAAGGCACTTTGGGCAAAAGTTCCTAAGAAAGGGATTGTGCAATTGCTTCCCAGAA 75503

Chimpanzee TCTGTAAGGCACTTTGGGCAAAAGTTCCTAAGAAAGGGATTGTGCAATTGCTTCCCAGAA 84385

Gorilla TCTGTAAGGCACTTTGGGCAAAAGTTCCTAAGAAAGGGATTGTGCAATTGCTTCCCAAAA 75461

*** ***************************************************** **

Human GCCCTCGTCTAGGCAGAATAAATCAGCCAGCGCTCCTGTGATGAAAGAAACACAGAACAG 75563

Chimpanzee GACCACGTCTAGGCAGAATAAATCAGCCAGCTCTCCTGTGATGAAAGAAACACAGAACAG 84445

Gorilla GCCCTCGTCTAGGCAGAATAAATCAGCCAGCTCTCCTGTGATGAAAGAAACACACAACAG 75521

* ** ************************** ********************** *****

Human AGAAGAATGTGGGGTTTGATAATGTTAGTGCTAAGAATACCTCAAAACATTT-AAATACA 75622

Chimpanzee AGAAGAATGTGGGGTTTGATAATGTTAGTGCTAAGAATACCTCAAAACATTTTAAATACA 84505

Gorilla AGAAGAATGTGGGGTTTGATAATGTTAGTGCTAAGAATACCTCGAAACATTT-AAATACA 75580

******************************************* ******** *******

Human AATTCAGACCCCCAGAAAATGGTATCTTTCCCCTCCAGGAACCCCAAAGTAGTCTGATTC 75682

Chimpanzee AATTCAGACCCCCAGAAAATGGTATCTTTCCCCTCCAGGAACCCCAAAGTAGTCTCATTC 84565

Gorilla AATTCAGACCCCCAGAAAATGGTATCTTTCCCCTCCAGGAACCCCAAAGTAGTCTGATTC 75640

******************************************************* ****

Human ACCTCCTTTACTAAATCTAGTTCCATAAACTTGACCCCTTTAGCATCATCCAAAACATCT 75742

Chimpanzee ACCTCCTTTACTAAATCTAGTTCCATAAACTTGACCCCTTTAGCATCATCCAAAACATCT 84625

Gorilla ACCTCCTTTACTAAATCTAGTTCCATAAACTTGACCCCTTTAGCATCATCCAAAACATCT 75700

************************************************************

Human CCTAGTACTTCAATGTCTTAACCAGAAGGTATACAACTAATAGAATCGTAAAAATTACAA 75802

Chimpanzee CCCAGTACTTCAATGTCTTAACCAGAAGGTATACAACTAATAGAATCGTAAAAATTACAA 84685

Gorilla CCTAGTACTTCAATGTCTTAACCAGAAGGTATACAACTAATAGAATCGTAAAAATTACAA 75760

** *********************************************************

Human GGGTCCAACCCAACAGTAATGAGGCTGGTATTCAGGAAATCACACTAAAGAATGCTTGAT 75862

Chimpanzee GGGTCCAACCCAACAGTAATGAGGCTGGTATTCAGGAAATCGCACTAAAGAATGCTTGAT 84745

Gorilla GGGTCCAACCCAACAGTAATGAGGCTGGTATTCAGGAAATCACACTAAAGAATGCTTGAT 75820

***************************************** ******************

Human GGACTTGCTGGAATTTGTCAGGGAAACATTAACTATTGGAAGAATAATAAACCTTCCCAA 75922

Chimpanzee GGACTTGCTGGAATTTGTCAGGGAAACATTAACTATTGCAAGAATAATAAACCTTCCCAA 84805

Gorilla GGACTTGCTGGAATTTGTCAGGGAAACATTAACTATTGGAAGAATAATAAACCTTCCCAA 75880

************************************** *********************

Human ACTTAGCTGTGCTGGGGGGAATTGTTTTTATTATCCAGGAAAGCCAACAAATGTGGACAT 75982

Chimpanzee ACTTAGCTGTGCTGGGGGGAATTATTTTTATTATCCAGGAAAGCAAACAAATGTGGACAT 84865

Gorilla ACTTAGCTGTGCCGGGGGGAATTATTTTTATTATCCAGGAAAGCAAACAAATGTGGACAT 75940

************ ********** ******************** ***************

Human TATCTCTACCATCTAAGGCCACTCACTACACAAATATCCTGGAAAAACGTAGTACAGAAC 76042

Chimpanzee TATCTCTACCATCTAAGGCCACTCACTACACAAATATCCTGGAAAAACGTAGTGCAGAAC 84925

Gorilla TATCTCTACCATCTAAGGCCACTCACTACACAAATATCCTGGAAAAGTGTAGTACAGAAC 76000

********************************************** ***** ******

Human AAAAGCTCTTTCTTTATATTTTTGTGTTTTTTATTTGTTTCTTTGTTTGTCTGAGACAGA 76102

Chimpanzee AAAAGCTCTTTCTTTATATTTTTGTGTTTTTTATTTGTTTCTTTGTTTGTCTGAGACAGA 84985

Gorilla AAAAGCTCTTTCTTTATATTTTTGTGCTTTTTATTTGTTTCTTTGTTTGTCTGAGACAGA 76060

************************** *********************************

Human GTCTTTCTTTGTTGCCCAGGCTGGAGTACGGTGGTGTGATCACAACTAACTACAGCCTCC 76162

Chimpanzee GTCTTTCTTTGTTGCCCAGGCTGGAGTACGGTGGTGTGATCACAACTAACTACAGCCTCC 85045

Gorilla GTCTTTCTTTGTTGCCCGGGCTGGAGTACGGTGGTGTGATCACAACTAACTACAGCCTCC 76120

***************** ******************************************

Human ACTTCCCAGGCTCAATCAATACTCCTGCCTCAGCCTCTTGAATAGGTGGGATTAGAAGCA 76222

Chimpanzee ACTTCCCAGGCTCAATCAATACTCCTGCCTCAGCCTCTTGAATAGGTGGGATTAGAAGCA 85105

Gorilla ACTTCCCAGGCTCAATCAATCCTCCTGCCTCAGCCTCTTGAATAGGTGGGATTAGAAGCA 76180

******************** ***************************************

Human TTCACCCCCATGCCTACCTAATTTTTCTATTTTTTATCGTGAGATGGGGTCTCACTATAT 76282

Chimpanzee TGCACCCCCATGCCTACCTAATTTTTCTATTTTTTATCGTGAGATGGGGTCTCACTGTAT 85165

Gorilla TGCTCCCCCATGCCTACCTAATTTTTCTATTTTTTATCGTGAGATGGGGTCTCACTATAT 76240

* * **************************************************** ***

Human TGCCCAGGCTGATCTTGAACTCCTAGCCTCAAGCAATCCTCCTACCTCAGCCCTTCAAAG 76342

Chimpanzee TGCCCAGGCTGATCTTGAACTCCTAGCCTCAAGCAATCCTCCTACCTCAGCCCTTCAAAG 85225

Gorilla TGCCCAGGCTGATCTTGAACTCCTAGCCTCAAGCAATCCTCCTACCTCAGCCCTTCAAAG 76300

************************************************************

Human TGCTGGGATTACAAACATGAGCCACCATACCAAGGCAGATCAAGGGTTGTCATGACATTG 76402

Chimpanzee TGCTGGGATTACAAACATGAGCCACCATACCAAGGCAGATCAAGGGTTGTCATGACATTG 85285

Gorilla TGCTGGGGTTACAAACATGAGCCACCATACCAAGGCAGATCAAGGGTTGTCATGACATTG 76360

******* ****************************************************

Human CTTGCAAGTCATGCAAACACATCAGTGACTGAGAGGAAATCAGCTTCTAGAAACCTGAAA 76462

Chimpanzee CTGGCAAGTCATGCAAACACATCAGTGACTCAGAGGAAATCAGCTTCTAGAAACCTGAAA 85345

Gorilla CTTGCGAGTCATGCAAACACATCAGTGACTGAGAGGAAATCAGCTTCTAGAAACCTGAAA 76420

** ** ************************ *****************************

Human GAAATGGGGTAGATAGAAAAGGTCACATTGGGATCACCAGTCATTTTAGGTTGAGAGACA 76522

Chimpanzee GAAATGGGGTAGATAGAAAAGGTCACATTGGGATCACCAGTCATTTTAGGTTGAGAGACA 85405

Gorilla GAAATGGGGTAGATAGAAAAGGTCACACTGGGATCACCAGTCATTTTAGGTTGAGAGACA 76480

*************************** ********************************

Human CTGCATGCCTAAGCTGGTTCTGCAAGCAGGAAGGCCATGATGGAAATCTCTAATCTCAAT 76582

Chimpanzee CTGCATGCCTAAGCTGGTTCTGCAAGCAGGAAGGCCATGATGGAAATCTCTAATCTCAAT 85465

Gorilla CTGCATGCCTAAGCTGGTTCTGCAAGCAGGAAGGCCATGATGGAAATCTCTAATCTCAAT 76540

************************************************************

Human CCCCTGCCATCTTTTGAAATCTTAGTTTAGCCCACATGGAAGCCTCAAGTCAATGGATCC 76642

Chimpanzee CCCCTGCCATCTTTTGAAATCTTAGTTTACCCCACATGGAAGCCTCAAGTCAATGGATCC 85525

Gorilla CCCCTGCCATCTTTTGAAATCTTAGTTTAACCCACATGGAAGCCTCAAGTCAATGGATCC 76600

***************************** ******************************

Human TATTATTATACAAAACTCAATCCTGGGCACAGGGTAGGGTGGAGAGACATAAACAGTTGT 76702

Chimpanzee TATTATTATACAAAACTCAATCCTGGGCACAGGGTAGGGTGGAGAGACATAAACAGTTGT 85585

Gorilla TATTATTATACAAAACTCAATCCTGGGCACAGGGTAGGGTGGAGAGACATAAACAGTTGT 76660

************************************************************

Human GGCAGAATCTATGCTCTGCAGTCACTCTACTCATCCATTCAACTTCACACTGCCTTCGTA 76762

Chimpanzee GGCAGAATCTATGTTCTGCAGTCACTCTACTCATCCATTCAACTTCACACTGCCTTCGTA 85645

Gorilla GGCAGAATCTATGTTCTGCAGTCACTCTACTCATCCATTCAACTTCACACTGCCTTCGTA 76720

************* **********************************************

Human AGGATCATCTGAGGTGATTTTAGTATAATATCAGCATCTGGATGGAAAAGAGTGGGTCAG 76822

Chimpanzee AGGATCATCTGAGGTGATTTTAGTATAATATCAGCATCTGGATGGAAAAGAATGGGTCAG 85705

Gorilla AGGATCATCTGAGGTGATTTTAGTATAATAACAGCATCTGGATGGAAAAGAATGGGTCAG 76780

****************************** ******************** ********

Human GCAGGAGACCCAGTTCTCTTTTATTAGTCTAGCAAGAATCCATAATTGTTTCCCACTCCT 76882

Chimpanzee GCAGGAGACCCAGTTCTCTTTTATTAGTCTAGCAAGAATCCATAATTGTTTCCCACTCCT 85765

Gorilla GCAGGAGACTCAGTTCTCTTTTATTAGTCTAGCAAGAATCCATAATTGTTTCCCACTCCT 76840

********* **************************************************

Human GACAAACTTGGGACAAAATCCTTCTTCTCCTTTGAACTCCAAACACTGACCCTATCTATT 76942

Chimpanzee GACAAATTTGGGACAAAATCCTTCTTCTCCTTTGAACTCCAAACACTGACCCTATCTATT 85825

Gorilla GACAAATTTGGGACAAAATCCTTCTTCTCCTTTGAACTCCAAACACTGACCCTATCTATT 76900

****** *****************************************************

Human ATTCAGTTGTGACTCAATATAAACCACAGCACCGTGACAGTCTTATTGTCTAGGTTCAGG 77002

Chimpanzee ATTCAGTTGTGACTCAGTATAAACCACAGCACCGTGATAGTCTTATTGTCTAGGTTCAGG 85885

Gorilla ATTCAGTTGTGACTCAATATAAACCACAGCAACGTGATAGTCTTATTGTCTAGGTTCAGG 76960

**************** ************** ***** **********************

Human CAAAAGTTTCAGATTTATCCACTTGCATATATATCTCTGTGTTTTTCTCTTATCTCTCTA 77062

Chimpanzee CAAAAGTTTCAGATTTATCCACTTGCATATATATCTGTGTGTTTTTCTCTTATCTCTCTA 85945

Gorilla CAAAAGTTTCAGATTTATCCACTTGCATATATATATGTGTGTTTTTCTCTTATCTCTCTA 77020

********************************** * ***********************

Human ATAGCATTATTTAAATGTGGTGGATTTGATGTTAGTCCCTTGGTCTCTCATTTTAGTAGC 77122

Chimpanzee ATAGCATTATTTAAATGTGGTGGATTTGATGTTAGTCCCTTGGTCTCTCACTTTAGTAGC 86005

Gorilla ATAGCATTATTTAAATGTGCTGGATTTGATGTTAGTCCCTTGGTCTCTCACTTTAGTAGC 77080

******************* ****************************** *********

Human TGGATGTAACATTAAGGTAAATTACTGAGTGTGTCTAGATCTGCATTTCATCACAAGATA 77182

Chimpanzee TGGATGTAACATTAAGGCAAATTACTGAGTGTGTCTAGATCTGCATTTCATCACAAGATA 86065

Gorilla TGGACGTAACATTAAGGTAAATTACTGAGTGTGTCTAGATCTGCATTTCATCACAAGATA 77140

**** ************ ******************************************

Human GCACTAGTATTTATGCAAATTTCCAATTATATATTGCTAGGTTCTAAATGTGTGTACCTT 77242

Chimpanzee GCACTAGTATTTATGCAAATTTCCAATTATATATTGCTAGGTTCTAAATGTGTGTACCTT 86125

Gorilla GCACTAGCATTTATGCAAATTTCCAATTATATATTGCTAGTTTCTAAATGTGTGTACCTT 77200

******* ******************************** *******************

Human CTGAATTTTTTTT-GTTGAAGACTTAATCATCAAATTGATGGCATTATGAGATGAGGTGT 77301

Chimpanzee CTGAATTTTTTTTTGTTGAAGACTTAATCATCAAATTGATGGCATTATGAGATGAGGTGT 86185

Gorilla CTGAATTTTTTTN-GTTGAAGACTTAATCATCAAATTGATGGCATTATGAGATGAGGTGT 77259

************ **********************************************

Human TTGGAAAGTGATTAAATCATGAGGACAGAACCCTCATGAAGATTTGTATCCTTATAAAAG 77361

Chimpanzee TTGGAAAGTGATTAAATCATGAGGACAGAACCCTCATGAAGATTTGTATCCTTATAAAAG 86245

Gorilla TTGGAAAGTGATTAAATCATGAGGACAGAACCCTCATGGAGATTTGTATCCTTAAAAAAG 77319

************************************** *************** *****

Human AAGAAACAACCGGAAGTCACCATCTATGTACAGAAAATTGGCCCTCACCAGAAATTCAAT 77421

Chimpanzee AAGAAACAACCGGAAGTCACCATCTATGTACAGAAAATTGGCCCTCACCAGAAATTCAAT 86305

Gorilla AAGAAACAACCGGAAGTCACCGTCTATGTACAGAAAATTGGCCCTCACCAGAAATTCAAT 77379

********************* **************************************

Human CAGCATTACTCTTGCACTTTCCAGACTCCTGAATTATAAGAAACAAATTTTTATTGTTGA 77481

Chimpanzee CAGCATTACTCTTGCACTTTCCAGACTCCTGAATTATAAGAAACAAATTTTTATTGTTGA 86365

Gorilla CAGCATTACTCTTGCACTTTCCAGACTCNTGNANNNNNNNNNNNNNNNNNNNNNNNNNGA 77439

**************************** ** * **

Human TAAGCCACCCATTTTATGCCTGAAACACATTTGTATTTAACATGTTAGCAAGGGATAAGA 77541

Chimpanzee TAAGCCACCCATTTTATGCCTGAAACACATTTGTATTTAACATGTTAGCAAGGGATAAGA 86425

Gorilla TAAGCCACCCATTTTATGCCTGAAACACATTTGTGTTTAACATGTTAGCAAGGGATAAGA 77499

********************************** *************************

Human TCCCAGGTGTACTCAATAAGTATTAAGGCGAAAGAACAGCACTCAATGGTAAATTCTTAT 77601

Chimpanzee CCCCAGGTGTACTCAATAAGTATTAAGGCGAAAGAACAGCACTCAATGGTAAATTCTTAT 86485

Gorilla TNNNNNNNNNNNNNNNNNAGTATTAAGGCGAAAGAACAGCACTCAATGGTAAATTCTTAT 77559

******************************************

Human CATTGATGTTTCATTAATAGTTCTTAATCAAATGCTAACTTTAGGTCTCCATGTCTAAAT 77661

Chimpanzee CATTGATGTTTCATTAATAGTTCTTAATCAAATGCTAACTTTAGGTCTCCATGTTTAAAT 86545

Gorilla CACTGATGTTTCATTAATAGTTCTCAATCAAATGCTAACTTTAGGTCTCNNNNNNNNNNN 77619

** ********************* ************************

Human AACTAAACCCCAATAAAAACCCTGGACATCAAGTCTCAGGTGAGCCTCTCTAGTCAACAA 77721

Chimpanzee AACTAAACCCCAATAAAAACCCTGGACATCAAGTCTCAGGTGAGCCTCTCTAGTCAACAA 86605

Gorilla NNNNNNNNNNNNNNNNNNNNNNNNNNNNNCAAGTCTCAGGTGAGCCTCTCTAGTCAACAA 77679

*******************************

Human TGCTTCGTAAGTATTGTAATACATTGTTGATGTCATACATTGTTGATGTTACACATTGTT 77781

Chimpanzee TGCTTCATAAGTATTGTAATACATTGTTGATGTCAAACATTGTTGATGTTACACATTGTT 86665

Gorilla TGCTTCGTAAGTATTGTAATACGTTGTTGATGTGATACATTGTTGATGTTACACATTGTT 77739

****** *************** ********** * ************************

Human TCTTATAAGTCAAATCAAAGGGGACAACTTGTGCCTTGTTTCCTGAACTCAGCCATATGC 77841

Chimpanzee GCTTATAAGTCAAATCAAAGGGGACAACTTGTGCCTTGTTTCCTGAACTCAGCCATATGC 86725

Gorilla GCTTATAAGTCAAATCAAAGGGGACAACTTGTGCCTTGTTTCCTGACCTCAGCCATATGC 77799

********************************************* *************

Human CCCTTTTCCTTTGTTGATTCTAACCTGTAACCTTTCACTGTAATCATCCATAACCATTAA 77901

Chimpanzee CCCTTTTCCTTTGTTGATTCTAATCTGTAACCTTTCCCTGTAATCATCCATAACCATTAA 86785

Gorilla CCCCTTTTCTTTGTTGATTGTAATCTGTAACGTTTCACTGTAATCATCCATAACCATTAA 77859

*** *** *********** *** ******* **** ***********************

Human CATGATAGATTTTCTGGGTACCATAAGTTCTCCAGCATATCATCCTACCTGAGGATGGGC 77961

Chimpanzee CATGATAGATTTTCTGGGTACCATAAGTTCTCCAGCATATCATCCTACCTGAGGATGGGC 86845

Gorilla CATGATAGATTTTCTGGGTACCATAAGTTCTCCAGCAAATCATCCTACCTGCGGATGAGC 77919

************************************* ************* ***** **

Human TGACACAATAACCAACTAGAAAATTTGAAATTTGCAAAAATGTTTAATGATCTTAATTAA 78021

Chimpanzee TGACACAATAACCAACTAGAAAATTTGAAATTTGCAAAAATGTTTAATGATCTTAATTAA 86905

Gorilla TGACACAATAACCAACCAGAAAATTTGAAATTTGCAAAAATGTTTAATGATCTTGATTAA 77979

**************** ************************************* *****

Human AACTAAGCATTAACCTCAAGAAAACATTAAAGATTATCTTCTTCGCATTTAAATTCTGGA 78081

Chimpanzee AACTAAGCATTAACCTCAAGAAAACATTAAAGATTATCTTCTTTGCATTTAAATTCTGGA 86965

Gorilla AACTAAGCATTAACCTCAAGAAAACATTAAAGATTATCTTCTTCGCATTTAAATTCTGGA 78039

******************************************* ****************

Human TTAATTTTAATACGAGGTTCCCTGTAAATATTCATTCTCTGTCTATTCAAAGAGGGTAGT 78141

Chimpanzee TTAATTTTAATATGAGGTTCCCTGTAAATATTCATTCTCTGTCTATTCAAAGAGGGTAGT 87025

Gorilla TTAATTTTAATATGAGGTTCCCTGTAAATATTCATTCTCCGTCTATTCAAAGAGGGTAGT 78099

************ ************************** ********************

Human TAATTTTTACTGATTCCATTGCAAACTCACAT-AAAATATTTCTATACTTAGTGAACATA 78200

Chimpanzee TAATTTTTACTGATTCCATTGCAAACTCACATTAAAATATTTCTATACTTAGTGAACATA 87085

Gorilla TAATTTTTACTGATTCCATTGCAAACTCACAT-AAAATATTTCTATACTTAGTGAACATA 78158

******************************** ***************************

Human TACAGTAGAGATTCTGTTTTGTGTATCCAAATCATTTT-ATGAAATAAATCTTCTTTACA 78259

Chimpanzee TACAGTAGAGATTCTGTTTTGTGTATCCAAATCATTTTTATGAAATAAATCTTCTTTACA 87145

Gorilla TACAGTAGAGATTCTGTTTTGTGTATCCAAATCATTTT-ATGAAATAAATCTTATTTACA 78217

************************************** ************** ******

Human TATTTATCTCAATTGCCAACTTATTGGTTATAGTTCATATGCCATGCAGTACTCTGTATT 78319

Chimpanzee TATTTATCTCAATTGCTAACTTATTGGTTATAGTTCATATGCCATGCAGTACTCTGTATT 87205

Gorilla TATTTATTTCAATTGCTAACTTATTGGTTATAGTTCATATGCCATGCAGTACTCTGTATT 78277

******* ******** *******************************************

Human AGAAATAACAGATTATTCTTTGACAAACCTGAAAAAAACAAGCAATGGGAAAGGATTCT- 78378

Chimpanzee AGAAATAACAGATTATTCTTTGACAAACCTGAAAAAAACAAGCAATGGGGAAGGATTCTT 87265

Gorilla AGAAGTAACAGATTATTCTTTGACAAACCTGAAAAAAACAAGCAACGGGAAAGGATTNN- 78336

**** **************************************** *** *******

Human CTATTTAACAAGTGGTGCTGGGAAACCTGGCTAGACATATGCAGCAAGCTGAAACTGGAT 78438

Chimpanzee CTATTTAATAAGTGGTGCTGGGAAAACTGGCTAGACATATGCAGCAAGCTGAAACTGGAT 87325

Gorilla NNNNNNNNNNNNNNNNNNNNNNNNNNNTGGCTAGACATATGCANNNNNNNNNNNNNNNNN 78396

****************

Human CCCTTCCTTACACCTTATACAAAAATTAATTCAAGATGGATTAAAGACTTAAACATAACA 78498

Chimpanzee CCCTTCCTTACACCTTATACAAAAATTAATTCAAGATGGATTAAAGACTTAAACATAATA 87385

Gorilla NNNNNNNNNNNNCCTTANNNNNNNNNNNNNNNNAGATGGATTNNNNNNTTAANNNNNNNN 78456

***** ********* ****

Human CCTAAAACCATAGAAACGCTAGAAGAAAACCTAGGCAAAACCATTGAGAACATTGGCATG 78558

Chimpanzee CCTAAAACCATAGAAACCCTAGAAGAAAACCTAGGCAAAACCATTGAGAACATTGGCATG 87445

Gorilla NNNNNNNNCNTAGAAACCCTAGAAGAAAACCTAGGCAAAACCATTGAGAACATTGGCATG 78516

* ******* ******************************************

Human GGCAAGGTCTTCATGACTAAAACACTGAAAGTAATGGCAACAAAAGCCAAAATTGACACA 78618

Chimpanzee GGCAAGGTCTTCATGACTAAAACACTGAGAGTAATGGCAACAAAAGCCAAAATTGACACA 87505

Gorilla GGCAAGGTCTTCATGACTAAAACACTGAAAGTAATGGCAACAAAAGCCAAAATTGACACG 78576

**************************** ******************************

Human TACAGTCTGATTAAACCAAAGAGCTTCTGCACAGCAAAATAAACTATCATCAGAGTGAAG 78678

Chimpanzee TACAGTCTGATTAAACCAAAGAGCTTCTGCACAGCAAAAGAAACTATCATCAGAGTGAAG 87565

Gorilla TACAGTCTGATTAAACCANNNNNNNNNTGCACAGCAAAAGAAACTATCATCAGAGTGAAG 78636

****************** ************ ********************

Human AAGCAACCTACAGATTGGGAGACAATTTTTGCAATCTATCCATCTGACAAAGGGCCAATA 78738

Chimpanzee AAGCAACCTACAGATTGGGAGAAAATTTTTGCAATCTATCCATCTGACAAAGGGCCAATA 87625

Gorilla AAGCAACCTACAGATTNNGAGNNNNNNNNNNNNNNNNNNNNNNNNNACAAAGGGCCAATA 78696

**************** *** **************

Human TTCAGAATATACAAAGAACTTAAACAGATTTACAAGAAAAAAAGTAACCCATCAAAAAAC 78798

Chimpanzee TTCAGAATATACAAAGAAATTAAACAGATTTACAAGAAAAAAAGCAACCCATCAAAAAAC 87685

Gorilla TTCAGAATATACAAAGAACTTAAACAGATTTACAAGAAAAAAAGTAACCCATCAAAAAAC 78756

****************** ************************* ***************

Human GGGTGAAGGATATGAACAGACATTTATCAAAAGAAGACATTTATGCAGCCAACAAACATG 78858

Chimpanzee GGGTGAAGGATATGAACAGACATTTATCAAAAGAAGACATTTATGCAGCCAACAAACATG 87745

Gorilla GGGTGAAGGATGTGAANAGACACTTATCAAAAGAAGACATTTATGCAGCCAACAAATATG 78816

*********** **** ***** ********************************* ***

Human AAAAACAGCTCATCATCATGGGTCATTAGAAAAATCCAAGTCAAAACCATCTCACACCAG 78918

Chimpanzee AAAAACAGCTCATCATCACGGGTCATTAGAAAAATCCAAGTCAAAACCATCTCACACCAG 87805

Gorilla ANNNNNNNNNNNNNNNNNNNNNNNNNNNNNNNNNTCCAAGTGAAAACCATCTCACACCAG 78876

* ******* ******************

Human TTAGAATGGCAATCGTTAAAAAGTCAGGAAACAACAGATGCTGGGGAGGGTGTGGAGAAA 78978

Chimpanzee TTAGAATGGCAATCATTAAAAAGTCAGGAAACAACAGATGCTAGGGAGGGTGTGGAGAAA 87865

Gorilla TTAGAATGGCNNNNNNNNNNNNNNNNNNNNNNNNNNNATGCTGGGGAGGGTGTGGAGAAA 78936

********** ***** *****************

Human TAGGAATGTGTTTACACTGTTGGTTGGAGTGTAGATTATTCAATCATTGTGGAAGACAGT 79038

Chimpanzee TAGGAATGTGTTTACACTGTTGGTTGGAGTGTAGATTATTCAATCATTGTGGAAGACAGT 87925

Gorilla TAGGAATGTGTTTACACTGTTGGTTGGAGTGTAAATTATTCAATCATTGTGGAAGACAGT 78996

********************************* **************************

Human GTGACAATTCCTCAAGTATCTAGAACCAGAAATACCATTGAACCCAGCAATCCCATTACT 79098

Chimpanzee GTGACAATTCCTCAAGTATCTGGAACCAGAAATACCATTGAACCCAGCGATCCCATTACT 87985

Gorilla GTGACAATTCCTCAAGTATCTAGAACCAGAAATACCATTGAACCCAGCAATCCCATTACT 79056

********************* ************************** ***********

Human GGATATATACCCAAAGGATTTTAAATCACTC---TATAGAGACATGCACATGTATGTTTA 79155

Chimpanzee GGATATATACCCAAAGGATGTTAAATCACTCGACTATAGAGTCATGCACATGTATGTTTA 88045

Gorilla GGATATATACCCAAAGGATTTTAAATCACTC---TATAGAGACATGCACATGTATGTTTA 79113

******************* *********** ******* ******************

Human TTGCTGCACTGTTCACAAAAGGAAAGATGTGGAGCCAACACAAATGCCTGTCAATGATAG 79215

Chimpanzee TTGCTGCACTGTTCACAAAAGGAAAGATGTGGAACCAACACAAATGCCTGTCAATGATAG 88105

Gorilla TTGCTGCACTGTTCACAAAAGGAAAGATGTGGAACCAACACAAATGCCTGTCAATGATAG 79173

********************************* **************************

Human ACTGGATAAAGAAAATGTGGCAATATACACCATGGAATACTATGCAGCCATGAAAAAGGA 79275

Chimpanzee ACTGGATAAAGAAAATGTGGCAATACACACCATGGAATACTATGCAGCCATGAAAAAGGA 88165

Gorilla ACTGGATAAAGAAAATGTGGCAATATACACCATGGAATACTATGCAGCCATGAAAAAGGA 79233

************************* **********************************

Human TGAGTTCATGTCCTTTGCAGGGACACGGATGATGCTGAAATCCATCATTCTCAGCAAACT 79335

Chimpanzee TGAGTTCATGTCCTTTGCAGGGACACGGATGATGCTGAAATCCATCATTCTCAGCAAACT 88225

Gorilla TGAGTTCGTGTCCTTTGCAGGGACACGGATGATGCTGAAATCCATCATTCTCAGCAAACT 79293

******* ****************************************************

Human AACACAAGACAGAAAACCAAACACCAGATGTTCTCACTCGTAAATGAGAGCTGAACAATG 79395

Chimpanzee AACACAAGACAGAAAACCAAACACCAGATGGTCTCACTCGTAAATGAGAGCTGAACAATG 88285

Gorilla AACACAAGACAGAAAACCAAACACCAGATGTTCTCACTCGTAAATGAGAGCTGAACAATG 79353

****************************** *****************************

Human AGAACACATAGAGGGGAACATCACACATCAAAGCCTGTCTGGAGTTGGGGGACTAGGGGA 79455

Chimpanzee AGAACACATGGAGGGGAACATCACACATCAAAGCCTGTCTGGAGTCGGGGGACTAGGGGA 88345

Gorilla AGAACACATGGAGGGGAACATCACACATCAAAGCCTGTCTGGAATTGGGGGACTAGGGGA 79413

********* ********************************* * **************

Human AGGACAGCATTCAGAGAAATACCTAATGTAGATGATGGATTGATGGGTGAAGCAAACCAC 79515
[truncated: 354,990 more chars]
