# Supplementary figures and images for: Recombination Dynamics of a Human Y-Chromosomal Palindrome: Rapid GC-Biased Gene Conversion, Multi-kilobase Conversion Tracts, and Rare Inversions
Source: PLoS Genet. 2013 Jul 25;9(7):e1003666. doi: 10.1371/journal.pgen.1003666 (PMC3723533; doi:10.1371/journal.pgen.1003666)

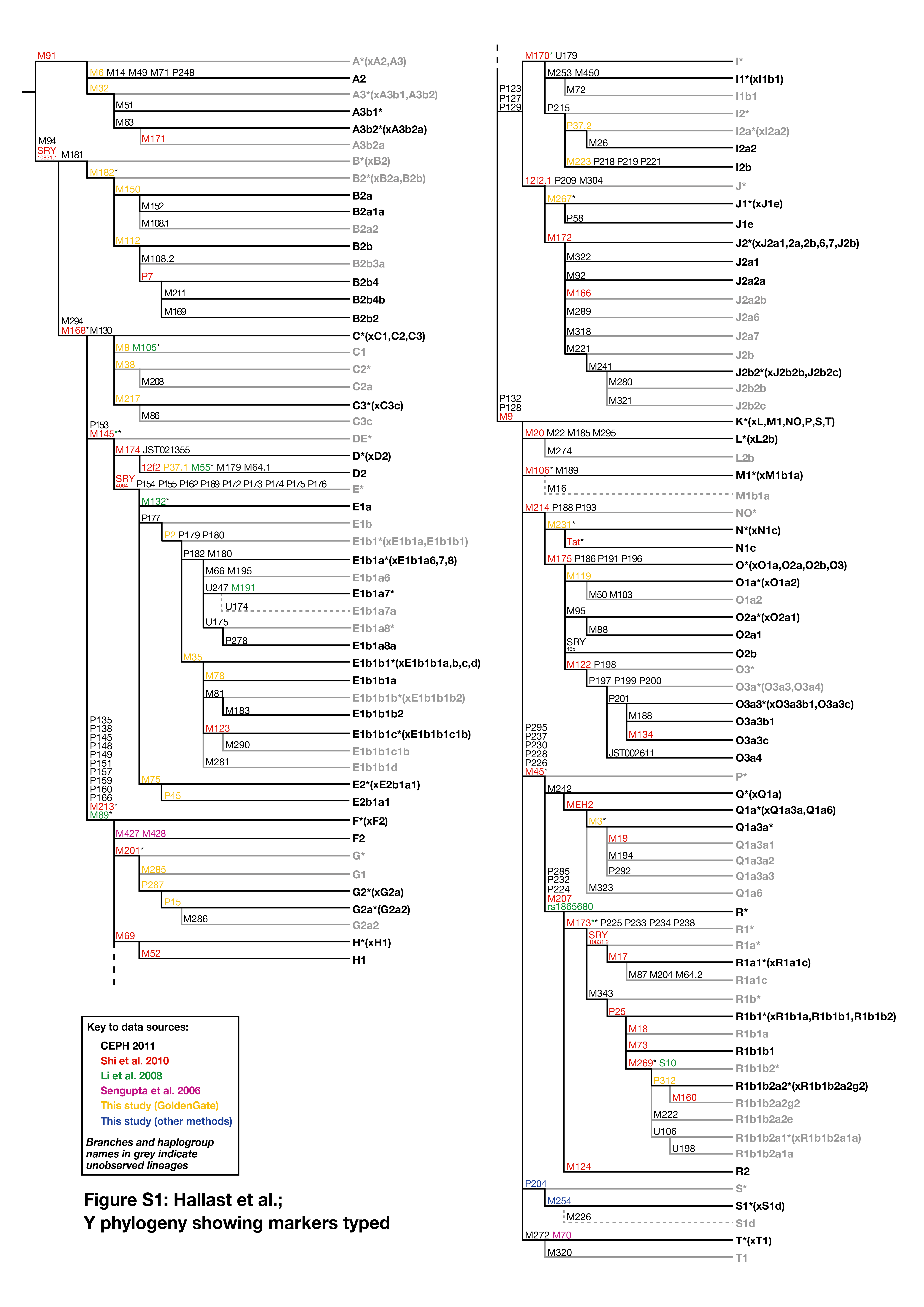

Supplement: Figure S1 — Y phylogeny, showing markers typed and data sources. (TIF) [file pgen.1003666.s002.tif]
