# Supplementary material for: Recombination Dynamics of a Human Y-Chromosomal Palindrome: Rapid GC-Biased Gene Conversion, Multi-kilobase Conversion Tracts, and Rare Inversions
Source: PLoS Genet. 2013 Jul 25;9(7):e1003666. doi: 10.1371/journal.pgen.1003666 (PMC3723533; doi:10.1371/journal.pgen.1003666)
Supplement: Protocol S1 — Sequencing and analysis of gorilla Y-chromosome palindrome, containing summary of NGS sequence data and coverage. (DOCX) [file pgen.1003666.s009.docx]

**Protocol S1.**

**Sequencing and analysis of gorilla Y-chromosome palindrome P6**

RNA baits for Agilent SureSelect (Agilent Technologies Inc., CA, USA) custom target enrichment were designed using Agilent eArray with default parameters for Illumina Paired-End Long Read sequencing (Bait length: 120 bp; Design strategy: centered; Tiling frequency: 1X; Avoid overlap: 20 bp; Strand: sense; Avoid regions: Repeat Masker) and human reference sequence hg19/GRCh37 (February 2009). As the arms of the palindrome are >99.9% identical, baits were designed for one arm (proximal), spacer and 1 kb of flanking sequence using the following genomic coordinates: chrY:18270274-18427656 and chrY:18537677-18538845. Repeats identified by Repeat Masker were avoided and as a result only 26.8% of the palindrome sequence was suitable for bait design. Boosting was used for ‘orphan’ (located >20 bp from flanking baits) and GC-rich (GC content ≥63%) baits by direct replication (orphans 2X, GC-rich 3X).

3 μg of genomic DNA from a male gorilla GoM5 (*Gorilla gorilla*) was used for library preparation and target enrichment using Agilent SureSelect^XT^ Target Enrichment System for Illumina Paired-End Sequencing Library kit (Version 1.2) according to manufacturer’s recommendations. Sequencing was done on an Illumina HiSeq 2000 instrument (Illumina, CA, USA) with paired-end 100-bp run to high coverage. Library preparation, target enrichment and sequencing were carried out in the GenePool genomics facility at the University of Edinburgh (Edinburgh, UK).

Sequence data were mapped to human genome reference (hg19/GRCh37) using Stampy v1.0.13 [[1](#_ENREF_1)]. The distal arm of P6 was masked (replaced with poly[N]) in the reference sequence to simplify analysis. Local realignment was done using The Genome Analysis Toolkit (GATK) v1.4-9 [[2](#_ENREF_2),[3](#_ENREF_3)], followed by read duplicate removal with picard v1.59 (<http://picard.sourceforge.net>).

Low-coverage whole-genome paired-end Illumina sequence data from two additional male gorillas Kwanza and Mukisi was received from Aylwyn Scally in bam format for chromosomes X and Y, with human sex chromosome sequences used as reference [[4](#_ENREF_4)]. Reads mapped to palindrome P6 plus 10 kb flanking sequence were extracted using samtools v0.1.17 [[5](#_ENREF_5)], and the resulting bam was converted to fastq with bam2fastq v1.1.0 (<http://www.hudsonalpha.org/gsl/software/bam2fastq.php>). Generated fastq reads were re-mapped to human reference sequence (with the distal arm of P6 masked) using Stampy. Local realignment and duplicate removal were performed as for the enrichment data described above.

Base calling was done with GATK UnifiedGenotyper multi-sample calling option which simultaneously used data from all three gorillas with the following parameters: genotype_likelihoods_model: SNP; min_base_quality_score: 20; genotyping_mode: DISCOVERY; and output_mode: EMIT_ALL_CONFIDENT_SITES. Obtained list of raw calls was filtered using GATK VariantFiltration tool with the following parameters: filterExpression DP>4 and MQ>30.00. As a result, only bases covered by at least 5 independent reads and mapping quality higher than 30 were retained in the final sequence. To be conservative, any discordant sites found between gorilla samples were replaced with ‘N’ and therefore treated as missing data in all consecutive analysis. For summary of sequence data used, see Table 1.

**Table 1. Sequence data used to assemble gorilla palindrome P6**

| Sample | Read length | Total no of reads | Mean coverage of P6 region* | Mean coverage of P6 proximal arm | Mean coverage of P6 spacer |
| --- | --- | --- | --- | --- | --- |
| GoM5 | 100 bp | 146,681 | 71.72 | 80.53 | 50.54 |
| Kwanza** | 35-37 bp | 27,024 | 3.37 | 3.97 | 1.91 |
| Mukisi** | 37-54 bp | 7984 | 2.14 | 2.55 | 1.16 |

* - P6 proximal arm, spacer and 1 kb of flanking sequence

** - sequence data from ref. 4.

**References**

1. Lunter G, Goodson M (2011) Stampy: a statistical algorithm for sensitive and fast mapping of Illumina sequence reads. Genome Res 21: 936-939.

2. DePristo MA, Banks E, Poplin R, Garimella KV, Maguire JR, et al. (2011) A framework for variation discovery and genotyping using next-generation DNA sequencing data. Nat Genet 43: 491-498.

3. McKenna A, Hanna M, Banks E, Sivachenko A, Cibulskis K, et al. (2010) The Genome Analysis Toolkit: a MapReduce framework for analyzing next-generation DNA sequencing data. Genome Res 20: 1297-1303.

4. Scally A, Dutheil JY, Hillier LW, Jordan GE, Goodhead I, et al. (2012) Insights into hominid evolution from the gorilla genome sequence. Nature 483: 169-175.

5. Li H, Handsaker B, Wysoker A, Fennell T, Ruan J, et al. (2009) The Sequence Alignment/Map format and SAMtools. Bioinformatics 25: 2078-2079.
